# Fatty acid metabolism-related lncRNAs are potential biomarkers for survival prediction in clear cell renal cell carcinoma

**DOI:** 10.1097/MD.0000000000037207

**Published:** 2024-02-23

**Authors:** Ming-Qing Zhang, Bai-Zhi Yang, Zhi-Qiang Wang, Shanchun Guo

**Affiliations:** a Department of Urology, Weifang Pepole’s Hospital, Weifang, Shandong, China; b Department of Urology, Shouguang Hospital of Traditional Chinese Medicine, Shouguang, China; c Department of Urology, Shouguang Hospital of Traditional Chinese Medicine, Shouguang, China; d RCMI Cancer Research Center, Xavier University of Louisiana, New Orleans, LA.

**Keywords:** clear cell renal cell carcinoma, fatty acid metabolism, lncRNAs, prognosis

## Abstract

Metabolic reprogramming of energy is a newly recognized characteristic of cancer. In our current investigation, we examined the possible predictive importance of long noncoding RNAs (lncRNAs) associated to fatty acid metabolism in clear cell renal cell carcinoma (ccRCC). We conducted an analysis of the gene expression data obtained from patients diagnosed with ccRCC using the Cancer Genome Atlas (TCGA) database and the ArrayExpress database. We performed a screening to identify lncRNAs that are differentially expressed in fatty acid metabolism. Based on these findings, we developed a prognostic risk score model using these fatty acid metabolism-related lncRNAs. We then validated this model using Cox regression analysis, Kaplan–Meier survival analysis, and principal-component analysis (PCA). Furthermore, the prognostic risk score model was successfully validated using both the TCGA cohort and the E-MTAB-1980 cohort. We utilized gene set variation analysis (GSVA) and gene set enrichment analysis (GSEA) to determine the correlation between fatty acid metabolism and the PPAR signaling pathway in patients with ccRCC at various clinical stages and prognoses. We have discovered compelling evidence of the interaction between immune cells in the tumor microenvironment and tumor cells, which leads to immune evasion and resistance to drugs. This was achieved by the utilization of advanced techniques such as the CIBERSORT method, ESTIMATE R package, ssGSEA algorithm, and TIMER database exploration. Ultimately, we have established a network of competing endogenous RNA (ceRNA) that is related to fatty acid metabolism. The findings of our study suggest that medicines focused on fatty acid metabolism could be clinically significant for individuals with ccRCC. The utilization of this risk model, which is centered around the lncRNAs associated with fatty acid metabolism, could potentially provide valuable prognostic information and hold immunotherapeutic implications for patients with ccRCC.

## 1. Introdution

Renal cell carcinoma (RCC) is a prevalent malignant tumor of the urinary system that mostly arises from renal tubular epithelial cells. It constitutes around 2% to 3% of all adult malignancies.^[1]^ The prevalence of RCC is rising by 2% annually in developed nations.^[2]^ The clear cell renal cell carcinoma (ccRCC) is a prevalent histopathologic subtype, making up 80% to 90% of all RCC.^[3]^ Approximately 30% of patients with ccRCC were already in an advanced stage due to the absence of distinctive clinical signs.^[4–7]^ Approximately 40% of patients continue to encounter disease recurrence, primarily within the initial 5 years following surgery. Furthermore, patients with ccRCC exhibit notably inferior 5-year recurrence-free survival (RFS) rates in comparison to patients with other forms of renal cell carcinoma (RCC).^[8]^ ccRCC exhibits a highly aggressive biological behavior, resulting in a dismal prognosis^[9]^{Kim, 2021 #99}. Hence, there is a pressing requirement for novel clinical indicators for ccRCC patients.

Energy metabolic reprogramming is a newly recognized characteristic of cancer that is becoming increasingly important.^[10,11]^ Abnormal fatty acid (FA) metabolism has been detected in various cancer forms, such as RCC, breast cancer, gastric cancer, prostate cancer, and lung cancer.^[12–18]^ Notably, the TCGA analysis of ccRCC revealed that metabolic changes play a crucial role in the evolution of ccRCC.^[14,19,20]^ A study and its subsequent analysis revealed a correlation between poorer survival of ccRCC patients and the increased expression of genes related to the pentose phosphate pathway and fatty acid synthesis pathway, as well as the decreased expression of genes related to the TCA cycle.^[21]^ The significance of FA metabolism in cancer cell functioning, as well as the Warburg effect (perturbed glucose metabolism) and altered amino acid metabolism (specifically glutamine metabolism), is gaining recognition. These discoveries may reveal the molecular mechanisms responsible for lipid accumulation in ccRCC.^[22]^ Du et al discovered that abnormal cancer metabolism causes alterations in FA outcomes, such as a shift in ccRCC towards excessive lipid storage. They also found the crucial role of HIF in controlling FA metabolism for ccRCC carcinogenesis.^[23]^ ccRCC cells exhibit characteristics of malignant epithelial cells, displaying transparent cytoplasm as a result of abundant lipid and glycogen accumulations.^[23,24]^ Understanding the process of FA metabolism and the molecular mechanism of ccRCC will enable the discovery of new prognostic markers and treatment targets.^[12,22]^ Nevertheless, the regulation mechanism of the FA metabolic pathway in ccRCC has not been thoroughly investigated. Hence, the discovery of genes associated with FA metabolism could have substantial predictive value in diagnosing ccRCC and assisting in clinical decision-making.

Long noncoding RNAs (lncRNAs) are RNA transcripts that are above 200 nucleotides in length and do not code for proteins. They play a role in gene regulation and numerous cellular processes.^[25,26]^ LncRNA dysregulation is prevalent in cancer and has been demonstrated to facilitate carcinogenesis and progression.^[27,28]^ Several researchers have investigated the expression profiling of lncRNA in ccRCC and have discovered that they can function as biomarkers for the diagnosis and prognosis of ccRCC.^[29–32]^ Recent investigations have shown that lncRNAs play a role in controlling the expression of important genes, as well as networks of genes involved in processes related to cholesterol and FA production, reverse cholesterol transport, and lipid storage.^[33–35]^ LncRNAs also seem to selectively affect many transcription factors that have crucial functions in controlling lipid metabolism, including liver X receptors,^[36]^ sterol regulatory element binding proteins,^[37,38]^ and peroxisome proliferator-activated receptor α (PPARα).^[39]^ The Salmena et al introduced the competitive endogenous RNA (ceRNA) theory, which is grounded on a comprehensive regulatory network system that illustrates the intricate communication between coding and non-coding RNAs.^[40]^ The ceRNA hypothesis suggests that lncRNAs can influence the production of messenger RNAs (mRNAs) relevant to fatty acid (FA) metabolism by acting as sponges for microRNAs (miRNAs) in ccRCC. While several studies have established ceRNA networks in ccRCC,^[29–32]^ the specific regulatory role of FA metabolism-related ceRNA is still not well understood. This study conducted a detailed analysis of the genomic information from 539 ccRCC samples to evaluate the pattern of FA metabolism and develop a predictive risk score model for FA. The prognostic risk score model accurately predicted the survival result of ccRCC patients, regardless of other factors. Furthermore, an examination was conducted on the correlation between the predictive risk score model and the features of cells infiltrating the tumor microenvironment. The findings indicate that the metabolism of FA plays a crucial role in determining the unique characteristics of the tumor microenvironment in individuals. These findings offer a new viewpoint for investigating the metabolic mechanism and management of ccRCC patients.

## 2. Methods

### 2.1. Data processing

The High Throughput Sequencing (HTSeq)-fragments per kilobase of transcript per million mapped reads (FPKM) workflow type was used to download the raw RNA sequencing (RNA-seq) data profiles from the TCGA database (https://www.cancer.gov/about-nci/organization/ccg/research/structural-genomics/tcga). The data includes 539 samples of renal clear cell carcinoma and 72 samples of normal kidney tissue. The TCGA database provided the clinical data of 537 ccRCC samples, encompassing information such as gender, age, pathological stage, AJCC TNM stage, and prognostic details. We obtained the E-MTAB-1980 dataset from the ArrayExpress database (https://www.ebi.ac.uk/arrayexpress/) for use as an external validation cohort.^[41]^ The 309 genes associated with FA metabolism were obtained from previously published investigations (Table S1, Supplemental Digital Content, http://links.lww.com/MD/L731).^[42]^ Subsequently, a total of 283 genes that were common to both the TCGA and E-MTAB-1980 cohorts were selected. Due to the utilization of an online database, ethical approval and patient permission were not necessary for the studies.

### 2.2. Enrichment analysis of the differentially expressed genes (DEGs) in normal and cancer tissue samples

Using Pearson’s correlation analysis, we discovered 2581 FAM-related lncRNAs. These lncRNAs were then utilized to create a coexpression network using the “ggalluvial” packages in the R software. We selected lncRNAs with a Pearson’s correlation coefficient (|Pearson R|) >0.5 and a *P* value (*P*) < .01. The “limma” R program was utilized to examine the differential expression of lncRNAs related to FA metabolism in both normal and cancerous tissue samples. Genes with a false discovery rate (FDR) of < 0.05 were deemed to be statistically significant. The R package “org.Hs.e.g..db” was subsequently employed to transform the gene symbol of each differentially expressed gene (DEG) into its matching Entrez Gene ID. The GO enrichment studies were conducted on DEGs using the “clusterProfiler” R package in order to uncover significant biological characteristics. A *P* value (q value) < .05 was deemed to indicate a statistically significant difference. The enrichment analysis findings were visualized using the R packages “enrichplot” and “ggplot2.”

### 2.3. Development and verification of a prognostic risk score model

The TCGA cohort samples were designated as the training set, whereas the E-MTAB-1980 samples were designated as the test set. The expression levels of lncRNAs relevant to FA metabolism, which were differently expressed, were combined with the associated predictive outcomes for each sample using the sample’s ID. The lncRNAs associated with prognosis were identified by doing univariate Cox regression analysis on the differentially expressed lncRNAs relevant to FA metabolism in the training set. The genes that had a *P* value of < .05 were chosen. The “glmnet” R package was utilized to further analyze the prognosis-related lncRNAs using LASSO Cox regression analysis. This study was conducted to create a prognostic risk score model for predicting the overall survival (OS) of ccRCC samples. A 10-fold cross-validation was performed to ascertain the penalty parameter (λ) of the model. The following formula was utilized to compute the risk score for each sample. The *risk score* = $\sum_{i=1}^{n}coef_{i}\times Expr_{i}$

The variable *Expr*_*i*_ represents the expression values of genes *i* in the prognostic risk score model, while *coef*_*i*_ represents the regression coefficient of gene *i* in the signature. The samples were categorized into low- and high-risk score groups using the median value of the risk scores. The OS difference between low-risk and high-risk score groups was compared using Kaplan–Meier analysis with the log-rank test. The predictive accuracy of the prognostic risk score model was evaluated by plotting time-dependent receiver operating characteristic (ROC) curves using the “timeROC” package in R. Furthermore, the test set was used to further assess the reliability and applicability of the prognostic risk score model.

### 2.4. Principal-component analysis (PCA) comparison before and after prognostic risk score model

The limma package in R was effectively utilized to conduct PCA on gene expression profiles before and after applying the prognostic risk score model in the training set. This analysis aimed to discern the significant differences between the low- and high-risk score groups. PCA was initially conducted on the expression patterns of all genes relevant to FA metabolism that showed differential expression. PCA was subsequently employed to examine the expression profiles of genes derived from the predictive risk score model. Ultimately, the ggplot2 tool was employed to visually display the PCA outcomes on two-dimensional plots based on the first 2 main components. PCA was employed to efficiently reduce the dimensions of high-dimensional data, specifically the gene expression profiles of 309 FAM genes, 53 FAM-related lncRNAs, and a risk model categorized based on the expression profiles of 15 FAM-related lncRNAs. PCA was utilized for model identification and grouping display.

### 2.5. Development of a nomogram for predicting OS

A nomogram was created using the “rms” package in R, utilizing age, gender, clinical stage, and prognostic risk score model. The nomogram was produced based on the TCGA cohort to predict overall survival in ccRCC. Calibration curves that vary with time were created in order to forecast the precision of the nomogram. Furthermore, a multivariate Cox regression analysis was employed to confirm whether the prognostic risk score model might function as an autonomous indicator for predicting overall survival in ccRCC. The area under the curve (AUC) was subsequently computed to quantify the prognostic significance of the nomogram using online ROC curves.

### 2.6. Gene set variation analysis (GSVA) and gene set enrichment analysis (GSEA)

The gene profile was subjected to GSVA analysis using the “GSVA” R package to assess the differences in biological processes between the low- and high-risk score groups. GSVA is a non-parametric and unsupervised technique that may assess the changes in pathways or biological processes using an expression matrix sample.^[43]^ In TCGA-ccRCC, gene set enrichment analysis (GSEA) was employed to discover the biological processes that were significantly altered between the high-risk and low-risk subgroups.^[44]^ The reference gene sets utilized were the “c2.cp.kegg.v7.4.symbols” gene sets obtained from the molecular signatures database available at https://www.gsea-msigdb.org/gsea/msigdb. A statistically significant enrichment route was established by FDR < 0.05.

### 2.7. Correlation of risk score with tumor infiltrating immune cells characterization

The immune infiltration information includes the fractions of several immune cell types in each specimen, such as B cells, CD4 + T cells, CD8 + T cells, dendritic cells, macrophages, and neutrophils. This data was obtained from the tumor immune estimation resource (TIMER) at the following link: https://cistrome.shinyapps.io/timer/. A correlation analysis was performed to evaluate the association between the infiltration of immune cells in tumors and the prognostic risk score. The enrichment of the 2 separate hazard categories in 29 immune function-associated gene sets was determined using a single sample gene-set enrichment analysis (ssGSEA) with the R package “GSEAbase.” Afterwards, the R package “ESTIMATE” was used to evaluate the purity of the tumor and the presence of infiltrating cells, namely stromal and immune cells. This analysis aimed to confirm significant differences in the immunological microenvironment of the tumor between 2 high-risk groupings. The proportion of 22 immune cell types in each tumor specimen was determined by identifying cell types based on the estimation of relative subsets of RNA transcripts using CIBERSORT (https://cibersort.stanford.edu/).

### 2.8. Construction of CeRNA and TF-lncRNA regulatory networks

The ENCORI database was utilized to forecast the relationships between lncRNAs linked to FA metabolism and potential differentially expressed microRNAs (DEmiRNAs). The DEmiRNAs relevant to FA metabolism and targeted by DEmiRNAs were obtained using the TargetScan, miRDB, and ENCORI databases. Only the common parts of the 3 databases were considered as potential candidate DEmiRNAs. The false discovery rate (FDR) is less than 0.05 and the absolute value of the logarithm of the fold change (FC) is > 1. In addition, the list of 1639 transcription factors (TFs, Table S2, Supplemental Digital Content, http://links.lww.com/MD/L732) was referred from a previous research project,^[45]^ and the RNA-seq profiles of TFs were downloaded from the TCGA database. The Spearman correlation analysis was performed to evaluate the association between the TFs and the FAM-related lncRNAs (Table S3, Supplemental Digital Content, http://links.lww.com/MD/L733). *P* < .001 and correlation coefficient > 0.6 was the cutoff values. Finally, Cytoscape (version 3.8.0) was employed to build an underlying ceRNA and TF-lncRNA regulatory network.

### 2.9. Statistical analysis

The Wilcoxon test was utilized to compare 2 groups, whereas the Kruskal–Wallis test was conducted to compare more than 2 groups. Overall survival (OS) is the period of time between the date of diagnosis and the date of death. Survival curves were generated using the Kaplan–Meier log rank test. Pearson correlation tests were used to examine the associations between risk scores, clinical factors, immune cell infiltration extent, and immunological checkpoints. Results from the CIBERSORT method with a *P* value *P* ≥ .05 were excluded from further analysis. The independent prognostic prediction performance of the risk signature was validated by Cox regression models, which included both univariate and multivariate analyses. The ROC curves were generated to evaluate the prediction efficacy of the prognostic risk score model and nomogram. The statistical analyses were conducted using R software (version 4.1.1).

## 3. Results

### 3.1. Identification of FAM-related lncRNAs in patients with ccRCC

**Figure 1.**
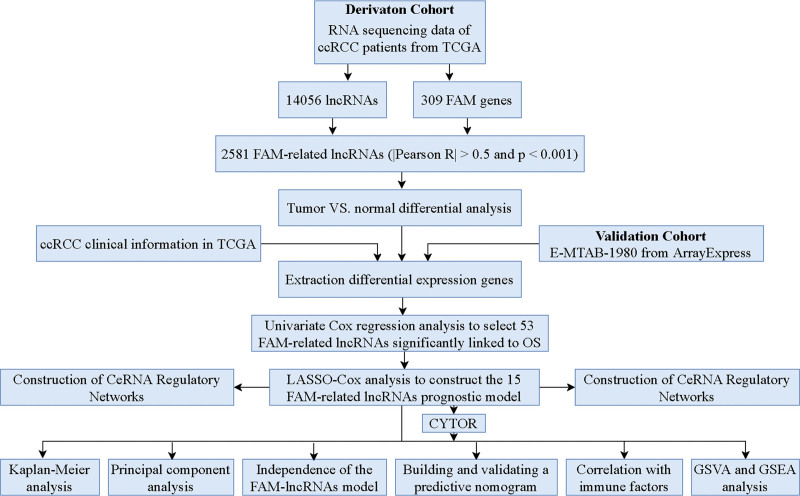
Flow chart of this study.

**Figure 2.**
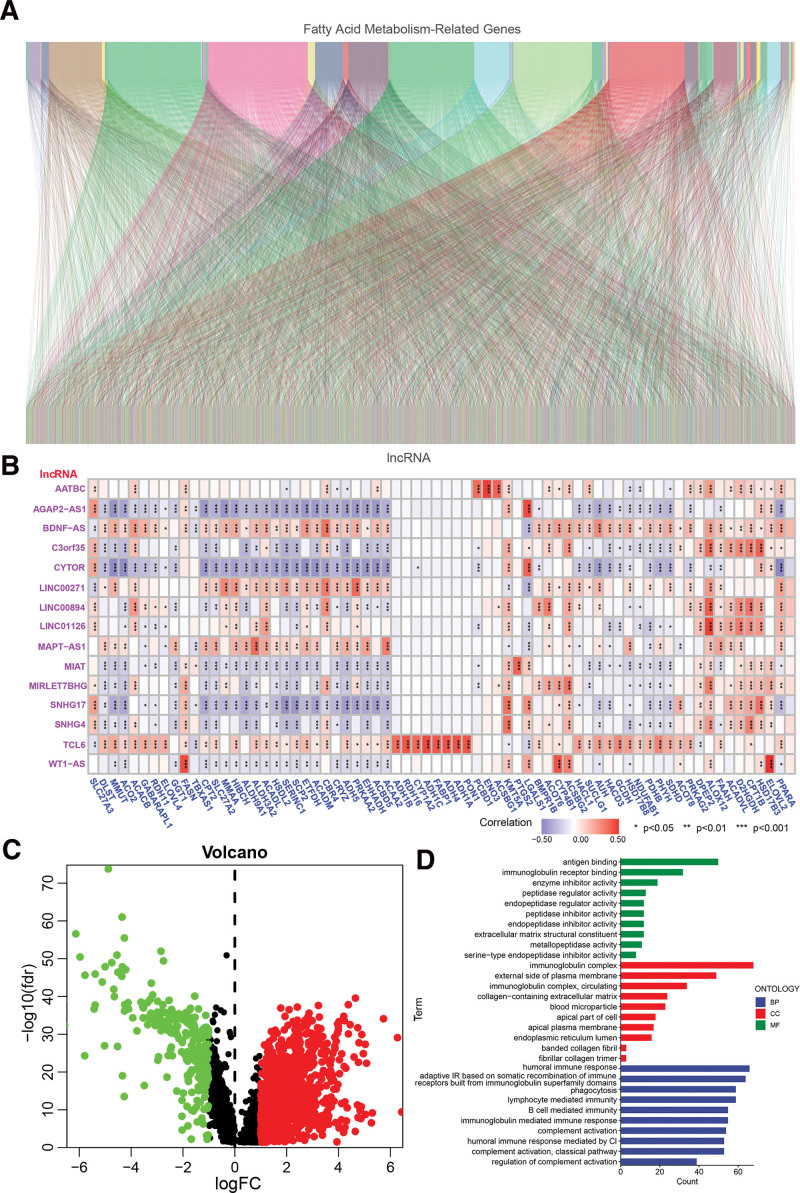
Identification of FA metabolism-related lncRNAs in the TCGA-ccRCC cohort. A. Sankey relational diagram for FA metabolism-related genes and FA metabolism-related lncRNAs. B. Heatmap for the correlations between FA metabolism-related genes and the 15 prognostic FA metabolism-related lncRNAs. C. The volcano map of 1614 differentially expressed FA metabolism-related lncRNAs. D. The result of GO enrichment analysis of differentially expressed FA metabolism-related lncRNAs.

Figure 1 illustrates the comprehensive process for constructing a risk model and conducting subsequent studies. The clinical attributes of ccRCC patients were obtained from the TCGA database. We acquired a total of 537 instances of TCGA-ccRCC, and the clinical characteristics are presented in Table 1. The matrix representation of 309 FAM genes and 14056 lncRNAs was extracted from the TCGA database. FAM-related lncRNAs were identified as lncRNAs that exhibited a substantial correlation (|Pearson R| > 0.5 and *P* < .001) with at least one of the 309 FAM genes. The FAM-lncRNA coexpression network was shown using a Sankey diagram in Figure 2A. A total of 2581 lncRNAs associated with FAM were identified in the network (Table S4, Supplemental Digital Content, http://links.lww.com/MD/L734). Figure 2B displays the connection between FAM genes and FAM-related lncRNAs in the complete TCGA dataset. A comparison was made between the expression levels of FAM-related lncRNAs in normal and cancer tissue samples. From the TCGA cohort, a total of 1614 genes with a false discovery rate (FDR) < 0.05 were selected. The cancer tissue samples exhibited upregulation of 1344 genes and downregulation of 270 genes (Table S5, Supplemental Digital Content, http://links.lww.com/MD/L735). Figure 2C displays the genes that are expressed differently, known as DEGs. The biological processes that were significantly enriched in the study included the humoral immune response, which is an adaptive immune response that involves the recombination of immune receptors made up of immunoglobulin superfamily domains. Other enriched processes included phagocytosis, lymphocyte mediated immunity, and B cell mediated immunity (Fig. 2D). The results demonstrate that FAM-related lncRNAs have a substantial impact on the development of ccRCC.

**Table 1:** Clinical characteristics of 530 patients with ccRCC in this study.

Characteristics	Variable	Low risk dataset	High risk dataset
Vital status	Alive	218	139
	Dead	47	126
Age	≤60	130	134
	>60	135	131
Gender	Female	94	92
	Male	171	173
Tumor grade	G1	9	5
	G2	139	88
	G3	95	111
	G4	18	57
	Unknown	4	4
Tumor stage	Stage I	165	100
	Stage II	26	31
	Stage III	50	73
	Stage IV	23	59
	Unknown	1	2
T staging	T1	168	103
	T2	30	39
	T3	66	113
	T4	1	10
M staging	M0	236	184
	M1	22	56
	Unknown	7	25
N staging	N0	123	116
	N1	4	12
	Unknown	138	137

### 3.2. Construction of a prognostic model according to FAM-related lncRNAs in ccRCC patients

**Figure 3.**
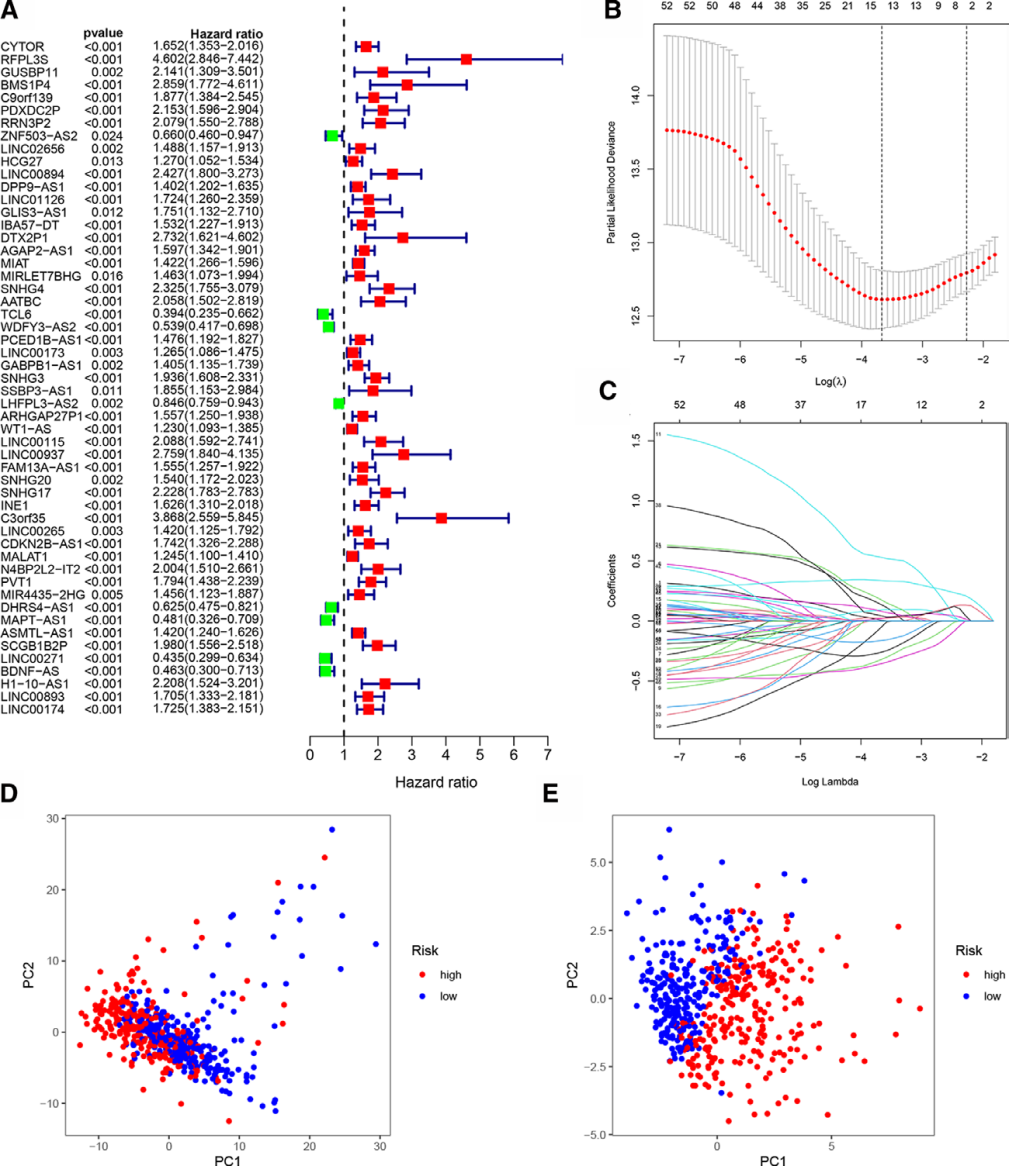
Development of prognostic risk score model. A. The forest plot of 53 FA metabolism-related lncRNAs related with prognosis. B. The tuning parameters (log λ) of OS-related lncRNAs were selected to cross-verify the error curve. According to the minimal criterion and 1-se criterion, perpendicular imaginary lines were drawn at the optimal value. C. The LASSO coefficient profile of 15 OS-related lncRNAs and perpendicular imaginary line were drawn at the value chosen by 10-fold cross-validation. D. Principal component analysis based on all FA metabolism-related lncRNAs in ccRCC. E. Principal component analysis based on FA metabolism-related lncRNAs risk score to distinguish tumors from the normal samples in the TCGA cohort. The group marked with red represented high-risk patients, and the group marked with blue represented low-risk patients.

We conducted univariate Cox regression analysis to identify predictive lncRNAs associated with FAM in a training set of 2581 FAM-related lncRNAs from the TCGA dataset. There were a total of fifty-three lncRNAs associated to the FAM gene family that showed a significant correlation with OS in the TCGA dataset (Fig. 3A). Utilizing LASSO-Cox analysis not only improves the precision and interpretability of the statistical model, but also allows for simultaneous consideration of variable alternatives and regularization. This method is often used to choose ideal features in high-dimensional data that have a weak correlation but a strong predictive value, in order to prevent overfitting. Therefore, this approach may accurately identify the most accessible forecast indicators and generate a prognostic measure to anticipate clinical outcomes. The dashed perpendicular line represents the logarithm of λ’s first-rank value, which minimizes the bias in segment likelihood. In order to develop a prognostic risk score model that can accurately measure the risk level of each patient, we selected 15 lncRNAs associated with FAM. These lncRNAs are CYTOR, LINC00894, LINC01126, AGAP2-AS1, MIAT, MIRLET7BHG, SNHG4, AATBC, TCL6, WT1-AS, SNHG17, C3orf35, MAPT-AS1, LINC00271, and BDNF-AS. We used the LASSO-Cox regression model with a minimum value of λ to identify and retain these lncRNAs (Fig. 3B and C; Table 2). Later on, the risk score model was employed to fully differentiate ccRCC samples (low or high risk) (Fig. 3D and E).

**Table 2:** FAM-related lncRNAs with significant prognostic value in ccRCC identified by Cox regression analysis.

lncRNA	HR	HR.95L	HR.95H	pvalue
CYTOR	1.651534507	1.352789598	2.01625311	8.31E-07
LINC00894	2.427060556	1.799923679	3.27270707	6.12E-09
LINC01126	1.724458823	1.260428813	2.35932264	0.000656
AGAP2-AS1	1.597262398	1.341975619	1.9011129	1.36E-07
MIAT	1.421872255	1.266444188	1.59637569	2.53E-09
MIRLET7BHG	1.462540972	1.072720021	1.99402086	0.016225
SNHG4	2.325059473	1.755496575	3.07941447	3.98E-09
AATBC	2.057701641	1.50193178	2.81912674	7.05E-06
TCL6	0.394090692	0.234622516	0.66194616	0.000433
WT1-AS	1.230198257	1.092814851	1.38485284	0.000606
SNHG17	2.227927888	1.783486345	2.78312345	1.71E-12
C3orf35	3.867887237	2.559383953	5.84537215	1.36E-10
MAPT-AS1	0.4806276	0.326009886	0.70857633	0.000216
LINC00271	0.435344763	0.298885413	0.6341061	1.46E-05
BDNF-AS	0.462530812	0.299877243	0.71340776	0.000488

### 3.3. Validation of the FAM-LPM in the TCGA and E-MTAB-1980 dataset

**Figure 4.**
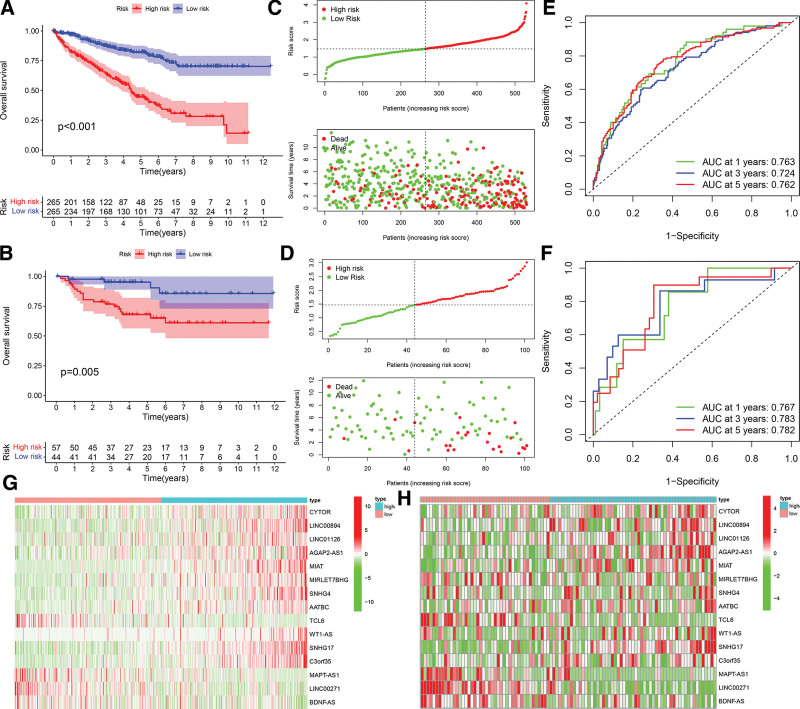
Prognostic value of the risk model of the 15 FA metabolism-related lncRNAs of ccRCC patients. A. The comparison of overall survival (OS) between low- and high-risk score groups in the TCGA cohort. B. The comparison of OS between low- and high-risk score groups in the E-MTAB-1980 cohort. C. Distribution of risk score, survival status, and survival time of FAM-related lncRNA prognostic model in the TCGA cohort. D. Distribution of risk score, survival status, and survival time of FAM-related lncRNA prognostic model in the E-MTAB-1980 cohort. E. AUC of time-dependent ROC curves verified the prognostic performance of the risk score in the TCGA cohort. F. AUC of time-dependent ROC curves verified the prognostic performance of the risk score in the E-MTAB-1980 cohort. G. Distribution of heatmap of the fifteen prognostic FAM-related lncRNAs for each patient in the TCGA cohort. H. Distribution of heatmap of the fifteen prognostic FAM-related lncRNAs for each patient in the E-MTAB-1980 cohort.

In order to evaluate the predictive ability of this well-established model, we computed risk scores for each patient in the TCGA and E-MTAB-1980 datasets using a standardized formula. ccRCC patients in the TCGA and E-MTAB-1980 databases were categorized into low- and high-risk categories using the median risk score. The findings exhibited uniformity in both the TCGA and ICGC datasets: ccRCC patients with elevated risk scores exhibited decreased OS rates and a reduced OS duration (Fig. 4A and B). The distribution of risk scores and survival status is depicted in Fig. 4C and D. The results indicate that patients with higher risk scores experienced shorter OS and had a greater mortality rate. The heatmap results illustrate the distinct expressions of twenty genes in the low- and high-risk score groups. The ROC analysis shown that FAM-LPM had a robust predictive significance for ccRCC patients in the TCGA dataset (1-year AUC = 0.763, 3-year AUC = 0.724, 5-year AUC = 0.762; Fig. 4E) and E-MTAB-1980 dataset (1-year AUC = 0.767, 3-year AUC = 0.783, 5-year AUC = 0.782; Fig. 4F). The results demonstrated that the FAM-LPM exhibited a strong and consistent capacity to predict OS. The heatmap reveals that the genes CYTOR, LINC00894, LINC01126, AGAP2-AS1, MIAT, MIRLET7BHG, SNHG4, AATBC, WT1-AS, SNHG17, and C3orf35 had elevated expressions in the high-risk score group. Conversely, the low-risk score group displayed increased levels of TCL6, MAPT-AS1, LINC00271, and BDNF-AS (Fig. 4G and H).

### 3.4. Principal-component analysis (PCA) further verifies the grouping ability of the FAM-LPM

**Figure 5.**
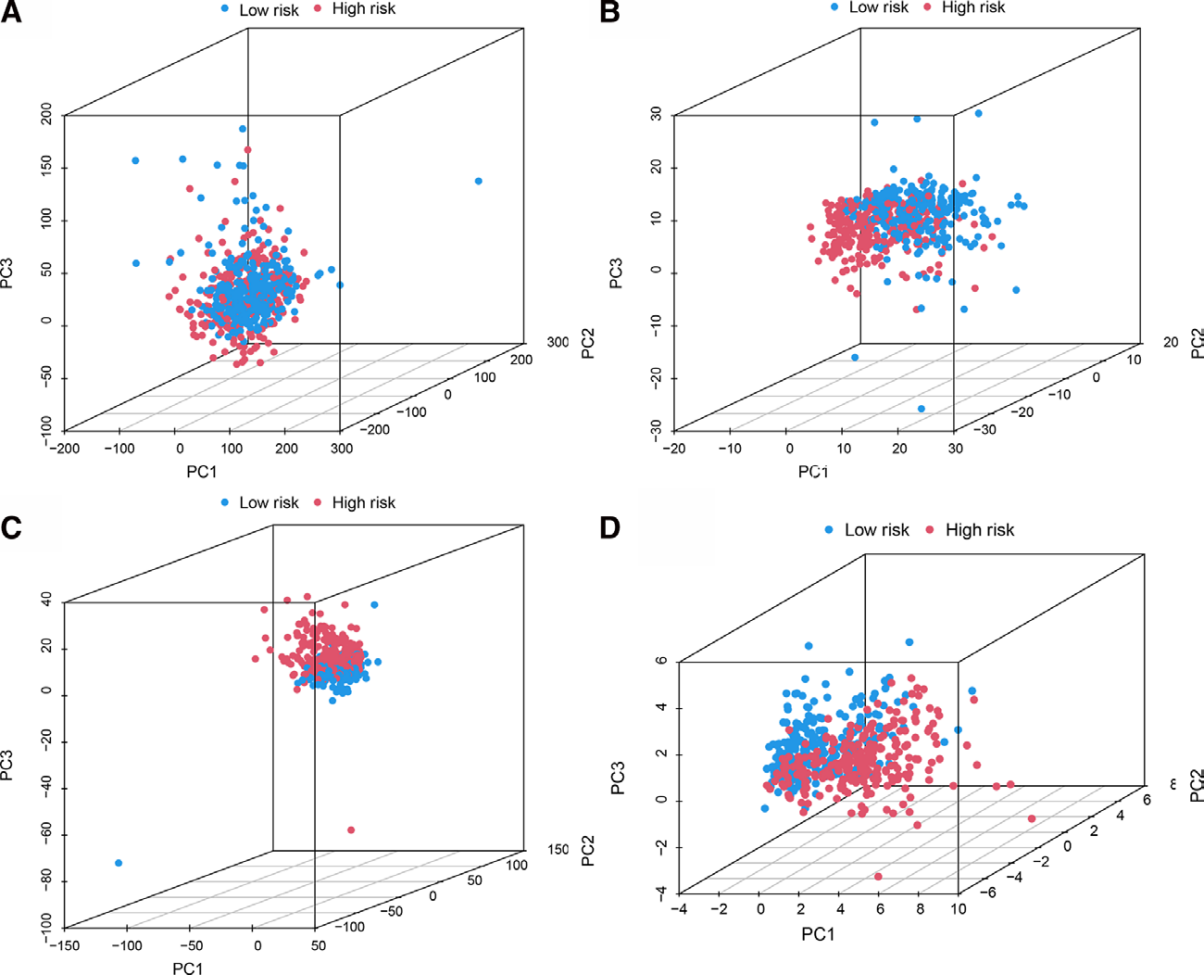
Principal component analysis between the high- and low-risk groups based on incomplete sentence. A. Entire gene expression profiles, B. 309 FAM genes, C. 53 FAM-related lncRNAs, and D. Risk model based on the representation profiles of the 15 FAM-related lncRNAs in the TCGA cohort.

PCA was performed to assess the distinction between the low-risk and high-risk groups using the complete gene expression profiles, which included 309 FAM genes and 53 FAM-related lncRNAs. The risk model was constructed based on the expression profiles of 15 FAM-related lncRNAs (Fig. 5A–D). The graphs in Figure 5A–C demonstrate that the high- and low-risk groups were distributed in a somewhat dispersed manner. Nevertheless, the outcomes derived from our model demonstrated that the low- and high-risk groups exhibited distinct distributions (Fig. 5D). These findings indicate that the prognostic signature is capable of differentiating between the low- and high-risk groups.

### 3.5. Construction of a nomogram for predicting survival

**Figure 6.**
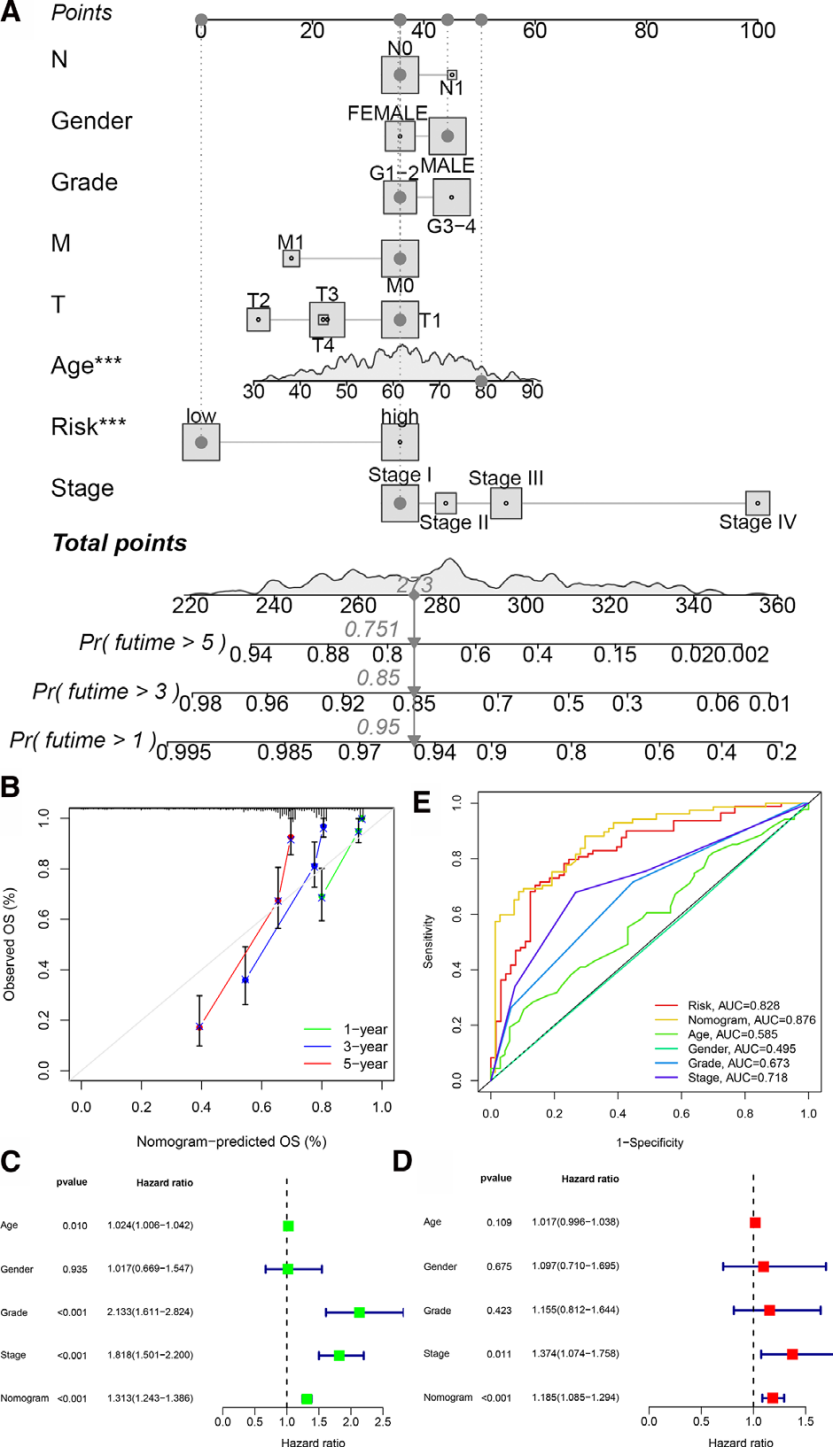
Construction and evaluation of a prognostic nomogram. A. Nomogram predicting OS of patients from TCGA cohort. B. The calibration plots of the nomogram. The x axis is nomogram-predicted survival, and the y axis is actual survival. C. Univariate Cox regression analysis of the nomogram. D. Multivariate Cox regression analysis of the nomogram. E. ROC curves for risk score and clinical pathological characteristics.

A nomogram was developed to predict OS in ccRCC samples. The nomogram incorporates age, gender, International Society of Urologic Pathologists (ISUP) grade, pathological stage, and predictive risk score model. The construction of the nomogram is illustrated in Figure 6A. The calibration curves at the 1-year, 3-year, and 5-year marks demonstrated that the nomogram was able to reliably forecast the OS of ccRCC patients, as depicted in Figures 6B. The results of the univariate Cox regression analysis demonstrate that the prognostic risk score model, age, ISUP grade, and clinical-pathological stage are independent markers of prognosis (Fig. 6C). The findings of the multivariate Cox regression analysis demonstrated that both the prognostic risk score model and the clinical-pathological stage are independent prognostic indicators (Fig. 6D). The 5-year area under the ROC curves (AUC) revealed that the nomogram (AUC = 0.876) exhibited superior predictive value compared to individual indicators, such as age (AUC = 0.585), ISUP grade (AUC = 0.673), pathological stage (AUC = 0.718), and prognostic risk score model (AUC = 0.828; Figure 6E).

### 3.6. Gene set variation analysis (GSVA) and gene set enrichment analysis (GSEA)

**Figure 7.**
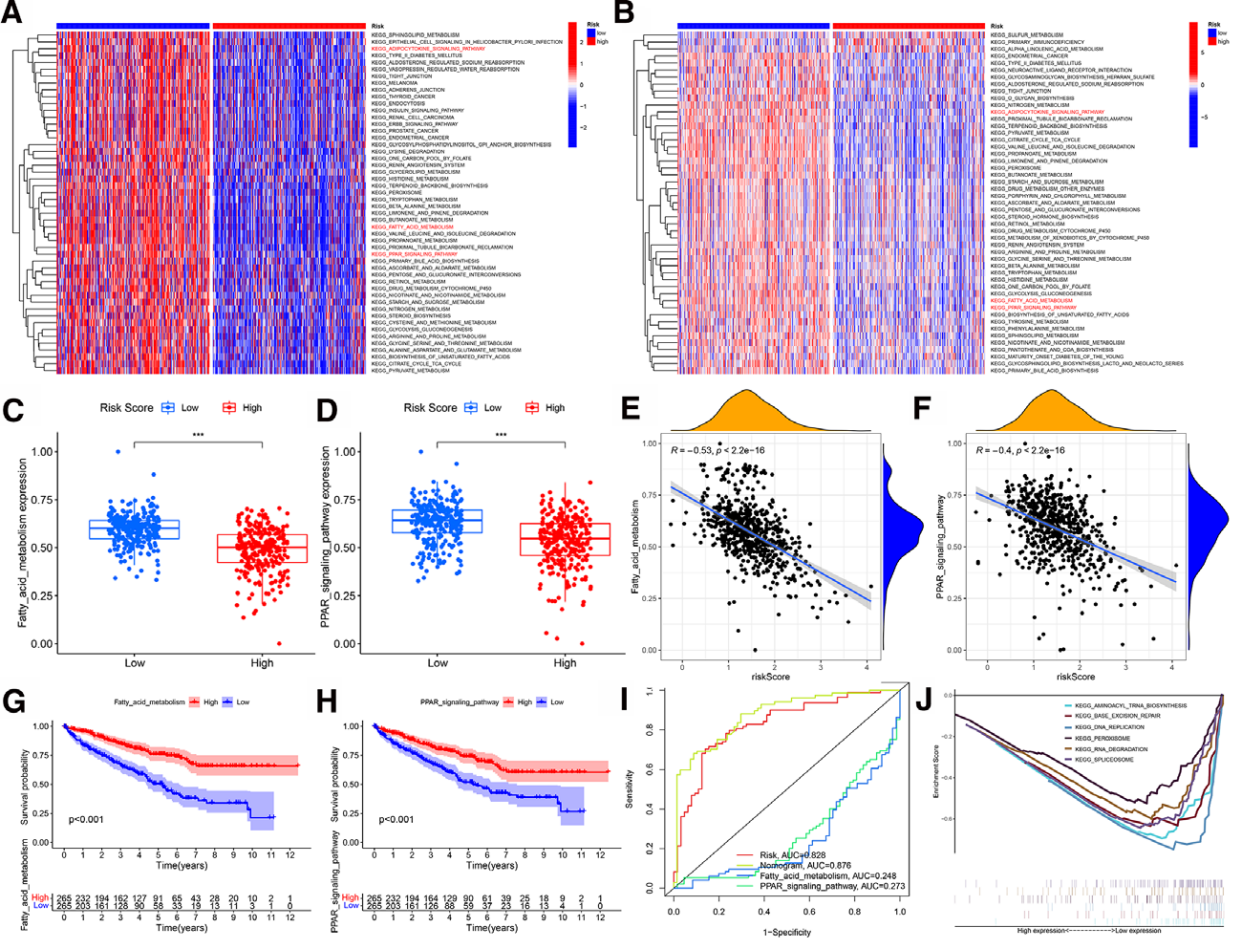
Gene set variation analysis and gene set enrichment analysis. A. Heatmap representing differences in stroma-activated pathways between low- and high-risk score groups. B. Heatmap of the ssGSEA score calculated by pathways gene sets between low- and high-risk score groups. C. The FA metabolism score difference between high risk score and low risk score groups. D. The PPAR signaling pathway score difference between high risk score and low risk score groups. E. The relationship between risk score model and FA metabolism score. F. The relationship between risk score model and PPAR signaling pathway score. G. Survival analysis for subgroup patients stratified by FA metabolism score. H. Survival analysis for subgroup patients stratified by PPAR signaling pathway score. I. The predictive value of FA metabolism and PPAR signaling pathway scores in ccRCC patients. J. Gene set enrichment analysis map of low- and high-risk score groups.

GSVA enrichment analysis was performed using the gene sets of “c2.cp.kegg.v7.4.symbols” obtained from the Molecular Signatures Database (MSigDB) to investigate the biological behaviors in the 2 groups. Curiously, the low-risk score showed an enrichment of various metabolic pathways, such as FA metabolism, PPAR signaling system, and adipocytokin signaling circuit (Fig. 7A). We conducted an analysis on the gene sets labeled as “c2.cp.kegg.v7.4.symbols” from the MSigDB using the GSEA method. The computed outcomes of single-sample gene set enrichment analysis (ssGSEA) were essentially in agreement with GSVA (Fig. 7B). Consequently, we conducted a more in-depth analysis of the predictive importance of the FA metabolism and PPAR signaling pathway score. The findings indicate that the low-risk group exhibited a greater PPAR signaling pathway score (Fig. 7C and D); The scores of FA metabolism and PPAR signaling route were shown to have a negative correlation with risk scores, as shown in Figure 7E and F. Additionally, a higher score in FA metabolism and PPAR signaling pathway was associated with increased overall survival, as depicted in Figure 7G and H. The time-dependent receiver operating characteristic (ROC) analysis at the 5-year mark showed that the predictive accuracy of FA metabolism and PPAR signaling pathway scores (AUC = 0.248, AUC = 0.273) was lower compared to the risk score model (AUC = 0.828) (Fig. 7I). Analysis using GSEA on TCGA data showed that genes related to spliceosome, aminoacyl tRNA biosynthesis, RNA degradation, DNA replication, base excision repair, and peroxisome were found to be considerably more abundant in samples with low risk (Fig. 7J). The results suggest that the high-risk group’s enrichment pathways are linked to metabolic biological processes.

### 3.7. Immune-related characteristic in the low- and high-risk score groups

**Figure 8.**
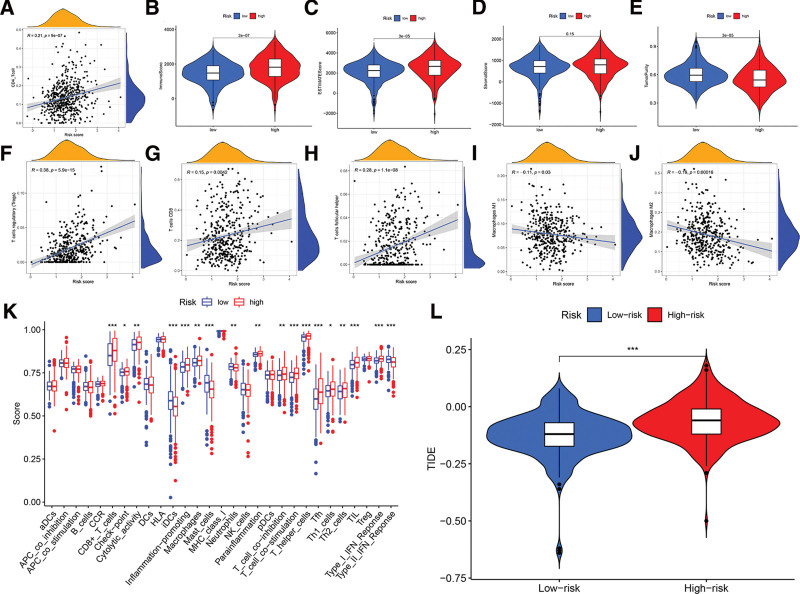
Immune-related characteristic in the low- and high-risk score groups. A. Relationship between CD4 + T cell and risk score. B. Comparison of ImmuneScore between low- and high-risk groups. C. Comparison of ESTIMATEScore between low- and high-risk groups. D. Comparison of StromalScore between low- and high-risk groups. E. Comparison of tumor purity between low- and high-risk groups. F. Relationship between T cells regulatory (Tregs) and risk score. G. Relationship between T cells follicular helper and risk score. H. Relationship between macrophages M1 and risk score. I. Relationship between macrophages M2 and risk score. J. Relationship between CD8 + T cells and risk score. K. Heatmap displayed enrichment of 29 immune signatures of low- and high-risk groups. Blue represents low activity, and red represent high activity. L. TIDE prediction difference in high- and low-risk patients.

In order to investigate the potential of risk score as immune indicators, we conducted correlation analyses between prognostic risk score and various immune factors. These factors include tumor-infiltrating immune cells (TIICs) obtained from TIMER, immune score calculated using the ESTIMATE algorithm, ssGSEA signatures, as well as TIICs subtype and level determined through the CIBERSORT method. The TIMER results revealed a significant positive correlation between the as-constructed signature and CD4 + T cells (*r* = 0.21; *P* = 9e − 07) (Fig. 8A), suggesting that high-risk samples had a higher presence of killer immune cells. Similarly, patients at a higher risk level exhibited elevated immune score and estimate score, indicating a greater presence of immune infiltration (Fig. 8B and C). There was no notable disparity in terms of stromal score (Fig. 8D). Nevertheless, low-risk patients had more tumor purity, indicating a reduced presence of immune infiltration (Fig. 8E). The CIBERSORT algorithm findings revealed a significant abundance of immune-suppressive cell infiltration, specifically T regulatory cells (Tregs) and T cell follicular helper cells, in the group. This observation aligns with the survival disadvantage observed in the high-risk group (Fig. 8F and G). The high-risk group had an enrichment of M1 macrophages, M2 macrophages (which activate natural killer cells), and CD8 + T cells (Fig. 8H–J). Furthermore, the high-risk group exhibited activation of type I interferon (IFN) response, inflammation-promoting function, and checkpoint, suggesting that patients in this group were susceptible to immunological escape (Fig. 8K). The interaction between immune cells in the tumor microenvironment and cancer cells has a role in the development of immune evasion mechanisms and resistance to drugs. Not unexpectedly, our findings revealed that the low-risk group exhibited a higher likelihood of responding to immunotherapy compared to the high-risk group. This suggests that the risk score-based classifier index could potentially be used as an indicator for predicting Tumor Immune Dysfunction and Exclusion (TIDE) (Fig. 8L).

### 3.8. CYTOR is an independently prognostic factor

**Figure 9.**
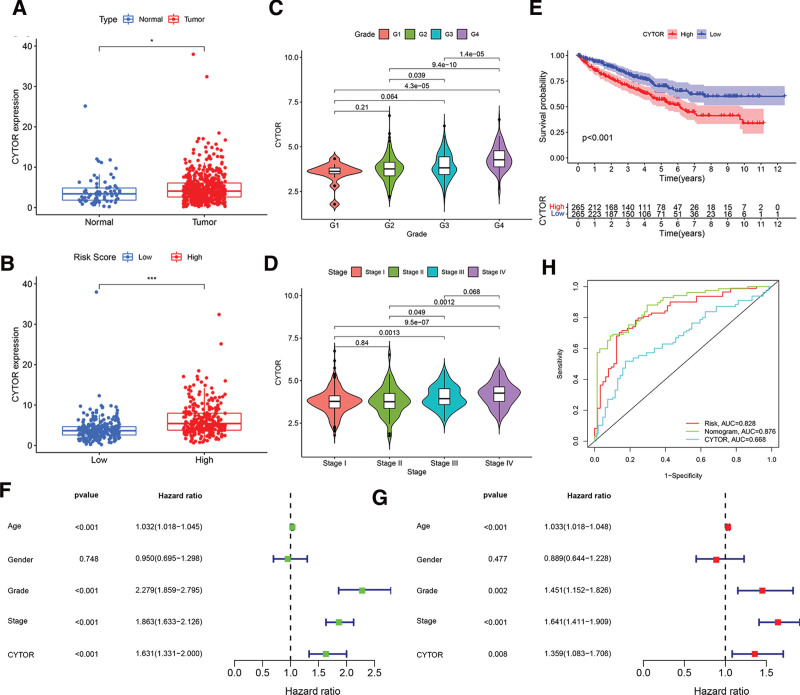
The clinical significance of CYTOR in ccRCC. A. CYTOR are overexpressed in ccRCC tumor tissue. B. CYTOR are overexpressed in ccRCC high risk score group. C. Correlation of risk score with tumor grade. D. Correlation of risk score with tumor stage. E. The comparison of overall survival (OS) between low- and high-expression CYTOR level in the TCGA cohort. F. Univariate OS analysis of ccRCC patients by the COX proportional hazards model. G. Multivariate OS analysis of ccRCC patients by the COX proportional hazards model. H. The predictive value of CYTOR expression level in ccRCC patients.

Among the prognostic FAM-related lncRNAs, only the gene CYTOR showed an increase in expression levels. Consequently, its involvement in ccRCC was further examined. The expression level of CYTOR was examined between normal tissues and tumor samples using TCGA data. CYTOR expression level in tumor specimens was greater compared to surrounding normal tissues (Fig. 9A). CYTOR exhibited significant expression levels in the high-risk group, as depicted in Figure 9B. Furthermore, there is a positive correlation between the advanced tumor stage and grade, and an elevated risk score, as depicted in Figure 9C and D. In order to evaluate the predictive significance of CYTOR in ccRCC, a Kaplan–Meier analysis was conducted to compare patients with low and high levels of CYTOR expression. Figure 9E demonstrates that decreased CYTOR expression levels are associated with extended OS (*P* < .001). The results of the Univariate Cox and Multivariate Cox analysis indicated that age, grade, stage, and CYTOR were all independently associated with OS in patients with ccRCC. Specifically, CYTOR showed a hazard ratio (HR) of 1.631 (95% confidence interval [CI] = 1.331−2.000) and grade showed an HR of 1.359 (95% CI = 1.083−1.706). These findings suggest that CYTOR may serve as an independent prognostic indicator for ccRCC, as shown in Figure 9F and G. Ultimately, we conducted a time-dependent ROC curve analysis. While the time-dependent ROC at 5 years indicated that the CYTOR score could predict the patients’ survival rate, its prognostic value (AUC = 0.668) was lower than that of the risk score model (AUC = 0.828) (Fig. 9H).

### 3.9. Role of CYTOR in the context of TIME

**Figure 10.**
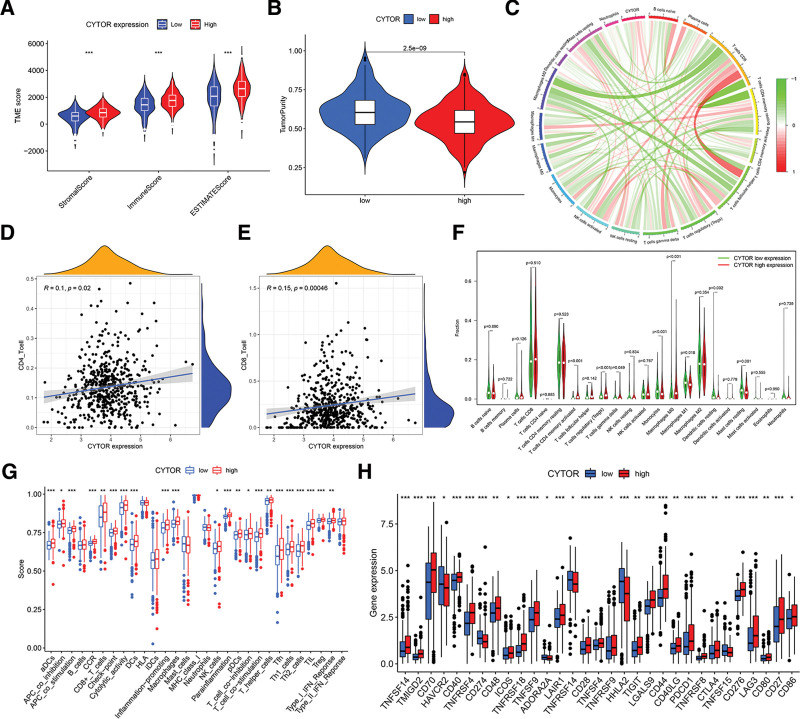
The role of CYTOR in TIME features. A. Comparison of stromal score, immune score, and ESTIMATE score between low- and high-CYTOR groups. B. Comparison of tumor purity between low- and high-CYTOR groups. C. Correlation of CYTOR expression with immune infiltration level. D. Relationship between CD4 + T cells and CYTOR expression. E. Relationship between CD8 + T cells and CYTOR expression. F. Comparison of CIBERSORT results between low- and high-CYTOR groups. G. Comparison of ssGSEA enrichment between low- and high-CYTOR groups. H. Comparison of immune checkpoint blockade-related genes expression levels between low-CYTOR group and high-CYTOR group.

In order to provide a clearer understanding of the connection between CYTOR and TIME traits in ccRCC, a thorough analysis was conducted following the previously outlined methodology. Patients with ccRCC were categorized into high- and low-CYTOR subtypes, using the median CYTOR expression level as the criterion. The ESTIMATE results showed that patients with elevated CYTOR expression exhibited a notably increased stromal score and immunological score compared to patients in the low CYTOR group. This suggests that low-risk samples have a lower abundance of stromal cells and immune cells (Fig. 10A and B). Figure 10C demonstrates the link between CYTOR expression and immune cells. Following that, the TIMER results indicated a positive correlation between the expression level of CYTOR and the infiltration of CD4 + and CD8 + T cells (Fig. 10D and E). Furthermore, the CIBERSORT analysis revealed a notable increase in the percentage of activated memory CD4 + T cells among patients classified as high-risk (Fig. 10F). The results of ssGSEA analysis revealed a significant decrease in the infiltration fraction of CD8 + T cells, checkpoint, NK cells, T helper cells, Tfh, Treg and TIL, APC co-inhibition, APC co-stimulation, T cell co-inhibition, T cell co-stimulation, CCR, cytolytic activity, inflammation-promoting, and IFN-response type-I expression when the risk score decreased (Fig. 10G). In addition, the expression levels of 31 out of 47 genes linked with immunological check blockage (such as CD274 and CTLA4) were significantly dysregulated between the low-CYTOR and high-CYTOR groups in distinct subgroups (Fig. 10H). This suggests that the higher presence of immune cells in the group with high expression indicates that inflammation connected to CYTOR may decrease the survival rate of ccRCC patients.

### 3.10. Development of the CeRNA and TF-lncRNA regulatory network

**Figure 11.**
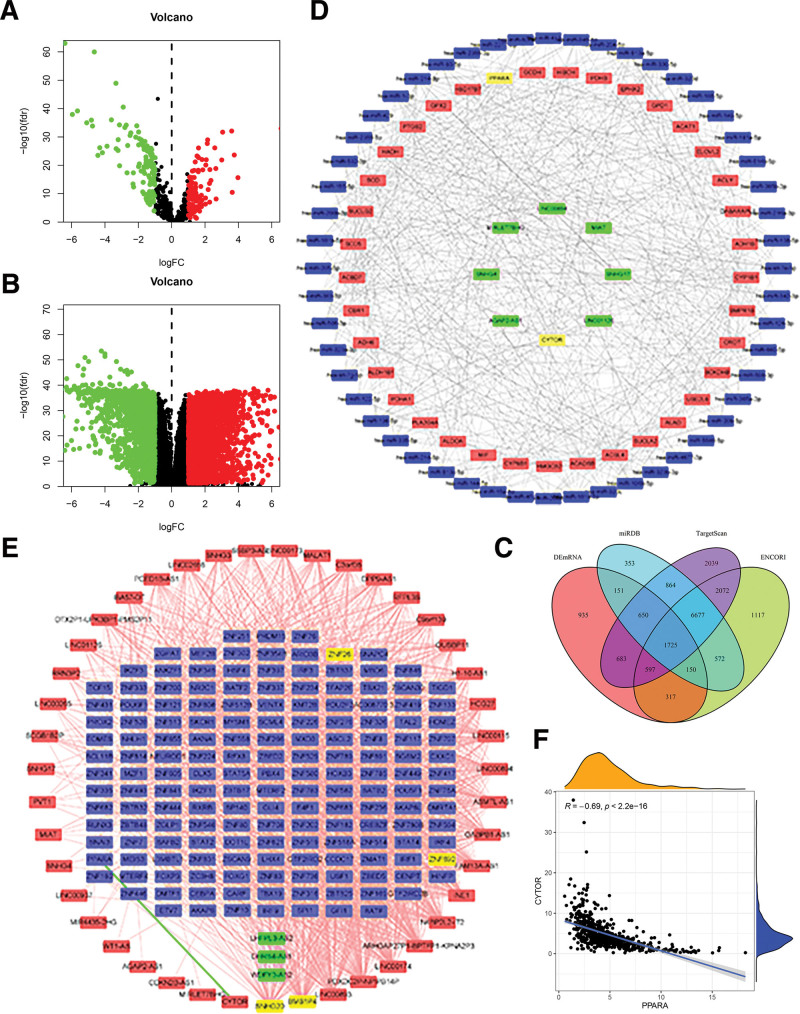
CeRNA and TF-lncRNA regulatory network. A. The volcano map of differentially expressed mRNAs. B. The volcano map of differentially expressed miRNAs. C. The intersection of predicted miRNAs and DEmiRNAs. D. FA metabolism-related ceRNA network. The red, green, and blue rectangles represented mRNA, lncRNAs, and miRNA, respectively. E. The regulatory network between TFs and prognostic FAM-related lncRNAs. The red, green, and blue rectangles represented up-regulated lncRNAs, down-regulated lncRNAs, and down-regulated TFs, respectively. The positive and negative correlations (*r* > 0.6 or *r* < −0.6) between TFs and prognostic FAM-related lncRNAs were indicated with red and green lines. F. Correlation between CYTOR and PPARA expression.

We utilized the ENCORI databases to map the 15 lncRNAs associated with FA metabolism. This mapping allowed us to predict the miRNAs that these lncRNAs target. After comparing these predicted miRNAs with a set of 219 DEmiRNAs, we identified 52 miRNAs that were common to both sets (as shown in Fig. 11A). We then selected the mRNAs that interacted with these 52 candidate DEmiRNAs in all 3 datasets. By further comparing these mRNAs with a set of DEmRNAs (as shown in Fig. 11B and C), we identified 37 DEmRNAs that are predicted to be involved in FA metabolism. Using Cytoscape software, we created a ceRNA network consisting of 8 lncRNAs linked to FA metabolism (shown in green), 52 DEmiRNAs (shown in blue), and 37 DEmRNAs related to FA metabolism (shown in red) (Fig. 11D). A correlation network was created to clarify the method by which TFs regulate lncRNAs associated to FAM prognosis. A total of 1473 possible interactions between transcription factors (TFs) and lncRNAs were found. Among these, 43 lncRNAs were elevated and marked in red, while 3 lncRNAs were down-regulated and marked in green. Additionally, there were 167 TFs involved, marked in blue (Fig. 11E). Within our regulatory network, the most important nodes were identified as the hub transcription factors (TFs) or lncRNAs (Table S6, Supplemental Digital Content, http://links.lww.com/MD/L736). These included 2 upregulated lncRNAs (SNHG20 and BMS1P4) and 2 TFs (ZNF26 and ZNF692). Furthermore, we discovered a negative correlation between CYTOR and PPARA (green). As a result, we conducted additional correlation analysis, as shown in Figure 11F. Therefore, these transcription factors have shown great promise in their ability to act as key regulators in the disruption of lncRNAs in ccRCC, thereby facilitating the genesis and advancement of tumors.

## 4. Discussion

ccRCC, characterized by its molecular complexity and heterogeneities, often leads to a significant risk of recurrence and metastasis in patients, along with a poor prognosis.^[46]^ There is an urgent need to discover a new biomarker and elucidate the underlying process for the progression and spread of ccRCC. Despite extensive investigation, the specific molecular fingerprints and regulatory mechanisms of many lncRNAs in ccRCC remain largely unknown.^[47,48]^ After conducting extensive investigations on metabolic reprogramming, researchers have come to recognize the significance of FA metabolism in ccRCC.^[10,11,13,19]^ Lin et al^[17]^ developed twenty mRNA signatures associated to fatty acid metabolism in the TCGA dataset. These signatures showed a high prediction ability, as indicated by time-dependent ROC and KM survival analysis. The high-risk group exhibited a significant decrease in response to anti-PD-1/PD-L1 therapy compared to the low-risk group. The research conducted by Dr Luo’s team was the first to present extensive evidence of the substantial correlation between the prognosis prediction of ccRCC and the tumor environment, as characterized by necroptosis and pyroptosis.^[49]^ The Necroptosis-Pyroptosis Genes (NPG) score has been found as a potential prognostic biomarker for ccRCC. The NPG score-based risk model accurately and consistently predicted the OS and tumor microenvironment of ccRCC. Bao et al^[50]^ devised a unique nomogram and miRNA profile linked to necroptosis to accurately forecast the prognosis of patients with ccRCC. In addition, the study found that 7 specific genes (ARHGAP42, BTG2, DUSP1, PCDHA12, TAL1, TGFBR3, and SIX4) and 6 microRNAs (hsa-miR-101-3p, hsa-miR-193a-3p, hsa-miR-200a-5p, hsa-miR-214-3p, hsa-miR-221-3p, and hsa-miR-223-3p) showed abnormal activity in ccRCC. Nevertheless, our comprehension of the correlation between lncRNA and FA metabolism in ccRCC remains restricted. We have established a ceRNA network that is specifically linked to the metabolic process of FA. Within this network, we have identified a total of fifteen lncRNAs that are also closely involved with FA metabolism. The lncRNAs were utilized to create a prognostic indicator for ccRCC. In order to enhance the accuracy of predictions, we integrated the prognostic signature with clinical characteristics to create a nomogram that demonstrates exceptional consistency and dependability. Furthermore, we have showcased the significant role of lncRNA CYTOR, which is part of the signature associated with FA metabolism in ccRCC. The present study proposes that a predictive signature, which relies on lncRNAs associated with FA metabolism, might be employed to categorize patients with ccRCC depending on their prognosis. This predictive characteristic will help to clarify the molecular process of ccRCC and offer novel insights for targeting FA metabolism in treatments.

We initially computed the GSVA enrichment score for both the FA metabolism and PPAR signaling pathway in each patient with ccRCC. Consequently, our investigation revealed an association between the scores and tumor stages as well as the prognosis of patients, as determined by Kaplan–Meier survival analysis and correlation analysis. In addition, lncRNAs are essential regulators of numerous physiological and pathological processes. They also have vital functions in controlling the FA metabolism of different types of malignancies through ceRNA-related mechanisms.^[33–35]^ Xie et al discovered that PVT1 enhances the growth of ccRCC cells by acting as a sponge for miR-328-3p, resulting in the upregulation of FAM193B and the activation of the MAPK/ERK and PI3K/AKT pathways.^[51]^ Zhang et al^[52]^ conducted a recent study which found that PVT1 levels were significantly increased in ccRCC tissues. Furthermore, patients with ccRCCs who exhibited high PVT1 expression had a more unfavorable prognosis. PVT1 inhibited the degradation of HIF2α protein by inhibiting ubiquitination. This interaction between PVT1 and HIF2α increased the stability of HIF2α and had a significant role in the formation and progression of ccRCC. Furthermore, in the context of colorectal cancer (CRC), lncRNA SNHG16 can potentially serve as a target for ceRNA against as many as 26 families of miRNAs, hence influencing genes relevant to lipid metabolism.^[53]^ Subsequently, we detected RNAs that were expressed differently and constructed a ceRNA network associated with FA metabolism. While previous studies have examined ceRNA networks in ccRCC, our research is the first to establish a ceRNA network specifically connected to FA metabolism in ccRCC. Our main focus was on the lncRNAs inside the ceRNA network. Remarkably, apart from the previously mentioned PVT1 and SNHG16, numerous other lncRNAs have been extensively studied and found to have significant involvement in FA metabolism., such as PCA3,^[54]^ HAGLROS,^[55]^ NEAT1,^[56]^ MACC1-AS1,^[35]^ H19,^[57]^ HAGLR,^[58]^ and MALAT1.^[59]^ The legitimacy of the FA metabolism-related ceRNA network that we have constructed is well demonstrated by this evidence. Ultimately, a total of fifteen lncRNAs were selected from the ceRNA network to create a predictive signature specifically for ccRCC. While other prognostic signatures based on lncRNA have been developed for ccRCC, our study possesses exceptional strengths. Our created signature specifically targets lncRNAs that are involved in FA metabolism. This signature is designed to aid in the development of therapies that target FA metabolism in ccRCC. In addition, unlike previous studies that used co-expression methodologies, we discovered important DElncRNAs that were differentially expressed by building a ceRNA network. Afterwards, we performed a sequence of regression studies to identify lncRNAs associated with OS, which resulted in a notable enhancement in the precision of the signature. Furthermore, the signature has undergone verification in both the TCGA internal validation cohort and the E-MTAB-1980 validation cohort, thereby confirming its clinical practicability. The FAM-LPM was developed using fifteen prognostic FAM-associated lncRNAs, namely, CYTOR, LINC00894,^[60]^ LINC01126,^[61]^ AGAP2-AS1,^[62]^ MIAT,^[63]^ MIRLET7BHG, SNHG4, AATBC, WT1-AS,^[64]^ SNHG17, C3orf35, TCL6,^[65]^ MAPT-AS1, LINC00271, and BDNF-AS, some of these lncRNAs were identified as immune-related by Pearson’s correlation test and are closely associated with cancer etiology and the prognosis of cancer patients. Compelling evidence has demonstrated a close connection between immunology and metabolism in cancer, as indicated by studies.^[66–69]^ It is hypothesized that lncRNA may influence the immune response of ccRCC by modulating FA metabolism. However, additional investigations are need to confirm this hypothesis. Previous study indicated that CYTOR,^[70]^ MIRLET7BHG,^[71]^ SNHG17,^[72]^ and MAPT-AS1^[73]^ were autophagy-related lncRNAs by Pearson’s correlation test. Autophagy has the ability to control FA metabolism, as evidenced by studies,^[74,75]^ which is probably why these lnc RNAs were implicated in our ceRNA network connected to FA metabolism. Moreover, the administration of berberine enhances cardiac hypertrophy by activating autophagy through the mediation of lnc RNA MIAT.^[76]^ SNHG4^[77]^ and BDNF-AS^[78]^ were m6A-related lncRNAs. Previous studies have indicated that dendritic cells regulate the immune response against tumors by means of mRNA m6A methylation and YTHDF1.^[79]^ Furthermore, there is evidence to show that LINC00894 is a specific lncRNA found in ccRCC tumors. It has been observed to be more active in tumor tissues and is associated with OS.^[80]^ LINC00894 potentially modulates the advancement of cancer, spread to other parts of the body, and resistance to drugs through ceRNA-associated processes in ccRCC.^[60]^ The lncRNA AGAP2-AS1, produced by MSCs, enhances the characteristics of stemness and resistance to trastuzumab in breast cancer by controlling the expression of CPT1 and the oxidation of FA.^[81]^

The functional lncRNA cytoskeleton regulator lnc RNA (CYTOR), also referred to as Linc00152, was previously discovered as a gene with a length of 828 base pairs situated on the human chromosome 2p11.2,^[82]^ and the aberrant expression of this gene is associated with the inflammatory response and apoptosis.^[83]^ CYTOR exhibits various cancer-related processes, such as invasion, metastasis, malignant proliferation, glycolysis, and inflammatory response. Moreover, there is a significant association between the deregulation of CYTOR and many clinicopathological characteristics, such as tumor stage, lymph node metastases, infiltration, and adverse prognosis in patients with tumors.^[84]^ CYTOR expression has found to be increased in various cancer forms, including gastric cancer, breast cancer, hepatocellular carcinoma, colon cancer, lung adenocarcinoma, esophageal squamous cell carcinoma, and RCC,^[85–88]^ where CYTOR expression functions as an oncogenic lncRNA. Nevertheless, the specific biological function and the underlying mechanism by which CYTOR influences the proliferation and programmed cell death of ccRCC cells have not been determined. Our research revealed that CYTOR dramatically decreased the expression of PPARA, a key regulator of lipid homeostasis.^[89]^ It is hypothesized that CYTOR may control FA metabolism in ccRCC via reducing the expression of PPARA. Furthermore, as previously stated, CYTOR also governs FA metabolism via autophagy and immunityimmunity.^[70]^ All of these examples demonstrate the significance of CYTOR in FA metabolism, however additional investigations are required to confirm these hypotheses. An investigation was undertaken to uncover the function of lncRNAs related to FA metabolism within the context of tumor immune microenvironment in ccRCC. This investigation involved analyzing the TIMER database, utilizing the ESTIMATE algorithm, employing the ssGSEA approach, and utilizing CIBERSORT. The results indicated that the high-risk score group had a significant increase in immune cell infiltration and a more pronounced state of immunological activation. This could potentially facilitate immune evasion and expedite tumor advancement. In individuals at high risk, these cytotoxic immune cells may already exhibit impaired function. In a recent report,^[90]^ flow cytometric examination demonstrated the presence of exhausted T-cells in advanced disease, as shown by the high expression of PD-1 and CD39 on newly extracted immune cells, as well as their reduced production of inflammatory cytokines upon stimulation in vitro. Glucose uptake deficiencies and alterations in the mitochondrial phenotype were observed as common indications of metabolic insufficiency in fatigued T-cells from late-stage disease. There is compelling data indicating that macrophages play a significant role in promoting the process of epithelial-mesenchymal transition and invasiveness in tumor cells. Additionally, they stimulate a robust regulatory T cell response, which provides protection to tumor cells against adaptive immunity.^[69,91]^

Furthermore, our findings demonstrate that even after accounting for traditional clinical variables, the prognostic signature of FA metabolism-related lncRNAs remains an independent factor in predicting the prognosis of patients with ccRCC. This suggests that the signature has the capacity to enhance the predictive accuracy of traditional clinical variables. Hence, we developed a nomogram that incorporated both the signature and clinical factors. Crucially, we confirmed the findings in both the TCGA internal validation cohort and the E-MTAB-1980 validation cohort, demonstrating the exceptional capacity of the prognostic model to be replicated and trusted.

The significance of our investigation lies in the revelation of a ceRNA network associated to FA metabolism, providing distinct insights into the prognosis and treatment of ccRCC. Nevertheless, this investigation has some constraints. Currently, we are conducting more investigations both in vivo and in vitro to gain a better understanding of the ceRNA-related mechanisms involved in FA metabolism. Our focus is on unraveling the intricacies of the ceRNA network. Furthermore, the data available on ccRCC from the TCGA database is constrained and deficient, thereby compromising the predictive precision of this nomogram. However, it is challenging to identify a suitable cohort in alternative databases for the purpose of validating this nomogram. Furthermore, it is necessary to do additional validation using ccRCC cohorts with bigger sample counts in order to further validate the lncRNA signature and nomogram, despite already validating them in the TCGA internal cohort and E-MTAB-1980 external cohort. Further investigation is required to elucidate the mechanisms that link the development of ccRCC with the characteristic lncRNAs and their target genes. Nevertheless, our research provides a well-informed basis for further inquiries.

## 5. Conclusion

In summary, a FA metabolism-related ceRNA network was constructed for the first time in the current study, and this network may elucidate the molecular regulatory mechanism of FA metabolism in ccRCC. Moreover, this study contributes to our understanding of the regulation of FA metabolism-related lncRNAs in ccRCC progression and provides novel potential biomarkers for prognosis and therapy.

## Author contributions

**Conceptualization:** Zhi-Qiang Wang.

**Data curation:** Ming-Qing Zhang.

**Funding acquisition:** Shanchun Guo.

**Investigation:** Ming-Qing Zhang, Zhi-Qiang Wang, Shanchun Guo.

**Methodology:** Ming-Qing Zhang, Bai-Zhi Yang, Zhi-Qiang Wang, Shanchun Guo.

**Validation:** Bai-Zhi Yang.

**Writing – original draft:** Zhi-Qiang Wang.

**Writing – review & editing:** Shanchun Guo.

## Supplementary Material












