# Supplementary material for: Fatty acid metabolism-related lncRNAs are potential biomarkers for survival prediction in clear cell renal cell carcinoma
Source: Medicine (Baltimore). 2024 Feb 23;103(8):e37207. doi: 10.1097/MD.0000000000037207 (PMC11309608; doi:10.1097/MD.0000000000037207)
Supplement: Supplementary file 1 [file medi-103-e37207-s001.pdf]

FADS2
RAP1GDS1
SDHA
ALDH2
ACSBG1
HSD17B4
HACD1
HSD17B3
PTGES2
DLD
CBR1
ADIPOR2
HPGD
CD1D
ERP29
PTGES
AADAT
PDHA1
ACAA2
ACSF3
MAOA
PRDX6
CD36
PRKAG2
ACADVL
ELOVL2
ALOX15B
PON1
MDH1
PECR
PPT2
GPX1
ENO3
ACBD4
CA6
CEL
CYP4F8
ACBD6

MORC2
SDHC
UBE2L6
CYP2C9
CYP4A22
ADH1A
DPEP3
ACBD5
AWAT1
ALOXE3
SLC25A1
EHHADH
ACACA
ALDH1A1
ACAA1
CA4
OLAH
PRKAA2
ACOT7
ACOT8
ABCD1
ACSL3
GLUL
ACOT9
NCAPH2
ADH5
HACD2
OSTC
CYP1B1
CYP4F22
FADS1
HMGCS2
IDH3G
ALOX15
MECR
CPOX
HSD17B11
ACSM6
HACD4
FAAH
CYP2U1

NTHL1
MIX23
SDHD
HSPH1
KMT5A
MID1IP1
ALDH1B1
PPARD
HTD2
SUCLA2
ADH4
UROD
PTGDS
HADHA
ABCC1
PTGR1
DHCR24
HCCS
FABP2
GPD1
PHYH
CYP2C8
ECI1
CYP2J2
RXRA
THEM4
FABP1
PDHB
PTGR2
ACOXL
NDUFAB1
CBR3
MCEE
PTGES3
CRYZ
PCTP
THEM5
CPT1B
LTA4H
CYP8B1
ADH1C

HSD17B8
BLVRA
SUCLG2
THRSP
ACOX3
ECH1
CYP1A2
GCDH
ELOVL3
PON3
TDO2
ACADS
SCD5
YWHAH
GGT5
TBXAS1
AMACR
PCCA
ACOT11
PTGS1
EPHX1
ACSBG2
ACSF2
ALDH3A1
ME1
FASN
DPEP1
GPX2
GAD2
AKR1C3
CYP4F3
MAPKAPK2
CBR4
MCAT
ODC1
ALAD
CIDEA
ELOVL4
CA2
RDH11
AOC3

GGT1
UROS
CYP2C19
LDHA
ACADM
ACOT13
HADHB
ADH6
ACADSB
ACSL1
ACAT1
G0S2
ELOVL1
CROT
GABARAPL1
ALOX5AP
NSDHL
FMO1
ACAT2
ALOX5
PTS
TECRL
GPX4
CPT1A
FAAH2
ECHS1
GSTZ1
ACSS1
HACD3
IDH3B
GRHPR
FH
ETFDH
ACSM3
PLA2G4A
ACAD10
HADH
ACBD7
SCP2
AUH
D2HGDH

SLC22A5
H2AZ1
SMS
XIST
ALOX12B
ELOVL5
NUDT7
ALDOA
VNN1
CYP4B1
MMUT
ACO2
UGDH
HSD17B12
MDH2
PPT1
DPEP2
EPHX2
ACOT6
PON2
PSME1
ACOT4
PTGS2
PTPRG
HAO2
ACOT2
SLC27A3
SERINC1
DLST
HSD17B10
ACLY
TECR
HMGCS1
ECI2
BPHL
METAP1
APEX1
ELOVL6
MIF
RETSAT
NUDT19

HPGDS
HSDL2
ADSL
PCCB
ACSL5
SCD
RDH16
PRXL2B
DECR2
SLC25A20
ACOX1
ACSL6
ACSL4
AQP7
IL4I1
ACADL
PTGIS
ALDH3A2
REEP6
HMGCL
ALDH7A1
ACAD11
INMT
ACACB
CPT1C
CYP4F11
ADH1B
ACOX2
DECR1
ENO2
CYP4F2
IDI1
ALDH9A1
HIBCH
LTC4S
ELOVL7
ALOX12
MMAA
NBN
LGALS1
GAPDHS

MGLL
SLC27A2
PPARA
HSP90AA1
MLYCD
CYP1A1
PCBD1
SLC25A17
BCKDHB
DBI
SUCLG1
CPT2
ACOT12
HACL1
ADH7
GPD2
TP53INP2
IDH1
S100A10
CRAT
HSD17B7
CYP4A11
ACOT1
PRKAB2
BMPR1B
