# Supplementary material for: Fatty acid metabolism-related lncRNAs are potential biomarkers for survival prediction in clear cell renal cell carcinoma
Source: Medicine (Baltimore). 2024 Feb 23;103(8):e37207. doi: 10.1097/MD.0000000000037207 (PMC11309608; doi:10.1097/MD.0000000000037207)
Supplement: Supplementary file 3 [file medi-103-e37207-s003.pdf]

CYTOR
RFPL3S
GUSBP11
BMS1P4
C9orf139
PDXDC2P-NPIPB14P
RRN3P2
ZNF503-AS2
LINC02656
HCG27
LINC00894
DPP9-AS1
LINC01126
GLIS3-AS1
IBA57-DT
DTX2P1-UPK3BP1-PMS2P11
AGAP2-AS1
MIAT
MIRLET7BHG
SNHG4
AATBC
TCL6
WDFY3-AS2
PCED1B-AS1
LINC00173
GABPB1-AS1
SNHG3
SSBP3-AS1
LHFPL3-AS2
ARHGAP27P1-BPTFP1-
KPNA2P3
WT1-AS
LINC00115
LINC00937
FAM13A-AS1
SNHG20
SNHG17
INE1
C3orf35
LINC00265
CDKN2B-AS1

MALAT1
N4BP2L2-IT2
PVT1
MIR4435-2HG
DHRS4-AS1
MAPT-AS1
ASMTL-AS1
SCGB1B2P
LINC00271
BDNF-AS
H1-10-AS1
LINC00893
LINC00174
