# Supplementary material for: Fatty acid metabolism-related lncRNAs are potential biomarkers for survival prediction in clear cell renal cell carcinoma
Source: Medicine (Baltimore). 2024 Feb 23;103(8):e37207. doi: 10.1097/MD.0000000000037207 (PMC11309608; doi:10.1097/MD.0000000000037207)
Supplement: Supplementary file 4 [file medi-103-e37207-s004.pdf]

ELOVL2	MAFTRR	0.74860539	6.61E-98	postive
ACSBG2	MAFTRR	0.64251709	4.38E-64	postive
CYP4B1	MAFTRR	0.81567278	9.71E-130	postive
FASN	MAFTRR	0.72562684	3.09E-89	postive
PDHB	FGD5-AS1	0.60046142	4.12E-54	postive
HACD3	FGD5-AS1	0.52512792	1.56E-39	postive
HACL1	FGD5-AS1	0.64595059	5.71E-65	postive
RDH11	FGD5-AS1	0.51265793	1.86E-37	postive
GABARAPL1	FGD5-AS1	0.52182961	5.63E-39	postive
D2HGDH	AC092296.2	0.51881486	1.80E-38	postive
ALOX12	AC092296.2	0.57746392	2.95E-49	postive
ACOT6	AC092794.2	0.54703356	2.14E-43	postive
ALOX12	AC005746.1	0.55345287	1.40E-44	postive
ELOVL2	AC009119.1	0.81333182	2.04E-128	postive
ACSBG2	AC009119.1	0.65120286	2.41E-66	postive
CYP4B1	AC009119.1	0.86488703	7.28E-163	postive
FASN	AC009119.1	0.80868103	7.63E-126	postive
DPEP2	AC011899.2	0.62092928	8.92E-59	postive
TBXAS1	AC011899.2	0.60258271	1.40E-54	postive
ELOVL2	AC005776.2	0.71779399	1.77E-86	postive

ACSBG2	AC005776.2	0.72064638	1.79E-87	postive
CYP4B1	AC005776.2	0.74011471	1.35E-94	postive
FASN	AC005776.2	0.69527752	4.71E-79	postive
CPT1B	PXN-AS1	0.5108079	3.71E-37	postive
D2HGDH	PXN-AS1	0.54053643	3.21E-42	postive
BMPR1B	MAN2A1-DT	0.6012073	2.83E-54	postive
RDH11	MAN2A1-DT	0.50904238	7.15E-37	postive
GABARAPL1	MAN2A1-DT	0.53783563	9.71E-42	postive
ACACB	MAN2A1-DT	0.51865605	1.91E-38	postive
ACOT6	AP000866.6	0.63781092	6.84E-63	postive
CPT1B	AL162586.1	0.61304888	6.12E-57	postive
D2HGDH	AL162586.1	0.61381972	4.07E-57	postive
ALOX12	AL162586.1	0.63618997	1.74E-62	postive
ELOVL2	AC010463.2	0.83183164	2.06E-139	postive
ACSBG2	AC010463.2	0.67878817	4.89E-74	postive
CYP4B1	AC010463.2	0.89546592	6.64E-191	postive
FASN	AC010463.2	0.81857486	2.10E-131	postive
BMPR1B	AL161804.1	0.5153488	6.73E-38	postive
D2HGDH	STPG3-AS1	0.52697594	7.55E-40	postive
ELOVL2	AC008543.4	0.76964572	1.03E-106	postive
ACSBG2	AC008543.4	0.67471251	7.56E-73	postive
CYP4B1	AC008543.4	0.84055666	4.47E-145	postive

FASN	AC008543.4	0.73982101	1.75E-94	postive
CPT1B	AC084018.2	0.54043322	3.35E-42	postive
ALOX12	AC084018.2	0.63213849	1.76E-61	postive
CPT1B	CTBP1-AS	0.60025567	4.58E-54	postive
D2HGDH	CTBP1-AS	0.56141631	4.33E-46	postive
ALOX12	CTBP1-AS	0.68777621	9.92E-77	postive
ALOX12	AP001893.1	0.58871672	1.39E-51	postive
ALOX12	AP000907.2	0.66395408	8.47E-70	postive
ACSBG2	AP000907.2	0.5672273	3.24E-47	postive
ELOVL2	LINC02626	0.81431757	5.69E-129	postive
ACSBG2	LINC02626	0.70005666	1.43E-80	postive
CYP4B1	LINC02626	0.87366989	3.70E-170	postive
FASN	LINC02626	0.80703968	5.93E-125	postive
CPT1B	AC008764.8	0.68073464	1.30E-74	postive
D2HGDH	AC008764.8	0.63061948	4.16E-61	postive
ALOX12	AC008764.8	0.69765034	8.38E-80	postive
SDHD	AC016866.1	0.54277488	1.27E-42	postive
HACD3	AC016866.1	0.56815586	2.13E-47	postive
AUH	AC016866.1	0.53255371	8.23E-41	postive
ELOVL4	AC016866.1	0.67713599	1.49E-73	postive
D2HGDH	LINCR-0001	0.51752922	2.94E-38	postive

ALOX12	LINCR-0001	0.59222864	2.50E-52	postive
ELOVL2	AC107294.1	0.83225804	1.11E-139	postive
ACSBG2	AC107294.1	0.65524074	2.03E-67	postive
CYP4B1	AC107294.1	0.89035461	1.19E-185	postive
FASN	AC107294.1	0.82835441	3.02E-137	postive
RDH11	LINC00885	0.59491964	6.62E-53	postive
GABARAPL1	LINC00885	0.55492717	7.39E-45	postive
ALOX12	AC004771.4	0.67082353	9.90E-72	postive
CPT1B	MED8-AS1	0.60796909	8.78E-56	postive
D2HGDH	AP000345.2	0.5716283	4.38E-48	postive
ACOT6	AL078581.4	0.58131503	4.83E-50	postive
ACOT8	POLR2J4	0.56880862	1.58E-47	postive
ELOVL2	AC092756.1	0.81958707	5.41E-132	postive
ACSBG2	AC092756.1	0.66165964	3.65E-69	postive
CYP4B1	AC092756.1	0.87690106	5.57E-173	postive
FASN	AC092756.1	0.80892234	5.63E-126	postive
ELOVL2	AC010618.2	0.75573807	8.60E-101	postive
ACSBG2	AC010618.2	0.71905976	6.42E-87	postive
CYP4B1	AC010618.2	0.81733628	1.09E-130	postive
FASN	AC010618.2	0.7513583	5.22E-99	postive
CPT1B	AC009133.1	0.50100354	1.36E-35	postive

D2HGDH	AC009133.1	0.67348043	1.71E-72	postive
ACADVL	AC009133.1	0.55987797	8.53E-46	postive
ALOX12	AC009133.1	0.56335054	1.84E-46	postive
ACOT6	AF230666.1	0.50617237	2.06E-36	postive
ELOVL2	RCCD1-AS1	0.74882294	5.42E-98	postive
ACSBG2	RCCD1-AS1	0.68944073	3.07E-77	postive
CYP4B1	RCCD1-AS1	0.77896703	6.33E-111	postive
FASN	RCCD1-AS1	0.74280329	1.25E-95	postive
LGALS1	CYTOR	0.56266309	2.49E-46	postive
ELOVL2	AC100830.1	0.69180182	5.74E-78	postive
ACSBG2	AC100830.1	0.72696409	1.02E-89	postive
CYP4B1	AC100830.1	0.74646494	4.65E-97	postive
FASN	AC100830.1	0.66497639	4.40E-70	postive
CPT1B	AC005519.1	0.55253584	2.07E-44	postive
D2HGDH	AC005519.1	0.57518097	8.54E-49	postive
ALOX12	AC005519.1	0.78961898	5.40E-116	postive
SDHD	PDE11A-AS1	0.5169164	3.71E-38	postive
PHYH	PDE11A-AS1	0.56280891	2.34E-46	postive
HACD3	PDE11A-AS1	0.58429015	1.17E-50	postive
ELOVL4	PDE11A-AS1	0.61650897	9.71E-58	postive
RDH11	PDE11A-AS1	0.51430765	9.97E-38	postive

GABARAPL1	PDE11A-AS1	0.63861847	4.28E-63	postive
ALOX12	AC107884.1	0.62356368	2.11E-59	postive
EHHADH	LINC02027	0.55553047	5.69E-45	postive
CRYZ	LINC02027	0.54130809	2.33E-42	postive
ACADM	LINC02027	0.52492316	1.69E-39	postive
HIBCH	LINC02027	0.51040867	4.30E-37	postive
SLC27A2	LINC02027	0.53204277	1.01E-40	postive
ALOX12	AC027796.5	0.55395077	1.13E-44	postive
ELOVL2	AC044840.1	0.74032298	1.13E-94	postive
ACSBG2	AC044840.1	0.66792828	6.55E-71	postive
CYP4B1	AC044840.1	0.80587149	2.53E-124	postive
FASN	AC044840.1	0.72268246	3.45E-88	postive
ACOT6	AL589863.1	0.62654993	4.06E-60	postive
ELOVL2	AC103769.1	0.67078889	1.01E-71	postive
ACSBG2	AC103769.1	0.66526281	3.66E-70	postive
CYP4B1	AC103769.1	0.73261666	8.88E-92	postive
FASN	AC103769.1	0.65721668	5.94E-68	postive
HSD17B3	KMT2E-AS1	0.50959654	5.82E-37	postive
CPT1B	KMT2E-AS1	0.57253763	2.89E-48	postive
D2HGDH	KMT2E-AS1	0.60475878	4.61E-55	postive

ELOVL2	TCF4-AS2	0.77584774	1.71E-109	postive
ACSBG2	TCF4-AS2	0.65361423	5.52E-67	postive
CYP4B1	TCF4-AS2	0.84288591	1.20E-146	postive
FASN	TCF4-AS2	0.76332355	5.68E-104	postive
ACOT6	ASTN2-AS1	0.81894583	1.28E-131	postive
BMPR1B	ASTN2-AS1	0.57122059	5.28E-48	postive
CPT1B	AL133215.1	0.52060591	9.03E-39	postive
D2HGDH	AL133215.1	0.50101934	1.35E-35	postive
ALOX12	AL133215.1	0.60652102	1.86E-55	postive
ELOVL2	AC011479.2	0.59649945	3.02E-53	postive
ALOX12	AC011479.2	0.54617091	3.08E-43	postive
ACSBG2	AC011479.2	0.64012637	1.78E-63	postive
CYP4B1	AC011479.2	0.64163015	7.37E-64	postive
FASN	AC011479.2	0.61511044	2.05E-57	postive
ALOX12	AC008555.2	0.60240024	1.54E-54	postive
ACAA2	U91328.1	0.59628971	3.35E-53	postive
ETFDH	U91328.1	0.52238554	4.54E-39	postive
ALDH3A2	U91328.1	0.56807616	2.21E-47	postive
HIBCH	U91328.1	0.60860467	6.31E-56	postive
SLC27A2	U91328.1	0.52680247	8.08E-40	postive

MMUT	U91328.1	0.61660799	9.20E-58	postive
PRKAG2	LINC01187	0.61101387	1.79E-56	postive
PDHB	LINC01187	0.5534198	1.42E-44	postive
HACD3	LINC01187	0.54545815	4.15E-43	postive
AUH	LINC01187	0.5335472	5.52E-41	postive
HACL1	LINC01187	0.55711437	2.86E-45	postive
BMPR1B	LINC01187	0.78895353	1.14E-115	postive
ELOVL4	LINC01187	0.58679884	3.52E-51	postive
RDH11	LINC01187	0.63996979	1.95E-63	postive
GABARAPL1	LINC01187	0.72704405	9.58E-90	postive
ACACB	LINC01187	0.63295499	1.11E-61	postive
ELOVL2	USP12-AS2	0.82979319	3.89E-138	postive
ACSBG2	USP12-AS2	0.66967437	2.10E-71	postive
CYP4B1	USP12-AS2	0.88515727	1.44E-180	postive
FASN	USP12-AS2	0.82509306	2.94E-135	postive
ELOVL2	AC008115.1	0.83213931	1.32E-139	postive
ACSBG2	AC008115.1	0.64543841	7.76E-65	postive
CYP4B1	AC008115.1	0.89488023	2.74E-190	postive
FASN	AC008115.1	0.82891272	1.37E-137	postive
ELOVL2	AC078962.1	0.59441985	8.48E-53	postive
ACSBG2	AC078962.1	0.62414817	1.53E-59	postive
CYP4B1	AC078962.1	0.6505748	3.53E-66	postive

FASN	AC078962.1	0.57317425	2.16E-48	postive
HSD17B3	AL513218.1	0.59687119	2.51E-53	postive
CPT1B	AL513218.1	0.6459037	5.88E-65	postive
D2HGDH	AL513218.1	0.55788816	2.04E-45	postive
ALOX12	AL513218.1	0.50487937	3.31E-36	postive
ELOVL2	ETV7-AS1	0.53083545	1.64E-40	postive
ACSBG2	ETV7-AS1	0.5752844	8.14E-49	postive
CYP4B1	ETV7-AS1	0.56957075	1.12E-47	postive
FASN	ETV7-AS1	0.51443592	9.50E-38	postive
CPT1B	AC027682.4	0.51446126	9.41E-38	postive
CPT1B	AL391684.1	0.63597333	1.97E-62	postive
D2HGDH	AL391684.1	0.57317294	2.16E-48	postive
ALOX12	AL391684.1	0.66635069	1.82E-70	postive
ELOVL2	AC022431.1	0.58388694	1.42E-50	postive
CYP4B1	AC022431.1	0.66413603	7.54E-70	postive
FASN	AC022431.1	0.58851751	1.53E-51	postive
ELOVL2	AC027373.1	0.51846398	2.05E-38	postive
HSD17B3	AC027373.1	0.51867061	1.90E-38	postive
ACSBG2	AC027373.1	0.59876161	9.72E-54	postive
CYP4B1	AC027373.1	0.50746299	1.28E-36	postive
ALOX12	AL035416.1	0.55484664	7.65E-45	postive

ALOX12	AC134407.1	0.52674075	8.28E-40	postive
ENO3	C22orf34	0.62370653	1.95E-59	postive
ELOVL2	MIR583HG	0.55650901	3.72E-45	postive
ACSBG2	MIR583HG	0.58159835	4.22E-50	postive
CYP4B1	MIR583HG	0.57309956	2.23E-48	postive
FASN	MIR583HG	0.51558981	6.14E-38	postive
ACOT6	AL161457.2	0.8652854	3.49E-163	postive
BMPR1B	AL161457.2	0.63433304	5.05E-62	postive
ELOVL2	RNF217-AS1	0.59800163	1.42E-53	postive
ACSBG2	RNF217-AS1	0.62696338	3.22E-60	postive
CYP4B1	RNF217-AS1	0.65633378	1.03E-67	postive
FASN	RNF217-AS1	0.56227257	2.96E-46	postive
ELOVL2	AC092802.2	0.6884395	6.22E-77	postive
ALOX12	AC092802.2	0.55621287	4.23E-45	postive
ACSBG2	AC092802.2	0.7161486	6.52E-86	postive
CYP4B1	AC092802.2	0.75541016	1.17E-100	postive
FASN	AC092802.2	0.67091847	9.30E-72	postive
ELOVL2	AC008764.6	0.73888768	3.97E-94	postive
ACSBG2	AC008764.6	0.68645781	2.50E-76	postive
CYP4B1	AC008764.6	0.80063239	1.49E-121	postive
FASN	AC008764.6	0.75397591	4.54E-100	postive
ACSBG1	AC025811.1	0.55368353	1.26E-44	postive

ELOVL2	LINC01252	0.65784691	4.01E-68	postive
ACSBG2	LINC01252	0.66070247	6.68E-69	postive
CYP4B1	LINC01252	0.69299298	2.45E-78	postive
FASN	LINC01252	0.63316238	9.86E-62	postive
RDH11	LINC01489	0.52870756	3.81E-40	postive
GABARAPL1	LINC01489	0.57368726	1.70E-48	postive
ACSBG2	AC079793.1	0.50431354	4.08E-36	postive
ELOVL2	AC121247.1	0.54319674	1.07E-42	postive
ACSBG2	AC121247.1	0.59917113	7.91E-54	postive
CYP4B1	AC121247.1	0.57847092	1.84E-49	postive
FASN	AC121247.1	0.51083477	3.67E-37	postive
ALOX12	HIF1A-AS3	0.56205709	3.26E-46	postive
DPEP2	MACORIS	0.52423948	2.21E-39	postive
ELOVL2	AC013549.1	0.82666254	3.29E-136	postive
ACSBG2	AC013549.1	0.67258641	3.10E-72	postive
CYP4B1	AC013549.1	0.8910523	2.37E-186	postive
FASN	AC013549.1	0.81948804	6.18E-132	postive
ACOT6	AC079035.1	0.66128513	4.62E-69	postive
ELOVL4	OLMALINC	0.56381361	1.49E-46	postive
GABARAPL1	OLMALINC	0.57618106	5.37E-49	postive
PRKAG2	AC010776.3	0.55004407	6.00E-44	postive

ACOT6	AC010776.3	0.52524854	1.49E-39	postive
BMPR1B	AC010776.3	0.59338493	1.42E-52	postive
RDH11	AC010776.3	0.51135946	3.02E-37	postive
GABARAPL1	AC010776.3	0.72015879	2.66E-87	postive
ACACB	AC010776.3	0.58035587	7.60E-50	postive
ELOVL2	AC007272.1	0.82237036	1.25E-133	postive
ACSBG2	AC007272.1	0.67690143	1.75E-73	postive
CYP4B1	AC007272.1	0.88833284	1.21E-183	postive
FASN	AC007272.1	0.81748364	8.94E-131	postive
ELOVL2	AC067747.1	0.56501896	8.73E-47	postive
ALOX12	AC067747.1	0.52714854	7.05E-40	postive
ACSBG2	AC067747.1	0.65079796	3.08E-66	postive
CYP4B1	AC067747.1	0.62335724	2.37E-59	postive
FASN	AC067747.1	0.53115878	1.44E-40	postive
ACOT6	AL355073.2	0.83365984	1.43E-140	postive
BMPR1B	AL355073.2	0.59731472	2.01E-53	postive
HSD17B3	AC009120.2	0.501036	1.34E-35	postive
CPT1B	AC009120.2	0.68298786	2.77E-75	postive
D2HGDH	AC009120.2	0.62142766	6.80E-59	postive
ALOX12	AC009120.2	0.62211449	4.67E-59	postive
PRKAG2	AC104958.2	0.55683044	3.24E-45	postive

GABARAPL1	AC104958.2	0.50822944	9.66E-37	postive
ACOT6	AC104170.1	0.67735639	1.29E-73	postive
HSD17B3	AC026471.4	0.52629284	9.88E-40	postive
CPT1B	AC026471.4	0.51278771	1.77E-37	postive
ALOX12	AC026471.4	0.56824667	2.04E-47	postive
ACSBG2	AC026471.4	0.6075902	1.07E-55	postive
ACOT6	AC025271.4	0.76112352	4.88E-103	postive
BMPR1B	AC025271.4	0.71234778	1.28E-84	postive
ELOVL2	TRIM7-AS1	0.80961823	2.34E-126	postive
ACSBG2	TRIM7-AS1	0.66057424	7.25E-69	postive
CYP4B1	TRIM7-AS1	0.87986236	1.23E-175	postive
FASN	TRIM7-AS1	0.79206001	3.38E-117	postive
ELOVL2	AL645728.1	0.74822795	9.34E-98	postive
ACSBG2	AL645728.1	0.61311012	5.93E-57	postive
CYP4B1	AL645728.1	0.78857682	1.74E-115	postive
FASN	AL645728.1	0.74639168	4.97E-97	postive
ELOVL2	AC012360.1	0.80633257	1.43E-124	postive
ACSBG2	AC012360.1	0.66782392	7.01E-71	postive
CYP4B1	AC012360.1	0.86426874	2.27E-162	postive
FASN	AC012360.1	0.80385733	3.00E-123	postive
ACOT6	AP000766.1	0.60558557	3.01E-55	postive

PRKAA2	AP000766.1	0.52141085	6.62E-39	postive
CBR4	AP000766.1	0.56293217	2.21E-46	postive
ELOVL2	AL157838.1	0.50150219	1.13E-35	postive
ALOX12	AL157838.1	0.5390841	5.83E-42	postive
ACSBG2	AL157838.1	0.62686999	3.40E-60	postive
CYP4B1	AL157838.1	0.52955007	2.73E-40	postive
SDHD	AL035661.1	0.65266595	9.87E-67	postive
PHYH	AL035661.1	0.65286613	8.73E-67	postive
HACD3	AL035661.1	0.70299911	1.61E-81	postive
AUH	AL035661.1	0.57659742	4.42E-49	postive
HACL1	AL035661.1	0.59305006	1.67E-52	postive
BMPR1B	AL035661.1	0.67031263	1.38E-71	postive
ELOVL4	AL035661.1	0.77827528	1.32E-110	postive
RDH11	AL035661.1	0.72980063	9.59E-91	postive
GABARAPL1	AL035661.1	0.65979349	1.18E-68	postive
ACACB	AL035661.1	0.51964375	1.31E-38	postive
ALOX12	AP001178.3	0.63064407	4.10E-61	postive
ALOX12	AC004466.2	0.52351096	2.93E-39	postive
ELOVL2	AC010525.1	0.8271229	1.72E-136	postive
ACSBG2	AC010525.1	0.66987517	1.84E-71	postive
CYP4B1	AC010525.1	0.88849338	8.42E-184	postive

FASN	AC010525.1	0.82257973	9.39E-134	postive
ACOT6	AC034234.1	0.91154965	2.08E-209	postive
BMPR1B	AC034234.1	0.71221069	1.43E-84	postive
ALOX12	AL157871.2	0.53750919	1.11E-41	postive
CPT1B	AL021707.4	0.50255651	7.73E-36	postive
ALOX12	AL021707.4	0.70010424	1.38E-80	postive
ACOT6	IRAG1-AS1	0.77200211	9.24E-108	postive
BMPR1B	IRAG1-AS1	0.57016038	8.57E-48	postive
ALOX12	AC009163.7	0.61945168	1.99E-58	postive
ACOT6	AC005740.3	0.66924231	2.79E-71	postive
ELOVL2	AC010422.4	0.66604708	2.21E-70	postive
ACSBG2	AC010422.4	0.643559	2.37E-64	postive
CYP4B1	AC010422.4	0.71842173	1.07E-86	postive
ACOT6	AC010422.4	0.56574662	6.30E-47	postive
FASN	AC010422.4	0.64454863	1.32E-64	postive
ELOVL2	AL355312.2	0.54263521	1.35E-42	postive
ACSBG2	AL355312.2	0.56967236	1.07E-47	postive
CYP4B1	AL355312.2	0.58691052	3.33E-51	postive
FASN	AL355312.2	0.50438374	3.97E-36	postive
HSD17B3	Z99916.1	0.520695	8.72E-39	postive
BMPR1B	AC126177.3	0.5654938	7.05E-47	postive
ELOVL4	AC126177.3	0.59900029	8.62E-54	postive

D2HGDH	C1RL-AS1	0.61957924	1.86E-58	postive
ACADVL	C1RL-AS1	0.55698098	3.03E-45	postive
ALOX12	C1RL-AS1	0.71119023	3.15E-84	postive
ACADL	C1RL-AS1	0.51284884	1.73E-37	postive
PHYH	SEMA3B-AS1	0.50063292	1.55E-35	postive
NDUFAB1	SEMA3B-AS1	0.50826447	9.54E-37	postive
HACL1	SEMA3B-AS1	0.51075108	3.79E-37	postive
CYP1A2	SEMA3B-AS1	0.52081743	8.32E-39	postive
GABARAPL1	SEMA3B-AS1	0.51446797	9.39E-38	postive
ALOX12	AC138956.1	0.57708119	3.53E-49	postive
ENO3	LINC00906	0.54522022	4.59E-43	postive
PCBD1	LINC00906	0.57489806	9.73E-49	postive
ALOX12	MUC20-OT1	0.52064128	8.91E-39	postive
ACOT6	MUC20-OT1	0.56810316	2.18E-47	postive
ACACB	MUC20-OT1	0.51866395	1.90E-38	postive
ELOVL2	AC005828.4	0.82299741	5.30E-134	postive
ACSBG2	AC005828.4	0.66675992	1.40E-70	postive
CYP4B1	AC005828.4	0.87230033	5.50E-169	postive
FASN	AC005828.4	0.82633925	5.17E-136	postive
ALOX12	AC245884.10	0.53494826	3.14E-41	postive
ACSBG2	AC245884.10	0.5853266	7.14E-51	postive

ACOT6	AC245884.10	0.5124071	2.04E-37	postive
CPT1B	STK24-AS1	0.56009371	7.76E-46	postive
D2HGDH	STK24-AS1	0.51954765	1.36E-38	postive
CPT1B	SLC30A6-DT	0.50848071	8.81E-37	postive
ELOVL2	AC010519.1	0.7503212	1.36E-98	postive
ACSBG2	AC010519.1	0.70549215	2.46E-82	postive
CYP4B1	AC010519.1	0.80126767	6.92E-122	postive
FASN	AC010519.1	0.73686203	2.32E-93	postive
HSD17B3	AC087741.1	0.6229108	3.02E-59	postive
CPT1B	AC087741.1	0.67382251	1.37E-72	postive
D2HGDH	AC087741.1	0.65276261	9.30E-67	postive
ACADVL	AC087741.1	0.54677327	2.39E-43	postive
ALOX12	AC087741.1	0.61301656	6.23E-57	postive
ACOT6	AC024145.1	0.83340085	2.09E-140	postive
BMPR1B	AC024145.1	0.6132302	5.56E-57	postive
ELOVL2	LINC00551	0.78069299	9.99E-112	postive
ACSBG2	LINC00551	0.6179084	4.58E-58	postive
CYP4B1	LINC00551	0.86143658	3.87E-160	postive
FASN	LINC00551	0.77233923	6.53E-108	postive
ALOX12	AC018695.4	0.58817798	1.81E-51	postive
ACOT6	AC130650.1	0.58499158	8.39E-51	postive
HSD17B3	AC084824.5	0.63229647	1.61E-61	postive
CPT1B	AC084824.5	0.50402833	4.52E-36	postive

HSD17B3	RFPL3S	0.54215415	1.64E-42	postive
CPT1B	RFPL3S	0.67086281	9.65E-72	postive
D2HGDH	RFPL3S	0.53425426	4.15E-41	postive
ALOX12	AC135050.5	0.54435512	6.59E-43	postive
ACOT6	AC135050.5	0.55671502	3.40E-45	postive
ACACB	AC135050.5	0.61005435	2.96E-56	postive
ELOVL2	FLVCR1-DT	0.55028023	5.42E-44	postive
ACSBG2	FLVCR1-DT	0.51490434	7.96E-38	postive
CYP4B1	FLVCR1-DT	0.59358146	1.28E-52	postive
FASN	FLVCR1-DT	0.56608805	5.40E-47	postive
ELOVL2	DNAJC27-AS1	0.71728358	2.65E-86	postive
ACSBG2	DNAJC27-AS1	0.67167687	5.65E-72	postive
CYP4B1	DNAJC27-AS1	0.79149175	6.47E-117	postive
FASN	DNAJC27-AS1	0.68403039	1.35E-75	postive
ACOT6	AC092574.2	0.54254331	1.40E-42	postive
ALOX12	AC002563.1	0.55537984	6.08E-45	postive
CBR4	TMEM161B-AS1	0.54174327	1.95E-42	postive
PRKAG2	AC104984.4	0.50737205	1.33E-36	postive
PDHB	AC104984.4	0.50641589	1.89E-36	postive
BMPR1B	AC104984.4	0.70208323	3.18E-81	postive

ELOVL4	AC104984.4	0.61917338	2.31E-58	postive
RDH11	AC104984.4	0.55549954	5.77E-45	postive
GABARAPL1	AC104984.4	0.6623625	2.34E-69	postive
ACACB	AC104984.4	0.58258744	2.64E-50	postive
ACSBG2	AL359878.2	0.55595139	4.74E-45	postive
ALOX12	AC092375.2	0.56575765	6.27E-47	postive
ALOX12	AC022558.1	0.54343093	9.68E-43	postive
ACOT6	AC022558.1	0.53066303	1.75E-40	postive
ELOVL2	AL589739.1	0.80111813	8.29E-122	postive
ACSBG2	AL589739.1	0.65996358	1.06E-68	postive
CYP4B1	AL589739.1	0.87614708	2.58E-172	postive
FASN	AL589739.1	0.79271203	1.60E-117	postive
ELOVL2	AL132639.2	0.74367603	5.73E-96	postive
ACSBG2	AL132639.2	0.63393261	6.35E-62	postive
CYP4B1	AL132639.2	0.80360518	4.08E-123	postive
FASN	AL132639.2	0.75836409	7.03E-102	postive
PON1	DCST1-AS1	0.52262846	4.13E-39	postive
ADH1A	DCST1-AS1	0.51895211	1.70E-38	postive
ADH4	DCST1-AS1	0.51756443	2.90E-38	postive
RDH16	DCST1-AS1	0.53126599	1.38E-40	postive

ALOX12	SMAD9-IT1	0.53464022	3.55E-41	postive
ACACB	AC106791.1	0.50052854	1.61E-35	postive
ELOVL2	AC018557.2	0.64715821	2.78E-65	postive
ACSBG2	AC018557.2	0.63187891	2.04E-61	postive
CYP4B1	AC018557.2	0.70275865	1.92E-81	postive
ACOT6	AC018557.2	0.52792599	5.19E-40	postive
FASN	AC018557.2	0.62488399	1.02E-59	postive
D2HGDH	AL162431.1	0.53404127	4.53E-41	postive
HSD17B3	AL662797.1	0.52254841	4.26E-39	postive
CPT1B	AL662797.1	0.52312291	3.41E-39	postive
HSD17B3	GUSBP11	0.50263591	7.51E-36	postive
CPT1B	GUSBP11	0.63552491	2.55E-62	postive
D2HGDH	GUSBP11	0.75269624	1.50E-99	postive
ACADVL	GUSBP11	0.62370941	1.95E-59	postive
FAAH	GUSBP11	0.52323873	3.26E-39	postive
ALOX12	GUSBP11	0.55490858	7.45E-45	postive
ALOX12	AC093690.1	0.52268455	4.04E-39	postive
ACOT6	AC087071.2	0.73510209	1.06E-92	postive
BMPR1B	AC087071.2	0.59535755	5.33E-53	postive
CBR4	AC087071.2	0.57861499	1.72E-49	postive

FASN	AL589765.4	0.57534853	7.90E-49	postive
ALOX12	BX649601.1	0.50531718	2.82E-36	postive
ACSBG2	BX649601.1	0.50819295	9.80E-37	postive
ALOX12	AC004076.2	0.57138928	4.89E-48	postive
ACSBG2	AC004076.2	0.60095939	3.20E-54	postive
SDHD	CA3-AS1	0.58297708	2.19E-50	postive
HACD3	CA3-AS1	0.5212439	7.06E-39	postive
AUH	CA3-AS1	0.58628607	4.50E-51	postive
BMPR1B	CA3-AS1	0.5606405	6.10E-46	postive
ELOVL4	CA3-AS1	0.58742299	2.60E-51	postive
RDH11	CA3-AS1	0.5321681	9.61E-41	postive
ELOVL2	LINC01976	0.60220954	1.70E-54	postive
ACSBG2	LINC01976	0.50660559	1.76E-36	postive
CYP4B1	LINC01976	0.661162	5.00E-69	postive
FASN	LINC01976	0.59903976	8.45E-54	postive
HSD17B3	AP001160.1	0.64616646	5.02E-65	postive
CPT1B	AP001160.1	0.59723732	2.09E-53	postive
D2HGDH	AP001160.1	0.55159417	3.10E-44	postive
ALOX12	AP001160.1	0.52787561	5.30E-40	postive
ELOVL2	AL731577.1	0.76239192	1.42E-103	postive
ACSBG2	AL731577.1	0.68724093	1.44E-76	postive
CYP4B1	AL731577.1	0.82624382	5.91E-136	postive
FASN	AL731577.1	0.7476886	1.53E-97	postive

ELOVL2	AP000346.1	0.59136626	3.82E-52	postive
ACSBG2	AP000346.1	0.64965162	6.18E-66	postive
CYP4B1	AP000346.1	0.67283333	2.63E-72	postive
FASN	AP000346.1	0.56784623	2.45E-47	postive
ALOX12	AC011337.1	0.50564865	2.50E-36	postive
HSD17B3	AL121852.1	0.52011215	1.09E-38	postive
CYP4B1	AL031727.2	0.55688959	3.15E-45	postive
ALOX12	LACTB2-AS1	0.53181512	1.11E-40	postive
ALOX12	SEMA6A-AS1	0.55895128	1.28E-45	postive
ALOX12	AP001625.2	0.52615225	1.04E-39	postive
PTGS2	AC132872.3	0.57386057	1.57E-48	postive
ELOVL2	LINC01655	0.8150777	2.11E-129	postive
ACSBG2	LINC01655	0.61478788	2.43E-57	postive
CYP4B1	LINC01655	0.86566406	1.73E-163	postive
FASN	LINC01655	0.80989206	1.66E-126	postive
D2HGDH	AL662844.4	0.57625703	5.18E-49	postive
FAAH	AL662844.4	0.50405567	4.48E-36	postive
ALOX12	AL662844.4	0.64635715	4.48E-65	postive
ALOX12	AC093484.4	0.58537672	6.97E-51	postive
ACOT6	AC020661.3	0.71391059	3.79E-85	postive
ALOX12	KDM4A-AS1	0.65975052	1.22E-68	postive

ACSBG2	KDM4A-AS1	0.55335595	1.46E-44	postive
PRKAA2	CASC2	0.52976372	2.51E-40	postive
HSD17B3	SUGT1-DT	0.56692554	3.71E-47	postive
CPT1B	SUGT1-DT	0.63195278	1.96E-61	postive
D2HGDH	SUGT1-DT	0.52088422	8.11E-39	postive
ELOVL2	AL139021.1	0.65464417	2.93E-67	postive
ACSBG2	AL139021.1	0.6098311	3.33E-56	postive
CYP4B1	AL139021.1	0.71662441	4.47E-86	postive
FASN	AL139021.1	0.60105109	3.06E-54	postive
ALOX12	AL049869.2	0.52083041	8.28E-39	postive
CPT1B	Z69706.1	0.55019837	5.62E-44	postive
D2HGDH	Z69706.1	0.61722712	6.60E-58	postive
ALOX12	BMS1P4	0.67251672	3.25E-72	postive
ELOVL2	AC090630.1	0.5038812	4.77E-36	postive
ACSBG2	AC090630.1	0.58054738	6.94E-50	postive
CYP4B1	AC090630.1	0.5616938	3.83E-46	postive
ALOX12	AC116903.2	0.50730062	1.36E-36	postive
HSD17B8	FLJ20021	0.50891645	7.49E-37	postive
ELOVL2	AC090115.1	0.79638976	2.26E-119	postive
ACSBG2	AC090115.1	0.67055575	1.18E-71	postive
CYP4B1	AC090115.1	0.87592985	4.00E-172	postive
FASN	AC090115.1	0.79500171	1.14E-118	postive

ELOVL2	AP001029.2	0.50785574	1.11E-36	postive
ALOX12	AP001029.2	0.6162325	1.13E-57	postive
ACSBG2	AP001029.2	0.63768112	7.37E-63	postive
CYP4B1	AP001029.2	0.54638376	2.82E-43	postive
ELOVL2	AC011443.1	0.81050989	7.57E-127	postive
ACSBG2	AC011443.1	0.70202113	3.33E-81	postive
CYP4B1	AC011443.1	0.87224521	6.13E-169	postive
FASN	AC011443.1	0.79868583	1.51E-120	postive
HSD17B3	AC018809.1	0.53169358	1.16E-40	postive
CPT1B	AC018809.1	0.57096573	5.93E-48	postive
ALOX12	AC018809.1	0.51774094	2.71E-38	postive
ELOVL2	DUSP5-DT	0.63089823	3.56E-61	postive
CYP4B1	DUSP5-DT	0.66643779	1.72E-70	postive
FASN	DUSP5-DT	0.69739719	1.01E-79	postive
ELOVL2	AC114939.1	0.81820771	3.42E-131	postive
ACSBG2	AC114939.1	0.68971957	2.52E-77	postive
CYP4B1	AC114939.1	0.88152396	3.69E-177	postive
FASN	AC114939.1	0.81116286	3.30E-127	postive
HSD17B3	AL158063.1	0.52883601	3.62E-40	postive
ALOX12	AL158063.1	0.52081025	8.34E-39	postive
ELOVL2	AC087164.1	0.78317214	6.83E-113	postive

ACSBG2	AC087164.1	0.70245972	2.40E-81	postive
CYP4B1	AC087164.1	0.84858896	1.34E-150	postive
FASN	AC087164.1	0.77526293	3.15E-109	postive
ELOVL2	LINC01715	0.68544025	5.08E-76	postive
ACSBG2	LINC01715	0.63904085	3.35E-63	postive
CYP4B1	LINC01715	0.71981047	3.52E-87	postive
FASN	LINC01715	0.66154574	3.92E-69	postive
ACOT6	AC093510.1	0.63696471	1.12E-62	postive
ACOT6	AC026470.2	0.67412977	1.11E-72	postive
CPT1B	AC073842.2	0.56947745	1.17E-47	postive
D2HGDH	AC073842.2	0.53111694	1.46E-40	postive
ALOX12	AC073842.2	0.68116661	9.68E-75	postive
ACSBG2	AC073842.2	0.59539234	5.24E-53	postive
ALOX12	AL390066.1	0.57706769	3.55E-49	postive
ACSBG2	AL390066.1	0.64681514	3.41E-65	postive
CYP4B1	AL390066.1	0.5111934	3.21E-37	postive
ELOVL2	AC010618.3	0.74213952	2.26E-95	postive
ACSBG2	AC010618.3	0.71758454	2.09E-86	postive
CYP4B1	AC010618.3	0.80995045	1.54E-126	postive
FASN	AC010618.3	0.74134332	4.57E-95	postive
ACOT6	AC009268.2	0.57090173	6.11E-48	postive

ALOX12	AL133227.1	0.50506317	3.10E-36	postive
ACSBG2	AL133227.1	0.52270296	4.01E-39	postive
ACOT6	AL133227.1	0.50570993	2.44E-36	postive
ELOVL2	AC137630.2	0.82259605	9.19E-134	postive
ACSBG2	AC137630.2	0.66367452	1.01E-69	postive
CYP4B1	AC137630.2	0.88554209	6.17E-181	postive
FASN	AC137630.2	0.82404071	1.26E-134	postive
ELOVL2	MORF4L2-AS1	0.5010182	1.35E-35	postive
ALOX12	MORF4L2-AS1	0.69002719	2.03E-77	postive
ACSBG2	MORF4L2-AS1	0.64000964	1.90E-63	postive
CYP4B1	MORF4L2-AS1	0.57326665	2.07E-48	postive
ALOX12	AC016394.1	0.50042833	1.67E-35	postive
HSD17B3	COA6-AS1	0.50617094	2.06E-36	postive
ACOT6	AP001429.1	0.74006855	1.41E-94	postive
ALOX12	PDC-AS1	0.59529809	5.49E-53	postive
ACSBG2	PDC-AS1	0.52171311	5.89E-39	postive
ALOX12	TMCO1-AS1	0.57863188	1.71E-49	postive
ALOX12	CR936218.1	0.64828955	1.41E-65	postive
ACOT6	AP001350.1	0.67706124	1.57E-73	postive
ALOX12	AC015911.3	0.50245382	8.02E-36	postive
ACOT6	AL121672.2	0.63490026	3.65E-62	postive

CPT1B	AC113139.1	0.60205017	1.84E-54	postive
ACOT6	AC109587.1	0.58595097	5.29E-51	postive
ACSBG2	SOX9-AS1	0.5100956	4.84E-37	postive
CYP4B1	SOX9-AS1	0.56655742	4.38E-47	postive
ALOX12	AC087392.4	0.58146696	4.50E-50	postive
ALOX12	AL139397.1	0.53188509	1.08E-40	postive
PON1	LINC01018	0.86165859	2.60E-160	postive
ADH1A	LINC01018	0.86220828	9.66E-161	postive
ADH4	LINC01018	0.8632757	1.39E-161	postive
FABP1	LINC01018	0.80124208	7.14E-122	postive
ADH1C	LINC01018	0.8641736	2.70E-162	postive
CYP1A2	LINC01018	0.67770602	1.02E-73	postive
RDH16	LINC01018	0.85976327	7.65E-159	postive
ADH1B	LINC01018	0.76581295	4.83E-105	postive
CPT1B	AC005253.1	0.61470278	2.55E-57	postive
D2HGDH	AC005253.1	0.53243469	8.63E-41	postive
ALOX12	AC005253.1	0.74731857	2.14E-97	postive
ALOX12	AL021937.4	0.55932973	1.09E-45	postive
ACSBG2	AL021937.4	0.50560375	2.54E-36	postive
HSD17B3	AC010331.1	0.59244907	2.24E-52	postive
ELOVL2	AC124947.1	0.82499057	3.39E-135	postive
ACSBG2	AC124947.1	0.65023959	4.33E-66	postive

CYP4B1	AC124947.1	0.8828812	2.03E-178	postive
FASN	AC124947.1	0.81659435	2.89E-130	postive
ALOX12	EPS15-AS1	0.52639881	9.47E-40	postive
CBR4	DNAJC3-DT	0.56414836	1.29E-46	postive
ACACB	DNAJC3-DT	0.53592912	2.11E-41	postive
ELOVL2	AP001628.1	0.63279107	1.22E-61	postive
ALOX12	AP001628.1	0.51539722	6.61E-38	postive
ACSBG2	AP001628.1	0.72710963	9.07E-90	postive
CYP4B1	AP001628.1	0.65682714	7.58E-68	postive
FASN	AP001628.1	0.63491535	3.62E-62	postive
HSD17B3	AC079906.1	0.65478511	2.68E-67	postive
SUCLG1	AC010997.4	0.52421776	2.23E-39	postive
ALOX12	AC009495.2	0.54783595	1.53E-43	postive
ACOT6	AC010261.1	0.64688519	3.27E-65	postive
ELOVL2	AP001099.1	0.79811493	2.97E-120	postive
ACSBG2	AP001099.1	0.62975781	6.76E-61	postive
CYP4B1	AP001099.1	0.83393723	9.49E-141	postive
FASN	AP001099.1	0.78945882	6.47E-116	postive
ACOT6	AC078993.1	0.6727321	2.82E-72	postive
BMPR1B	AC078993.1	0.61308869	6.00E-57	postive
ACOT6	AL162171.2	0.84027629	6.88E-145	postive

BMPR1B	AL162171.2	0.63832576	5.07E-63	postive
ALOX12	NSMCE1-DT	0.67332402	1.90E-72	postive
ACSBG2	NSMCE1-DT	0.61445727	2.90E-57	postive
ALOX12	AC025580.3	0.52443086	2.05E-39	postive
ACOT6	PABPC4-AS1	0.69691149	1.44E-79	postive
ELOVL2	AP003774.2	0.71758086	2.09E-86	postive
ACSBG2	AP003774.2	0.61960146	1.83E-58	postive
CYP4B1	AP003774.2	0.75634999	4.81E-101	postive
FASN	AP003774.2	0.72039607	2.20E-87	postive
ACOT6	AC068790.6	0.50871054	8.09E-37	postive
CPT1B	C9orf139	0.56823869	2.05E-47	postive
D2HGDH	C9orf139	0.52114885	7.32E-39	postive
ALOX12	C9orf139	0.63141921	2.65E-61	postive
DPEP2	C9orf139	0.55610308	4.44E-45	postive
CPT1B	AL031714.1	0.67144857	6.57E-72	postive
D2HGDH	AL031714.1	0.61597191	1.29E-57	postive
ALOX12	AL031714.1	0.64493238	1.05E-64	postive
HSD17B3	AC009318.4	0.60377266	7.65E-55	postive
ACSBG2	AC009318.4	0.55835451	1.66E-45	postive
ACOT6	AC090739.1	0.69091652	1.08E-77	postive

ACOT6	AL606834.1	0.58686157	3.41E-51	postive
BMPR1B	AL606834.1	0.61904989	2.47E-58	postive
RDH11	AL606834.1	0.54933541	8.11E-44	postive
GABARAPL1	AL606834.1	0.59429584	9.02E-53	postive
ACACB	AL606834.1	0.5021446	8.97E-36	postive
HSD17B3	AC087289.2	0.57133269	5.02E-48	postive
CPT1B	AC087289.2	0.65940244	1.52E-68	postive
D2HGDH	AC087289.2	0.61262065	7.68E-57	postive
ACADVL	AC087289.2	0.50779016	1.14E-36	postive
ALOX12	AC087289.2	0.52504401	1.61E-39	postive
ALOX12	AC008537.2	0.62174815	5.71E-59	postive
CPT1B	AC104532.2	0.54489408	5.26E-43	postive
ALOX12	AC104532.2	0.65888125	2.10E-68	postive
PRKAA2	AC124312.2	0.56206406	3.25E-46	postive
PRKAG2	AC103563.7	0.59689537	2.48E-53	postive
ACOT8	AC103563.7	0.52334285	3.13E-39	postive
SDHD	AC103563.7	0.57073832	6.58E-48	postive
PDHB	AC103563.7	0.63813134	5.68E-63	postive
GCDH	AC103563.7	0.51983534	1.21E-38	postive
HACD3	AC103563.7	0.65785625	3.99E-68	postive
AUH	AC103563.7	0.63120199	3.00E-61	postive

HACL1	AC103563.7	0.62405302	1.61E-59	postive
BMPR1B	AC103563.7	0.60209489	1.80E-54	postive
ELOVL4	AC103563.7	0.61297264	6.37E-57	postive
RDH11	AC103563.7	0.60092167	3.27E-54	postive
GABARAPL1	AC103563.7	0.74194516	2.68E-95	postive
ACACB	AC103563.7	0.57355336	1.81E-48	postive
ELOVL2	AC073896.3	0.67432033	9.81E-73	postive
ACSBG2	AC073896.3	0.66393017	8.60E-70	postive
CYP4B1	AC073896.3	0.74278667	1.27E-95	postive
FASN	AC073896.3	0.69942441	2.28E-80	postive
ELOVL2	PPM1F-AS1	0.50254684	7.75E-36	postive
ALOX12	PPM1F-AS1	0.66084158	6.12E-69	postive
ACSBG2	PPM1F-AS1	0.64794219	1.73E-65	postive
CYP4B1	PPM1F-AS1	0.58273009	2.47E-50	postive
ALOX12	AC009318.1	0.61273064	7.24E-57	postive
ACSBG2	AC009318.1	0.56542701	7.27E-47	postive
ACOT6	AC007996.1	0.69392651	1.25E-78	postive
BMPR1B	AC007996.1	0.69757841	8.83E-80	postive
CPT1B	AC104564.3	0.60910982	4.85E-56	postive
D2HGDH	AC104564.3	0.5744937	1.17E-48	postive
ALOX12	AC104564.3	0.71188637	1.84E-84	postive

ALOX12	AL158071.4	0.51789495	2.55E-38	postive
AUH	MPPED2-AS1	0.53463301	3.56E-41	postive
ELOVL4	MPPED2-AS1	0.54677397	2.39E-43	postive
RDH11	MPPED2-AS1	0.53813184	8.61E-42	postive
GABARAPL1	MPPED2-AS1	0.68846202	6.12E-77	postive
ACACB	MPPED2-AS1	0.51360968	1.30E-37	postive
ACOT6	AL445222.1	0.6618788	3.18E-69	postive
CPT1B	AL353708.3	0.51126707	3.12E-37	postive
ELOVL2	AC103719.1	0.74915603	3.99E-98	postive
ACSBG2	AC103719.1	0.651638	1.85E-66	postive
CYP4B1	AC103719.1	0.81680089	2.20E-130	postive
FASN	AC103719.1	0.74415711	3.72E-96	postive
ACOT6	AC114811.2	0.8342771	5.75E-141	postive
BMPR1B	AC114811.2	0.90290936	4.59E-199	postive
RDH11	AC114811.2	0.55181608	2.82E-44	postive
ALOX12	AC016722.2	0.67073193	1.05E-71	postive
ACSBG2	AC016722.2	0.58593102	5.34E-51	postive
ALOX12	AC016590.1	0.58720397	2.89E-51	postive
ACSBG2	AC016590.1	0.55099773	4.00E-44	postive
ELOVL2	AC040977.1	0.52311141	3.42E-39	postive
ALOX12	AC040977.1	0.57340861	1.94E-48	postive
ACSBG2	AC040977.1	0.5781471	2.15E-49	postive

CYP4B1	AC040977.1	0.60613629	2.27E-55	postive
HSD17B3	PDXDC2P-NPIPB14P	0.56491303	9.15E-47	postive
CPT1B	PDXDC2P-NPIPB14P	0.61733119	6.24E-58	postive
D2HGDH	PDXDC2P-NPIPB14P	0.58718531	2.92E-51	postive
ALOX12	PDXDC2P-NPIPB14P	0.6041444	6.32E-55	postive
CPT1B	AP006621.2	0.61318018	5.71E-57	postive
D2HGDH	AP006621.2	0.63146383	2.58E-61	postive
ALOX12	AP006621.2	0.72395677	1.22E-88	postive
ELOVL2	AC025682.1	0.6269158	3.31E-60	postive
ALOX12	AC025682.1	0.50158998	1.10E-35	postive
ACSBG2	AC025682.1	0.67777329	9.70E-74	postive
CYP4B1	AC025682.1	0.68220855	4.74E-75	postive
FASN	AC025682.1	0.62219246	4.48E-59	postive
ALOX12	KCNIP2-AS1	0.57995452	9.18E-50	postive
ALOX12	AC025766.1	0.64607256	5.31E-65	postive
ALOX12	IRF1-AS1	0.58117568	5.16E-50	postive
HSD17B3	AC027601.1	0.54542035	4.22E-43	postive
CPT1B	AC027601.1	0.7102304	6.62E-84	postive
D2HGDH	AC027601.1	0.66615344	2.07E-70	postive
ACADVL	AC027601.1	0.53138007	1.32E-40	postive
ALOX12	AC027601.1	0.60967169	3.62E-56	postive
ACOT6	AC078795.1	0.55031781	5.34E-44	postive

ALOX12	LINC02048	0.54260904	1.36E-42	postive
ELOVL2	AC104971.3	0.50725126	1.39E-36	postive
CYP4B1	AC104971.3	0.55214829	2.44E-44	postive
FASN	AC104971.3	0.51325681	1.48E-37	postive
HSD17B3	AL136295.7	0.55091765	4.13E-44	postive
CPT1B	AL136295.7	0.62182074	5.49E-59	postive
D2HGDH	AL136295.7	0.66953356	2.30E-71	postive
ACADVL	AL136295.7	0.52359429	2.84E-39	postive
ALOX12	AL136295.7	0.59530075	5.48E-53	postive
HSD17B3	AP006545.2	0.52962091	2.65E-40	postive
CPT1B	AP006545.2	0.51577818	5.72E-38	postive
ACOT6	AC025271.3	0.89729628	7.48E-193	postive
BMPR1B	AC025271.3	0.70063027	9.35E-81	postive
ELOVL2	AC005759.1	0.62434754	1.37E-59	postive
ACSBG2	AC005759.1	0.53352537	5.57E-41	postive
CYP4B1	AC005759.1	0.60706425	1.40E-55	postive
FASN	AC005759.1	0.54715162	2.04E-43	postive
ALOX12	Z97055.2	0.61983073	1.62E-58	postive
ACSBG1	AC117500.2	0.55520177	6.56E-45	postive
ALOX12	AC138393.2	0.55459136	8.54E-45	postive
ACSBG2	AC138393.2	0.5858461	5.56E-51	postive

D2HGDH	AL162274.2	0.53042831	1.92E-40	postive
ALOX12	AL162274.2	0.70903636	1.66E-83	postive
ELOVL2	AC015818.2	0.59192614	2.90E-52	postive
ACSBG2	AC015818.2	0.50669154	1.70E-36	postive
CYP4B1	AC015818.2	0.54040408	3.39E-42	postive
FASN	AC015818.2	0.57013128	8.68E-48	postive
CYP4B1	AL359715.3	0.54831466	1.25E-43	postive
ACOT6	AL359715.3	0.63688794	1.17E-62	postive
ELOVL2	AC091212.1	0.57785296	2.46E-49	postive
CYP4B1	AC091212.1	0.61969888	1.74E-58	postive
FASN	AC091212.1	0.64944386	7.01E-66	postive
PRKAA2	AC108449.2	0.53355556	5.50E-41	postive
CBR4	AC108449.2	0.50162805	1.08E-35	postive
ACOT6	NECTIN3-AS1	0.60723241	1.29E-55	postive
BMPR1B	NECTIN3-AS1	0.5913849	3.78E-52	postive
HSD17B3	BACE1-AS	0.52227043	4.74E-39	postive
D2HGDH	BACE1-AS	0.55104426	3.92E-44	postive
HSD17B3	AC092809.4	0.62612724	5.13E-60	postive
CPT1B	UBE2Q1-AS1	0.5852369	7.46E-51	postive
ALOX12	UBE2Q1-AS1	0.70524479	2.97E-82	postive
ALOX12	RRN3P2	0.53724541	1.24E-41	postive

ACOT6	AL137244.1	0.55244583	2.15E-44	postive
PRKAG2	ZNF503-AS2	0.52801226	5.02E-40	postive
ACOT8	ZNF503-AS2	0.61806915	4.20E-58	postive
SDHD	ZNF503-AS2	0.5713239	5.04E-48	postive
PHYH	ZNF503-AS2	0.52299544	3.58E-39	postive
PDHB	ZNF503-AS2	0.50882474	7.75E-37	postive
NDUFAB1	ZNF503-AS2	0.5231433	3.38E-39	postive
HSD17B8	ZNF503-AS2	0.55984887	8.64E-46	postive
GCDH	ZNF503-AS2	0.52366198	2.76E-39	postive
HACD3	ZNF503-AS2	0.59164105	3.34E-52	postive
AUH	ZNF503-AS2	0.53643824	1.72E-41	postive
SUCLG1	ZNF503-AS2	0.52363702	2.79E-39	postive
HACL1	ZNF503-AS2	0.51013011	4.77E-37	postive
ALOX12	AC009095.1	0.58695805	3.26E-51	postive
ACSBG2	AC009095.1	0.64186637	6.42E-64	postive
CYP4B1	AC009095.1	0.52963627	2.64E-40	postive
ELOVL2	AC008663.3	0.89134465	1.20E-186	postive
ACSBG2	AC008663.3	0.63860986	4.30E-63	postive
CYP4B1	AC008663.3	0.91441285	4.51E-213	postive
FASN	AC008663.3	0.86232692	7.79E-161	postive
ALOX12	MAP3K14-AS1	0.64766524	2.05E-65	postive

ELOVL2	WNT5A-AS1	0.57916351	1.33E-49	postive
CYP4B1	WNT5A-AS1	0.58000905	8.95E-50	postive
FASN	WNT5A-AS1	0.62426634	1.44E-59	postive
HSD17B3	AL359921.2	0.5516663	3.00E-44	postive
ALOX12	AL359921.2	0.50175156	1.03E-35	postive
ACSBG2	AL359921.2	0.52896355	3.44E-40	postive
CPT1B	AL356481.3	0.65452521	3.15E-67	postive
D2HGDH	AL356481.3	0.64473095	1.18E-64	postive
ALOX12	AL356481.3	0.57000576	9.19E-48	postive
ALOX12	PKP4-AS1	0.5340669	4.48E-41	postive
ALOX12	AC092436.2	0.51196585	2.41E-37	postive
ACSBG2	AC092436.2	0.54932496	8.14E-44	postive
ALOX12	AL662844.3	0.73826567	6.83E-94	postive
ACSBG2	AL662844.3	0.53698703	1.37E-41	postive
ALOX12	AC141002.1	0.57428965	1.29E-48	postive
HSD17B3	AC135050.3	0.70798964	3.70E-83	postive
CPT1B	AC135050.3	0.55881723	1.36E-45	postive
D2HGDH	AC135050.3	0.60070234	3.65E-54	postive
ALOX12	AC135050.3	0.55420628	1.01E-44	postive
ACSBG2	AC135050.3	0.52055278	9.21E-39	postive
ACSBG1	LINC00954	0.59434392	8.81E-53	postive

ENO3	LINC00954	0.63160901	2.38E-61	postive
ELOVL2	AL354707.1	0.50522921	2.92E-36	postive
CYP4B1	AL354707.1	0.54376084	8.44E-43	postive
FASN	AL354707.1	0.53281147	7.42E-41	postive
ACOT6	BCL2L1-AS1	0.52603157	1.09E-39	postive
ELOVL2	TMEM167B-DT	0.76787582	6.13E-106	postive
ACSBG2	TMEM167B-DT	0.63115454	3.08E-61	postive
CYP4B1	TMEM167B-DT	0.83152876	3.19E-139	postive
FASN	TMEM167B-DT	0.75228383	2.21E-99	postive
CPT1B	AC002553.1	0.54134701	2.30E-42	postive
D2HGDH	AC002553.1	0.50962175	5.77E-37	postive
ALOX12	AC002553.1	0.7036881	9.58E-82	postive
HSD17B3	DM1-AS	0.5383974	7.72E-42	postive
CPT1B	DM1-AS	0.55465697	8.30E-45	postive
D2HGDH	DM1-AS	0.63709158	1.04E-62	postive
ACADVL	DM1-AS	0.59488654	6.73E-53	postive
CPT1B	AC010542.5	0.60865979	6.13E-56	postive
ALOX12	AC010542.5	0.51017781	4.69E-37	postive
ACOT6	AL355916.2	0.53530927	2.71E-41	postive
ACOT6	AP002907.1	0.64015459	1.75E-63	postive
ELOVL2	AC022154.1	0.60658136	1.80E-55	postive
ALOX12	AC022154.1	0.5535182	1.36E-44	postive

ACSBG2	AC022154.1	0.65924425	1.67E-68	postive
CYP4B1	AC022154.1	0.67738668	1.26E-73	postive
FASN	AC022154.1	0.61351747	4.78E-57	postive
PRKAA2	SGMS1-AS1	0.5374252	1.15E-41	postive
CPT1B	AL022328.1	0.60993617	3.15E-56	postive
D2HGDH	AL022328.1	0.67468732	7.69E-73	postive
ALOX12	AL022328.1	0.71472327	2.00E-85	postive
PRKAA2	AC018752.1	0.56396089	1.40E-46	postive
ALOX12	AL033397.2	0.54433245	6.65E-43	postive
ALOX12	AL049552.1	0.55094558	4.09E-44	postive
CPT1B	RNF139-AS1	0.63794066	6.34E-63	postive
D2HGDH	RNF139-AS1	0.50661408	1.75E-36	postive
ALOX12	RNF139-AS1	0.64629971	4.64E-65	postive
ACOT6	PTPRJ-AS1	0.82212212	1.75E-133	postive
BMPR1B	PTPRJ-AS1	0.60007546	5.01E-54	postive
ELOVL4	AL133370.1	0.5708363	6.30E-48	postive
RDH11	AL133370.1	0.50162831	1.08E-35	postive
GABARAPL1	AL133370.1	0.58329883	1.88E-50	postive
D2HGDH	AC135178.2	0.51051466	4.14E-37	postive
ELOVL2	AL391832.2	0.80029681	2.22E-121	postive
ACSBG2	AL391832.2	0.63742992	8.52E-63	postive

CYP4B1	AL391832.2	0.84900111	6.83E-151	postive
FASN	AL391832.2	0.80478737	9.60E-124	postive
ACOT6	AL049840.6	0.57826354	2.03E-49	postive
ELOVL2	AC002059.1	0.53015991	2.14E-40	postive
ALOX12	AC002059.1	0.50780678	1.13E-36	postive
ACSBG2	AC002059.1	0.63102296	3.31E-61	postive
CYP4B1	AC002059.1	0.57602378	5.77E-49	postive
ACOT6	AKT3-IT1	0.5194551	1.41E-38	postive
ALOX12	EDRF1-AS1	0.62842398	1.43E-60	postive
ALOX12	AL049840.2	0.55911422	1.19E-45	postive
ACOT6	AL049840.2	0.58657914	3.91E-51	postive
ACOT6	AC022537.1	0.78335691	5.59E-113	postive
BMPR1B	AC022537.1	0.52558848	1.30E-39	postive
ALOX12	AF111169.3	0.61895067	2.61E-58	postive
ACOT6	AC004837.2	0.83528023	1.30E-141	postive
BMPR1B	AC004837.2	0.64071545	1.26E-63	postive
PRKAG2	C15orf56	0.51589676	5.47E-38	postive
PDHB	C15orf56	0.53342454	5.80E-41	postive
HACD3	C15orf56	0.54693344	2.24E-43	postive
HACL1	C15orf56	0.51586044	5.54E-38	postive
BMPR1B	C15orf56	0.54819215	1.32E-43	postive

ELOVL4	C15orf56	0.62793193	1.88E-60	postive
RDH11	C15orf56	0.53212101	9.79E-41	postive
GABARAPL1	C15orf56	0.62148026	6.61E-59	postive
ACOT6	AC027514.2	0.55521816	6.52E-45	postive
HSD17B3	MHENCR	0.68443813	1.02E-75	postive
CPT1B	MHENCR	0.60067302	3.70E-54	postive
D2HGDH	MHENCR	0.57908066	1.38E-49	postive
ACADVL	MHENCR	0.52356492	2.87E-39	postive
SERINC1	MHENCR	-0.5063117	1.96E-36	negative
PRKAG2	AC069234.1	0.58724048	2.84E-51	postive
PDHB	AC069234.1	0.5545543	8.68E-45	postive
HACD3	AC069234.1	0.54330145	1.02E-42	postive
GABARAPL1	AC069234.1	0.7794001	3.99E-111	postive
ACACB	AC069234.1	0.5870494	3.12E-51	postive
SUCLG1	AL928921.1	0.53750751	1.11E-41	postive
ACSBG1	AL928921.1	0.54852346	1.14E-43	postive
ENO3	AL928921.1	0.92457835	3.33E-227	postive
PCBD1	AL928921.1	0.70380632	8.77E-82	postive
ELOVL2	AC103858.1	0.78329664	5.97E-113	postive
ACSBG2	AC103858.1	0.67666265	2.05E-73	postive
CYP4B1	AC103858.1	0.84618974	6.45E-149	postive

FASN	AC103858.1	0.77810889	1.58E-110	postive
ACSBG2	AGBL5-AS1	0.55034013	5.29E-44	postive
RDH11	B3GAT1-DT	0.52365914	2.77E-39	postive
GABARAPL1	B3GAT1-DT	0.53927328	5.39E-42	postive
ACACB	B3GAT1-DT	0.61039395	2.48E-56	postive
AUH	AC108860.2	0.52597124	1.12E-39	postive
BMPR1B	AC108860.2	0.5848083	9.16E-51	postive
ELOVL4	AC108860.2	0.56583712	6.05E-47	postive
RDH11	AC108860.2	0.55678627	3.30E-45	postive
GABARAPL1	AC108860.2	0.60852763	6.57E-56	postive
ALOX12	AC073487.1	0.59865042	1.03E-53	postive
ACOT6	AC073487.1	0.53012432	2.17E-40	postive
ELOVL2	TTLL1-AS1	0.77340014	2.19E-108	postive
ACSBG2	TTLL1-AS1	0.70310132	1.49E-81	postive
CYP4B1	TTLL1-AS1	0.83038478	1.66E-138	postive
FASN	TTLL1-AS1	0.76997103	7.37E-107	postive
ACADVL	AC007406.2	0.502223	8.72E-36	postive
FAAH	AC007406.2	0.57249983	2.94E-48	postive
ACADL	AC007406.2	0.53952708	4.86E-42	postive
ELOVL2	CCDC28A-AS1	0.7882829	2.42E-115	postive
ACSBG2	CCDC28A-AS1	0.66675538	1.40E-70	postive
CYP4B1	CCDC28A-AS1	0.83731767	6.19E-143	postive

FASN	CCDC28A-AS1	0.75552393	1.05E-100	postive
PRKAG2	THRB-AS1	0.5513131	3.49E-44	postive
SDHD	THRB-AS1	0.55907355	1.21E-45	postive
HACD3	THRB-AS1	0.5779583	2.34E-49	postive
AUH	THRB-AS1	0.57754762	2.84E-49	postive
ACOT6	THRB-AS1	0.66740898	9.18E-71	postive
BMPR1B	THRB-AS1	0.88604622	2.03E-181	postive
ELOVL4	THRB-AS1	0.51945505	1.41E-38	postive
RDH11	THRB-AS1	0.64857327	1.19E-65	postive
GABARAPL1	THRB-AS1	0.67524985	5.28E-73	postive
ACACB	THRB-AS1	0.58954582	9.29E-52	postive
CPT1B	AC138028.4	0.55181973	2.81E-44	postive
D2HGDH	AC138028.4	0.59401425	1.04E-52	postive
ALOX12	AC138028.4	0.66463206	5.49E-70	postive
ALOX12	AC007663.4	0.56255468	2.62E-46	postive
ACOT6	AC007663.4	0.52189205	5.49E-39	postive
ENO3	TP73-AS3	0.58614096	4.83E-51	postive
ALOX12	H3-3A-DT	0.56018503	7.46E-46	postive
ACOT6	AC010168.1	0.65290513	8.53E-67	postive
BMPR1B	AC010168.1	0.55029765	5.38E-44	postive
HSD17B3	LINC00342	0.60108732	3.00E-54	postive

CPT1B	LINC00342	0.66249358	2.15E-69	postive
D2HGDH	LINC00342	0.63435303	5.00E-62	postive
ALOX12	LINC00342	0.63275037	1.25E-61	postive
CPT1B	AC110285.2	0.5515999	3.09E-44	postive
D2HGDH	AC110285.2	0.64531014	8.37E-65	postive
SDHD	OTUD6B-AS1	0.52634204	9.69E-40	postive
AUH	OTUD6B-AS1	0.56137332	4.41E-46	postive
PRKAA2	OTUD6B-AS1	0.5511215	3.79E-44	postive
CBR4	OTUD6B-AS1	0.57598799	5.87E-49	postive
ETFDH	OTUD6B-AS1	0.52977886	2.49E-40	postive
MMUT	OTUD6B-AS1	0.52174391	5.82E-39	postive
ACOT6	AC004223.2	0.51024428	4.57E-37	postive
CPT1B	ZNF775-AS1	0.50730973	1.36E-36	postive
ACACB	ZNF775-AS1	0.57101441	5.80E-48	postive
HSD17B3	AC007220.1	0.55406537	1.07E-44	postive
ALOX12	AC127024.5	0.66317599	1.39E-69	postive
ACOT6	AL121655.1	0.5071909	1.42E-36	postive
ALOX12	AC016727.1	0.54067448	3.03E-42	postive
HSD17B3	AL078604.2	0.56485566	9.39E-47	postive
ALOX12	TMEM30A-DT	0.63885618	3.73E-63	postive
ELOVL2	BRWD1-AS1	0.83069436	1.07E-138	postive
ACSBG2	BRWD1-AS1	0.6587979	2.21E-68	postive

CYP4B1	BRWD1-AS1	0.89229077	1.31E-187	postive
FASN	BRWD1-AS1	0.82785952	6.09E-137	postive
ELOVL2	AC106870.1	0.83601073	4.39E-142	postive
ACSBG2	AC106870.1	0.64239074	4.72E-64	postive
CYP4B1	AC106870.1	0.89721439	9.16E-193	postive
FASN	AC106870.1	0.83495011	2.13E-141	postive
ELOVL2	AL355001.1	0.78707128	9.36E-115	postive
ACSBG2	AL355001.1	0.70502272	3.51E-82	postive
CYP4B1	AL355001.1	0.84878749	9.69E-151	postive
FASN	AL355001.1	0.77546699	2.55E-109	postive
PRKAA2	AC004112.1	0.63775487	7.06E-63	postive
CBR4	AC004112.1	0.5417389	1.95E-42	postive
MMAA	AC004112.1	0.55701415	2.99E-45	postive
ELOVL2	AC099778.1	0.59070637	5.27E-52	postive
ALOX12	AC099778.1	0.51281239	1.75E-37	postive
ACSBG2	AC099778.1	0.63968351	2.30E-63	postive
CYP4B1	AC099778.1	0.66443675	6.22E-70	postive
FASN	AC099778.1	0.61304156	6.15E-57	postive
PRKAG2	AC092142.1	0.52433488	2.13E-39	postive
ALOX12	AC023830.3	0.54933878	8.09E-44	postive
ACSBG1	AC007785.1	0.79309905	1.03E-117	postive

ENO3	AC007785.1	0.71287576	8.51E-85	postive
PCBD1	AC007785.1	0.64278497	3.74E-64	postive
ALOX12	AC112722.1	0.55971488	9.17E-46	postive
ELOVL2	PRR7-AS1	0.59638718	3.19E-53	postive
ACSBG2	PRR7-AS1	0.65794753	3.77E-68	postive
CYP4B1	PRR7-AS1	0.64020515	1.70E-63	postive
FASN	PRR7-AS1	0.59530975	5.46E-53	postive
ACOT6	AC099811.4	0.61919107	2.29E-58	postive
ALOX12	AP001893.3	0.50733605	1.34E-36	postive
ALOX12	AP001469.2	0.66066454	6.85E-69	postive
CPT1B	AL022322.1	0.6208452	9.34E-59	postive
D2HGDH	AL022322.1	0.65128742	2.29E-66	postive
ALOX12	AL022322.1	0.69916473	2.76E-80	postive
CPT1B	CAPN10-DT	0.78951868	6.05E-116	postive
D2HGDH	CAPN10-DT	0.60012727	4.88E-54	postive
ALOX12	CAPN10-DT	0.57789735	2.41E-49	postive
ELOVL2	AC245884.9	0.72099269	1.36E-87	postive
ACSBG2	AC245884.9	0.73321718	5.32E-92	postive
CYP4B1	AC245884.9	0.6856928	4.26E-76	postive
FASN	AC245884.9	0.66328242	1.30E-69	postive
ELOVL2	AC005342.2	0.5013733	1.19E-35	postive
ACSBG2	AC005342.2	0.5533556	1.46E-44	postive

CYP4B1	AC005342.2	0.56133916	4.48E-46	postive
FASN	AC005342.2	0.51841778	2.09E-38	postive
CPT1B	AC092118.2	0.57815163	2.14E-49	postive
D2HGDH	AC092118.2	0.64230472	4.96E-64	postive
ACADVL	AC092118.2	0.50895889	7.38E-37	postive
ELOVL2	AC009962.1	0.53477766	3.36E-41	postive
CYP4B1	AC009962.1	0.55240572	2.19E-44	postive
ELOVL2	AP003419.3	0.52290109	3.71E-39	postive
ACSBG2	AP003419.3	0.61423356	3.27E-57	postive
CYP4B1	AP003419.3	0.57066458	6.81E-48	postive
FASN	AP003419.3	0.51108251	3.35E-37	postive
ELOVL2	AP003170.3	0.71119421	3.14E-84	postive
ACSBG2	AP003170.3	0.65549736	1.73E-67	postive
CYP4B1	AP003170.3	0.76478921	1.34E-104	postive
FASN	AP003170.3	0.69055113	1.40E-77	postive
ACOT6	ZBTB20-AS1	0.69052346	1.43E-77	postive
CPT1B	AC138932.4	0.51364669	1.28E-37	postive
ALOX12	AC138932.4	0.58465225	9.87E-51	postive
HSD17B3	PTOV1-AS2	0.53974101	4.45E-42	postive
CPT1B	PTOV1-AS2	0.76686909	1.68E-105	postive

D2HGDH	PTOV1-AS2	0.72454924	7.50E-89	postive
ACADVL	PTOV1-AS2	0.6019503	1.94E-54	postive
ALOX12	PTOV1-AS2	0.58261558	2.61E-50	postive
ELOVL2	RBM15-AS1	0.78387981	3.16E-113	postive
ACSBG2	RBM15-AS1	0.68354581	1.89E-75	postive
CYP4B1	RBM15-AS1	0.83138647	3.92E-139	postive
FASN	RBM15-AS1	0.79703963	1.06E-119	postive
ELOVL2	AC005096.1	0.66796879	6.38E-71	postive
ACSBG2	AC005096.1	0.68489351	7.43E-76	postive
CYP4B1	AC005096.1	0.72309872	2.46E-88	postive
FASN	AC005096.1	0.65071018	3.25E-66	postive
ELOVL2	AC005277.2	0.82146838	4.27E-133	postive
ACSBG2	AC005277.2	0.65550119	1.72E-67	postive
CYP4B1	AC005277.2	0.8682399	1.38E-165	postive
FASN	AC005277.2	0.80772396	2.53E-125	postive
ELOVL2	AC021755.2	0.77690376	5.63E-110	postive
ACSBG2	AC021755.2	0.61943516	2.01E-58	postive
CYP4B1	AC021755.2	0.84122957	1.58E-145	postive
FASN	AC021755.2	0.776448	9.10E-110	postive
ALOX12	AC011466.1	0.57783931	2.48E-49	postive
ELOVL2	AC026803.1	0.51488666	8.01E-38	postive
ACSBG2	AC026803.1	0.60538636	3.34E-55	postive

CYP4B1	AC026803.1	0.54932374	8.15E-44	postive
FASN	AC026803.1	0.50866056	8.24E-37	postive
DPEP2	TRG-AS1	0.53308091	6.66E-41	postive
DPEP2	AC026369.3	0.5045823	3.69E-36	postive
ALOX12	AC005899.7	0.52080045	8.38E-39	postive
ELOVL2	AC007036.2	0.5881009	1.87E-51	postive
ACSBG2	AC007036.2	0.61584458	1.38E-57	postive
CYP4B1	AC007036.2	0.65950464	1.42E-68	postive
FASN	AC007036.2	0.56543825	7.23E-47	postive
ELOVL2	AC091180.4	0.82221847	1.54E-133	postive
ACSBG2	AC091180.4	0.68306589	2.63E-75	postive
CYP4B1	AC091180.4	0.87257466	3.21E-169	postive
FASN	AC091180.4	0.82126174	5.65E-133	postive
ALOX12	AC022400.4	0.72085173	1.52E-87	postive
ACSBG2	AC022400.4	0.61659382	9.27E-58	postive
CYP4B1	AC022400.4	0.50100716	1.35E-35	postive
ACOT6	AC016737.2	0.67681929	1.84E-73	postive
PDHB	AC022007.1	0.50766215	1.19E-36	postive
GABARAPL1	AC022007.1	0.55563624	5.44E-45	postive
HSD17B3	AL021707.6	0.5507879	4.37E-44	postive
CPT1B	AL021707.6	0.61785022	4.72E-58	postive
D2HGDH	AL021707.6	0.62260006	3.58E-59	postive

ELOVL2	AC079210.1	0.60611701	2.29E-55	postive
ACSBG2	AC079210.1	0.55846112	1.59E-45	postive
CYP4B1	AC079210.1	0.61202234	1.05E-56	postive
FASN	AC079210.1	0.58674346	3.61E-51	postive
ACAA2	EMX2OS	0.6552303	2.04E-67	postive
EHHADH	EMX2OS	0.52167567	5.97E-39	postive
ALDH3A2	EMX2OS	0.65354615	5.75E-67	postive
HIBCH	EMX2OS	0.50423842	4.19E-36	postive
SLC27A2	EMX2OS	0.56436539	1.17E-46	postive
ALOX12	LINC02656	0.58683515	3.46E-51	postive
PRKAG2	AC103563.2	0.50363316	5.22E-36	postive
ACOT6	AC103563.2	0.60425385	5.98E-55	postive
BMPR1B	AC103563.2	0.74371536	5.53E-96	postive
RDH11	AC103563.2	0.54443809	6.36E-43	postive
GABARAPL1	AC103563.2	0.6131523	5.80E-57	postive
ACACB	AC103563.2	0.57946699	1.15E-49	postive
PRKAG2	SBF2-AS1	0.60488667	4.32E-55	postive
AUH	SBF2-AS1	0.53179056	1.12E-40	postive
GABARAPL1	SBF2-AS1	0.54023509	3.63E-42	postive
SUCLG1	AL139275.2	0.55266819	1.96E-44	postive
PCBD1	AL139275.2	0.57832504	1.97E-49	postive

D2HGDH	NDUFA6-DT	0.52027261	1.03E-38	postive
ALOX12	NDUFA6-DT	0.72431351	9.10E-89	postive
ACSBG2	NDUFA6-DT	0.54586437	3.50E-43	postive
ALOX12	AP005899.1	0.67011058	1.58E-71	postive
ELOVL2	AC145207.5	0.51293847	1.67E-37	postive
ACSBG2	AC145207.5	0.54728805	1.93E-43	postive
CYP4B1	AC145207.5	0.5484862	1.16E-43	postive
FASN	AC145207.5	0.53406269	4.49E-41	postive
ALOX12	AP002490.1	0.68205502	5.27E-75	postive
ACSBG2	AP002490.1	0.5737344	1.67E-48	postive
HSD17B3	AC104365.2	0.63178899	2.15E-61	postive
CPT1B	AL031186.1	0.65977717	1.20E-68	postive
D2HGDH	AL031186.1	0.51144863	2.92E-37	postive
ELOVL2	AC008429.1	0.75468312	2.33E-100	postive
ACSBG2	AC008429.1	0.66342361	1.19E-69	postive
CYP4B1	AC008429.1	0.80089718	1.08E-121	postive
FASN	AC008429.1	0.7696394	1.03E-106	postive
ELOVL2	AL161909.2	0.60819312	7.82E-56	postive
ACSBG2	AL161909.2	0.650184	4.48E-66	postive
CYP4B1	AL161909.2	0.66905534	3.15E-71	postive
FASN	AL161909.2	0.58872384	1.39E-51	postive
HSD17B3	AC245884.8	0.66253482	2.09E-69	postive
CPT1B	AC245884.8	0.5548768	7.55E-45	postive

D2HGDH	AC245884.8	0.51295034	1.66E-37	postive
ALOX12	AC245884.8	0.56864554	1.70E-47	postive
ACSBG2	AC245884.8	0.52761368	5.87E-40	postive
ALOX12	AC008770.3	0.5469297	2.24E-43	postive
ACOT6	AC008770.3	0.5456484	3.84E-43	postive
CBR4	AC008770.3	0.52017433	1.07E-38	postive
ALOX12	AC012409.1	0.50296632	6.66E-36	postive
ALOX12	AL034550.1	0.55890587	1.31E-45	postive
HSD17B3	AL390728.5	0.5909041	4.79E-52	postive
CPT1B	AL390728.5	0.52547794	1.36E-39	postive
ALOX12	AL353801.3	0.59996358	5.30E-54	postive
HSD17B3	AL356652.1	0.5311935	1.42E-40	postive
ACOT6	MAILR	0.53841883	7.65E-42	postive
BMPR1B	MAILR	0.53368034	5.23E-41	postive
ENO3	RHOXF1-AS1	0.52225231	4.78E-39	postive
ELOVL2	AC008127.1	0.8324979	7.81E-140	postive
ACSBG2	AC008127.1	0.63714042	1.01E-62	postive
CYP4B1	AC008127.1	0.8960647	1.54E-191	postive
FASN	AC008127.1	0.83177424	2.24E-139	postive
ACOT6	AC005070.3	0.56267716	2.48E-46	postive
ELOVL2	AL031848.1	0.59151762	3.55E-52	postive
ALOX12	AL031848.1	0.500117	1.87E-35	postive
ACSBG2	AL031848.1	0.57994666	9.22E-50	postive
CYP4B1	AL031848.1	0.70343276	1.16E-81	postive

FASN	AL031848.1	0.55328517	1.50E-44	postive
ALOX12	NARF-IT1	0.70971018	9.88E-84	postive
CPT1B	AC006064.3	0.50875638	7.95E-37	postive
D2HGDH	AC006064.3	0.58629414	4.49E-51	postive
ALOX12	AC006064.3	0.68596638	3.52E-76	postive
ELOVL2	AC124242.1	0.67824444	7.06E-74	postive
ACSBG2	AC124242.1	0.6135977	4.58E-57	postive
CYP4B1	AC124242.1	0.75832052	7.33E-102	postive
FASN	AC124242.1	0.64248704	4.46E-64	postive
ALOX12	AC090607.1	0.60446029	5.37E-55	postive
ACOT6	AL359220.1	0.77997838	2.15E-111	postive
BMPR1B	AL359220.1	0.63856309	4.42E-63	postive
CPT1B	AC024075.2	0.56260316	2.56E-46	postive
ELOVL2	AC022211.1	0.57227724	3.26E-48	postive
ALOX12	AC022211.1	0.59287198	1.82E-52	postive
ACSBG2	AC022211.1	0.67112856	8.10E-72	postive
CYP4B1	AC022211.1	0.59712087	2.21E-53	postive
FASN	AC022211.1	0.58554618	6.43E-51	postive
ELOVL2	AC116914.2	0.61315027	5.80E-57	postive
ALOX12	AC116914.2	0.59028964	6.47E-52	postive
ACSBG2	AC116914.2	0.65660794	8.68E-68	postive

CYP4B1	AC116914.2	0.65207535	1.42E-66	postive
FASN	AC116914.2	0.61895396	2.60E-58	postive
ACSBG2	AC009054.1	0.54613983	3.12E-43	postive
ELOVL2	AC079384.1	0.83075398	9.78E-139	postive
ACSBG2	AC079384.1	0.65738536	5.35E-68	postive
CYP4B1	AC079384.1	0.89158077	6.92E-187	postive
FASN	AC079384.1	0.82537457	1.99E-135	postive
GCDH	PCCA-DT	0.50920132	6.74E-37	postive
ACOT6	AC004241.3	0.52371929	2.70E-39	postive
ACOT6	LINC02115	0.52051936	9.33E-39	postive
BMPR1B	LINC02115	0.74164268	3.50E-95	postive
RDH11	LINC02115	0.6536916	5.26E-67	postive
ELOVL2	AL136295.2	0.53488995	3.21E-41	postive
HSD17B3	AL136295.2	0.57880354	1.58E-49	postive
D2HGDH	AL136295.2	0.50078388	1.47E-35	postive
ALOX12	AL136295.2	0.5467095	2.46E-43	postive
ACSBG2	AL136295.2	0.63815341	5.61E-63	postive
CYP4B1	AL136295.2	0.56844844	1.86E-47	postive
FASN	AL136295.2	0.50332841	5.84E-36	postive
ACOT6	ITCH-IT1	0.58407418	1.30E-50	postive
ALOX12	MKNK1-AS1	0.65381446	4.88E-67	postive

ELOVL2	AL136418.1	0.72020829	2.55E-87	postive
ACSBG2	AL136418.1	0.67679411	1.88E-73	postive
CYP4B1	AL136418.1	0.76917199	1.66E-106	postive
FASN	AL136418.1	0.70653636	1.12E-82	postive
ELOVL2	LINC02481	0.71151208	2.46E-84	postive
ACSBG2	LINC02481	0.67793341	8.71E-74	postive
CYP4B1	LINC02481	0.77792414	1.92E-110	postive
FASN	LINC02481	0.71657125	4.66E-86	postive
CPT1B	AL021707.3	0.64778297	1.91E-65	postive
D2HGDH	AL021707.3	0.57675116	4.12E-49	postive
ALOX12	AL021707.3	0.62669198	3.75E-60	postive
ALOX12	AC025627.1	0.67287419	2.56E-72	postive
ACOT6	HOXB-AS2	0.85509016	2.60E-155	postive
BMPR1B	HOXB-AS2	0.72520429	4.38E-89	postive
ALOX12	OBSCN-AS1	0.53015745	2.14E-40	postive
ACSBG2	OBSCN-AS1	0.51127921	3.11E-37	postive
ELOVL2	AL512408.1	0.52638939	9.51E-40	postive
CYP4B1	AL512408.1	0.53844545	7.57E-42	postive
FASN	AL512408.1	0.56476308	9.78E-47	postive
ACSBG2	AC002558.3	0.54294112	1.19E-42	postive

ELOVL2	AC024592.1	0.81000641	1.43E-126	postive
ACSBG2	AC024592.1	0.69967379	1.90E-80	postive
CYP4B1	AC024592.1	0.87480929	3.82E-171	postive
FASN	AC024592.1	0.80223815	2.15E-122	postive
ELOVL2	AC018761.1	0.83719486	7.45E-143	postive
ACSBG2	AC018761.1	0.66115014	5.04E-69	postive
CYP4B1	AC018761.1	0.8948353	3.05E-190	postive
FASN	AC018761.1	0.83115469	5.49E-139	postive
ALOX12	LINC01359	0.53900845	6.01E-42	postive
ACOT6	LINC01359	0.51685198	3.80E-38	postive
HSD17B3	AC010809.2	0.58039396	7.46E-50	postive
CPT1B	AC010809.2	0.56624205	5.04E-47	postive
ELOVL2	SUCLA2-AS1	0.54023149	3.64E-42	postive
ACSBG2	SUCLA2-AS1	0.51770038	2.75E-38	postive
CYP4B1	SUCLA2-AS1	0.59035637	6.26E-52	postive
FASN	SUCLA2-AS1	0.54137399	2.27E-42	postive
ACOT6	AC069257.1	0.57470975	1.06E-48	postive
ELOVL2	AC020913.3	0.74488323	1.94E-96	postive
ACSBG2	AC020913.3	0.70191853	3.59E-81	postive
CYP4B1	AC020913.3	0.81307649	2.84E-128	postive
FASN	AC020913.3	0.74070649	8.02E-95	postive

ACSBG1	CCDC144NL-AS1	0.56255962	2.61E-46	postive
RDH11	GTSE1-DT	0.50398921	4.59E-36	postive
GABARAPL1	GTSE1-DT	0.58523488	7.46E-51	postive
ALOX12	AL645941.1	0.59186604	2.99E-52	postive
TBXAS1	LINC01094	0.71149178	2.50E-84	postive
ACOT6	Z99289.2	0.59180442	3.08E-52	postive
ELOVL2	LINC00471	0.79796754	3.54E-120	postive
ACSBG2	LINC00471	0.65645724	9.53E-68	postive
CYP4B1	LINC00471	0.85825906	1.08E-157	postive
FASN	LINC00471	0.79842726	2.05E-120	postive
ACOT6	AC072022.1	0.61535901	1.79E-57	postive
CPT1B	LINC01160	0.59975709	5.89E-54	postive
D2HGDH	LINC01160	0.52800638	5.03E-40	postive
ALOX12	AC010320.3	0.56385889	1.46E-46	postive
ACSBG2	AC087742.1	0.51414964	1.06E-37	postive
ELOVL2	AC106738.1	0.67213022	4.19E-72	postive
ACSBG2	AC106738.1	0.56588069	5.93E-47	postive
CYP4B1	AC106738.1	0.71695252	3.45E-86	postive
FASN	AC106738.1	0.66890352	3.48E-71	postive
ACSBG2	AL157932.1	0.51518058	7.17E-38	postive

CYP4B1	AL157932.1	0.55405175	1.08E-44	postive
ACSBG2	AL807752.5	0.60589705	2.57E-55	postive
CYP4B1	AC015908.3	0.53931861	5.29E-42	postive
ALOX12	AC010260.1	0.52867639	3.86E-40	postive
ACSBG2	AC010260.1	0.56122767	4.71E-46	postive
CPT1B	AL512652.1	0.50609646	2.12E-36	postive
ALOX12	AL512652.1	0.52836813	4.36E-40	postive
SDHD	DPP10-AS1	0.51031833	4.45E-37	postive
PHYH	DPP10-AS1	0.52290313	3.71E-39	postive
HACD3	DPP10-AS1	0.5610685	5.05E-46	postive
ELOVL4	DPP10-AS1	0.59961573	6.32E-54	postive
RDH11	DPP10-AS1	0.55328971	1.50E-44	postive
D2HGDH	WDR5-DT	0.50776492	1.15E-36	postive
ACOT6	AC008467.1	0.58177641	3.88E-50	postive
ALOX12	OSMR-AS1	0.54384833	8.14E-43	postive
ELOVL2	AC037198.2	0.75782167	1.18E-101	postive
ACSBG2	AC037198.2	0.54298109	1.17E-42	postive
CYP4B1	AC037198.2	0.73883845	4.14E-94	postive
FASN	AC037198.2	0.71970149	3.84E-87	postive
ALOX12	AC007390.1	0.63379436	6.88E-62	postive
ACSBG2	AC007390.1	0.64695576	3.13E-65	postive

ENO3	AC010907.2	0.82791676	5.62E-137	postive
HSD17B3	LMNTD2-AS1	0.55364487	1.28E-44	postive
CPT1B	LMNTD2-AS1	0.54787534	1.50E-43	postive
D2HGDH	LMNTD2-AS1	0.62581278	6.11E-60	postive
ACADVL	LMNTD2-AS1	0.50787092	1.10E-36	postive
ELOVL2	AC145285.3	0.8001099	2.78E-121	postive
ACSBG2	AC145285.3	0.71042718	5.69E-84	postive
CYP4B1	AC145285.3	0.85497428	3.17E-155	postive
FASN	AC145285.3	0.79761827	5.34E-120	postive
ELOVL2	AC016876.3	0.68355748	1.87E-75	postive
ACSBG2	AC016876.3	0.61768891	5.15E-58	postive
CYP4B1	AC016876.3	0.74127353	4.86E-95	postive
FASN	AC016876.3	0.69823483	5.46E-80	postive
CPT1B	AC073575.2	0.59732552	2.00E-53	postive
D2HGDH	AC073575.2	0.52157463	6.21E-39	postive
ALOX12	AC073575.2	0.55801124	1.93E-45	postive
CPT1B	AC090772.3	0.51882022	1.79E-38	postive
ALOX12	AC015727.1	0.62259584	3.59E-59	postive
HSD17B3	HCG27	0.52594641	1.13E-39	postive
CPT1B	HCG27	0.56101186	5.18E-46	postive
D2HGDH	HCG27	0.60360661	8.32E-55	postive

ALOX12	HCG27	0.70557231	2.32E-82	postive
HSD17B3	CAHM	0.55632989	4.02E-45	postive
D2HGDH	CAHM	0.5301698	2.13E-40	postive
ELOVL2	AC136475.5	0.65400347	4.34E-67	postive
ACSBG2	AC136475.5	0.6093506	4.28E-56	postive
CYP4B1	AC136475.5	0.7414043	4.33E-95	postive
FASN	AC136475.5	0.63533182	2.85E-62	postive
ELOVL2	AL360091.2	0.57885635	1.54E-49	postive
ACSBG2	AL360091.2	0.55965395	9.42E-46	postive
CYP4B1	AL360091.2	0.63760319	7.71E-63	postive
ACOT6	AL360091.2	0.64581986	6.18E-65	postive
FASN	AL360091.2	0.55421983	1.00E-44	postive
ACOT6	AC010210.1	0.77075577	3.31E-107	postive
BMPR1B	AC010210.1	0.62087722	9.18E-59	postive
ENO3	AC091729.2	0.76690141	1.63E-105	postive
PCBD1	AC091729.2	0.60230422	1.62E-54	postive
ELOVL2	LINC00336	0.68167863	6.82E-75	postive
ACSBG2	LINC00336	0.62330402	2.44E-59	postive
CYP4B1	LINC00336	0.73910502	3.28E-94	postive
FASN	LINC00336	0.68937232	3.22E-77	postive
ACOT6	ZBTB20-AS4	0.72084397	1.53E-87	postive
BMPR1B	ZBTB20-AS4	0.51874745	1.84E-38	postive

ACOT6	C1QTNF7-AS1	0.77940631	3.96E-111	postive
BMPR1B	C1QTNF7-AS1	0.50864506	8.29E-37	postive
PHYH	AC120498.4	0.50100131	1.36E-35	postive
HACL1	AC120498.4	0.51290642	1.69E-37	postive
BMPR1B	AC120498.4	0.63094041	3.47E-61	postive
ELOVL4	AC120498.4	0.63839829	4.86E-63	postive
RDH11	AC120498.4	0.78609662	2.76E-114	postive
ACACB	AC120498.4	0.52760404	5.90E-40	postive
ACOT6	AC005550.1	0.78228203	1.80E-112	postive
BMPR1B	AC005550.1	0.88808632	2.11E-183	postive
RDH11	AC005550.1	0.5467386	2.43E-43	postive
ACOT6	LINC01091	0.65349897	5.92E-67	postive
BMPR1B	LINC01091	0.63621481	1.72E-62	postive
ELOVL2	AC006270.1	0.59948968	6.74E-54	postive
ACSBG2	AC006270.1	0.6192971	2.16E-58	postive
CYP4B1	AC006270.1	0.65438131	3.44E-67	postive
FASN	AC006270.1	0.58128021	4.91E-50	postive
ELOVL2	AC104463.2	0.56773661	2.57E-47	postive
ACSBG2	AC104463.2	0.63167853	2.29E-61	postive
CYP4B1	AC104463.2	0.60525654	3.57E-55	postive
FASN	AC104463.2	0.52981769	2.45E-40	postive

ELOVL2	AC005014.2	0.68755502	1.16E-76	postive
ACSBG2	AC005014.2	0.64086501	1.16E-63	postive
CYP4B1	AC005014.2	0.74542546	1.19E-96	postive
FASN	AC005014.2	0.67805679	8.01E-74	postive
ALOX12	MCM3AP-AS1	0.70731703	6.17E-83	postive
ACSBG2	MCM3AP-AS1	0.54358809	9.07E-43	postive
ELOVL2	GK-IT1	0.81277026	4.21E-128	postive
ACSBG2	GK-IT1	0.68702296	1.68E-76	postive
CYP4B1	GK-IT1	0.87111819	5.52E-168	postive
FASN	GK-IT1	0.80535166	4.80E-124	postive
ALOX12	AC020978.7	0.58604511	5.06E-51	postive
ACSBG2	AC020978.7	0.56373546	1.55E-46	postive
ALOX12	AC004494.1	0.685212	5.95E-76	postive
ELOVL2	AC011921.1	0.51545074	6.47E-38	postive
ALOX12	AC011921.1	0.52716383	7.01E-40	postive
ACSBG2	AC011921.1	0.59422572	9.34E-53	postive
CYP4B1	AC011921.1	0.55107075	3.87E-44	postive
FASN	AC011921.1	0.57240075	3.08E-48	postive
ELOVL2	MIR3945HG	0.8404634	5.16E-145	postive
ACSBG2	MIR3945HG	0.63553678	2.54E-62	postive
CYP4B1	MIR3945HG	0.88685328	3.37E-182	postive

FASN	MIR3945HG	0.82944564	6.39E-138	postive
HSD17B3	LINC01138	0.58306676	2.10E-50	postive
ELOVL2	LINC02255	0.84049403	4.92E-145	postive
ACSBG2	LINC02255	0.67524475	5.30E-73	postive
CYP4B1	LINC02255	0.89397642	2.40E-189	postive
FASN	LINC02255	0.83311038	3.19E-140	postive
ACSBG1	AL031123.2	0.62293814	2.98E-59	postive
ENO3	AL031123.2	0.65915785	1.77E-68	postive
PCBD1	AL031123.2	0.5678564	2.44E-47	postive
ELOVL2	Z97353.2	0.82628557	5.58E-136	postive
ACSBG2	Z97353.2	0.66375242	9.64E-70	postive
CYP4B1	Z97353.2	0.89034498	1.22E-185	postive
FASN	Z97353.2	0.82061507	1.36E-132	postive
ACOT6	AL359710.1	0.79661838	1.73E-119	postive
BMPR1B	AL359710.1	0.56928542	1.28E-47	postive
ELOVL2	AC007613.1	0.51506908	7.48E-38	postive
ACSBG2	AC007613.1	0.53053156	1.85E-40	postive
CYP4B1	AC007613.1	0.59095971	4.66E-52	postive
FASN	AC007613.1	0.56040075	6.78E-46	postive
ELOVL2	AC005529.1	0.83511792	1.66E-141	postive
ACSBG2	AC005529.1	0.69881961	3.56E-80	postive

CYP4B1	AC005529.1	0.89221447	1.57E-187	postive
FASN	AC005529.1	0.82765998	8.07E-137	postive
PRKAG2	AC011523.1	0.54364492	8.86E-43	postive
SDHD	AC011523.1	0.55611511	4.42E-45	postive
PHYH	AC011523.1	0.55754043	2.38E-45	postive
HACD3	AC011523.1	0.58993621	7.68E-52	postive
AUH	AC011523.1	0.56664518	4.21E-47	postive
HACL1	AC011523.1	0.50934869	6.38E-37	postive
BMPR1B	AC011523.1	0.68486493	7.57E-76	postive
ELOVL4	AC011523.1	0.7369123	2.22E-93	postive
RDH11	AC011523.1	0.78867343	1.56E-115	postive
GABARAPL1	AC011523.1	0.62037927	1.20E-58	postive
ACACB	AC011523.1	0.60606155	2.36E-55	postive
ALOX12	LINC00677	0.61966124	1.78E-58	postive
ELOVL2	AL021707.7	0.77013524	6.23E-107	postive
ACSBG2	AL021707.7	0.71628553	5.85E-86	postive
CYP4B1	AL021707.7	0.83492422	2.21E-141	postive
FASN	AL021707.7	0.75441942	2.99E-100	postive
CPT1B	CCDC18-AS1	0.63439673	4.87E-62	postive
D2HGDH	CCDC18-AS1	0.5769416	3.77E-49	postive
ALOX12	CCDC18-AS1	0.70339197	1.20E-81	postive

CPT1B	SH3BP5-AS1	0.56881176	1.58E-47	postive
D2HGDH	SH3BP5-AS1	0.58208554	3.35E-50	postive
ALOX12	SH3BP5-AS1	0.66277153	1.80E-69	postive
CPT1B	AC011498.6	0.53835173	7.87E-42	postive
ALOX12	AC011498.6	0.54911177	8.91E-44	postive
SERINC1	AL731577.2	0.53665211	1.57E-41	postive
ALOX12	RBFADN	0.60145822	2.49E-54	postive
ALOX12	AC090510.2	0.64623569	4.82E-65	postive
ACSBG2	AC090510.2	0.54206829	1.70E-42	postive
CPT1B	LINC00894	0.51149442	2.87E-37	postive
ALOX12	LINC00894	0.5880866	1.89E-51	postive
ELOVL2	LINC01842	0.70219422	2.93E-81	postive
CYP4B1	LINC01842	0.65315295	7.32E-67	postive
FASN	LINC01842	0.70924881	1.41E-83	postive
ELOVL2	AD001527.1	0.80675873	8.42E-125	postive
ACSBG2	AD001527.1	0.69436743	9.10E-79	postive
CYP4B1	AD001527.1	0.87120276	4.69E-168	postive
FASN	AD001527.1	0.79886975	1.22E-120	postive
PRKAG2	NNT-AS1	0.5725811	2.83E-48	postive
SDHD	NNT-AS1	0.59717513	2.15E-53	postive
PDHB	NNT-AS1	0.522844	3.80E-39	postive
HACD3	NNT-AS1	0.58096584	5.70E-50	postive

AUH	NNT-AS1	0.605517	3.12E-55	postive
HACL1	NNT-AS1	0.51619528	4.88E-38	postive
CBR4	NNT-AS1	0.53569094	2.32E-41	postive
RDH11	NNT-AS1	0.52134078	6.80E-39	postive
GABARAPL1	NNT-AS1	0.5941302	9.79E-53	postive
ACACB	NNT-AS1	0.55227878	2.31E-44	postive
ELOVL2	AP001107.9	0.56911223	1.38E-47	postive
CYP4B1	AP001107.9	0.57029039	8.07E-48	postive
FASN	AP001107.9	0.64678776	3.46E-65	postive
ELOVL2	HMGA1P4	0.79398097	3.72E-118	postive
ACSBG2	HMGA1P4	0.69221473	4.27E-78	postive
CYP4B1	HMGA1P4	0.85010743	1.11E-151	postive
FASN	HMGA1P4	0.79195044	3.83E-117	postive
HSD17B3	AL133410.1	0.58343393	1.77E-50	postive
CPT1B	AL133410.1	0.61948919	1.95E-58	postive
D2HGDH	AL133410.1	0.5396583	4.60E-42	postive
ALOX12	AL133410.1	0.53863235	7.01E-42	postive
CPT1B	AC254562.3	0.54896081	9.50E-44	postive
D2HGDH	AC254562.3	0.67704179	1.59E-73	postive
ACADVL	AC254562.3	0.53501336	3.06E-41	postive
ALOX12	AC254562.3	0.56284414	2.30E-46	postive

ELOVL2	AC105339.2	0.61486268	2.34E-57	postive
ALOX12	AC105339.2	0.57949891	1.14E-49	postive
ACSBG2	AC105339.2	0.71415541	3.13E-85	postive
CYP4B1	AC105339.2	0.66542527	3.30E-70	postive
FASN	AC105339.2	0.60119761	2.84E-54	postive
HSD17B3	AC017104.1	0.50381728	4.88E-36	postive
D2HGDH	AC017104.1	0.50000134	1.95E-35	postive
ACOT6	AL121672.1	0.61855565	3.23E-58	postive
ELOVL2	AC010524.1	0.76326812	6.00E-104	postive
ACSBG2	AC010524.1	0.68987994	2.25E-77	postive
CYP4B1	AC010524.1	0.80413714	2.13E-123	postive
FASN	AC010524.1	0.73317165	5.53E-92	postive
ALOX12	AC009084.1	0.56199417	3.35E-46	postive
ALOX12	AL590666.1	0.58876771	1.36E-51	postive
ACSBG2	AL590666.1	0.50366066	5.17E-36	postive
ACOT6	ANKRD44-IT1	0.65327039	6.82E-67	postive
CPT1B	AC008870.2	0.60473605	4.67E-55	postive
ALOX12	AC008870.2	0.59462744	7.65E-53	postive
CBR4	AL391001.1	0.50114114	1.29E-35	postive
ELOVL4	AC116312.1	0.56726527	3.18E-47	postive

GABARAPL1	AC116312.1	0.60118723	2.85E-54	postive
ELOVL2	LINC01290	0.81762828	7.38E-131	postive
ACSBG2	LINC01290	0.65468684	2.85E-67	postive
CYP4B1	LINC01290	0.87869837	1.39E-174	postive
FASN	LINC01290	0.80571312	3.07E-124	postive
ELOVL2	AC131009.1	0.71619309	6.29E-86	postive
ACSBG2	AC131009.1	0.59600926	3.85E-53	postive
CYP4B1	AC131009.1	0.72134233	1.02E-87	postive
FASN	AC131009.1	0.76363446	4.18E-104	postive
ELOVL2	AC013553.3	0.81179051	1.48E-127	postive
ACSBG2	AC013553.3	0.64609592	5.24E-65	postive
CYP4B1	AC013553.3	0.85686352	1.23E-156	postive
FASN	AC013553.3	0.78507693	8.50E-114	postive
ELOVL2	AC114271.1	0.62262557	3.53E-59	postive
CYP4B1	AC114271.1	0.63843752	4.75E-63	postive
FASN	AC114271.1	0.66211852	2.73E-69	postive
HSD17B3	DPP9-AS1	0.51880405	1.80E-38	postive
CPT1B	DPP9-AS1	0.55068992	4.56E-44	postive
D2HGDH	DPP9-AS1	0.50557228	2.57E-36	postive
ALOX12	DPP9-AS1	0.61312085	5.89E-57	postive

ACSBG2	DPP9-AS1	0.59726463	2.06E-53	postive
ALOX12	AC100830.2	0.53407942	4.46E-41	postive
ACOT6	AC100830.2	0.58746052	2.56E-51	postive
BMPR1B	AC078788.1	0.81419464	6.67E-129	postive
RDH11	AC078788.1	0.5950065	6.34E-53	postive
ALOX12	AC009159.2	0.56821709	2.07E-47	postive
ACOT6	AC009754.1	0.75763424	1.41E-101	postive
BMPR1B	AC009754.1	0.52510194	1.58E-39	postive
ALOX12	AL122035.2	0.59710488	2.23E-53	postive
HSD17B3	AL451050.2	0.50973473	5.53E-37	postive
CPT1B	AL451050.2	0.58982023	8.13E-52	postive
D2HGDH	AL451050.2	0.52565605	1.27E-39	postive
CPT1B	AL008582.1	0.54270933	1.31E-42	postive
ALOX12	AL008582.1	0.57087801	6.18E-48	postive
ACSBG2	AL008582.1	0.56473397	9.91E-47	postive
DPEP2	MMP2-AS1	0.54594325	3.39E-43	postive
ELOVL2	AC002310.1	0.70974802	9.60E-84	postive
ACSBG2	AC002310.1	0.59152993	3.52E-52	postive
CYP4B1	AC002310.1	0.77204021	8.89E-108	postive
FASN	AC002310.1	0.69944448	2.25E-80	postive
CPT1B	ZNF528-AS1	0.65688602	7.30E-68	postive

D2HGDH	ZNF528-AS1	0.51140309	2.97E-37	postive
ACOT6	AC090541.1	0.88426375	1.02E-179	postive
BMPR1B	AC090541.1	0.69837294	4.94E-80	postive
ELOVL2	LINC01985	0.82801592	4.88E-137	postive
ACSBG2	LINC01985	0.61882862	2.79E-58	postive
CYP4B1	LINC01985	0.90031734	3.77E-196	postive
FASN	LINC01985	0.82005384	2.89E-132	postive
ALOX12	LINC01126	0.66486716	4.72E-70	postive
ELOVL2	NUCB1-AS1	0.70794981	3.81E-83	postive
ACSBG2	NUCB1-AS1	0.74961866	2.61E-98	postive
CYP4B1	NUCB1-AS1	0.7564248	4.48E-101	postive
FASN	NUCB1-AS1	0.68773235	1.02E-76	postive
TBXAS1	AC006033.2	0.5813283	4.80E-50	postive
SERINC1	AC005288.1	0.51967716	1.29E-38	postive
PDHB	AC112220.2	0.51808002	2.38E-38	postive
HACL1	AC112220.2	0.55687041	3.18E-45	postive
BMPR1B	AC112220.2	0.54514161	4.74E-43	postive
GABARAPL1	AC112220.2	0.52722361	6.85E-40	postive
ALOX12	LINC01409	0.52983024	2.44E-40	postive
ELOVL2	AC011379.2	0.53246852	8.52E-41	postive
ALOX12	AC011379.2	0.57859963	1.74E-49	postive

ACSBG2	AC011379.2	0.64704739	2.97E-65	postive
CYP4B1	AC011379.2	0.56998923	9.26E-48	postive
FASN	AC011379.2	0.52627123	9.96E-40	postive
ACOT6	RDH10-AS1	0.86603321	8.70E-164	postive
BMPR1B	RDH10-AS1	0.65237589	1.18E-66	postive
PRKAG2	Z99572.1	0.52153832	6.30E-39	postive
PDHB	Z99572.1	0.50633162	1.95E-36	postive
HACD3	Z99572.1	0.51181403	2.55E-37	postive
GABARAPL1	Z99572.1	0.71389573	3.84E-85	postive
ACACB	Z99572.1	0.58038372	7.50E-50	postive
ELOVL2	AP000255.1	0.70303158	1.57E-81	postive
ACSBG2	AP000255.1	0.60158277	2.34E-54	postive
CYP4B1	AP000255.1	0.7632212	6.28E-104	postive
FASN	AP000255.1	0.67248908	3.31E-72	postive
ALOX12	AC098851.1	0.58166476	4.09E-50	postive
ALOX12	AL031717.1	0.62766321	2.18E-60	postive
PRKAG2	AL161729.1	0.5432564	1.04E-42	postive
PDHB	AL161729.1	0.52932504	2.98E-40	postive
AUH	AL161729.1	0.53640119	1.74E-41	postive
HACL1	AL161729.1	0.55467098	8.25E-45	postive
GABARAPL1	AL161729.1	0.53536926	2.65E-41	postive

ALOX12	AC007000.3	0.52589741	1.15E-39	postive
ELOVL2	AC073592.1	0.60360862	8.32E-55	postive
ACSBG2	AC073592.1	0.68523758	5.85E-76	postive
CYP4B1	AC073592.1	0.65701523	6.74E-68	postive
FASN	AC073592.1	0.58885619	1.30E-51	postive
ALOX12	TPT1-AS1	0.70271275	1.99E-81	postive
ACSBG2	TPT1-AS1	0.53223138	9.37E-41	postive
ACOT8	INSYN1-AS1	0.53556589	2.44E-41	postive
SDHD	INSYN1-AS1	0.72422293	9.80E-89	postive
PHYH	INSYN1-AS1	0.73991701	1.61E-94	postive
NDUFAB1	INSYN1-AS1	0.57586633	6.21E-49	postive
HACD3	INSYN1-AS1	0.76953634	1.15E-106	postive
AUH	INSYN1-AS1	0.57135219	4.97E-48	postive
ELOVL4	INSYN1-AS1	0.74874268	5.83E-98	postive
GABARAPL1	INSYN1-AS1	0.51210585	2.28E-37	postive
ACOT6	AC092422.1	0.79890684	1.16E-120	postive
BMPR1B	AC092422.1	0.83738688	5.58E-143	postive
RDH11	AC092422.1	0.52082912	8.28E-39	postive
GABARAPL1	AC092422.1	0.6087211	5.94E-56	postive
ACACB	AC092422.1	0.54159061	2.08E-42	postive

ALOX12	AC093864.1	0.52671547	8.37E-40	postive
ELOVL2	AC012186.2	0.76504385	1.04E-104	postive
ACSBG2	AC012186.2	0.70170896	4.20E-81	postive
CYP4B1	AC012186.2	0.82308483	4.70E-134	postive
FASN	AC012186.2	0.75946053	2.45E-102	postive
ACOT6	AC078883.1	0.57897636	1.45E-49	postive
ALOX12	AL157400.3	0.54027935	3.57E-42	postive
ACOT6	AL138895.1	0.71948234	4.58E-87	postive
BMPR1B	AL138895.1	0.50205034	9.28E-36	postive
HSD17B3	AL136304.1	0.54036706	3.44E-42	postive
CPT1B	AL136304.1	0.5407065	2.99E-42	postive
D2HGDH	AL136304.1	0.56177431	3.70E-46	postive
ACADVL	AL136304.1	0.5053373	2.80E-36	postive
ALOX12	AL136304.1	0.55104224	3.92E-44	postive
ELOVL2	AL807757.2	0.60698517	1.46E-55	postive
ACSBG2	AL807757.2	0.6447518	1.17E-64	postive
CYP4B1	AL807757.2	0.65663307	8.55E-68	postive
FASN	AL807757.2	0.57172738	4.19E-48	postive
ALOX12	AC012409.3	0.59134923	3.85E-52	postive
D2HGDH	AC118754.1	0.62635737	4.52E-60	postive
ACADVL	AC118754.1	0.55535072	6.15E-45	postive
FAAH	AC118754.1	0.50122809	1.25E-35	postive

ELOVL2	LINC02663	0.82987571	3.45E-138	postive
ACSBG2	LINC02663	0.64335489	2.67E-64	postive
CYP4B1	LINC02663	0.89434283	9.99E-190	postive
FASN	LINC02663	0.82880649	1.59E-137	postive
ELOVL2	AL121999.1	0.63776508	7.02E-63	postive
ACSBG2	AL121999.1	0.58696971	3.24E-51	postive
CYP4B1	AL121999.1	0.67624318	2.72E-73	postive
FASN	AL121999.1	0.64656751	3.95E-65	postive
ELOVL2	AL391095.1	0.5933343	1.45E-52	postive
ACSBG2	AL391095.1	0.68338795	2.10E-75	postive
CYP4B1	AL391095.1	0.6404141	1.50E-63	postive
FASN	AL391095.1	0.61118818	1.63E-56	postive
ACOT6	AC093801.1	0.81018424	1.14E-126	postive
BMPR1B	AC093801.1	0.59364556	1.24E-52	postive
SDHD	IQCH-AS1	0.62357958	2.09E-59	postive
PHYH	IQCH-AS1	0.57216592	3.43E-48	postive
HACD3	IQCH-AS1	0.67098776	8.89E-72	postive
AUH	IQCH-AS1	0.59524814	5.63E-53	postive
HACL1	IQCH-AS1	0.52656139	8.89E-40	postive
BMPR1B	IQCH-AS1	0.66844419	4.69E-71	postive
CBR4	IQCH-AS1	0.53749924	1.11E-41	postive

ELOVL4	IQCH-AS1	0.65913649	1.79E-68	postive
RDH11	IQCH-AS1	0.58208667	3.35E-50	postive
GABARAPL1	IQCH-AS1	0.59445352	8.34E-53	postive
ACACB	IQCH-AS1	0.57518869	8.51E-49	postive
CPT1B	AC001226.1	0.50578998	2.37E-36	postive
ALOX12	AC001226.1	0.53851221	7.37E-42	postive
ELOVL2	LINC00910	0.51112091	3.30E-37	postive
ACSBG2	LINC00910	0.55037883	5.20E-44	postive
CYP4B1	LINC00910	0.58151907	4.39E-50	postive
FASN	LINC00910	0.52799701	5.05E-40	postive
ACOT6	AC114763.1	0.52712788	7.11E-40	postive
ENO3	RNF157-AS1	0.50883635	7.72E-37	postive
PRKAG2	MCF2L-AS1	0.59704996	2.29E-53	postive
PDHB	MCF2L-AS1	0.55337439	1.44E-44	postive
HACD3	MCF2L-AS1	0.54309219	1.11E-42	postive
AUH	MCF2L-AS1	0.57331862	2.02E-48	postive
BMPR1B	MCF2L-AS1	0.62092783	8.93E-59	postive
ELOVL4	MCF2L-AS1	0.63151885	2.50E-61	postive
RDH11	MCF2L-AS1	0.64388045	1.96E-64	postive
GABARAPL1	MCF2L-AS1	0.72256497	3.79E-88	postive

ACACB	MCF2L-AS1	0.63288417	1.15E-61	postive
ALOX12	AC023510.1	0.51808394	2.38E-38	postive
DPEP2	AC145098.1	0.52937133	2.93E-40	postive
TBXAS1	AC145098.1	0.59663148	2.83E-53	postive
HACD3	LINC01543	0.53513214	2.91E-41	postive
GABARAPL1	LINC01543	0.51352687	1.34E-37	postive
ELOVL2	AL049539.1	0.61986575	1.59E-58	postive
ACSBG2	AL049539.1	0.66683105	1.33E-70	postive
CYP4B1	AL049539.1	0.66837419	4.90E-71	postive
FASN	AL049539.1	0.59447701	8.25E-53	postive
ELOVL2	AC145423.2	0.56627931	4.96E-47	postive
ALOX12	AC145423.2	0.60847818	6.74E-56	postive
ACSBG2	AC145423.2	0.67746491	1.19E-73	postive
CYP4B1	AC145423.2	0.6290126	1.03E-60	postive
FASN	AC145423.2	0.57137713	4.92E-48	postive
ELOVL2	AC113143.1	0.71226762	1.37E-84	postive
ACSBG2	AC113143.1	0.64593321	5.77E-65	postive
CYP4B1	AC113143.1	0.76986007	8.25E-107	postive
FASN	AC113143.1	0.69095098	1.05E-77	postive
ELOVL2	AC009090.1	0.73913426	3.20E-94	postive
ACSBG2	AC009090.1	0.67508787	5.88E-73	postive

CYP4B1	AC009090.1	0.81191193	1.27E-127	postive
FASN	AC009090.1	0.72161246	8.22E-88	postive
ELOVL2	AC000068.1	0.76163904	2.96E-103	postive
ACSBG2	AC000068.1	0.64058178	1.36E-63	postive
CYP4B1	AC000068.1	0.82912164	1.01E-137	postive
FASN	AC000068.1	0.78701625	9.95E-115	postive
ALOX12	AC012531.1	0.63962434	2.38E-63	postive
ACADVL	AL121992.3	0.5084493	8.91E-37	postive
FAAH	AL121992.3	0.58486028	8.93E-51	postive
ELOVL2	AC003965.2	0.81571562	9.18E-130	postive
ACSBG2	AC003965.2	0.62819125	1.63E-60	postive
CYP4B1	AC003965.2	0.88083059	1.60E-176	postive
FASN	AC003965.2	0.82140666	4.64E-133	postive
ELOVL2	AC004039.1	0.67612182	2.95E-73	postive
ACSBG2	AC004039.1	0.71520843	1.37E-85	postive
CYP4B1	AC004039.1	0.73435938	2.00E-92	postive
FASN	AC004039.1	0.65358041	5.63E-67	postive
ELOVL2	AC074029.3	0.63698437	1.10E-62	postive
ALOX12	AC074029.3	0.59356541	1.29E-52	postive
ACSBG2	AC074029.3	0.68196634	5.60E-75	postive
CYP4B1	AC074029.3	0.69962924	1.96E-80	postive

FASN	AC074029.3	0.64551562	7.41E-65	postive
D2HGDH	MIR210HG	0.61932826	2.13E-58	postive
ACADVL	MIR210HG	0.54291038	1.20E-42	postive
FAAH	MIR210HG	0.54810564	1.36E-43	postive
ALOX12	TMEM202-AS1	0.53252146	8.34E-41	postive
ACOT6	AP001033.2	0.64076764	1.22E-63	postive
ALOX12	NDUFV2-AS1	0.57397003	1.49E-48	postive
ACOT6	NDUFV2-AS1	0.57933804	1.23E-49	postive
ACOT6	AC011939.2	0.6057242	2.81E-55	postive
ELOVL2	AL138921.2	0.61902981	2.50E-58	postive
ACSBG2	AL138921.2	0.61777868	4.91E-58	postive
CYP4B1	AL138921.2	0.67736043	1.28E-73	postive
FASN	AL138921.2	0.5970992	2.24E-53	postive
ELOVL2	AC092338.1	0.57988391	9.49E-50	postive
ACSBG2	AC092338.1	0.66478876	4.97E-70	postive
CYP4B1	AC092338.1	0.62859374	1.30E-60	postive
FASN	AC092338.1	0.56035588	6.91E-46	postive
ALOX12	AC012313.6	0.63322619	9.50E-62	postive
ACOT6	AC023590.1	0.91346384	7.62E-212	postive
BMPR1B	AC023590.1	0.68582244	3.89E-76	postive
ELOVL2	AC138230.1	0.78190836	2.69E-112	postive

ACSBG2	AC138230.1	0.69533502	4.52E-79	postive
CYP4B1	AC138230.1	0.85042857	6.55E-152	postive
FASN	AC138230.1	0.77054014	4.13E-107	postive
PRKAG2	AP000757.1	0.53599983	2.05E-41	postive
SDHD	AP000757.1	0.50601002	2.19E-36	postive
PDHB	AP000757.1	0.58243367	2.84E-50	postive
GCDH	AP000757.1	0.50854948	8.58E-37	postive
HACD3	AP000757.1	0.54544772	4.17E-43	postive
AUH	AP000757.1	0.57441026	1.22E-48	postive
HACL1	AP000757.1	0.51226227	2.15E-37	postive
BMPR1B	AP000757.1	0.54729362	1.92E-43	postive
ELOVL4	AP000757.1	0.64600364	5.54E-65	postive
RDH11	AP000757.1	0.61046231	2.39E-56	postive
GABARAPL1	AP000757.1	0.79842571	2.06E-120	postive
ACACB	AP000757.1	0.59300443	1.71E-52	postive
ELOVL2	AC006960.2	0.779058	5.75E-111	postive
ACSBG2	AC006960.2	0.67165438	5.73E-72	postive
CYP4B1	AC006960.2	0.82758521	8.97E-137	postive
FASN	AC006960.2	0.76548027	6.73E-105	postive
HSD17B3	AL355488.1	0.55499682	7.17E-45	postive
CPT1B	AL355488.1	0.59338514	1.42E-52	postive

ALOX12	AL355488.1	0.61753389	5.60E-58	postive
ALOX12	AL357874.2	0.62280165	3.21E-59	postive
ACSBG2	AL357874.2	0.70361974	1.01E-81	postive
CYP4B1	AL357874.2	0.54293845	1.19E-42	postive
ELOVL2	AC012676.4	0.5979693	1.45E-53	postive
ALOX12	AC012676.4	0.50928804	6.53E-37	postive
ACSBG2	AC012676.4	0.7120065	1.67E-84	postive
CYP4B1	AC012676.4	0.64325096	2.84E-64	postive
FASN	AC012676.4	0.57564598	6.88E-49	postive
ELOVL2	AL731563.3	0.50659262	1.77E-36	postive
ALOX12	AL731563.3	0.64596076	5.68E-65	postive
ACSBG2	AL731563.3	0.68072629	1.31E-74	postive
CYP4B1	AL731563.3	0.55958487	9.71E-46	postive
CPT1B	AL354733.3	0.59241541	2.28E-52	postive
D2HGDH	AL354733.3	0.52674768	8.26E-40	postive
ALOX12	AL354733.3	0.68391314	1.46E-75	postive
ELOVL2	AL136531.1	0.53888802	6.32E-42	postive
ALOX12	AL136531.1	0.58958119	9.13E-52	postive
ACSBG2	AL136531.1	0.66628826	1.89E-70	postive
CYP4B1	AL136531.1	0.60184018	2.05E-54	postive
FASN	AL136531.1	0.55356751	1.33E-44	postive

PRKAG2	AC010776.2	0.53603266	2.02E-41	postive
GABARAPL1	AC010776.2	0.73250936	9.73E-92	postive
ACACB	AC010776.2	0.55377024	1.22E-44	postive
CPT1B	AC132192.2	0.64132603	8.82E-64	postive
D2HGDH	AC132192.2	0.56221704	3.04E-46	postive
ALOX12	AC132192.2	0.55886785	1.33E-45	postive
D2HGDH	PHKA2-AS1	0.54295552	1.18E-42	postive
ACADVL	PHKA2-AS1	0.59431335	8.94E-53	postive
ALOX12	AL355075.2	0.69811275	5.97E-80	postive
ACOT6	AC078955.1	0.90928492	1.34E-206	postive
BMPR1B	AC078955.1	0.68870145	5.17E-77	postive
ALOX12	DGUOK-AS1	0.5437249	8.57E-43	postive
ELOVL2	AC092720.1	0.72666786	1.31E-89	postive
ACSBG2	AC092720.1	0.68811863	7.80E-77	postive
CYP4B1	AC092720.1	0.81932911	7.65E-132	postive
FASN	AC092720.1	0.72143197	9.51E-88	postive
AUH	LINC02568	0.5031401	6.25E-36	postive
GABARAPL1	LINC02568	0.61753547	5.59E-58	postive
ELOVL2	LINC01012	0.63899838	3.43E-63	postive
ACSBG2	LINC01012	0.59966899	6.15E-54	postive
CYP4B1	LINC01012	0.661307	4.56E-69	postive
FASN	LINC01012	0.64613413	5.12E-65	postive

ELOVL2	SYNE1-AS1	0.52287079	3.76E-39	postive
ACSBG2	SYNE1-AS1	0.55946781	1.02E-45	postive
CYP4B1	SYNE1-AS1	0.58818619	1.80E-51	postive
ELOVL4	AC129507.4	0.55169554	2.97E-44	postive
RDH11	AC129507.4	0.51253239	1.95E-37	postive
ALOX12	AC067852.3	0.72773311	5.40E-90	postive
ACOT6	AC130650.2	0.61525686	1.90E-57	postive
PRKAG2	MAP4K3-DT	0.51366811	1.27E-37	postive
ELOVL2	LINC01827	0.60625814	2.13E-55	postive
ACSBG2	LINC01827	0.52741665	6.35E-40	postive
CYP4B1	LINC01827	0.65793315	3.80E-68	postive
FASN	LINC01827	0.60226306	1.65E-54	postive
SDHD	AL162400.2	0.62167592	5.94E-59	postive
PHYH	AL162400.2	0.60451921	5.21E-55	postive
NDUFAB1	AL162400.2	0.53663127	1.59E-41	postive
HACD3	AL162400.2	0.65532418	1.92E-67	postive
ELOVL4	AL162400.2	0.56950891	1.15E-47	postive
ELOVL2	BET1-AS1	0.52158511	6.19E-39	postive
ACSBG2	BET1-AS1	0.61127396	1.56E-56	postive
CYP4B1	BET1-AS1	0.54277127	1.27E-42	postive

ELOVL2	FRY-AS1	0.81732764	1.10E-130	postive
ACSBG2	FRY-AS1	0.68478328	8.02E-76	postive
CYP4B1	FRY-AS1	0.87955759	2.32E-175	postive
FASN	FRY-AS1	0.81089714	4.63E-127	postive
ELOVL2	AL360091.1	0.73515162	1.02E-92	postive
ACSBG2	AL360091.1	0.69551177	3.98E-79	postive
CYP4B1	AL360091.1	0.79864914	1.58E-120	postive
FASN	AL360091.1	0.71778985	1.77E-86	postive
ELOVL2	AC107993.1	0.82276168	7.32E-134	postive
ACSBG2	AC107993.1	0.70707285	7.43E-83	postive
CYP4B1	AC107993.1	0.86598761	9.47E-164	postive
FASN	AC107993.1	0.79626233	2.63E-119	postive
ELOVL2	AC006273.1	0.5284485	4.22E-40	postive
CYP4B1	AC006273.1	0.60337922	9.35E-55	postive
FASN	AC006273.1	0.50748324	1.27E-36	postive
ACOT6	AC099811.1	0.54210246	1.68E-42	postive
CPT1B	NFE2L1-DT	0.52425835	2.19E-39	postive
ALOX12	NFE2L1-DT	0.57946917	1.15E-49	postive
PTGS2	LINC01679	0.5010956	1.31E-35	postive
ACOT6	LINC01768	0.54375235	8.47E-43	postive
BMPR1B	LINC01768	0.90194554	5.69E-198	postive

RDH11	LINC01768	0.68518405	6.07E-76	postive
GABARAPL1	LINC01768	0.56995187	9.42E-48	postive
ACACB	LINC01768	0.54160541	2.06E-42	postive
ALOX12	AL158835.2	0.64068575	1.28E-63	postive
ACOT6	SRI-AS1	0.59389036	1.10E-52	postive
CPT1B	AP006621.4	0.55068608	4.56E-44	postive
D2HGDH	AP006621.4	0.64438371	1.45E-64	postive
ACADVL	AP006621.4	0.5044023	3.95E-36	postive
ALOX12	AP006621.4	0.63859534	4.34E-63	postive
ALOX12	AC021851.1	0.52956488	2.71E-40	postive
CPT1B	AL358072.1	0.56004527	7.93E-46	postive
CPT1B	TTC28-AS1	0.5667639	3.99E-47	postive
D2HGDH	TTC28-AS1	0.56413274	1.30E-46	postive
ALOX12	TTC28-AS1	0.64624633	4.79E-65	postive
ELOVL2	AC005324.4	0.6100309	3.00E-56	postive
ACSBG2	AC005324.4	0.62948774	7.87E-61	postive
CYP4B1	AC005324.4	0.63006192	5.70E-61	postive
FASN	AC005324.4	0.61820897	3.89E-58	postive
ACOT6	SREBF2-AS1	0.58621915	4.65E-51	postive
BMPR1B	SREBF2-AS1	0.56903782	1.43E-47	postive
ALOX12	AC091185.1	0.6957054	3.46E-79	postive
ELOVL2	AL034417.2	0.73462769	1.59E-92	postive

ACSBG2	AL034417.2	0.62390743	1.75E-59	postive
CYP4B1	AL034417.2	0.77338487	2.22E-108	postive
FASN	AL034417.2	0.71742697	2.36E-86	postive
D2HGDH	AP001107.8	0.54686283	2.30E-43	postive
ALOX12	TTN-AS1	0.54036689	3.44E-42	postive
ALOX12	FAM182B	0.54878177	1.02E-43	postive
ACSBG2	FAM182B	0.50531021	2.83E-36	postive
ALOX12	AC011477.1	0.53427594	4.12E-41	postive
CBR4	AC011477.1	0.52555697	1.32E-39	postive
ELOVL2	AL109947.1	0.81098089	4.16E-127	postive
ACSBG2	AL109947.1	0.71047893	5.47E-84	postive
CYP4B1	AL109947.1	0.87736884	2.14E-173	postive
FASN	AL109947.1	0.79472305	1.58E-118	postive
HACL1	ERVE-1	0.52902975	3.35E-40	postive
BMPR1B	ERVE-1	0.5772515	3.26E-49	postive
RDH11	ERVE-1	0.55759677	2.32E-45	postive
GABARAPL1	ERVE-1	0.52195635	5.36E-39	postive
CPT1B	AC087289.5	0.54939404	7.91E-44	postive
D2HGDH	AC087289.5	0.55539769	6.03E-45	postive
ELOVL2	AP003086.1	0.67546412	4.58E-73	postive
ACSBG2	AP003086.1	0.67178611	5.26E-72	postive

CYP4B1	AP003086.1	0.72972374	1.02E-90	postive
FASN	AP003086.1	0.65451013	3.18E-67	postive
HSD17B3	AC015813.1	0.52262044	4.14E-39	postive
ALOX12	AC015813.1	0.61833683	3.63E-58	postive
ELOVL2	LINC02416	0.59703187	2.31E-53	postive
ACSBG2	LINC02416	0.53289313	7.18E-41	postive
CYP4B1	LINC02416	0.6082968	7.41E-56	postive
FASN	LINC02416	0.62786084	1.96E-60	postive
ELOVL2	AL021707.2	0.54375892	8.45E-43	postive
ALOX12	AL021707.2	0.59942045	6.97E-54	postive
ACSBG2	AL021707.2	0.65063263	3.41E-66	postive
CYP4B1	AL021707.2	0.60191937	1.97E-54	postive
FASN	AL021707.2	0.53522223	2.81E-41	postive
ACOT6	AC021739.3	0.57640699	4.83E-49	postive
ELOVL2	AL078587.1	0.56649587	4.50E-47	postive
ALOX12	AL078587.1	0.54720829	1.99E-43	postive
ACSBG2	AL078587.1	0.6952624	4.76E-79	postive
CYP4B1	AL078587.1	0.59045958	5.95E-52	postive
FASN	AL078587.1	0.55246524	2.13E-44	postive
CPT1B	AC103691.1	0.55632459	4.03E-45	postive
D2HGDH	AC103691.1	0.52718217	6.96E-40	postive

ACOT6	AL731568.1	0.51943944	1.41E-38	postive
CPT1B	LINC02878	0.5805375	6.98E-50	postive
ALOX12	KANSL1L-AS1	0.57575071	6.56E-49	postive
ACOT8	LINC01607	0.58814207	1.84E-51	postive
SDHD	LINC01607	0.66808393	5.92E-71	postive
PHYH	LINC01607	0.69853952	4.37E-80	postive
NDUFAB1	LINC01607	0.60190887	1.98E-54	postive
HACD3	LINC01607	0.70901923	1.68E-83	postive
ELOVL4	LINC01607	0.79102831	1.10E-116	postive
CPT1B	RUSC1-AS1	0.69880659	3.59E-80	postive
D2HGDH	RUSC1-AS1	0.72822863	3.58E-90	postive
ACADVL	RUSC1-AS1	0.50340963	5.67E-36	postive
ALOX12	RUSC1-AS1	0.7608391	6.44E-103	postive
D2HGDH	AC044849.1	0.5024753	7.96E-36	postive
ELOVL2	LINC01841	0.83886382	5.97E-144	postive
ACSBG2	LINC01841	0.67478982	7.18E-73	postive
CYP4B1	LINC01841	0.88102548	1.06E-176	postive
FASN	LINC01841	0.83713956	8.10E-143	postive
ELOVL2	AC021491.4	0.73385865	3.08E-92	postive
ACSBG2	AC021491.4	0.67499534	6.26E-73	postive
CYP4B1	AC021491.4	0.80433317	1.68E-123	postive
FASN	AC021491.4	0.74172446	3.26E-95	postive

CPT1B	AL365330.1	0.64258523	4.20E-64	postive
D2HGDH	AL365330.1	0.69218477	4.36E-78	postive
ACADVL	AL365330.1	0.57373909	1.66E-48	postive
ALOX12	AL365330.1	0.65690494	7.22E-68	postive
ACOT6	AC106873.1	0.86987923	6.04E-167	postive
BMPR1B	AC106873.1	0.627263	2.73E-60	postive
SDHD	AC099552.3	0.55572408	5.23E-45	postive
PHYH	AC099552.3	0.50809409	1.02E-36	postive
HACD3	AC099552.3	0.51555001	6.23E-38	postive
ACOT6	AC099552.3	0.55872993	1.41E-45	postive
BMPR1B	AC099552.3	0.7344359	1.88E-92	postive
ELOVL4	AC099552.3	0.590592	5.58E-52	postive
RDH11	AC099552.3	0.6428572	3.58E-64	postive
CPT1B	MCCC1-AS1	0.59812956	1.33E-53	postive
D2HGDH	MCCC1-AS1	0.51532943	6.78E-38	postive
ALOX12	MCCC1-AS1	0.63331852	9.02E-62	postive
ACOT6	AC036103.1	0.59480307	7.02E-53	postive
ACOT6	AC009163.4	0.54619522	3.05E-43	postive
ELOVL2	AC107057.1	0.55102469	3.95E-44	postive
CYP4B1	AC107057.1	0.60472186	4.70E-55	postive
FASN	AC107057.1	0.58347842	1.73E-50	postive
ACOT6	AC040174.1	0.86414873	2.83E-162	postive
BMPR1B	AC040174.1	0.8884703	8.87E-184	postive

RDH11	AC040174.1	0.53197913	1.04E-40	postive
ACOT6	ST7-OT4	0.89032658	1.27E-185	postive
BMPR1B	ST7-OT4	0.63768427	7.36E-63	postive
ELOVL2	AC103923.1	0.64163597	7.35E-64	postive
ALOX12	AC103923.1	0.50141834	1.17E-35	postive
ACSBG2	AC103923.1	0.67572105	3.85E-73	postive
CYP4B1	AC103923.1	0.68474694	8.22E-76	postive
FASN	AC103923.1	0.62185214	5.39E-59	postive
ELOVL2	TOB1-AS1	0.65607853	1.21E-67	postive
ACSBG2	TOB1-AS1	0.59464877	7.57E-53	postive
CYP4B1	TOB1-AS1	0.74086371	6.98E-95	postive
FASN	TOB1-AS1	0.68560209	4.54E-76	postive
D2HGDH	AL121845.4	0.60354123	8.61E-55	postive
FAAH	AL121845.4	0.604823	4.46E-55	postive
ALOX12	AC004771.2	0.52751569	6.10E-40	postive
KMT5A	AL133215.2	0.52009405	1.10E-38	postive
PRKAA2	AC021483.2	0.63844267	4.74E-63	postive
ALOX12	AC011306.1	0.54889606	9.76E-44	postive
ELOVL2	RAD21-AS1	0.76115357	4.74E-103	postive
ACSBG2	RAD21-AS1	0.57488837	9.78E-49	postive
CYP4B1	RAD21-AS1	0.81981253	4.00E-132	postive

FASN	RAD21-AS1	0.72317343	2.31E-88	postive
ALOX12	FMR1-IT1	0.59991724	5.43E-54	postive
ELOVL2	AC087301.1	0.82579309	1.11E-135	postive
ACSBG2	AC087301.1	0.68266456	3.47E-75	postive
CYP4B1	AC087301.1	0.88773289	4.69E-183	postive
FASN	AC087301.1	0.82191052	2.34E-133	postive
ALOX12	AL353803.5	0.56152476	4.13E-46	postive
ACSBG2	AL353803.5	0.56771761	2.59E-47	postive
CPT1B	AC120053.1	0.5315384	1.24E-40	postive
D2HGDH	AC120053.1	0.52165515	6.02E-39	postive
HSD17B3	AP002807.1	0.51782821	2.62E-38	postive
CPT1B	AP002807.1	0.60168543	2.22E-54	postive
D2HGDH	AP002807.1	0.52762438	5.85E-40	postive
ALOX12	AP002807.1	0.50353056	5.42E-36	postive
ACOT6	AL391834.1	0.51809176	2.37E-38	postive
CBR4	AL391834.1	0.53204135	1.01E-40	postive
HSD17B3	AC040162.3	0.54915136	8.76E-44	postive
CPT1B	AC040162.3	0.71759331	2.07E-86	postive
D2HGDH	AC040162.3	0.70088812	7.73E-81	postive
ACADVL	AC040162.3	0.54277782	1.27E-42	postive
ALOX12	AC040162.3	0.52079563	8.39E-39	postive

ALOX12	AP001271.1	0.50096353	1.38E-35	postive
ALOX12	AC010976.1	0.61113344	1.68E-56	postive
ACSBG2	AC010976.1	0.63770296	7.28E-63	postive
HSD17B3	AC009159.1	0.58911736	1.14E-51	postive
ALOX12	AL158163.1	0.58186205	3.73E-50	postive
ACOT6	AL158163.1	0.57529073	8.11E-49	postive
ELOVL2	AC096540.1	0.8242874	8.98E-135	postive
ACSBG2	AC096540.1	0.63815302	5.61E-63	postive
CYP4B1	AC096540.1	0.8706426	1.39E-167	postive
FASN	AC096540.1	0.8107219	5.78E-127	postive
CPT1B	AC013356.2	0.53479598	3.34E-41	postive
D2HGDH	AC013356.2	0.50382615	4.87E-36	postive
ALOX12	AC013356.2	0.58223161	3.13E-50	postive
ELOVL2	AL031289.1	0.81924944	8.51E-132	postive
ACSBG2	AL031289.1	0.65331172	6.64E-67	postive
CYP4B1	AL031289.1	0.88735547	1.09E-182	postive
FASN	AL031289.1	0.82157042	3.72E-133	postive
ACOT6	AC139720.1	0.89161329	6.41E-187	postive
BMPR1B	AC139720.1	0.66965735	2.13E-71	postive
ELOVL2	AL583856.2	0.61532338	1.83E-57	postive
ACSBG2	AL583856.2	0.65056951	3.54E-66	postive
CYP4B1	AL583856.2	0.68520369	5.99E-76	postive

FASN	AL583856.2	0.53277236	7.54E-41	postive
ALOX12	AC139887.2	0.67473206	7.46E-73	postive
ACACB	MIR600HG	0.68289	2.97E-75	postive
ELOVL2	AC012360.2	0.53676201	1.50E-41	postive
CYP4B1	AC012360.2	0.5853047	7.22E-51	postive
ELOVL2	AP001207.3	0.74547524	1.14E-96	postive
ACSBG2	AP001207.3	0.68601935	3.39E-76	postive
CYP4B1	AP001207.3	0.78439568	1.80E-113	postive
FASN	AP001207.3	0.71907582	6.34E-87	postive
ALOX12	AC068790.5	0.51244108	2.01E-37	postive
CPT1B	GARS1-DT	0.50834847	9.25E-37	postive
ALOX12	GARS1-DT	0.64748464	2.28E-65	postive
ALOX12	RBM5-AS1	0.64429141	1.53E-64	postive
ACSBG2	RBM5-AS1	0.60994523	3.13E-56	postive
CYP4B1	RBM5-AS1	0.51415445	1.06E-37	postive
ELOVL2	SPON1-AS1	0.78511212	8.17E-114	postive
ACSBG2	SPON1-AS1	0.68889025	4.53E-77	postive
CYP4B1	SPON1-AS1	0.84792028	3.97E-150	postive
FASN	SPON1-AS1	0.78343258	5.15E-113	postive
SDHD	AC011503.1	0.62414132	1.54E-59	postive
PHYH	AC011503.1	0.6514514	2.07E-66	postive
NDUFAB1	AC011503.1	0.5635116	1.71E-46	postive

HACD3	AC011503.1	0.61823638	3.84E-58	postive
ELOVL4	AC011503.1	0.66517973	3.86E-70	postive
HSD17B3	AL121782.1	0.60313001	1.06E-54	postive
ALOX12	AC092123.1	0.72537662	3.80E-89	postive
ACSBG2	AC092123.1	0.65283521	8.90E-67	postive
CYP4B1	AC092123.1	0.50184213	1.00E-35	postive
HSD17B3	AL035071.1	0.54535006	4.35E-43	postive
ELOVL2	AC018450.1	0.52749791	6.15E-40	postive
CYP4B1	AC018450.1	0.53583451	2.19E-41	postive
D2HGDH	AL137127.1	0.54088846	2.78E-42	postive
ACADVL	AL137127.1	0.52449253	2.00E-39	postive
ACSBG2	AC020978.3	0.58875283	1.37E-51	postive
ELOVL2	DENND5B-AS1	0.67606736	3.06E-73	postive
ACSBG2	DENND5B-AS1	0.62891146	1.09E-60	postive
CYP4B1	DENND5B-AS1	0.71810366	1.38E-86	postive
FASN	DENND5B-AS1	0.68168956	6.77E-75	postive
PRKAG2	LINC01230	0.51287544	1.71E-37	postive
SDHD	LINC01230	0.56915266	1.35E-47	postive
PHYH	LINC01230	0.53833628	7.92E-42	postive
PDHB	LINC01230	0.50271516	7.29E-36	postive

HACD3	LINC01230	0.61180891	1.18E-56	postive
AUH	LINC01230	0.6432779	2.79E-64	postive
ELOVL4	LINC01230	0.61626052	1.11E-57	postive
RDH11	LINC01230	0.53476822	3.38E-41	postive
GABARAPL1	LINC01230	0.81365294	1.35E-128	postive
ACACB	LINC01230	0.56229371	2.94E-46	postive
CPT1B	AL139349.1	0.54508818	4.85E-43	postive
ELOVL2	LINC02289	0.72511227	4.72E-89	postive
ACSBG2	LINC02289	0.53649429	1.68E-41	postive
CYP4B1	LINC02289	0.80117159	7.77E-122	postive
FASN	LINC02289	0.63481646	3.83E-62	postive
HSD17B3	AC005837.4	0.57512199	8.77E-49	postive
CPT1B	AL592211.1	0.6565438	9.03E-68	postive
D2HGDH	AL592211.1	0.57038376	7.74E-48	postive
HSD17B3	AC004918.1	0.55725267	2.69E-45	postive
D2HGDH	AC004918.1	0.62013002	1.38E-58	postive
ACADVL	AC004918.1	0.54017578	3.72E-42	postive
ELOVL2	AC084809.1	0.57740416	3.04E-49	postive
CYP4B1	AC084809.1	0.63531024	2.89E-62	postive
FASN	AC084809.1	0.59588334	4.10E-53	postive
ELOVL2	AL022344.1	0.51772341	2.73E-38	postive
CYP4B1	AL022344.1	0.5918793	2.97E-52	postive

FASN	AL022344.1	0.52296767	3.62E-39	postive
ACSBG1	AC073263.1	0.52955942	2.72E-40	postive
ENO3	AC073263.1	0.50223539	8.68E-36	postive
ALOX12	AC008982.2	0.52555416	1.32E-39	postive
ELOVL2	AL157904.1	0.69295592	2.51E-78	postive
ACSBG2	AL157904.1	0.58945023	9.73E-52	postive
CYP4B1	AL157904.1	0.72643713	1.58E-89	postive
FASN	AL157904.1	0.72111683	1.23E-87	postive
ACOT6	AC093585.1	0.55560199	5.52E-45	postive
ACAA2	AC009060.1	0.53728472	1.22E-41	postive
ELOVL2	LINC02798	0.58976482	8.35E-52	postive
CYP4B1	LINC02798	0.60815448	7.97E-56	postive
FASN	LINC02798	0.59796795	1.45E-53	postive
ALOX12	AC025178.1	0.55487528	7.56E-45	postive
ACOT6	AC023158.2	0.84829264	2.17E-150	postive
BMPR1B	AC023158.2	0.86444927	1.63E-162	postive
RDH11	AC023158.2	0.5312667	1.38E-40	postive
ELOVL2	FO393418.1	0.80772498	2.53E-125	postive
ACSBG2	FO393418.1	0.68159064	7.24E-75	postive
CYP4B1	FO393418.1	0.84130449	1.41E-145	postive
FASN	FO393418.1	0.80972462	2.05E-126	postive

ELOVL2	AC010422.2	0.77756296	2.81E-110	postive
ACSBG2	AC010422.2	0.7055236	2.41E-82	postive
CYP4B1	AC010422.2	0.82933623	7.47E-138	postive
FASN	AC010422.2	0.78473363	1.24E-113	postive
HSD17B3	U47924.3	0.57644487	4.75E-49	postive
CPT1B	U47924.3	0.5489549	9.52E-44	postive
ALOX12	OSGEPL1-AS1	0.58354295	1.68E-50	postive
ACACB	AC010205.1	0.50894163	7.42E-37	postive
ACOT6	AL035658.1	0.5531551	1.59E-44	postive
ALOX12	AC011442.1	0.52772812	5.61E-40	postive
PON1	LINC02766	0.56063429	6.12E-46	postive
ADH1A	LINC02766	0.56018645	7.45E-46	postive
ADH4	LINC02766	0.56016951	7.51E-46	postive
FABP1	LINC02766	0.52333253	3.14E-39	postive
ADH1C	LINC02766	0.53914293	5.69E-42	postive
RDH16	LINC02766	0.56914459	1.36E-47	postive
ALOX12	TSPOAP1-AS1	0.5441489	7.18E-43	postive
ACOT6	AC027607.1	0.52627066	9.96E-40	postive
ELOVL2	AC008514.1	0.62178463	5.60E-59	postive
CYP4B1	AC008514.1	0.6575999	4.68E-68	postive
FASN	AC008514.1	0.63239023	1.53E-61	postive
ALOX12	SEC62-AS1	0.54473551	5.62E-43	postive
ALOX12	AC079921.1	0.52803487	4.97E-40	postive

ELOVL2	AC009831.3	0.78633999	2.11E-114	postive
ACSBG2	AC009831.3	0.70182113	3.86E-81	postive
CYP4B1	AC009831.3	0.8494063	3.52E-151	postive
FASN	AC009831.3	0.77140887	1.70E-107	postive
HSD17B3	AL117379.1	0.62679729	3.54E-60	postive
CPT1B	AL117379.1	0.57897709	1.45E-49	postive
D2HGDH	AL117379.1	0.50876556	7.92E-37	postive
ALOX12	AL117379.1	0.50343742	5.61E-36	postive
HSD17B3	AP001767.2	0.50487448	3.32E-36	postive
D2HGDH	AP001767.2	0.55563897	5.43E-45	postive
ALOX12	AP001767.2	0.54397585	7.72E-43	postive
ELOVL2	AC034102.5	0.63868669	4.11E-63	postive
ACSBG2	AC034102.5	0.64753569	2.21E-65	postive
CYP4B1	AC034102.5	0.70069996	8.88E-81	postive
FASN	AC034102.5	0.60937259	4.23E-56	postive
HSD17B3	LINC02604	0.60966677	3.63E-56	postive
CPT1B	LINC02604	0.61620995	1.14E-57	postive
D2HGDH	LINC02604	0.63514763	3.17E-62	postive
ACADVL	LINC02604	0.50620131	2.04E-36	postive
HSD17B3	AL110115.2	0.51093985	3.53E-37	postive
ALOX12	LINC02157	0.50253086	7.80E-36	postive

ELOVL2	AC096733.2	0.67528919	5.14E-73	postive
CYP4B1	AC096733.2	0.76233902	1.49E-103	postive
FASN	AC096733.2	0.69193551	5.21E-78	postive
HSD17B3	U91328.3	0.59808355	1.37E-53	postive
D2HGDH	U91328.3	0.51965854	1.30E-38	postive
ALOX12	AC074138.1	0.60102276	3.10E-54	postive
ACOT6	AC074138.1	0.53574431	2.27E-41	postive
ACOT6	AC022973.4	0.58190755	3.65E-50	postive
HSD17B3	AL359504.1	0.55213695	2.46E-44	postive
CPT1B	AL359504.1	0.56557922	6.79E-47	postive
D2HGDH	AL359504.1	0.59042529	6.05E-52	postive
ALOX12	AL359504.1	0.56274823	2.40E-46	postive
ELOVL2	AC091180.2	0.80291548	9.45E-123	postive
ACSBG2	AC091180.2	0.70213819	3.05E-81	postive
CYP4B1	AC091180.2	0.86756142	4.97E-165	postive
FASN	AC091180.2	0.8070248	6.05E-125	postive
ALOX12	AL158151.2	0.62189003	5.28E-59	postive
ACSBG2	AL158151.2	0.59562861	4.66E-53	postive
CPT1B	AC073957.3	0.5313053	1.36E-40	postive
D2HGDH	AC073957.3	0.51635184	4.60E-38	postive
ALOX12	AC073957.3	0.68006978	2.05E-74	postive
CPT1B	LINC02062	0.5835976	1.63E-50	postive

D2HGDH	LINC02062	0.5206416	8.90E-39	postive
PRKAG2	AL157935.1	0.51279625	1.76E-37	postive
ENO3	AL157935.1	0.5371497	1.28E-41	postive
CPT1B	AC245060.6	0.51236524	2.07E-37	postive
ALOX12	AC245060.6	0.69502988	5.64E-79	postive
HSD17B3	CR559946.2	0.5340485	4.51E-41	postive
CPT1B	CR559946.2	0.69982922	1.69E-80	postive
D2HGDH	CR559946.2	0.66745254	8.92E-71	postive
ACADVL	CR559946.2	0.55041095	5.13E-44	postive
ALOX12	CR559946.2	0.57441514	1.22E-48	postive
ACSBG2	GLIS3-AS1	0.50899521	7.28E-37	postive
ELOVL2	AL158847.1	0.75283763	1.32E-99	postive
ACSBG2	AL158847.1	0.56537106	7.45E-47	postive
CYP4B1	AL158847.1	0.78971256	4.86E-116	postive
FASN	AL158847.1	0.74323427	8.51E-96	postive
D2HGDH	AL731571.1	0.53193085	1.06E-40	postive
ALOX12	AL731571.1	0.69368722	1.49E-78	postive
ACOT6	AC012404.2	0.5885368	1.52E-51	postive
ACOT6	AC092279.1	0.53030181	2.02E-40	postive
CBR4	AC092279.1	0.51743057	3.05E-38	postive
ALOX12	PPP3CB-AS1	0.62080461	9.55E-59	postive
ELOVL2	AC018521.5	0.71393958	3.71E-85	postive

ACSBG2	AC018521.5	0.56923702	1.30E-47	postive
CYP4B1	AC018521.5	0.80195697	3.02E-122	postive
FASN	AC018521.5	0.73899525	3.61E-94	postive
D2HGDH	IBA57-DT	0.52324953	3.24E-39	postive
ALOX12	IBA57-DT	0.57119556	5.34E-48	postive
ACSBG2	IBA57-DT	0.52160483	6.14E-39	postive
ACOT6	CARMN	0.726519	1.48E-89	postive
ELOVL2	DDN-AS1	0.53574877	2.27E-41	postive
ACSBG2	DDN-AS1	0.54239163	1.49E-42	postive
CYP4B1	DDN-AS1	0.53822075	8.30E-42	postive
FASN	DDN-AS1	0.58208197	3.36E-50	postive
ELOVL2	AL021937.1	0.56892186	1.50E-47	postive
ALOX12	AL021937.1	0.53933927	5.25E-42	postive
ACSBG2	AL021937.1	0.64036173	1.55E-63	postive
CYP4B1	AL021937.1	0.60014525	4.84E-54	postive
FASN	AL021937.1	0.60530156	3.49E-55	postive
GABARAPL1	LINC02185	0.76745398	9.37E-106	postive
ACACB	LINC02185	0.53594744	2.09E-41	postive
ACSBG1	AL109804.1	0.64023105	1.67E-63	postive
ENO3	AL109804.1	0.58443475	1.09E-50	postive
ALOX12	DTX2P1-UPK3BP1-PMS2P11	0.55695988	3.06E-45	postive

ACOT6	AC008124.1	0.61305646	6.10E-57	postive
BMPR1B	AC008124.1	0.53275511	7.59E-41	postive
CBR4	AC008124.1	0.63669063	1.31E-62	postive
ACACB	AC008124.1	0.57443225	1.21E-48	postive
ALOX12	PCBP1-AS1	0.7050841	3.35E-82	postive
ALOX12	AC012157.2	0.51356072	1.32E-37	postive
HSD17B3	AC009159.3	0.64743514	2.35E-65	postive
D2HGDH	AC009159.3	0.52912015	3.24E-40	postive
ACOT6	AC100763.1	0.77543382	2.64E-109	postive
BMPR1B	AC100763.1	0.53541623	2.60E-41	postive
ELOVL2	LINC01356	0.6515781	1.92E-66	postive
ACSBG2	LINC01356	0.57728236	3.21E-49	postive
CYP4B1	LINC01356	0.67910946	3.93E-74	postive
FASN	LINC01356	0.66722147	1.04E-70	postive
ELOVL2	AL354928.1	0.53966802	4.59E-42	postive
ACSBG2	AL354928.1	0.57203891	3.63E-48	postive
CYP4B1	AL354928.1	0.57943055	1.17E-49	postive
FASN	AL354928.1	0.52760534	5.89E-40	postive
ACOT6	AC010261.2	0.68501092	6.84E-76	postive
ACOT6	LINC02334	0.89786724	1.81E-193	postive
BMPR1B	LINC02334	0.69383218	1.34E-78	postive

ALOX12	AC145285.2	0.53437032	3.96E-41	postive
ACSBG1	IGFL2-AS1	0.79584082	4.30E-119	postive
ENO3	IGFL2-AS1	0.71750269	2.23E-86	postive
PCBD1	IGFL2-AS1	0.67190005	4.88E-72	postive
CPT1B	LINC00106	0.6136821	4.38E-57	postive
D2HGDH	LINC00106	0.59132028	3.91E-52	postive
ALOX12	LINC00106	0.5860507	5.04E-51	postive
CPT1B	AC079174.2	0.58986206	7.97E-52	postive
ALOX12	LINC00861	0.52557774	1.31E-39	postive
ALOX12	AL513550.1	0.51779132	2.66E-38	postive
ALOX12	AC093110.1	0.57233493	3.17E-48	postive
ALOX12	AL513327.2	0.5189608	1.70E-38	postive
ACSBG2	AC104984.5	0.55092866	4.12E-44	postive
ELOVL2	AC011603.2	0.82244413	1.13E-133	postive
ACSBG2	AC011603.2	0.68417487	1.22E-75	postive
CYP4B1	AC011603.2	0.88139741	4.83E-177	postive
FASN	AC011603.2	0.81696556	1.77E-130	postive
LGALS1	AGAP2-AS1	0.6387845	3.89E-63	postive
ALOX12	LINC00539	0.56366221	1.60E-46	postive
ACOT8	AC006942.1	0.52996701	2.31E-40	postive
ACOT6	AL021154.1	0.6238815	1.77E-59	postive
BMPR1B	AL021154.1	0.69481935	6.56E-79	postive
RDH11	AL021154.1	0.50685626	1.60E-36	postive

ACACB	AL021154.1	0.52256942	4.23E-39	postive
HSD17B3	AL139123.1	0.53865098	6.96E-42	postive
CPT1B	AL139123.1	0.65755204	4.82E-68	postive
D2HGDH	AL139123.1	0.55357637	1.32E-44	postive
ALOX12	AL139123.1	0.5534114	1.42E-44	postive
ALOX12	AC026471.1	0.52851675	4.11E-40	postive
ELOVL2	AC104806.2	0.73809643	7.92E-94	postive
ACSBG2	AC104806.2	0.69109784	9.47E-78	postive
CYP4B1	AC104806.2	0.79144305	6.83E-117	postive
FASN	AC104806.2	0.69852518	4.42E-80	postive
PRKAG2	AC006547.1	0.60605528	2.37E-55	postive
GABARAPL1	AC006547.1	0.58392086	1.40E-50	postive
ACACB	AC006547.1	0.61524369	1.91E-57	postive
ELOVL2	AC138811.1	0.8270784	1.83E-136	postive
ACSBG2	AC138811.1	0.6732608	1.98E-72	postive
CYP4B1	AC138811.1	0.8881529	1.82E-183	postive
FASN	AC138811.1	0.82264517	8.59E-134	postive
AUH	AC243964.3	0.50589199	2.29E-36	postive
BMPR1B	AC243964.3	0.60784117	9.38E-56	postive
ELOVL4	AC243964.3	0.61303145	6.18E-57	postive
RDH11	AC243964.3	0.52639183	9.50E-40	postive
GABARAPL1	AC243964.3	0.5814664	4.50E-50	postive

ACACB	AC243964.3	0.51096331	3.50E-37	postive
ALOX12	AC092653.1	0.52786786	5.31E-40	postive
ELOVL2	AC015849.1	0.7673206	1.07E-105	postive
ACSBG2	AC015849.1	0.68477012	8.09E-76	postive
CYP4B1	AC015849.1	0.80180061	3.64E-122	postive
FASN	AC015849.1	0.75500595	1.72E-100	postive
ALOX12	AC004263.1	0.61407953	3.55E-57	postive
ENO3	AC131097.2	0.51469088	8.63E-38	postive
ELOVL2	LINC02803	0.78557047	4.93E-114	postive
ACSBG2	LINC02803	0.68861545	5.50E-77	postive
CYP4B1	LINC02803	0.82836354	2.98E-137	postive
FASN	LINC02803	0.77105557	2.44E-107	postive
HSD17B3	AC095057.3	0.6629507	1.61E-69	postive
CPT1B	AC095057.3	0.51874471	1.85E-38	postive
PTGS2	PACERR	0.56335239	1.84E-46	postive
ACOT6	AC211433.1	0.63562867	2.41E-62	postive
ELOVL2	AC073517.1	0.65683238	7.55E-68	postive
ACSBG2	AC073517.1	0.68981717	2.35E-77	postive
CYP4B1	AC073517.1	0.69974415	1.80E-80	postive
FASN	AC073517.1	0.65050541	3.68E-66	postive
ELOVL2	AL359697.1	0.68503326	6.74E-76	postive

ACSBG2	AL359697.1	0.69000255	2.06E-77	postive
CYP4B1	AL359697.1	0.75779065	1.22E-101	postive
FASN	AL359697.1	0.65854591	2.59E-68	postive
ALOX12	LINC02649	0.59287101	1.82E-52	postive
ACOT6	AC040174.2	0.84352037	4.45E-147	postive
BMPR1B	AC040174.2	0.77074967	3.33E-107	postive
PRKAG2	NCKAP5-AS2	0.63078025	3.80E-61	postive
ACOT8	NCKAP5-AS2	0.66497772	4.40E-70	postive
SDHD	NCKAP5-AS2	0.62210835	4.69E-59	postive
PHYH	NCKAP5-AS2	0.54252843	1.41E-42	postive
NDUFAB1	NCKAP5-AS2	0.5172631	3.25E-38	postive
HACD3	NCKAP5-AS2	0.65833371	2.96E-68	postive
AUH	NCKAP5-AS2	0.53563456	2.38E-41	postive
HACL1	NCKAP5-AS2	0.54880681	1.01E-43	postive
CPT1B	AC144548.1	0.50014806	1.85E-35	postive
ALOX12	AC144548.1	0.59325074	1.51E-52	postive
ACOT6	AC130895.1	0.61809681	4.14E-58	postive
ACOT6	AC027348.1	0.6439382	1.89E-64	postive
BMPR1B	AC027348.1	0.51952709	1.37E-38	postive
ALOX12	AC138150.1	0.50732748	1.35E-36	postive
ELOVL2	AC090559.2	0.80547867	4.10E-124	postive

ACSBG2	AC090559.2	0.68954016	2.86E-77	postive
CYP4B1	AC090559.2	0.86985793	6.29E-167	postive
FASN	AC090559.2	0.79542264	6.99E-119	postive
ACOT6	AC011815.1	0.58386875	1.43E-50	postive
BMPR1B	AC011815.1	0.50935754	6.36E-37	postive
CBR4	AC011815.1	0.549442	7.75E-44	postive
SUCLG1	LINC01928	0.52824989	4.57E-40	postive
ACOT6	OPA1-AS1	0.77150293	1.54E-107	postive
BMPR1B	OPA1-AS1	0.5132618	1.48E-37	postive
ELOVL2	PRC1-AS1	0.59811854	1.34E-53	postive
ALOX12	PRC1-AS1	0.52396453	2.46E-39	postive
ACSBG2	PRC1-AS1	0.68331767	2.21E-75	postive
CYP4B1	PRC1-AS1	0.63463529	4.25E-62	postive
FASN	PRC1-AS1	0.57505576	9.05E-49	postive
D2HGDH	CTD-3080P12.3	0.51231013	2.11E-37	postive
HSD17B3	AL158212.2	0.52218866	4.90E-39	postive
CPT1B	AL158212.2	0.60515559	3.76E-55	postive
D2HGDH	AL158212.2	0.53829422	8.05E-42	postive
ALOX12	PSPC1-AS2	0.55177615	2.87E-44	postive
ALOX12	AL354977.1	0.57543368	7.59E-49	postive
ACSBG2	AL354977.1	0.57902579	1.42E-49	postive

ALOX12	LINC00921	0.63842972	4.78E-63	postive
ACSBG2	AC006504.1	0.52418565	2.25E-39	postive
ACOT6	AC006504.1	0.53156049	1.23E-40	postive
ACOT6	SHANK2-AS2	0.54522699	4.58E-43	postive
ACOT6	AC009570.1	0.5296857	2.59E-40	postive
BMPR1B	AC009570.1	0.535702	2.31E-41	postive
RDH11	AC009570.1	0.52915642	3.19E-40	postive
GABARAPL1	AC009570.1	0.50456621	3.72E-36	postive
ELOVL2	AP001160.4	0.56491132	9.16E-47	postive
ACSBG2	AP001160.4	0.67214127	4.16E-72	postive
CYP4B1	AP001160.4	0.60399986	6.81E-55	postive
FASN	AP001160.4	0.5344519	3.84E-41	postive
ALOX12	RFX3-AS1	0.63680696	1.22E-62	postive
ALOX12	PSORS1C3	0.54698626	2.19E-43	postive
ALOX12	AC006017.1	0.5412107	2.43E-42	postive
HACL1	KIF9-AS1	0.50244919	8.03E-36	postive
GABARAPL1	KIF9-AS1	0.60741827	1.17E-55	postive
ACACB	KIF9-AS1	0.61283928	6.84E-57	postive
PTGS2	MIAT	0.55913263	1.18E-45	postive
ACSBG2	MIRLET7BHG	0.5080405	1.04E-36	postive
ELOVL2	SIM1-AS1	0.55265546	1.97E-44	postive
CYP4B1	SIM1-AS1	0.6066456	1.74E-55	postive
ACOT6	SIM1-AS1	0.72342792	1.88E-88	postive

BMPR1B	SIM1-AS1	0.53196721	1.04E-40	postive
FASN	SIM1-AS1	0.53498871	3.09E-41	postive
CPT1B	YEATS2-AS1	0.55590921	4.83E-45	postive
ALOX12	YEATS2-AS1	0.65966886	1.28E-68	postive
ACOT6	AP005482.2	0.56068962	5.97E-46	postive
ALOX12	AL135791.1	0.59884967	9.30E-54	postive
ALOX12	AC023825.2	0.50526492	2.88E-36	postive
ACSBG2	AC023825.2	0.54638732	2.81E-43	postive
ACOT6	AC009087.1	0.59177985	3.12E-52	postive
ACOT6	AC090971.2	0.76314509	6.77E-104	postive
ELOVL2	AC011471.2	0.83044182	1.53E-138	postive
ACSBG2	AC011471.2	0.69790466	6.96E-80	postive
CYP4B1	AC011471.2	0.88917647	1.78E-184	postive
FASN	AC011471.2	0.82096581	8.44E-133	postive
ALOX12	LINC02097	0.55469986	8.15E-45	postive
CPT1B	AP003096.1	0.51821895	2.26E-38	postive
ALOX12	AP003096.1	0.574064	1.43E-48	postive
ACOT6	ITGA6-AS1	0.73919327	3.04E-94	postive
BMPR1B	ITGA6-AS1	0.50740282	1.31E-36	postive
ALOX12	AC010998.2	0.713835	4.02E-85	postive
ALOX12	AL133406.2	0.56408901	1.32E-46	postive

ELOVL2	RN7SL832P	0.80980738	1.84E-126	postive
ACSBG2	RN7SL832P	0.65457568	3.05E-67	postive
CYP4B1	RN7SL832P	0.86869549	5.80E-166	postive
FASN	RN7SL832P	0.80649533	1.17E-124	postive
CPT1B	AC004034.1	0.5449303	5.18E-43	postive
ALOX12	AC004034.1	0.57516265	8.61E-49	postive
ACOT6	AL353699.1	0.83655438	1.95E-142	postive
BMPR1B	AL353699.1	0.94440064	1.42E-261	postive
RDH11	AL353699.1	0.58850593	1.54E-51	postive
ELOVL2	AF131215.4	0.54123059	2.41E-42	postive
ACSBG2	AF131215.4	0.54351295	9.36E-43	postive
CYP4B1	AF131215.4	0.60196648	1.92E-54	postive
ACOT6	AF131215.4	0.64123813	9.28E-64	postive
FASN	AF131215.4	0.52410076	2.33E-39	postive
ELOVL2	AC068888.2	0.78084009	8.53E-112	postive
ACSBG2	AC068888.2	0.71050076	5.37E-84	postive
CYP4B1	AC068888.2	0.84228524	3.08E-146	postive
FASN	AC068888.2	0.76542019	7.15E-105	postive
CPT1B	AC010326.3	0.60904732	5.01E-56	postive
ALOX12	AC010326.3	0.52627848	9.93E-40	postive
ACSBG2	AC007938.3	0.51036612	4.37E-37	postive

GABARAPL1	AC144831.1	0.57755191	2.83E-49	postive
ACACB	AC144831.1	0.51061237	3.99E-37	postive
KMT5A	SNHG4	0.52193631	5.40E-39	postive
D2HGDH	ZEB2-AS1	0.58587149	5.50E-51	postive
ALOX12	ZEB2-AS1	0.57848843	1.83E-49	postive
ELOVL2	UFL1-AS1	0.82240261	1.20E-133	postive
ACSBG2	UFL1-AS1	0.69350941	1.69E-78	postive
CYP4B1	UFL1-AS1	0.8826869	3.08E-178	postive
FASN	UFL1-AS1	0.80644041	1.25E-124	postive
ELOVL2	AC084357.2	0.58751038	2.49E-51	postive
ACSBG2	AC084357.2	0.66313571	1.43E-69	postive
CYP4B1	AC084357.2	0.61901998	2.51E-58	postive
FASN	AC084357.2	0.58378076	1.50E-50	postive
CPT1B	AP000442.1	0.65523464	2.03E-67	postive
D2HGDH	AP000442.1	0.50471032	3.53E-36	postive
ALOX12	AP000442.1	0.58809551	1.88E-51	postive
ALOX12	MIR194-2HG	0.52281145	3.85E-39	postive
ACSBG2	MIR194-2HG	0.54724181	1.96E-43	postive
ELOVL2	STK32A-AS1	0.65714542	6.21E-68	postive
ACSBG2	STK32A-AS1	0.55675866	3.34E-45	postive
CYP4B1	STK32A-AS1	0.69491122	6.14E-79	postive

FASN	STK32A-AS1	0.65015438	4.56E-66	postive
CYP4B1	LINC01220	0.52955947	2.72E-40	postive
ELOVL2	AC093462.1	0.76931283	1.44E-106	postive
ACSBG2	AC093462.1	0.6808966	1.16E-74	postive
CYP4B1	AC093462.1	0.83739767	5.49E-143	postive
FASN	AC093462.1	0.75813378	8.77E-102	postive
HSD17B3	LINC01311	0.53466445	3.52E-41	postive
CPT1B	LINC01311	0.69329076	1.97E-78	postive
D2HGDH	LINC01311	0.59922649	7.69E-54	postive
ALOX12	AL513327.1	0.61068994	2.12E-56	postive
ALOX12	HEXD-IT1	0.71270185	9.75E-85	postive
ACSBG2	HEXD-IT1	0.62529301	8.14E-60	postive
ELOVL2	AC108134.2	0.59287755	1.82E-52	postive
ACSBG2	AC108134.2	0.57826421	2.03E-49	postive
CYP4B1	AC108134.2	0.66690916	1.27E-70	postive
FASN	AC108134.2	0.62006567	1.43E-58	postive
ELOVL2	AC048382.1	0.56682675	3.88E-47	postive
ALOX12	AC048382.1	0.61387842	3.95E-57	postive
ACSBG2	AC048382.1	0.68535072	5.40E-76	postive
CYP4B1	AC048382.1	0.62454994	1.23E-59	postive
FASN	AC048382.1	0.56623242	5.06E-47	postive

HSD17B3	AC010761.1	0.52026485	1.03E-38	postive
ALOX12	AC010761.1	0.5453189	4.40E-43	postive
ACAA2	AC073346.1	0.55550739	5.75E-45	postive
ALDH3A2	AC073346.1	0.50298607	6.61E-36	postive
SLC27A2	AC073346.1	0.54789534	1.49E-43	postive
ALOX12	LINC01237	0.60015156	4.82E-54	postive
ALOX12	PLCG1-AS1	0.53876636	6.64E-42	postive
ELOVL2	AC099518.1	0.80635125	1.40E-124	postive
ACSBG2	AC099518.1	0.63634308	1.60E-62	postive
CYP4B1	AC099518.1	0.84010141	9.00E-145	postive
FASN	AC099518.1	0.80689661	7.09E-125	postive
ALOX12	NEAT1	0.51405712	1.10E-37	postive
ACOT6	NEAT1	0.55265117	1.97E-44	postive
ALOX12	FAM87B	0.50725158	1.39E-36	postive
CPT1B	AC105020.6	0.52777345	5.51E-40	postive
D2HGDH	AC105020.6	0.59197519	2.83E-52	postive
ALOX12	AC105020.6	0.65908208	1.85E-68	postive
PRKAG2	ATP6V0E2-AS1	0.55802751	1.92E-45	postive
PDHB	ATP6V0E2-AS1	0.53554586	2.46E-41	postive
GCDH	ATP6V0E2-AS1	0.53175812	1.13E-40	postive
HACL1	ATP6V0E2-AS1	0.52878765	3.69E-40	postive

RDH11	ATP6V0E2-AS1	0.56990124	9.64E-48	postive
GABARAPL1	ATP6V0E2-AS1	0.53771945	1.02E-41	postive
ACACB	ATP6V0E2-AS1	0.54758233	1.70E-43	postive
ACSBG2	AC087284.1	0.51650258	4.34E-38	postive
ELOVL2	LINC01397	0.83071862	1.03E-138	postive
ACSBG2	LINC01397	0.65326301	6.85E-67	postive
CYP4B1	LINC01397	0.89015276	1.90E-185	postive
FASN	LINC01397	0.82876497	1.69E-137	postive
ELOVL4	AC023794.2	0.52392178	2.50E-39	postive
ELOVL2	AC002128.2	0.60814435	8.02E-56	postive
ACSBG2	AC002128.2	0.68431942	1.11E-75	postive
CYP4B1	AC002128.2	0.66745078	8.93E-71	postive
FASN	AC002128.2	0.59940991	7.01E-54	postive
HSD17B3	FSIP2-AS2	0.52941834	2.88E-40	postive
D2HGDH	AP001062.1	0.561622	3.95E-46	postive
FAAH	CNNM3-DT	0.55354583	1.34E-44	postive
BMPR1B	AL138756.1	0.6109771	1.82E-56	postive
ELOVL4	AL138756.1	0.50794913	1.07E-36	postive
RDH11	AL138756.1	0.51591203	5.43E-38	postive
GABARAPL1	AL138756.1	0.62686883	3.40E-60	postive
ACACB	AL138756.1	0.55123899	3.60E-44	postive

CPT1B	AL138921.1	0.50092163	1.40E-35	postive
ALOX12	AL138921.1	0.63852661	4.52E-63	postive
ALOX12	AC016747.3	0.5227432	3.95E-39	postive
ELOVL2	AC005546.1	0.69766385	8.30E-80	postive
ACSBG2	AC005546.1	0.71605277	7.03E-86	postive
CYP4B1	AC005546.1	0.74155042	3.80E-95	postive
FASN	AC005546.1	0.68939173	3.18E-77	postive
CPT1B	SLC25A25-AS1	0.67824063	7.08E-74	postive
BMPR1B	SLC25A25-AS1	0.51694008	3.68E-38	postive
ACACB	SLC25A25-AS1	0.58355552	1.67E-50	postive
ELOVL2	AC127070.2	0.59033738	6.32E-52	postive
ACSBG2	AC127070.2	0.56818001	2.10E-47	postive
CYP4B1	AC127070.2	0.58620065	4.69E-51	postive
FASN	AC127070.2	0.51467485	8.68E-38	postive
PRKAG2	AC019197.1	0.55430191	9.68E-45	postive
SDHD	AC019197.1	0.50954978	5.92E-37	postive
HACD3	AC019197.1	0.62371051	1.95E-59	postive
AUH	AC019197.1	0.57945284	1.16E-49	postive
BMPR1B	AC019197.1	0.52266737	4.07E-39	postive
ELOVL4	AC019197.1	0.51974663	1.26E-38	postive
GABARAPL1	AC019197.1	0.64826652	1.43E-65	postive

ACACB	AC019197.1	0.59101051	4.55E-52	postive
ELOVL2	AC078980.1	0.82888206	1.43E-137	postive
ACSBG2	AC078980.1	0.64791571	1.76E-65	postive
CYP4B1	AC078980.1	0.89169808	5.26E-187	postive
FASN	AC078980.1	0.82755068	9.42E-137	postive
ACOT6	AC007546.1	0.58692468	3.31E-51	postive
ALOX12	AC012358.1	0.51560828	6.10E-38	postive
ALOX12	AC027097.1	0.5468506	2.32E-43	postive
PHYH	HORMAD2-AS1	0.51605872	5.14E-38	postive
HACL1	HORMAD2-AS1	0.54151437	2.14E-42	postive
PON1	HORMAD2-AS1	0.64919868	8.13E-66	postive
ADH1A	HORMAD2-AS1	0.64928936	7.70E-66	postive
ADH4	HORMAD2-AS1	0.65449197	3.22E-67	postive
FABP1	HORMAD2-AS1	0.60063371	3.78E-54	postive
ADH1C	HORMAD2-AS1	0.71123554	3.05E-84	postive
CYP1A2	HORMAD2-AS1	0.56110581	4.97E-46	postive
RDH16	HORMAD2-AS1	0.64741845	2.37E-65	postive
ADH1B	HORMAD2-AS1	0.58251148	2.74E-50	postive
ELOVL2	AC067750.1	0.54942764	7.79E-44	postive
ACSBG2	AC067750.1	0.54601908	3.28E-43	postive
CYP4B1	AC067750.1	0.57571718	6.66E-49	postive

FASN	AC067750.1	0.56939856	1.21E-47	postive
LGALS1	AC026401.3	0.51514173	7.28E-38	postive
KMT5A	AC026401.3	0.562356	2.86E-46	postive
ALOX12	AC015853.1	0.60331695	9.65E-55	postive
ACSBG2	AC015853.1	0.54970518	6.93E-44	postive
ELOVL2	AL133371.3	0.83178107	2.21E-139	postive
ACSBG2	AL133371.3	0.64832818	1.37E-65	postive
CYP4B1	AL133371.3	0.89137243	1.12E-186	postive
FASN	AL133371.3	0.82768855	7.76E-137	postive
ALOX12	MANEA-DT	0.65665051	8.45E-68	postive
ACSBG2	AL359962.2	0.57988552	9.48E-50	postive
LGALS1	AC147067.1	0.53254576	8.26E-41	postive
HSD17B3	PRANCR	0.60594861	2.50E-55	postive
ALOX12	HCG18	0.52892156	3.50E-40	postive
ACOT8	AC135507.1	0.61677553	8.41E-58	postive
SDHD	AC135507.1	0.59454246	7.98E-53	postive
PHYH	AC135507.1	0.51553832	6.26E-38	postive
HACD3	AC135507.1	0.61079102	2.01E-56	postive
ELOVL4	AC135507.1	0.68083895	1.21E-74	postive
ELOVL2	AC009950.1	0.59156842	3.46E-52	postive
ALOX12	AC009950.1	0.57144993	4.76E-48	postive

ACSBG2	AC009950.1	0.64006192	1.85E-63	postive
CYP4B1	AC009950.1	0.63741922	8.58E-63	postive
FASN	AC009950.1	0.56999697	9.23E-48	postive
ELOVL2	AC132192.1	0.65603401	1.24E-67	postive
ACSBG2	AC132192.1	0.65932634	1.59E-68	postive
CYP4B1	AC132192.1	0.68959642	2.75E-77	postive
FASN	AC132192.1	0.62864996	1.26E-60	postive
ELOVL2	LINC02550	0.66644524	1.71E-70	postive
CYP4B1	LINC02550	0.68675629	2.03E-76	postive
FASN	LINC02550	0.67843681	6.20E-74	postive
SUCLG1	CEROX1	0.53981257	4.32E-42	postive
CPT1B	AC092143.3	0.68342	2.06E-75	postive
D2HGDH	AC092143.3	0.53949795	4.92E-42	postive
ALOX12	AC092143.3	0.51463811	8.80E-38	postive
HSD17B3	AC073655.2	0.60436253	5.65E-55	postive
ALOX12	AC073655.2	0.52277045	3.91E-39	postive
CPT1B	AC025287.3	0.50414857	4.33E-36	postive
ALOX12	AC025287.3	0.67761505	1.08E-73	postive
ACACB	ZKSCAN7-AS1	0.51351261	1.35E-37	postive
ACACB	AC009093.4	0.53248508	8.46E-41	postive
ALOX12	AP002812.2	0.66688107	1.29E-70	postive

ACSBG2	AP002812.2	0.53770614	1.02E-41	postive
ALOX12	AC005954.1	0.63797214	6.23E-63	postive
ELOVL2	AC009955.3	0.82372341	1.96E-134	postive
ACSBG2	AC009955.3	0.64665846	3.74E-65	postive
CYP4B1	AC009955.3	0.82151428	4.01E-133	postive
FASN	AC009955.3	0.78095994	7.50E-112	postive
ALOX12	AC048341.1	0.51678482	3.90E-38	postive
ELOVL2	TMED2-DT	0.50781975	1.12E-36	postive
CPT1B	TMED2-DT	0.51214832	2.25E-37	postive
D2HGDH	TMED2-DT	0.50006875	1.90E-35	postive
ALOX12	TMED2-DT	0.67505075	6.03E-73	postive
ACSBG2	TMED2-DT	0.70013876	1.35E-80	postive
CYP4B1	TMED2-DT	0.55540618	6.01E-45	postive
FASN	TMED2-DT	0.52821812	4.63E-40	postive
ACSBG1	AATBC	0.56963414	1.09E-47	postive
ENO3	AATBC	0.67479875	7.14E-73	postive
PCBD1	AATBC	0.53519341	2.84E-41	postive
ELOVL2	AC002398.1	0.6433301	2.71E-64	postive
HSD17B3	AC002398.1	0.50614824	2.08E-36	postive
ACSBG2	AC002398.1	0.60599319	2.44E-55	postive
CYP4B1	AC002398.1	0.67893005	4.44E-74	postive

FASN	AC002398.1	0.65817197	3.28E-68	postive
GABARAPL1	AC025154.2	0.60135135	2.63E-54	postive
ELOVL2	AC023813.3	0.81118743	3.20E-127	postive
ACSBG2	AC023813.3	0.66564395	2.87E-70	postive
CYP4B1	AC023813.3	0.86528618	3.48E-163	postive
FASN	AC023813.3	0.80789729	2.04E-125	postive
ACSBG2	AC008781.2	0.55684669	3.21E-45	postive
ACAA2	AP001542.3	0.57593953	6.00E-49	postive
ALDH3A2	AP001542.3	0.51891632	1.73E-38	postive
ELOVL2	AC010149.1	0.59745525	1.87E-53	postive
ACSBG2	AC010149.1	0.66483854	4.81E-70	postive
CYP4B1	AC010149.1	0.64268539	3.96E-64	postive
FASN	AC010149.1	0.5850587	8.12E-51	postive
ELOVL2	AC012653.2	0.72142692	9.55E-88	postive
ACSBG2	AC012653.2	0.58176164	3.91E-50	postive
CYP4B1	AC012653.2	0.77526029	3.16E-109	postive
FASN	AC012653.2	0.7240111	1.17E-88	postive
ELOVL2	C6orf99	0.76864196	2.83E-106	postive
ACSBG2	C6orf99	0.61719017	6.74E-58	postive
CYP4B1	C6orf99	0.8321192	1.36E-139	postive
FASN	C6orf99	0.78828538	2.42E-115	postive
PHYH	INSIG1-DT	0.50043363	1.67E-35	postive

ELOVL4	INSIG1-DT	0.54839789	1.21E-43	postive
GABARAPL1	INSIG1-DT	0.59471372	7.33E-53	postive
ELOVL2	AC109597.2	0.74644385	4.74E-97	postive
ACSBG2	AC109597.2	0.71496297	1.66E-85	postive
CYP4B1	AC109597.2	0.80558512	3.60E-124	postive
FASN	AC109597.2	0.74359806	6.14E-96	postive
ALOX12	AP001001.1	0.51207681	2.31E-37	postive
ACOT6	AP001001.1	0.63526747	2.96E-62	postive
DPEP2	LINC01857	0.61243441	8.47E-57	postive
ALOX12	AC016747.2	0.68306592	2.63E-75	postive
ACSBG2	AC016747.2	0.59588131	4.11E-53	postive
DPEP2	AC002091.2	0.505998	2.20E-36	postive
TBXAS1	AC002091.2	0.58530394	7.22E-51	postive
ACOT6	AC008147.2	0.90378994	4.50E-200	postive
BMPR1B	AC008147.2	0.70395777	7.83E-82	postive
CPT1B	PSMA3-AS1	0.57539328	7.74E-49	postive
D2HGDH	PSMA3-AS1	0.52930703	3.01E-40	postive
ALOX12	PSMA3-AS1	0.66393285	8.59E-70	postive
ALOX12	AL590133.1	0.66344313	1.17E-69	postive
ALOX12	CKMT2-AS1	0.55873084	1.41E-45	postive
ACSBG2	CKMT2-AS1	0.55189506	2.72E-44	postive

ELOVL2	LCMT1-AS1	0.62404846	1.62E-59	postive
ACSBG2	LCMT1-AS1	0.59175013	3.16E-52	postive
CYP4B1	LCMT1-AS1	0.66724889	1.02E-70	postive
FASN	LCMT1-AS1	0.65142682	2.10E-66	postive
ALOX12	AC145207.8	0.62048775	1.13E-58	postive
ACSBG2	AC145207.8	0.55580745	5.05E-45	postive
ELOVL2	AL118511.1	0.61170893	1.24E-56	postive
CYP4B1	AL118511.1	0.57403017	1.45E-48	postive
FASN	AL118511.1	0.66118093	4.94E-69	postive
ELOVL2	LINC01635	0.71731696	2.58E-86	postive
ACSBG2	LINC01635	0.62622674	4.86E-60	postive
CYP4B1	LINC01635	0.75181397	3.42E-99	postive
FASN	LINC01635	0.70004901	1.44E-80	postive
CPT1B	AL031600.1	0.66278093	1.79E-69	postive
D2HGDH	AL031600.1	0.51171117	2.65E-37	postive
ALOX12	AL031600.1	0.63370757	7.22E-62	postive
PRKAG2	PRDM16-DT	0.56032041	7.02E-46	postive
PDHB	PRDM16-DT	0.54214436	1.65E-42	postive
AUH	PRDM16-DT	0.50802391	1.04E-36	postive
ELOVL4	PRDM16-DT	0.55671681	3.40E-45	postive

RDH11	PRDM16-DT	0.58877378	1.35E-51	postive
GABARAPL1	PRDM16-DT	0.72332754	2.04E-88	postive
ACACB	PRDM16-DT	0.5852119	7.55E-51	postive
CPT1B	AL161452.1	0.62994264	6.09E-61	postive
D2HGDH	AL161452.1	0.58386103	1.44E-50	postive
PRKAG2	LINC00323	0.59269121	1.99E-52	postive
SDHD	LINC00323	0.68853081	5.84E-77	postive
PHYH	LINC00323	0.66366809	1.02E-69	postive
PDHB	LINC00323	0.5269809	7.54E-40	postive
NDUFAB1	LINC00323	0.54087177	2.79E-42	postive
HACD3	LINC00323	0.72575004	2.79E-89	postive
AUH	LINC00323	0.53593302	2.11E-41	postive
HACL1	LINC00323	0.50046413	1.65E-35	postive
ELOVL4	LINC00323	0.74455241	2.61E-96	postive
GABARAPL1	LINC00323	0.5325034	8.40E-41	postive
ALOX12	RNF213-AS1	0.66385995	9.00E-70	postive
ACOT8	LINC01532	0.53502129	3.05E-41	postive
SDHD	LINC01532	0.69128886	8.27E-78	postive
PHYH	LINC01532	0.72759659	6.05E-90	postive
NDUFAB1	LINC01532	0.61671524	8.69E-58	postive
HACD3	LINC01532	0.69906205	2.98E-80	postive

ELOVL4	LINC01532	0.81239027	6.86E-128	postive
ELOVL2	WWTR1-IT1	0.62132923	7.18E-59	postive
ACSBG2	WWTR1-IT1	0.64902525	9.03E-66	postive
CYP4B1	WWTR1-IT1	0.68302725	2.70E-75	postive
FASN	WWTR1-IT1	0.59754591	1.79E-53	postive
ELOVL2	AC245297.2	0.67244263	3.41E-72	postive
ACSBG2	AC245297.2	0.64793083	1.75E-65	postive
CYP4B1	AC245297.2	0.70301466	1.59E-81	postive
FASN	AC245297.2	0.63888158	3.67E-63	postive
ALOX12	AC078846.1	0.61107624	1.73E-56	postive
ELOVL2	AL360157.1	0.82893371	1.33E-137	postive
ACSBG2	AL360157.1	0.63683325	1.20E-62	postive
CYP4B1	AL360157.1	0.89344325	8.57E-189	postive
FASN	AL360157.1	0.82806817	4.53E-137	postive
ELOVL4	LMO7-AS1	0.67405295	1.17E-72	postive
ACOT6	AC105429.1	0.78214575	2.08E-112	postive
BMPR1B	AC105429.1	0.53545808	2.55E-41	postive
ELOVL2	AC027279.1	0.69742545	9.88E-80	postive
ACSBG2	AC027279.1	0.68945945	3.03E-77	postive
CYP4B1	AC027279.1	0.74151531	3.92E-95	postive

FASN	AC027279.1	0.65832011	2.99E-68	postive
ELOVL2	AC090844.2	0.52491765	1.69E-39	postive
ALOX12	AC090844.2	0.52823354	4.60E-40	postive
ACSBG2	AC090844.2	0.67746601	1.19E-73	postive
CYP4B1	AC090844.2	0.57447869	1.18E-48	postive
FASN	AC090844.2	0.50647954	1.84E-36	postive
ELOVL2	AC009630.1	0.66837667	4.90E-71	postive
CYP4B1	AC009630.1	0.64954937	6.58E-66	postive
FASN	AC009630.1	0.5805733	6.86E-50	postive
ALOX12	AL157786.1	0.55465343	8.32E-45	postive
ELOVL2	AC087645.2	0.53152983	1.24E-40	postive
FASN	AC087645.2	0.59027654	6.51E-52	postive
ALOX12	AC010531.3	0.56689829	3.75E-47	postive
ACSBG2	AC010531.3	0.51052643	4.12E-37	postive
ELOVL2	AL139274.2	0.70500033	3.57E-82	postive
ACSBG2	AL139274.2	0.70307233	1.52E-81	postive
CYP4B1	AL139274.2	0.75609502	6.13E-101	postive
FASN	AL139274.2	0.6763149	2.59E-73	postive
CPT1B	ZNF32-AS2	0.59635082	3.25E-53	postive
D2HGDH	ZNF32-AS2	0.53902897	5.96E-42	postive
ALOX12	ZNF32-AS2	0.66711973	1.11E-70	postive
ACOT6	AC009032.1	0.8814136	4.66E-177	postive

BMPR1B	AC009032.1	0.65505543	2.27E-67	postive
CPT1B	AL021707.1	0.58208554	3.35E-50	postive
D2HGDH	AL021707.1	0.52741165	6.36E-40	postive
ALOX12	AL021707.1	0.56486826	9.33E-47	postive
ACSBG2	AC018462.1	0.58296134	2.21E-50	postive
CYP4B1	AC018462.1	0.51686959	3.78E-38	postive
ALOX12	GAS8-AS1	0.69285733	2.70E-78	postive
ACSBG2	GAS8-AS1	0.58856074	1.50E-51	postive
ACSBG2	AC104794.2	0.54012745	3.80E-42	postive
ELOVL2	AL121890.4	0.61066852	2.15E-56	postive
ALOX12	AL121890.4	0.53284503	7.32E-41	postive
ACSBG2	AL121890.4	0.63639957	1.54E-62	postive
CYP4B1	AL121890.4	0.63668745	1.31E-62	postive
FASN	AL121890.4	0.5902056	6.74E-52	postive
ELOVL2	LINC02541	0.51840125	2.10E-38	postive
HSD17B3	AC055855.1	0.5170718	3.50E-38	postive
CPT1B	AC055855.1	0.62892354	1.08E-60	postive
D2HGDH	AC055855.1	0.55684556	3.22E-45	postive
ALOX12	AC055855.1	0.61161689	1.30E-56	postive
ELOVL2	AC099850.1	0.61160193	1.31E-56	postive
CYP4B1	AC099850.1	0.63018473	5.32E-61	postive

FASN	AC099850.1	0.55111974	3.79E-44	postive
SDHD	LINC01612	0.68403032	1.35E-75	postive
PHYH	LINC01612	0.7231849	2.29E-88	postive
PDHB	LINC01612	0.51911069	1.60E-38	postive
NDUFAB1	LINC01612	0.55446366	9.03E-45	postive
HACD3	LINC01612	0.74321648	8.64E-96	postive
AUH	LINC01612	0.57466951	1.08E-48	postive
HACL1	LINC01612	0.53948525	4.94E-42	postive
ELOVL4	LINC01612	0.76242795	1.37E-103	postive
RDH11	LINC01612	0.56029254	7.11E-46	postive
GABARAPL1	LINC01612	0.69327881	1.99E-78	postive
ALOX12	FLJ31104	0.52629438	9.87E-40	postive
ACSBG2	FLJ31104	0.58751281	2.49E-51	postive
HSD17B3	ZNF32-AS1	0.62741705	2.50E-60	postive
CPT1B	ZNF32-AS1	0.5363672	1.77E-41	postive
CPT1B	AC092119.2	0.70350402	1.10E-81	postive
D2HGDH	AC092119.2	0.60574773	2.77E-55	postive
ALOX12	AC092119.2	0.5939018	1.10E-52	postive
ACOT6	AC097504.2	0.63691429	1.15E-62	postive
BMPR1B	AC097504.2	0.50579595	2.37E-36	postive
CBR4	AC097504.2	0.50512556	3.03E-36	postive
ELOVL2	SLC25A30-AS1	0.75310129	1.03E-99	postive

ACSBG2	SLC25A30-AS1	0.65329653	6.71E-67	postive
CYP4B1	SLC25A30-AS1	0.82352534	2.57E-134	postive
FASN	SLC25A30-AS1	0.75919679	3.16E-102	postive
CPT1B	AL139287.1	0.60685797	1.56E-55	postive
D2HGDH	AL139287.1	0.66877239	3.79E-71	postive
ALOX12	AL139287.1	0.72287721	2.94E-88	postive
ELOVL2	AL121944.1	0.58145814	4.51E-50	postive
ACSBG2	AL121944.1	0.50929105	6.52E-37	postive
CYP4B1	AL121944.1	0.6748238	7.02E-73	postive
FASN	AL121944.1	0.58155402	4.31E-50	postive
ACOT6	NPTN-IT1	0.73584424	5.59E-93	postive
BMPR1B	NPTN-IT1	0.52211577	5.04E-39	postive
ELOVL2	LINC02435	0.81510801	2.03E-129	postive
ACSBG2	LINC02435	0.67271742	2.84E-72	postive
CYP4B1	LINC02435	0.87989594	1.14E-175	postive
FASN	LINC02435	0.81202185	1.10E-127	postive
ALOX12	AC012313.1	0.50037059	1.70E-35	postive
BMPR1B	MMEL1-AS1	0.74306499	9.89E-96	postive
RDH11	MMEL1-AS1	0.65372416	5.16E-67	postive
ACOT6	AL159972.1	0.51033301	4.43E-37	postive
ELOVL2	AC114956.1	0.6732078	2.05E-72	postive

ACSBG2	AC114956.1	0.69541387	4.27E-79	postive
CYP4B1	AC114956.1	0.69156857	6.77E-78	postive
FASN	AC114956.1	0.66838408	4.87E-71	postive
HSD17B3	AC005393.1	0.52449465	2.00E-39	postive
CPT1B	AC005393.1	0.63004754	5.74E-61	postive
PON1	TCL6	0.61185651	1.15E-56	postive
ADH1A	TCL6	0.61161305	1.31E-56	postive
ADH4	TCL6	0.61579345	1.42E-57	postive
FABP1	TCL6	0.60314285	1.06E-54	postive
ADH1C	TCL6	0.64592389	5.81E-65	postive
CYP1A2	TCL6	0.54676792	2.40E-43	postive
RDH16	TCL6	0.60794703	8.88E-56	postive
ADH1B	TCL6	0.53026501	2.05E-40	postive
ACOT6	AL355472.3	0.71126778	2.97E-84	postive
BMPR1B	AL355472.3	0.54912921	8.85E-44	postive
DPEP2	AC012645.3	0.52310239	3.44E-39	postive
CPT1B	AC007066.2	0.57722025	3.31E-49	postive
ALOX12	AC007066.2	0.60050321	4.04E-54	postive
ELOVL2	SLC16A1-AS1	0.57377659	1.63E-48	postive
ALOX12	SLC16A1-AS1	0.56777751	2.52E-47	postive

ACSBG2	SLC16A1-AS1	0.66603638	2.23E-70	postive
CYP4B1	SLC16A1-AS1	0.57148819	4.67E-48	postive
FASN	SLC16A1-AS1	0.54781421	1.54E-43	postive
ELOVL2	Z93241.1	0.61275083	7.17E-57	postive
ACSBG2	Z93241.1	0.56228333	2.95E-46	postive
CYP4B1	Z93241.1	0.61096429	1.84E-56	postive
FASN	Z93241.1	0.57891614	1.50E-49	postive
ACAA2	WDFY3-AS2	0.61668048	8.85E-58	postive
ACBD5	WDFY3-AS2	0.57534512	7.91E-49	postive
EHHADH	WDFY3-AS2	0.63259251	1.36E-61	postive
PRKAA2	WDFY3-AS2	0.61021068	2.73E-56	postive
ADH5	WDFY3-AS2	0.60226367	1.65E-54	postive
CRYZ	WDFY3-AS2	0.69512172	5.28E-79	postive
CBR4	WDFY3-AS2	0.5282885	4.50E-40	postive
ACADM	WDFY3-AS2	0.67859813	5.56E-74	postive
ETFDH	WDFY3-AS2	0.51398522	1.13E-37	postive
SCP2	WDFY3-AS2	0.56646719	4.56E-47	postive
SERINC1	WDFY3-AS2	0.54204614	1.72E-42	postive
HSDL2	WDFY3-AS2	0.50363024	5.23E-36	postive
ACADL	WDFY3-AS2	0.54708859	2.10E-43	postive
ALDH3A2	WDFY3-AS2	0.56588417	5.92E-47	postive

ALDH9A1	WDFY3-AS2	0.55594717	4.75E-45	postive
HIBCH	WDFY3-AS2	0.60344954	9.02E-55	postive
MMAA	WDFY3-AS2	0.5347633	3.38E-41	postive
SLC27A2	WDFY3-AS2	0.61334897	5.22E-57	postive
CPT2	WDFY3-AS2	0.52809721	4.85E-40	postive
CPT1B	AC020931.1	0.62112378	8.03E-59	postive
D2HGDH	AC020931.1	0.54533237	4.38E-43	postive
ALOX12	AC020931.1	0.62682505	3.48E-60	postive
ELOVL2	AC011477.3	0.76729166	1.10E-105	postive
ACSBG2	AC011477.3	0.65602922	1.24E-67	postive
CYP4B1	AC011477.3	0.84824444	2.35E-150	postive
FASN	AC011477.3	0.75704443	2.48E-101	postive
ALOX12	AC010973.1	0.61348694	4.86E-57	postive
ACSBG2	AC010973.1	0.53969179	4.54E-42	postive
ACOT6	AC004943.1	0.56676114	3.99E-47	postive
ALOX12	AC012593.2	0.6116881	1.26E-56	postive
ACBD5	AP001372.2	0.54687849	2.29E-43	postive
PRKAA2	AP001372.2	0.53073039	1.71E-40	postive
SCP2	AP001372.2	0.53529123	2.73E-41	postive
SERINC1	AP001372.2	0.51420375	1.04E-37	postive
ACACB	AC026992.1	0.52572511	1.23E-39	postive
ELOVL2	AP005264.1	0.7495012	2.90E-98	postive

ACSBG2	AP005264.1	0.58509628	7.98E-51	postive
CYP4B1	AP005264.1	0.81834184	2.86E-131	postive
FASN	AP005264.1	0.75605985	6.34E-101	postive
PON1	LINC01485	0.84427825	1.35E-147	postive
ADH1A	LINC01485	0.84302279	9.72E-147	postive
ADH4	LINC01485	0.84344998	4.97E-147	postive
FABP1	LINC01485	0.81816823	3.60E-131	postive
ADH1C	LINC01485	0.83986125	1.30E-144	postive
CYP1A2	LINC01485	0.65048313	3.73E-66	postive
RDH16	LINC01485	0.84408281	1.84E-147	postive
ADH1B	LINC01485	0.77733614	3.57E-110	postive
ELOVL2	JARID2-AS1	0.68056817	1.46E-74	postive
ACSBG2	JARID2-AS1	0.69494761	5.98E-79	postive
CYP4B1	JARID2-AS1	0.74181702	3.00E-95	postive
FASN	JARID2-AS1	0.66473205	5.15E-70	postive
ACOT6	AL139022.1	0.52397207	2.45E-39	postive
ACOT6	AL049555.1	0.5773834	3.07E-49	postive
BMPR1B	AL049555.1	0.74640411	4.91E-97	postive
RDH11	AL049555.1	0.52670688	8.39E-40	postive
GABARAPL1	AL049555.1	0.64236722	4.78E-64	postive
CPT1B	AC090589.3	0.61921216	2.27E-58	postive

D2HGDH	AC090589.3	0.62557565	6.96E-60	postive
ALOX12	AC090589.3	0.73776753	1.05E-93	postive
DPEP2	PCED1B-AS1	0.50646597	1.85E-36	postive
TBXAS1	PCED1B-AS1	0.60876138	5.82E-56	postive
ELOVL2	ASAP1-IT2	0.71990172	3.27E-87	postive
ACSBG2	ASAP1-IT2	0.69645853	2.00E-79	postive
CYP4B1	ASAP1-IT2	0.77369851	1.60E-108	postive
FASN	ASAP1-IT2	0.69009538	1.93E-77	postive
ELOVL2	AC136469.1	0.83456635	3.75E-141	postive
ACSBG2	AC136469.1	0.66788411	6.74E-71	postive
CYP4B1	AC136469.1	0.89625897	9.60E-192	postive
FASN	AC136469.1	0.82770358	7.59E-137	postive
CPT1B	AC005674.1	0.55019689	5.62E-44	postive
D2HGDH	AC005674.1	0.50275834	7.18E-36	postive
ALOX12	AC005674.1	0.52963062	2.64E-40	postive
ACOT6	STX18-AS1	0.64104931	1.04E-63	postive
BMPR1B	STX18-AS1	0.59470761	7.36E-53	postive
CBR4	STX18-AS1	0.56366744	1.60E-46	postive
ACACB	STX18-AS1	0.53275933	7.58E-41	postive
ACOT6	AC108727.1	0.75938936	2.62E-102	postive

BMPR1B	AC108727.1	0.50079135	1.46E-35	postive
CPT1B	AC010319.4	0.52388909	2.53E-39	postive
ALOX12	AC010319.4	0.64744458	2.34E-65	postive
ACSBG2	AC010319.4	0.60575129	2.77E-55	postive
CYP4B1	AC010319.4	0.50296267	6.67E-36	postive
ALOX12	AP4B1-AS1	0.69104734	9.82E-78	postive
ACSBG2	AP4B1-AS1	0.60231522	1.61E-54	postive
ACOT6	AC096741.1	0.67437816	9.44E-73	postive
ELOVL2	AC026471.5	0.5948677	6.80E-53	postive
ACSBG2	AC026471.5	0.62470864	1.12E-59	postive
CYP4B1	AC026471.5	0.64297777	3.34E-64	postive
FASN	AC026471.5	0.57624638	5.21E-49	postive
CPT1B	AC012615.6	0.68297107	2.81E-75	postive
D2HGDH	AC012615.6	0.71971673	3.79E-87	postive
ACADVL	AC012615.6	0.55255468	2.05E-44	postive
ALOX12	AC012615.6	0.7572518	2.04E-101	postive
CPT1B	AC017083.1	0.62812492	1.69E-60	postive
D2HGDH	AC017083.1	0.61091414	1.89E-56	postive
ALOX12	AC017083.1	0.59142421	3.71E-52	postive
ELOVL2	AC131571.1	0.79866065	1.56E-120	postive
ACSBG2	AC131571.1	0.68476353	8.13E-76	postive

CYP4B1	AC131571.1	0.86621857	6.16E-164	postive
FASN	AC131571.1	0.7890407	1.04E-115	postive
ELOVL2	C5orf34-AS1	0.66016588	9.37E-69	postive
ACSBG2	C5orf34-AS1	0.6097107	3.54E-56	postive
CYP4B1	C5orf34-AS1	0.64956727	6.50E-66	postive
FASN	C5orf34-AS1	0.65127064	2.31E-66	postive
ELOVL2	AP001178.1	0.75810901	8.98E-102	postive
ACSBG2	AP001178.1	0.71237745	1.26E-84	postive
CYP4B1	AP001178.1	0.81975235	4.33E-132	postive
FASN	AP001178.1	0.76288656	8.72E-104	postive
ACOT6	ARRDC3-AS1	0.74367191	5.75E-96	postive
BMPR1B	ARRDC3-AS1	0.69164381	6.42E-78	postive
ELOVL2	AL359541.1	0.68948926	2.97E-77	postive
ACSBG2	AL359541.1	0.57349851	1.86E-48	postive
CYP4B1	AL359541.1	0.74318623	8.88E-96	postive
FASN	AL359541.1	0.64062293	1.33E-63	postive
CPT1B	AC008735.4	0.59275715	1.93E-52	postive
ALOX12	AC008735.4	0.61163372	1.29E-56	postive
ALOX12	AC010530.1	0.52385346	2.56E-39	postive
ACSBG2	AC010530.1	0.61288071	6.69E-57	postive
CYP4B1	AC010530.1	0.55437452	9.38E-45	postive

ACOT6	AC010530.1	0.60358017	8.44E-55	postive
ELOVL2	AC023355.1	0.60047737	4.09E-54	postive
ALOX12	AC023355.1	0.63775633	7.06E-63	postive
ACSBG2	AC023355.1	0.69521905	4.92E-79	postive
CYP4B1	AC023355.1	0.66310987	1.45E-69	postive
FASN	AC023355.1	0.57729043	3.20E-49	postive
ELOVL2	AC099684.2	0.51677212	3.92E-38	postive
CYP4B1	AC099684.2	0.60236281	1.57E-54	postive
FASN	AC099684.2	0.51307805	1.58E-37	postive
ACOT6	AL024498.1	0.54348979	9.45E-43	postive
ACOT6	BTG3-AS1	0.52600789	1.10E-39	postive
BMPR1B	BTG3-AS1	0.85508735	2.61E-155	postive
RDH11	BTG3-AS1	0.65753745	4.87E-68	postive
GABARAPL1	BTG3-AS1	0.53496087	3.12E-41	postive
ACACB	BTG3-AS1	0.52071973	8.64E-39	postive
ALOX12	AC025569.1	0.53758078	1.08E-41	postive
SDHD	Z82185.1	0.6465279	4.05E-65	postive
PHYH	Z82185.1	0.67986888	2.35E-74	postive
PDHB	Z82185.1	0.52742113	6.34E-40	postive
NDUFAB1	Z82185.1	0.50515868	2.99E-36	postive
HACD3	Z82185.1	0.69351283	1.68E-78	postive

AUH	Z82185.1	0.66087751	5.98E-69	postive
HACL1	Z82185.1	0.55512744	6.78E-45	postive
BMPR1B	Z82185.1	0.51642021	4.48E-38	postive
ELOVL4	Z82185.1	0.77927486	4.56E-111	postive
RDH11	Z82185.1	0.66203586	2.87E-69	postive
GABARAPL1	Z82185.1	0.69670982	1.67E-79	postive
ACACB	Z82185.1	0.54723153	1.97E-43	postive
CPT1B	AL928654.2	0.60406027	6.60E-55	postive
D2HGDH	AL928654.2	0.58467171	9.78E-51	postive
ALOX12	AL928654.2	0.66205162	2.85E-69	postive
ALOX12	AL022238.2	0.58115162	5.22E-50	postive
ALOX12	AC005332.3	0.5119792	2.39E-37	postive
ELOVL2	AC004233.3	0.77388317	1.33E-108	postive
ACSBG2	AC004233.3	0.65093134	2.84E-66	postive
CYP4B1	AC004233.3	0.84106091	2.06E-145	postive
FASN	AC004233.3	0.77229037	6.87E-108	postive
HSD17B3	AC011462.4	0.54572512	3.72E-43	postive
CPT1B	AC011462.4	0.63308579	1.03E-61	postive
D2HGDH	AC011462.4	0.64063471	1.32E-63	postive
ALOX12	AC011462.4	0.65094594	2.82E-66	postive
HSD17B3	AC087588.1	0.59345473	1.37E-52	postive

CPT1B	TNFRSF14-AS1	0.58150853	4.41E-50	postive
D2HGDH	TNFRSF14-AS1	0.52990308	2.37E-40	postive
ACSBG2	AC009269.2	0.57059785	7.02E-48	postive
CYP4B1	AC009269.2	0.50009758	1.88E-35	postive
ALOX12	AL161669.1	0.54783026	1.53E-43	postive
CPT1B	HPN-AS1	0.55053243	4.87E-44	postive
D2HGDH	HPN-AS1	0.66765206	7.84E-71	postive
ACADVL	HPN-AS1	0.50510711	3.05E-36	postive
ALOX12	HPN-AS1	0.64511187	9.42E-65	postive
ALOX12	AC090617.4	0.51523331	7.03E-38	postive
ACSBG2	AC090617.4	0.57062112	6.94E-48	postive
ELOVL2	AC022613.2	0.69130355	8.18E-78	postive
CYP4B1	AC022613.2	0.72438565	8.58E-89	postive
FASN	AC022613.2	0.63105206	3.26E-61	postive
CPT1B	AC108134.3	0.51474977	8.44E-38	postive
D2HGDH	AC108134.3	0.55207474	2.52E-44	postive
ALOX12	AC108134.3	0.51955864	1.35E-38	postive
HSD17B3	AC025265.1	0.57184251	3.97E-48	postive
ALOX12	AC079584.2	0.55935134	1.08E-45	postive
PRKAA2	AC073073.2	0.51165533	2.70E-37	postive

PCBD1	LINC01443	0.65533235	1.91E-67	postive
ALOX12	AC016026.1	0.63402971	6.01E-62	postive
ACOT6	AC006042.3	0.88184523	1.86E-177	postive
BMPR1B	AC006042.3	0.69206818	4.74E-78	postive
ALOX12	LINC00173	0.59148722	3.60E-52	postive
ACSBG2	AC016027.3	0.55585678	4.94E-45	postive
ACOT6	AC103739.2	0.50762863	1.21E-36	postive
HSD17B3	AP000553.2	0.61042212	2.44E-56	postive
ALOX12	AC087289.1	0.67215326	4.13E-72	postive
HSD17B3	MAP3K2-DT	0.50630286	1.97E-36	postive
ACOT6	AC000061.1	0.91307254	2.42E-211	postive
BMPR1B	AC000061.1	0.78317481	6.81E-113	postive
HSD17B3	Z84485.1	0.54192674	1.81E-42	postive
ALOX12	Z84485.1	0.54313143	1.10E-42	postive
ACSBG2	Z84485.1	0.54623518	3.00E-43	postive
ALOX12	AC053527.2	0.57917457	1.33E-49	postive
CPT1B	SNHG10	0.54848201	1.16E-43	postive
ELOVL2	AC022146.2	0.67412493	1.12E-72	postive
ACSBG2	AC022146.2	0.62624877	4.80E-60	postive
CYP4B1	AC022146.2	0.73501082	1.15E-92	postive

ACOT6	AC022146.2	0.57817649	2.12E-49	postive
FASN	AC022146.2	0.65053277	3.62E-66	postive
HSD17B3	AL354760.1	0.70918376	1.48E-83	postive
CPT1B	AL354760.1	0.51933024	1.47E-38	postive
ACOT6	AC010148.1	0.51800151	2.45E-38	postive
ALOX12	AC008635.1	0.53940892	5.10E-42	postive
CPT1B	AL583810.1	0.50962037	5.77E-37	postive
D2HGDH	AL583810.1	0.52266493	4.07E-39	postive
ELOVL2	AL512306.2	0.71376131	4.26E-85	postive
ACSBG2	AL512306.2	0.69730518	1.08E-79	postive
CYP4B1	AL512306.2	0.74454071	2.64E-96	postive
FASN	AL512306.2	0.68735991	1.33E-76	postive
CPT1B	AC004466.1	0.50440784	3.94E-36	postive
D2HGDH	AC004466.1	0.50962556	5.76E-37	postive
ELOVL2	AL023803.2	0.8257052	1.25E-135	postive
ACSBG2	AL023803.2	0.64068075	1.29E-63	postive
CYP4B1	AL023803.2	0.8666631	2.69E-164	postive
FASN	AL023803.2	0.82700719	2.03E-136	postive
ACOT6	ZDHHC20-IT1	0.63641374	1.53E-62	postive
CPT1B	AL121832.3	0.59154316	3.50E-52	postive
ALOX12	AL121832.3	0.51519221	7.14E-38	postive

ACSBG1	AC124852.1	0.55700985	2.99E-45	postive
ENO3	AC124852.1	0.55932365	1.09E-45	postive
PCBD1	AC124852.1	0.5717329	4.18E-48	postive
HACL1	AC020978.4	0.51850462	2.02E-38	postive
BMPR1B	AC020978.4	0.57042568	7.59E-48	postive
RDH11	AC020978.4	0.72022013	2.53E-87	postive
ACACB	AC020978.4	0.56621988	5.09E-47	postive
ALOX12	AL031320.2	0.68052292	1.50E-74	postive
ALOX12	AL355574.1	0.56922808	1.31E-47	postive
CPT1B	MIR4453HG	0.56957789	1.12E-47	postive
SDHD	AC007342.4	0.5382349	8.25E-42	postive
HACD3	AC007342.4	0.56022501	7.33E-46	postive
AUH	AC007342.4	0.5617136	3.80E-46	postive
ACOT6	AC007342.4	0.5356137	2.40E-41	postive
BMPR1B	AC007342.4	0.81413597	7.20E-129	postive
ELOVL4	AC007342.4	0.57515853	8.63E-49	postive
RDH11	AC007342.4	0.67436856	9.50E-73	postive
GABARAPL1	AC007342.4	0.61786387	4.69E-58	postive
ACACB	AC007342.4	0.55687941	3.17E-45	postive
ALOX12	DLGAP4-AS1	0.58148001	4.47E-50	postive
ACSBG2	DLGAP4-AS1	0.55535416	6.14E-45	postive
ACOT6	AC104779.1	0.78824273	2.53E-115	postive

BMPR1B	AC104779.1	0.58168835	4.05E-50	postive
ELOVL2	AC027228.1	0.51274472	1.80E-37	postive
ACSBG2	AC027228.1	0.58706637	3.09E-51	postive
CYP4B1	AC027228.1	0.56910534	1.38E-47	postive
ACOT6	AC027228.1	0.50347671	5.53E-36	postive
ELOVL2	AC025576.2	0.72612831	2.04E-89	postive
ACSBG2	AC025576.2	0.7004613	1.06E-80	postive
CYP4B1	AC025576.2	0.7799215	2.29E-111	postive
FASN	AC025576.2	0.7182907	1.19E-86	postive
ACACB	AC024075.1	0.57979567	9.89E-50	postive
ACOT6	AC022272.1	0.52843749	4.24E-40	postive
ALOX12	AC079807.1	0.59539499	5.23E-53	postive
ALOX12	AC026771.1	0.59549643	4.97E-53	postive
EHHADH	LINC02747	0.54958949	7.28E-44	postive
SLC27A2	LINC02747	0.51350825	1.35E-37	postive
CPT1B	AC010201.2	0.52251464	4.32E-39	postive
D2HGDH	AC010201.2	0.50910698	6.98E-37	postive
ALOX12	AC010201.2	0.71273969	9.47E-85	postive
D2HGDH	AC021016.2	0.58750891	2.50E-51	postive
ALOX12	AC021016.2	0.53182776	1.10E-40	postive
CPT1B	AC093726.2	0.52186733	5.55E-39	postive
D2HGDH	AC093726.2	0.53023951	2.07E-40	postive

ELOVL2	AL450384.2	0.68012861	1.97E-74	postive
HSD17B3	AL450384.2	0.53486109	3.25E-41	postive
ALOX12	AL450384.2	0.53316176	6.45E-41	postive
ACSBG2	AL450384.2	0.68514213	6.25E-76	postive
CYP4B1	AL450384.2	0.70894812	1.77E-83	postive
FASN	AL450384.2	0.64416922	1.65E-64	postive
ACOT6	Z83843.1	0.60215724	1.74E-54	postive
HSD17B3	AC232271.1	0.50485944	3.34E-36	postive
CPT1B	AC232271.1	0.69745087	9.70E-80	postive
D2HGDH	AC232271.1	0.62682766	3.48E-60	postive
ALOX12	AC232271.1	0.68887761	4.57E-77	postive
ACSBG2	AC232271.1	0.51788652	2.56E-38	postive
CPT1B	AC084125.2	0.53646935	1.69E-41	postive
D2HGDH	AC084125.2	0.55659805	3.58E-45	postive
ELOVL2	FAM66C	0.67156486	6.08E-72	postive
ACSBG2	FAM66C	0.62356857	2.11E-59	postive
CYP4B1	FAM66C	0.70293221	1.69E-81	postive
FASN	FAM66C	0.68056566	1.46E-74	postive
ELOVL2	AC079414.3	0.67198806	4.60E-72	postive
ACSBG2	AC079414.3	0.65825863	3.10E-68	postive

CYP4B1	AC079414.3	0.73047159	5.46E-91	postive
FASN	AC079414.3	0.65645338	9.56E-68	postive
SERINC1	AC005332.6	0.50362253	5.24E-36	postive
ELOVL2	LINC01786	0.53493238	3.16E-41	postive
CPT1B	LINC01786	0.50181003	1.01E-35	postive
D2HGDH	LINC01786	0.58193067	3.61E-50	postive
ALOX12	LINC01786	0.6343308	5.06E-62	postive
ACSBG2	LINC01786	0.64914442	8.40E-66	postive
CYP4B1	LINC01786	0.57807013	2.22E-49	postive
FASN	LINC01786	0.53145914	1.28E-40	postive
ALOX12	GABPB1-AS1	0.65813996	3.34E-68	postive
HSD17B3	AC091057.3	0.51010928	4.81E-37	postive
KMT5A	SNHG3	0.55496678	7.26E-45	postive
HSD17B3	AC026412.3	0.50147222	1.14E-35	postive
SERINC1	SNHG9	-0.5065514	1.79E-36	negative
HSD17B3	RAD51-AS1	0.50220843	8.77E-36	postive
CPT1B	RAD51-AS1	0.68265917	3.48E-75	postive
D2HGDH	RAD51-AS1	0.63649791	1.46E-62	postive
ALOX12	RAD51-AS1	0.6958656	3.08E-79	postive
ALOX12	AL050343.2	0.58241671	2.87E-50	postive
ACOT6	AC007637.1	0.51087962	3.61E-37	postive
BMPR1B	AC007637.1	0.6215897	6.23E-59	postive

CBR4	AC007637.1	0.53676808	1.50E-41	postive
GABARAPL1	AC007637.1	0.51577438	5.73E-38	postive
ACACB	AC007637.1	0.73668047	2.71E-93	postive
ELOVL2	LINC02569	0.56906119	1.41E-47	postive
ACSBG2	LINC02569	0.59630633	3.32E-53	postive
CYP4B1	LINC02569	0.64769682	2.01E-65	postive
FASN	LINC02569	0.55921053	1.14E-45	postive
D2HGDH	AC136475.2	0.52949579	2.79E-40	postive
ALOX12	AC136475.2	0.50507704	3.08E-36	postive
HSD17B3	PTOV1-AS1	0.58701591	3.17E-51	postive
CPT1B	PTOV1-AS1	0.52030897	1.01E-38	postive
ALOX12	PTOV1-AS1	0.50774894	1.15E-36	postive
ALOX12	AL121987.2	0.57257557	2.84E-48	postive
CPT1B	AC107375.1	0.56875672	1.62E-47	postive
D2HGDH	AC107375.1	0.63303636	1.06E-61	postive
ALOX12	AC107375.1	0.66959335	2.22E-71	postive
ACSBG2	AC107375.1	0.51514318	7.27E-38	postive
ALOX12	AC009120.3	0.67451565	8.62E-73	postive
ACOT6	AL136115.1	0.54818918	1.32E-43	postive
TBXAS1	LINC02528	0.51949346	1.38E-38	postive

ELOVL2	AC092112.1	0.64794213	1.73E-65	postive
ACSBG2	AC092112.1	0.58910472	1.15E-51	postive
CYP4B1	AC092112.1	0.6947053	7.13E-79	postive
FASN	AC092112.1	0.64858853	1.17E-65	postive
ELOVL2	LINC02544	0.66182143	3.29E-69	postive
ACSBG2	LINC02544	0.51248874	1.98E-37	postive
CYP4B1	LINC02544	0.6595489	1.38E-68	postive
FASN	LINC02544	0.66116195	5.00E-69	postive
CPT1B	AC132872.1	0.50108252	1.32E-35	postive
D2HGDH	AC132872.1	0.5621353	3.15E-46	postive
ELOVL2	CENPN-AS1	0.65547668	1.75E-67	postive
ACSBG2	CENPN-AS1	0.56591276	5.85E-47	postive
CYP4B1	CENPN-AS1	0.67833946	6.62E-74	postive
FASN	CENPN-AS1	0.62953563	7.66E-61	postive
ELOVL2	AC100861.1	0.52699775	7.49E-40	postive
FASN	AC100861.1	0.61497411	2.20E-57	postive
ELOVL2	AC007292.3	0.82215502	1.68E-133	postive
ACSBG2	AC007292.3	0.67604407	3.10E-73	postive
CYP4B1	AC007292.3	0.88747346	8.40E-183	postive
FASN	AC007292.3	0.81680574	2.19E-130	postive
ACOT6	AC083805.2	0.70491433	3.81E-82	postive
ELOVL2	AC114980.1	0.61519923	1.95E-57	postive

ACSBG2	AC114980.1	0.66812242	5.78E-71	postive
CYP4B1	AC114980.1	0.67432986	9.75E-73	postive
FASN	AC114980.1	0.57213232	3.48E-48	postive
ALOX12	AC004908.2	0.54049759	3.26E-42	postive
ACOT6	AC004908.2	0.62192156	5.19E-59	postive
SDHD	LINC01802	0.5319283	1.06E-40	postive
PHYH	LINC01802	0.55231959	2.27E-44	postive
HACD3	LINC01802	0.56915029	1.36E-47	postive
AUH	LINC01802	0.61118967	1.63E-56	postive
HACL1	LINC01802	0.55255161	2.06E-44	postive
BMPR1B	LINC01802	0.64999421	5.02E-66	postive
ELOVL4	LINC01802	0.6350705	3.31E-62	postive
RDH11	LINC01802	0.74655111	4.30E-97	postive
GABARAPL1	LINC01802	0.63263428	1.33E-61	postive
ACACB	LINC01802	0.58466887	9.79E-51	postive
ACOT6	AC036108.1	0.51496322	7.78E-38	postive
ACOT6	AC007342.5	0.71142958	2.62E-84	postive
BMPR1B	AC007342.5	0.78791997	3.63E-115	postive
ELOVL4	AC007342.5	0.50770388	1.17E-36	postive
RDH11	AC007342.5	0.58638369	4.30E-51	postive
GABARAPL1	AC007342.5	0.54143117	2.22E-42	postive

ACACB	AC007342.5	0.51284384	1.73E-37	postive
D2HGDH	AC010976.2	0.56050016	6.49E-46	postive
ALOX12	AC027801.1	0.52647003	9.21E-40	postive
ACACB	AC010333.1	0.51004921	4.92E-37	postive
ELOVL2	AC119396.1	0.83507158	1.78E-141	postive
ACSBG2	AC119396.1	0.66847967	4.58E-71	postive
CYP4B1	AC119396.1	0.8979489	1.48E-193	postive
FASN	AC119396.1	0.82606657	7.58E-136	postive
ACSBG1	LINC02012	0.66509016	4.09E-70	postive
ENO3	LINC02012	0.71549515	1.09E-85	postive
PCBD1	LINC02012	0.56011584	7.69E-46	postive
ALOX12	AC104564.1	0.53979575	4.35E-42	postive
ACSBG2	AC104564.1	0.56637469	4.75E-47	postive
ELOVL2	AC006441.4	0.71832983	1.15E-86	postive
ACSBG2	AC006441.4	0.66593837	2.37E-70	postive
CYP4B1	AC006441.4	0.77170977	1.25E-107	postive
FASN	AC006441.4	0.68672866	2.07E-76	postive
DPEP2	AL590764.1	0.54087369	2.79E-42	postive
TBXAS1	AL590764.1	0.63397942	6.19E-62	postive
PRKAG2	SLC25A5-AS1	0.59760772	1.73E-53	postive
SDHD	SLC25A5-AS1	0.55326949	1.51E-44	postive

PHYH	SLC25A5-AS1	0.51178817	2.57E-37	postive
PDHB	SLC25A5-AS1	0.59188129	2.97E-52	postive
HACD3	SLC25A5-AS1	0.61056736	2.26E-56	postive
AUH	SLC25A5-AS1	0.6668956	1.28E-70	postive
HACL1	SLC25A5-AS1	0.58012337	8.48E-50	postive
BMPR1B	SLC25A5-AS1	0.58841628	1.61E-51	postive
ELOVL4	SLC25A5-AS1	0.57045338	7.50E-48	postive
RDH11	SLC25A5-AS1	0.67808244	7.88E-74	postive
GABARAPL1	SLC25A5-AS1	0.67622546	2.75E-73	postive
ACACB	SLC25A5-AS1	0.62429865	1.41E-59	postive
DLST	SLC25A5-AS1	0.51229654	2.13E-37	postive
TBXAS1	LINC01150	0.55978559	8.89E-46	postive
ACOT6	SPATA13-AS1	0.74753965	1.75E-97	postive
BMPR1B	SPATA13-AS1	0.54367681	8.74E-43	postive
ALOX12	AC007991.4	0.51610771	5.05E-38	postive
CPT1B	AC087500.1	0.57506989	8.99E-49	postive
D2HGDH	AC087500.1	0.62909161	9.82E-61	postive
ALOX12	AC087500.1	0.79675504	1.47E-119	postive
ACOT6	AC007849.1	0.90152464	1.69E-197	postive
BMPR1B	AC007849.1	0.70179633	3.94E-81	postive
ELOVL2	AL109936.2	0.61065996	2.16E-56	postive

ACSBG2	AL109936.2	0.63995768	1.96E-63	postive
CYP4B1	AL109936.2	0.69956906	2.05E-80	postive
FASN	AL109936.2	0.5769559	3.74E-49	postive
HSD17B3	AC007038.1	0.50531249	2.83E-36	postive
ALOX12	AC007038.1	0.63518142	3.11E-62	postive
ACSBG2	AC007038.1	0.55728581	2.65E-45	postive
ACOT6	GRK5-IT1	0.58256647	2.67E-50	postive
PCBD1	AC010307.4	0.50235367	8.32E-36	postive
ALOX12	AC100778.2	0.66389902	8.78E-70	postive
ACSBG2	AC100778.2	0.66022863	9.01E-69	postive
CYP4B1	AC100778.2	0.56022362	7.33E-46	postive
DPEP2	AC004921.1	0.61664068	9.04E-58	postive
ACSBG2	AP003471.1	0.53390073	4.79E-41	postive
ACOT6	AC083843.2	0.88341754	6.38E-179	postive
BMPR1B	AC083843.2	0.68324436	2.32E-75	postive
HSD17B3	AC104794.3	0.56402937	1.36E-46	postive
ALOX12	AC093797.1	0.60131711	2.67E-54	postive
BMPR1B	AC090515.2	0.57841326	1.89E-49	postive
RDH11	AC090515.2	0.65665794	8.42E-68	postive
GABARAPL1	AC090515.2	0.53999686	4.01E-42	postive
ACOT6	MYCBP2-AS2	0.7487319	5.89E-98	postive

PDHB	AC005281.1	0.51862624	1.93E-38	postive
HACL1	AC005281.1	0.57917304	1.33E-49	postive
BMPR1B	AC005281.1	0.56401539	1.37E-46	postive
RDH11	AC005281.1	0.53197834	1.04E-40	postive
GABARAPL1	AC005281.1	0.61166496	1.27E-56	postive
PRKAG2	HOXB-AS3	0.57365992	1.72E-48	postive
ACOT8	HOXB-AS3	0.57569987	6.71E-49	postive
SDHD	HOXB-AS3	0.51882655	1.79E-38	postive
PDHB	HOXB-AS3	0.51263711	1.87E-37	postive
HACD3	HOXB-AS3	0.59735331	1.97E-53	postive
AUH	HOXB-AS3	0.53967845	4.57E-42	postive
HACL1	HOXB-AS3	0.61725217	6.51E-58	postive
ELOVL4	HOXB-AS3	0.64526584	8.59E-65	postive
RDH11	HOXB-AS3	0.60798606	8.70E-56	postive
GABARAPL1	HOXB-AS3	0.52532888	1.44E-39	postive
ALOX12	SSBP3-AS1	0.59674471	2.67E-53	postive
ELOVL2	AP001972.4	0.7476598	1.57E-97	postive
ACSBG2	AP001972.4	0.64390896	1.92E-64	postive
CYP4B1	AP001972.4	0.79064544	1.69E-116	postive
FASN	AP001972.4	0.75426111	3.47E-100	postive
ELOVL2	AC007823.1	0.50071737	1.50E-35	postive

ACSBG2	AC007823.1	0.50059326	1.57E-35	postive
CYP4B1	AC007823.1	0.51190588	2.46E-37	postive
ACOT6	AC007823.1	0.57288983	2.46E-48	postive
HSD17B3	AC139530.1	0.52236806	4.57E-39	postive
D2HGDH	AC139530.1	0.59322116	1.53E-52	postive
ACADVL	AC139530.1	0.50721813	1.40E-36	postive
FAAH	AC139530.1	0.50110782	1.31E-35	postive
ALOX12	AC020911.1	0.68383072	1.55E-75	postive
ACSBG2	AC020911.1	0.54984871	6.52E-44	postive
ACOT6	AL138689.1	0.67240566	3.49E-72	postive
ELOVL2	AL163051.1	0.74264525	1.44E-95	postive
ACSBG2	AL163051.1	0.59418999	9.51E-53	postive
CYP4B1	AL163051.1	0.78101709	7.05E-112	postive
FASN	AL163051.1	0.75929695	2.86E-102	postive
ALOX12	ZNF30-AS1	0.56871451	1.65E-47	postive
ELOVL2	AC123768.1	0.73180736	1.77E-91	postive
ACSBG2	AC123768.1	0.67772523	1.00E-73	postive
CYP4B1	AC123768.1	0.7997277	4.38E-121	postive
FASN	AC123768.1	0.73071556	4.44E-91	postive
ACOT6	AL589935.1	0.56818332	2.10E-47	postive
HSD17B3	AC004253.1	0.55637293	3.95E-45	postive

CPT1B	AC004253.1	0.58212309	3.29E-50	postive
D2HGDH	AC004253.1	0.58331751	1.87E-50	postive
ALOX12	AC004253.1	0.67816225	7.46E-74	postive
ACSBG2	CARNMT1-AS1	0.53374332	5.10E-41	postive
ACOT6	CARNMT1-AS1	0.60714692	1.35E-55	postive
ELOVL2	AP000692.2	0.51496775	7.77E-38	postive
CYP4B1	AP000692.2	0.51113806	3.28E-37	postive
ACOT6	ITFG1-AS1	0.71624609	6.03E-86	postive
BMPR1B	ITFG1-AS1	0.71175618	2.03E-84	postive
PRKAG2	BAALC-AS2	0.52547564	1.36E-39	postive
ACOT8	BAALC-AS2	0.61855938	3.22E-58	postive
SDHD	BAALC-AS2	0.69865327	4.02E-80	postive
PHYH	BAALC-AS2	0.62795182	1.86E-60	postive
NDUFAB1	BAALC-AS2	0.55967763	9.32E-46	postive
HACD3	BAALC-AS2	0.72734446	7.46E-90	postive
AUH	BAALC-AS2	0.61849764	3.33E-58	postive
ELOVL4	BAALC-AS2	0.76368139	3.99E-104	postive
GABARAPL1	BAALC-AS2	0.5302783	2.04E-40	postive
ALOX12	AC080162.1	0.57571084	6.68E-49	postive
ELOVL2	AC093001.1	0.90614023	8.16E-203	postive

ACSBG2	AC093001.1	0.61181958	1.17E-56	postive
CYP4B1	AC093001.1	0.92229425	7.31E-224	postive
FASN	AC093001.1	0.87445523	7.75E-171	postive
ACOT6	AC091167.5	0.60970581	3.55E-56	postive
ELOVL2	AC009509.4	0.71487055	1.79E-85	postive
ACSBG2	AC009509.4	0.60573435	2.79E-55	postive
CYP4B1	AC009509.4	0.80003245	3.05E-121	postive
FASN	AC009509.4	0.67615809	2.88E-73	postive
ELOVL2	AL356299.2	0.61591966	1.33E-57	postive
ALOX12	AL356299.2	0.54028121	3.56E-42	postive
ACSBG2	AL356299.2	0.67647905	2.32E-73	postive
CYP4B1	AL356299.2	0.65687368	7.36E-68	postive
FASN	AL356299.2	0.63155158	2.46E-61	postive
ALOX12	ATP1A1-AS1	0.51593889	5.38E-38	postive
ALOX12	AP003498.1	0.58269364	2.51E-50	postive
ACSBG2	AP003498.1	0.64006888	1.84E-63	postive
CYP4B1	AP003498.1	0.5016825	1.06E-35	postive
ALOX12	NRIR	0.59373976	1.19E-52	postive
ACOT6	AC087683.2	0.73963778	2.06E-94	postive
BMPR1B	AC087683.2	0.50431416	4.07E-36	postive
ALOX12	ZNF346-IT1	0.52997158	2.31E-40	postive

ACSBG2	ZNF346-IT1	0.58153115	4.36E-50	postive
ACOT6	ZNF346-IT1	0.60382701	7.44E-55	postive
ALOX12	AC007216.4	0.54548142	4.11E-43	postive
ACOT6	AC007216.4	0.60841879	6.95E-56	postive
CYP4B1	AC104667.2	0.5336054	5.39E-41	postive
ELOVL2	AC137932.3	0.61015639	2.81E-56	postive
ALOX12	AC137932.3	0.59737281	1.95E-53	postive
ACSBG2	AC137932.3	0.66796545	6.40E-71	postive
CYP4B1	AC137932.3	0.65668536	8.27E-68	postive
FASN	AC137932.3	0.61391761	3.86E-57	postive
FAAH	AC010503.4	0.55541948	5.97E-45	postive
ACAA2	LHFPL3-AS2	0.54954586	7.41E-44	postive
ALDH3A2	LHFPL3-AS2	0.50732928	1.35E-36	postive
SLC27A2	LHFPL3-AS2	0.59977579	5.83E-54	postive
GGT1	LHFPL3-AS2	0.5139391	1.15E-37	postive
ALOX12	AC005332.2	0.60355423	8.55E-55	postive
ALOX12	LINC02532	0.50388206	4.77E-36	postive
CPT1B	AC005104.1	0.58356287	1.66E-50	postive
D2HGDH	AC005104.1	0.54792625	1.47E-43	postive
ALOX12	AC005104.1	0.67476295	7.31E-73	postive
BMPR1B	LINC02041	0.6094488	4.06E-56	postive
CPT1B	AL117209.1	0.63894253	3.55E-63	postive

D2HGDH	AL117209.1	0.55340375	1.43E-44	postive
ALOX12	AL117209.1	0.55075617	4.43E-44	postive
ELOVL2	MIR3659HG	0.68990287	2.21E-77	postive
CYP4B1	MIR3659HG	0.57432066	1.27E-48	postive
FASN	MIR3659HG	0.54177062	1.93E-42	postive
ELOVL2	AL031733.2	0.70869433	2.16E-83	postive
ACSBG2	AL031733.2	0.64747957	2.29E-65	postive
CYP4B1	AL031733.2	0.76085371	6.35E-103	postive
FASN	AL031733.2	0.68766781	1.07E-76	postive
ACOT6	AC023051.1	0.88942918	9.99E-185	postive
BMPR1B	AC023051.1	0.68322277	2.36E-75	postive
ACOT6	AC007285.1	0.54020044	3.68E-42	postive
HACL1	AL035530.1	0.51480807	8.25E-38	postive
BMPR1B	AL035530.1	0.66894675	3.38E-71	postive
ELOVL4	AL035530.1	0.66854036	4.40E-71	postive
RDH11	AL035530.1	0.68764643	1.09E-76	postive
GABARAPL1	AL035530.1	0.52354317	2.89E-39	postive
ACSBG2	CRTC3-AS1	0.52523712	1.49E-39	postive
ALOX12	AL132989.1	0.70827452	2.97E-83	postive
ELOVL2	AP001269.2	0.65544082	1.79E-67	postive

ACSBG2	AP001269.2	0.55487923	7.54E-45	postive
CYP4B1	AP001269.2	0.72145829	9.31E-88	postive
FASN	AP001269.2	0.6338378	6.71E-62	postive
ELOVL2	AC026741.1	0.61677561	8.41E-58	postive
ACSBG2	AC026741.1	0.64210618	5.57E-64	postive
CYP4B1	AC026741.1	0.64499946	1.01E-64	postive
FASN	AC026741.1	0.5583298	1.68E-45	postive
ELOVL2	AC130469.1	0.82697319	2.13E-136	postive
ACSBG2	AC130469.1	0.68152894	7.56E-75	postive
CYP4B1	AC130469.1	0.87608088	2.95E-172	postive
FASN	AC130469.1	0.81214732	9.37E-128	postive
ALOX12	AP001619.1	0.55353186	1.35E-44	postive
ACSBG2	AP001619.1	0.51573693	5.81E-38	postive
ACOT6	LINC01266	0.63216048	1.74E-61	postive
BMPR1B	LINC01266	0.55345137	1.40E-44	postive
ALOX12	AC005899.6	0.65247473	1.11E-66	postive
ACSBG2	AC005899.6	0.5415604	2.10E-42	postive
HACL1	SUCLG2-AS1	0.54675148	2.41E-43	postive
ACOT6	AC011379.1	0.6288716	1.11E-60	postive
ACOT6	AC018761.3	0.67766573	1.04E-73	postive
HSD17B3	AC016957.2	0.53414246	4.35E-41	postive
PRKAG2	AC148477.4	0.52241099	4.49E-39	postive

SDHD	AC148477.4	0.50486385	3.33E-36	postive
PDHB	AC148477.4	0.54655208	2.63E-43	postive
HACD3	AC148477.4	0.57375547	1.65E-48	postive
AUH	AC148477.4	0.62191797	5.20E-59	postive
BMPR1B	AC148477.4	0.53609918	1.97E-41	postive
ELOVL4	AC148477.4	0.59911112	8.15E-54	postive
RDH11	AC148477.4	0.6808986	1.16E-74	postive
GABARAPL1	AC148477.4	0.74076823	7.60E-95	postive
ACACB	AC148477.4	0.61516168	1.99E-57	postive
ELOVL2	AL138999.1	0.53992443	4.13E-42	postive
ACSBG2	AL138999.1	0.59623415	3.44E-53	postive
CYP4B1	AL138999.1	0.58768217	2.30E-51	postive
FASN	AL138999.1	0.51220807	2.20E-37	postive
ACOT6	AC093535.1	0.65049862	3.70E-66	postive
ALOX12	Z98884.2	0.67200656	4.55E-72	postive
CPT1B	PRKCZ-AS1	0.52137966	6.70E-39	postive
D2HGDH	PRKCZ-AS1	0.61418383	3.35E-57	postive
ALOX12	PRKCZ-AS1	0.5376818	1.03E-41	postive
CBR4	AP003486.1	0.55821958	1.77E-45	postive
CPT1B	AC008760.1	0.57529399	8.10E-49	postive
D2HGDH	AC008760.1	0.60348347	8.86E-55	postive

ACADVL	AC008760.1	0.53728908	1.21E-41	postive
ALOX12	AC008760.1	0.59888632	9.13E-54	postive
ACOT6	AC005753.1	0.75735472	1.85E-101	postive
BMPR1B	AC005753.1	0.58577253	5.77E-51	postive
ALOX12	AC004771.1	0.65324894	6.91E-67	postive
HSD17B3	PLA2G4C-AS1	0.53101065	1.53E-40	postive
CPT1B	PLA2G4C-AS1	0.52433916	2.12E-39	postive
D2HGDH	PLA2G4C-AS1	0.57847126	1.84E-49	postive
HSD17B3	AC009118.3	0.54865101	1.08E-43	postive
CPT1B	AC009118.3	0.60053859	3.97E-54	postive
D2HGDH	AC009118.3	0.53631019	1.81E-41	postive
ALOX12	AC009118.3	0.56332613	1.86E-46	postive
ELOVL2	AC022400.1	0.65647838	9.41E-68	postive
ALOX12	AC022400.1	0.50808749	1.02E-36	postive
ACSBG2	AC022400.1	0.63645843	1.49E-62	postive
CYP4B1	AC022400.1	0.69780584	7.48E-80	postive
FASN	AC022400.1	0.65533273	1.91E-67	postive
ACOT6	LNX1-AS2	0.51412416	1.07E-37	postive
SDHD	B4GALT1-AS1	0.55439001	9.32E-45	postive
PHYH	B4GALT1-AS1	0.54284706	1.23E-42	postive

NDUFAB1	B4GALT1-AS1	0.55955209	9.85E-46	postive
HACD3	B4GALT1-AS1	0.50816082	9.91E-37	postive
ELOVL4	B4GALT1-AS1	0.56301583	2.13E-46	postive
RDH11	B4GALT1-AS1	0.56189478	3.50E-46	postive
ACSBG2	DNAJB5-DT	0.51211946	2.27E-37	postive
ACOT6	AC025271.2	0.8682473	1.36E-165	postive
BMPR1B	AC025271.2	0.7434173	7.22E-96	postive
ALOX12	AC010997.2	0.5654303	7.26E-47	postive
ACOT6	AP001021.2	0.65610579	1.19E-67	postive
BMPR1B	AP001021.2	0.53777195	9.97E-42	postive
TBXAS1	AC090559.1	0.61957173	1.86E-58	postive
ELOVL2	AL354872.1	0.6677797	7.22E-71	postive
ACSBG2	AL354872.1	0.57759313	2.78E-49	postive
CYP4B1	AL354872.1	0.71133248	2.83E-84	postive
FASN	AL354872.1	0.69244258	3.63E-78	postive
ELOVL2	AL157871.5	0.53245892	8.55E-41	postive
ALOX12	AL157871.5	0.58916265	1.12E-51	postive
ACSBG2	AL157871.5	0.67468041	7.72E-73	postive
CYP4B1	AL157871.5	0.58505462	8.14E-51	postive
FASN	AL157871.5	0.51055357	4.08E-37	postive
PRKAG2	AL031123.1	0.57602332	5.78E-49	postive
ELOVL2	AC083841.1	0.82570978	1.25E-135	postive

ACSBG2	AC083841.1	0.63330723	9.08E-62	postive
CYP4B1	AC083841.1	0.88472165	3.74E-180	postive
FASN	AC083841.1	0.84790707	4.05E-150	postive
HSD17B3	ARHGAP27P1-BPTFP1-KPNA2P3	0.53997156	4.05E-42	postive
CPT1B	ARHGAP27P1-BPTFP1-KPNA2P3	0.62725075	2.75E-60	postive
D2HGDH	ARHGAP27P1-BPTFP1-KPNA2P3	0.633872	6.58E-62	postive
ALOX12	ARHGAP27P1-BPTFP1-KPNA2P3	0.65935924	1.56E-68	postive
D2HGDH	AL360181.1	0.52659012	8.79E-40	postive
ALOX12	AL360181.1	0.57057868	7.08E-48	postive
ALOX12	AC244093.5	0.51896829	1.69E-38	postive
ELOVL2	AC026495.1	0.63173047	2.22E-61	postive
ACSBG2	AC026495.1	0.59947316	6.79E-54	postive
CYP4B1	AC026495.1	0.68975059	2.47E-77	postive
FASN	AC026495.1	0.5935205	1.32E-52	postive
ACOT6	LINC01535	0.55783561	2.09E-45	postive
BMPR1B	LINC01535	0.52453897	1.96E-39	postive
ALOX12	WASHC5-AS1	0.52228528	4.72E-39	postive
ACOT6	WASHC5-AS1	0.50006979	1.90E-35	postive
GABARAPL1	MID1IP1-AS1	0.52462442	1.90E-39	postive
ELOVL2	LIF-AS1	0.81526592	1.65E-129	postive
ACSBG2	LIF-AS1	0.63224683	1.66E-61	postive

CYP4B1	LIF-AS1	0.8541836	1.22E-154	postive
FASN	LIF-AS1	0.81749441	8.81E-131	postive
AUH	COLCA1	0.53328613	6.13E-41	postive
ACOT6	COLCA1	0.70610929	1.54E-82	postive
BMPR1B	COLCA1	0.76562936	5.80E-105	postive
RDH11	COLCA1	0.63530756	2.89E-62	postive
GABARAPL1	COLCA1	0.54729424	1.92E-43	postive
ACACB	COLCA1	0.5935586	1.30E-52	postive
HACL1	LINC01214	0.52228276	4.72E-39	postive
ELOVL4	LINC01214	0.57237446	3.11E-48	postive
RDH11	LINC01214	0.62942802	8.13E-61	postive
CPT1B	AL662884.1	0.58780417	2.16E-51	postive
D2HGDH	AL662884.1	0.62294605	2.96E-59	postive
ALOX12	AL662884.1	0.53903724	5.94E-42	postive
ELOVL2	AC018766.1	0.68411317	1.28E-75	postive
ACSBG2	AC018766.1	0.7121803	1.46E-84	postive
CYP4B1	AC018766.1	0.74654747	4.31E-97	postive
FASN	AC018766.1	0.66433417	6.65E-70	postive
ACOT6	AC069079.1	0.74175487	3.17E-95	postive
HSD17B3	AC010973.2	0.52035087	9.96E-39	postive
CPT1B	AC010973.2	0.68178971	6.32E-75	postive
D2HGDH	AC010973.2	0.54003416	3.95E-42	postive

ELOVL2	AP002812.3	0.50694956	1.55E-36	postive
ALOX12	AP002812.3	0.52140239	6.64E-39	postive
ACSBG2	AP002812.3	0.65660143	8.72E-68	postive
CYP4B1	AP002812.3	0.54741771	1.82E-43	postive
ELOVL2	AL365295.1	0.62282705	3.16E-59	postive
ACSBG2	AL365295.1	0.55779203	2.13E-45	postive
CYP4B1	AL365295.1	0.63006543	5.69E-61	postive
FASN	AL365295.1	0.56845178	1.86E-47	postive
ELOVL2	AL357874.1	0.66272717	1.85E-69	postive
ACSBG2	AL357874.1	0.65574246	1.49E-67	postive
CYP4B1	AL357874.1	0.73988945	1.65E-94	postive
FASN	AL357874.1	0.63006381	5.69E-61	postive
ALOX12	AL354893.2	0.52570546	1.24E-39	postive
ACOT6	AL354893.2	0.56390468	1.44E-46	postive
ALOX12	AC020915.1	0.55126118	3.57E-44	postive
ACSBG2	AC020915.1	0.60373661	7.79E-55	postive
PRKAG2	AL356740.1	0.52460221	1.92E-39	postive
BMPR1B	AL356740.1	0.58601021	5.14E-51	postive
ELOVL4	AL356740.1	0.52712515	7.12E-40	postive
RDH11	AL356740.1	0.53822025	8.30E-42	postive

GABARAPL1	AL356740.1	0.63185581	2.07E-61	postive
ACACB	AL356740.1	0.5937517	1.18E-52	postive
ELOVL2	AC012363.2	0.83276455	5.29E-140	postive
ACSBG2	AC012363.2	0.6418823	6.36E-64	postive
CYP4B1	AC012363.2	0.89569964	3.76E-191	postive
FASN	AC012363.2	0.83055067	1.31E-138	postive
ACOT6	AC008147.1	0.888777	4.42E-184	postive
BMPR1B	AC008147.1	0.81912172	1.01E-131	postive
ACOT6	Z82243.1	0.80832156	1.20E-125	postive
BMPR1B	Z82243.1	0.57534368	7.92E-49	postive
ACOT6	AC253536.3	0.54486441	5.33E-43	postive
MMAA	AC107027.3	0.51295916	1.66E-37	postive
CPT1B	AC104964.3	0.51213705	2.26E-37	postive
D2HGDH	AC104964.3	0.63166291	2.31E-61	postive
ACADVL	AC104964.3	0.53430893	4.06E-41	postive
ALOX12	NCBP2-AS1	0.61039915	2.47E-56	postive
ACOT6	AL031768.1	0.51344068	1.38E-37	postive
HSD17B3	MROCKI	0.53596315	2.08E-41	postive
D2HGDH	MROCKI	0.50892782	7.46E-37	postive
ACOT6	AL118556.1	0.67074818	1.04E-71	postive
CPT1B	AL031846.2	0.50006951	1.90E-35	postive
D2HGDH	AL031846.2	0.52835585	4.38E-40	postive

ALOX12	AL031846.2	0.77734747	3.53E-110	postive
HSD17B3	AC025171.4	0.61191134	1.12E-56	postive
ACSBG2	AC025171.4	0.59390308	1.10E-52	postive
ACSBG2	CHRM3-AS1	0.5084066	9.05E-37	postive
TBXAS1	AL133371.2	0.59480552	7.01E-53	postive
D2HGDH	LINC01569	0.51504986	7.53E-38	postive
ACOT6	LINC00472	0.84130195	1.42E-145	postive
BMPR1B	LINC00472	0.64763939	2.08E-65	postive
CBR4	LINC00472	0.51458019	9.00E-38	postive
CPT1B	AL359921.1	0.5331549	6.47E-41	postive
D2HGDH	AL359921.1	0.50499856	3.17E-36	postive
ALOX12	AL359921.1	0.70366834	9.72E-82	postive
ELOVL2	AL121929.2	0.56153076	4.12E-46	postive
ACSBG2	AL121929.2	0.62213648	4.62E-59	postive
CYP4B1	AL121929.2	0.61340513	5.07E-57	postive
FASN	AL121929.2	0.5381375	8.59E-42	postive
ELOVL2	AC008663.1	0.89618842	1.14E-191	postive
ACSBG2	AC008663.1	0.58900984	1.21E-51	postive
CYP4B1	AC008663.1	0.89765397	3.08E-193	postive
FASN	AC008663.1	0.85280712	1.25E-153	postive
ELOVL2	WT1-AS	0.70254336	2.26E-81	postive
CYP4B1	WT1-AS	0.61158618	1.32E-56	postive

FASN	WT1-AS	0.63358459	7.75E-62	postive
ALOX12	AL157392.3	0.66667902	1.47E-70	postive
SDHD	FOXP4-AS1	0.52791082	5.22E-40	postive
PHYH	FOXP4-AS1	0.53997926	4.04E-42	postive
HACD3	FOXP4-AS1	0.57426932	1.30E-48	postive
ELOVL4	FOXP4-AS1	0.57029291	8.07E-48	postive
ACOT6	HCFC1-AS1	0.62311058	2.71E-59	postive
ALOX12	AC011005.4	0.52068671	8.75E-39	postive
ELOVL2	SNED1-AS1	0.81263287	5.02E-128	postive
ACSBG2	SNED1-AS1	0.69790348	6.96E-80	postive
CYP4B1	SNED1-AS1	0.87079356	1.04E-167	postive
FASN	SNED1-AS1	0.79473206	1.56E-118	postive
HSD17B3	AC034236.2	0.50974426	5.51E-37	postive
CPT1B	AC034236.2	0.6752264	5.36E-73	postive
D2HGDH	AC034236.2	0.59366819	1.23E-52	postive
HSD17B3	AC048341.2	0.54749223	1.77E-43	postive
CPT1B	AC048341.2	0.59062218	5.50E-52	postive
ALOX12	AC009148.1	0.60457061	5.08E-55	postive
ELOVL2	AL022311.1	0.7416685	3.43E-95	postive
ACSBG2	AL022311.1	0.74357087	6.30E-96	postive
CYP4B1	AL022311.1	0.78838971	2.15E-115	postive

FASN	AL022311.1	0.72623303	1.87E-89	postive
ELOVL2	AL354809.1	0.64995732	5.14E-66	postive
ACSBG2	AL354809.1	0.67755154	1.13E-73	postive
CYP4B1	AL354809.1	0.68747464	1.23E-76	postive
FASN	AL354809.1	0.62669654	3.74E-60	postive
HSD17B3	AC015660.3	0.68118956	9.53E-75	postive
ACADVL	AC015660.3	0.50566105	2.49E-36	postive
ELOVL2	AC013724.1	0.79752746	5.95E-120	postive
ACSBG2	AC013724.1	0.63171659	2.24E-61	postive
CYP4B1	AC013724.1	0.86863356	6.53E-166	postive
FASN	AC013724.1	0.80707331	5.69E-125	postive
CYP4B1	FAM160A1-DT	0.53182678	1.10E-40	postive
ALOX12	AP000350.6	0.54405626	7.46E-43	postive
BMPR1B	PGM5-AS1	0.54168775	2.00E-42	postive
RDH11	PGM5-AS1	0.6241094	1.56E-59	postive
D2HGDH	AL139246.3	0.51547345	6.42E-38	postive
PRKAG2	PLBD1-AS1	0.53640874	1.74E-41	postive
HACD3	PLBD1-AS1	0.59282385	1.87E-52	postive
AUH	PLBD1-AS1	0.54542373	4.22E-43	postive
HACL1	PLBD1-AS1	0.54774199	1.59E-43	postive
BMPR1B	PLBD1-AS1	0.60442918	5.46E-55	postive

RDH11	PLBD1-AS1	0.54250102	1.42E-42	postive
GABARAPL1	PLBD1-AS1	0.61728293	6.41E-58	postive
ACACB	PLBD1-AS1	0.5817539	3.92E-50	postive
ELOVL2	AL354892.3	0.63000221	5.89E-61	postive
ALOX12	AL354892.3	0.61111839	1.69E-56	postive
ACSBG2	AL354892.3	0.69376485	1.40E-78	postive
CYP4B1	AL354892.3	0.67662985	2.10E-73	postive
FASN	AL354892.3	0.61154073	1.36E-56	postive
ACOT6	AL031716.1	0.74327713	8.19E-96	postive
BMPR1B	AL031716.1	0.50736564	1.33E-36	postive
D2HGDH	Z97653.1	0.58249431	2.76E-50	postive
ALOX12	AP003392.1	0.73935876	2.63E-94	postive
ALOX12	AC005154.4	0.59156175	3.47E-52	postive
ALOX12	MIR34AHG	0.50070904	1.51E-35	postive
ELOVL2	AL512770.1	0.52977035	2.50E-40	postive
ALOX12	AL512770.1	0.63974201	2.23E-63	postive
ACSBG2	AL512770.1	0.61299294	6.31E-57	postive
CYP4B1	AL512770.1	0.54213424	1.66E-42	postive
FASN	AL512770.1	0.52651399	9.06E-40	postive
ACSBG2	AL358075.1	0.6014425	2.51E-54	postive
CYP4B1	AL358075.1	0.51052275	4.12E-37	postive

CPT1B	AC010245.2	0.52121261	7.14E-39	postive
ELOVL2	PLS3-AS1	0.54466936	5.78E-43	postive
CYP4B1	PLS3-AS1	0.57023352	8.29E-48	postive
ACOT6	PLS3-AS1	0.56592522	5.81E-47	postive
BMPR1B	PLS3-AS1	0.51987922	1.19E-38	postive
FASN	PLS3-AS1	0.54062088	3.10E-42	postive
ACBD5	AC011477.2	0.52342516	3.03E-39	postive
PRKAA2	AC011477.2	0.53945851	5.00E-42	postive
CBR4	AC011477.2	0.57993793	9.25E-50	postive
ELOVL2	AC087878.1	0.75586624	7.62E-101	postive
ACSBG2	AC087878.1	0.62543792	7.52E-60	postive
CYP4B1	AC087878.1	0.81613789	5.27E-130	postive
FASN	AC087878.1	0.76683916	1.74E-105	postive
ALOX12	AC078906.1	0.5218829	5.51E-39	postive
ALOX12	AC009119.2	0.53296596	6.98E-41	postive
ELOVL2	AC114321.1	0.83605675	4.10E-142	postive
ACSBG2	AC114321.1	0.65006394	4.82E-66	postive
CYP4B1	AC114321.1	0.89778206	2.24E-193	postive
FASN	AC114321.1	0.83212344	1.35E-139	postive
ELOVL2	AC245014.3	0.76173627	2.69E-103	postive
ACSBG2	AC245014.3	0.69223434	4.21E-78	postive

CYP4B1	AC245014.3	0.82897658	1.25E-137	postive
FASN	AC245014.3	0.74927496	3.58E-98	postive
ELOVL2	AC096642.1	0.72030574	2.36E-87	postive
ALOX12	AC096642.1	0.52845109	4.22E-40	postive
ACSBG2	AC096642.1	0.72811672	3.93E-90	postive
CYP4B1	AC096642.1	0.78324389	6.32E-113	postive
FASN	AC096642.1	0.70356438	1.05E-81	postive
ELOVL2	AC100793.2	0.81617306	5.04E-130	postive
ACSBG2	AC100793.2	0.64339898	2.60E-64	postive
CYP4B1	AC100793.2	0.87372831	3.29E-170	postive
FASN	AC100793.2	0.8098658	1.71E-126	postive
ALOX12	AL031670.1	0.63047458	4.52E-61	postive
CPT1B	AL122125.1	0.51421198	1.03E-37	postive
D2HGDH	AL122125.1	0.51567296	5.95E-38	postive
GABARAPL1	LINC02432	0.5960041	3.86E-53	postive
CPT1B	AC008105.2	0.50236105	8.29E-36	postive
ALOX12	AC008105.2	0.51653166	4.29E-38	postive
KMT5A	AC008105.2	0.50121007	1.26E-35	postive
ACOT6	NCOA7-AS1	0.83761584	3.95E-143	postive
BMPR1B	NCOA7-AS1	0.83846697	1.09E-143	postive
RDH11	NCOA7-AS1	0.50366286	5.17E-36	postive

ACACB	NCOA7-AS1	0.52152797	6.32E-39	postive
ELOVL2	AC005616.1	0.83284164	4.73E-140	postive
ACSBG2	AC005616.1	0.68281863	3.12E-75	postive
CYP4B1	AC005616.1	0.88881172	4.09E-184	postive
FASN	AC005616.1	0.82315371	4.28E-134	postive
ACOT6	SDCBP2-AS1	0.56781578	2.48E-47	postive
ALOX12	AL161725.1	0.57394386	1.51E-48	postive
D2HGDH	AC004832.6	0.50702772	1.51E-36	postive
ELOVL2	GTF3C2-AS1	0.77661931	7.60E-110	postive
ACSBG2	GTF3C2-AS1	0.72677284	1.20E-89	postive
CYP4B1	GTF3C2-AS1	0.83437662	4.96E-141	postive
FASN	GTF3C2-AS1	0.76791986	5.87E-106	postive
ALOX12	AL031282.2	0.71399612	3.55E-85	postive
ACSBG2	AL031282.2	0.51224219	2.17E-37	postive
CBR4	AC012467.2	0.51236466	2.07E-37	postive
ELOVL2	AL024508.1	0.74225892	2.03E-95	postive
ACSBG2	AL024508.1	0.61457218	2.73E-57	postive
CYP4B1	AL024508.1	0.81840295	2.64E-131	postive
FASN	AL024508.1	0.7489853	4.67E-98	postive
HSD17B3	AC005387.1	0.52538399	1.41E-39	postive
CPT1B	AC005387.1	0.62732284	2.64E-60	postive

D2HGDH	AC005387.1	0.61634323	1.06E-57	postive
ACADVL	AC005387.1	0.52444305	2.04E-39	postive
HACL1	ZNF793-AS1	0.52167806	5.97E-39	postive
RDH11	ZNF793-AS1	0.53866007	6.93E-42	postive
SDHD	MIR200CHG	0.66310952	1.45E-69	postive
PHYH	MIR200CHG	0.69328396	1.98E-78	postive
NDUFAB1	MIR200CHG	0.59946495	6.82E-54	postive
HACD3	MIR200CHG	0.69826518	5.34E-80	postive
ELOVL4	MIR200CHG	0.80513985	6.23E-124	postive
ALOX12	AL008729.1	0.66116268	5.00E-69	postive
ACSBG2	AL008729.1	0.64819312	1.49E-65	postive
CYP4B1	AL008729.1	0.51678691	3.90E-38	postive
ALOX12	STARD4-AS1	0.61956103	1.88E-58	postive
ACSBG2	MRTFA-AS1	0.5248761	1.72E-39	postive
ELOVL2	AC006111.3	0.83128403	4.55E-139	postive
ACSBG2	AC006111.3	0.70447629	5.30E-82	postive
CYP4B1	AC006111.3	0.89021645	1.64E-185	postive
FASN	AC006111.3	0.82145218	4.37E-133	postive
HSD17B3	AC005306.1	0.54241171	1.48E-42	postive
CPT1B	AC005306.1	0.7233106	2.07E-88	postive
D2HGDH	AC005306.1	0.68605539	3.31E-76	postive

ACADVL	AC005306.1	0.59055267	5.69E-52	postive
ALOX12	AC005306.1	0.53310866	6.59E-41	postive
ALOX12	AC091887.1	0.59794666	1.46E-53	postive
ACSBG2	AC091887.1	0.59693332	2.43E-53	postive
ACOT6	SNRK-AS1	0.68975239	2.46E-77	postive
BMPR1B	SNRK-AS1	0.79501096	1.13E-118	postive
ELOVL4	SNRK-AS1	0.52008109	1.10E-38	postive
RDH11	SNRK-AS1	0.63041147	4.68E-61	postive
GABARAPL1	SNRK-AS1	0.52072941	8.61E-39	postive
ACACB	SNRK-AS1	0.64585554	6.05E-65	postive
ALOX12	AC022973.5	0.53112471	1.46E-40	postive
ACOT6	MECOM-AS1	0.88752294	7.52E-183	postive
BMPR1B	MECOM-AS1	0.69228854	4.05E-78	postive
ACOT6	AC012404.1	0.53857556	7.18E-42	postive
CPT1B	MRPS9-AS1	0.54774011	1.59E-43	postive
ALOX12	MRPS9-AS1	0.56885694	1.55E-47	postive
ENO3	LINC01942	0.52425735	2.19E-39	postive
ACOT8	AC073896.2	0.62219079	4.48E-59	postive
PHYH	AC073896.2	0.50332584	5.84E-36	postive
NDUFAB1	AC073896.2	0.58138296	4.68E-50	postive
ELOVL4	AC073896.2	0.5455062	4.07E-43	postive

ELOVL2	SAP30L-AS1	0.7236337	1.59E-88	postive
ACSBG2	SAP30L-AS1	0.7059123	1.79E-82	postive
CYP4B1	SAP30L-AS1	0.78805538	3.12E-115	postive
FASN	SAP30L-AS1	0.7127609	9.31E-85	postive
ELOVL2	AP000873.3	0.67308827	2.22E-72	postive
ALOX12	AP000873.3	0.56657783	4.34E-47	postive
ACSBG2	AP000873.3	0.70169449	4.25E-81	postive
CYP4B1	AP000873.3	0.72821782	3.61E-90	postive
FASN	AP000873.3	0.64421593	1.60E-64	postive
ALOX12	Z98885.3	0.59318952	1.56E-52	postive
ACSBG2	Z98885.3	0.5669813	3.62E-47	postive
ELOVL2	RNF216-IT1	0.66892282	3.43E-71	postive
ACSBG2	RNF216-IT1	0.67841499	6.29E-74	postive
CYP4B1	RNF216-IT1	0.72711363	9.04E-90	postive
FASN	RNF216-IT1	0.65322664	7.00E-67	postive
ELOVL2	AL391261.1	0.62607612	5.28E-60	postive
ACSBG2	AL391261.1	0.50362552	5.24E-36	postive
CYP4B1	AL391261.1	0.69173305	6.02E-78	postive
FASN	AL391261.1	0.62947887	7.91E-61	postive
KMT5A	AC079922.2	0.5366502	1.57E-41	postive
ELOVL2	AC117490.2	0.77379414	1.45E-108	postive
ACSBG2	AC117490.2	0.72198434	6.08E-88	postive
CYP4B1	AC117490.2	0.80909724	4.52E-126	postive

FASN	AC117490.2	0.75313045	1.00E-99	postive
ACSBG1	AC010904.2	0.56018174	7.47E-46	postive
ACOT6	AC064801.1	0.73783564	9.94E-94	postive
BMPR1B	AC064801.1	0.52399595	2.43E-39	postive
ACOT6	SIAH2-AS1	0.72096875	1.38E-87	postive
BMPR1B	SIAH2-AS1	0.71761374	2.04E-86	postive
ELOVL2	AC090907.2	0.595888	4.09E-53	postive
ACSBG2	AC090907.2	0.56025457	7.23E-46	postive
CYP4B1	AC090907.2	0.6284786	1.38E-60	postive
FASN	AC090907.2	0.55426651	9.83E-45	postive
ELOVL2	AP000763.3	0.63560811	2.43E-62	postive
ACSBG2	AP000763.3	0.65777619	4.19E-68	postive
CYP4B1	AP000763.3	0.68642614	2.55E-76	postive
FASN	AP000763.3	0.6174888	5.74E-58	postive
ELOVL2	LINC02812	0.82161955	3.48E-133	postive
ACSBG2	LINC02812	0.66566768	2.82E-70	postive
CYP4B1	LINC02812	0.88748797	8.13E-183	postive
FASN	LINC02812	0.82425373	9.41E-135	postive
ELOVL2	AL357497.1	0.83088161	8.14E-139	postive
ACSBG2	AL357497.1	0.649536	6.63E-66	postive
CYP4B1	AL357497.1	0.89361539	5.69E-189	postive
FASN	AL357497.1	0.82586651	1.00E-135	postive

ELOVL2	EDIL3-DT	0.68289464	2.96E-75	postive
ACSBG2	EDIL3-DT	0.57183374	3.99E-48	postive
CYP4B1	EDIL3-DT	0.69558965	3.76E-79	postive
FASN	EDIL3-DT	0.66781228	7.06E-71	postive
PRKAA2	AC098484.1	0.70528057	2.89E-82	postive
ACADM	AC098484.1	0.52635275	9.65E-40	postive
SERINC1	AC098484.1	0.56229969	2.93E-46	postive
MMAA	AC098484.1	0.52567089	1.26E-39	postive
ALOX12	AC053513.2	0.57345342	1.90E-48	postive
ACAA2	DOCK8-AS1	0.52228814	4.71E-39	postive
ACADL	DOCK8-AS1	0.50565958	2.49E-36	postive
CPT1B	AC253536.6	0.52138879	6.67E-39	postive
ACOT6	AC253536.6	0.61826187	3.78E-58	postive
ACACB	AC253536.6	0.53478522	3.35E-41	postive
ACOT6	AL353804.1	0.64908178	8.72E-66	postive
ELOVL2	AC098487.1	0.68673507	2.06E-76	postive
ACSBG2	AC098487.1	0.56436301	1.17E-46	postive
CYP4B1	AC098487.1	0.69617534	2.46E-79	postive
FASN	AC098487.1	0.65536152	1.88E-67	postive
ELOVL2	AL136141.1	0.52536237	1.42E-39	postive
ACSBG2	AL136141.1	0.5442231	6.96E-43	postive

CYP4B1	AL136141.1	0.5640433	1.35E-46	postive
HSD17B3	AL353622.1	0.54627926	2.94E-43	postive
CPT1B	AL353622.1	0.53321605	6.31E-41	postive
D2HGDH	AL353622.1	0.61854551	3.25E-58	postive
ACADVL	AL353622.1	0.52617352	1.04E-39	postive
ALOX12	AL353622.1	0.52438529	2.08E-39	postive
D2HGDH	AC012366.1	0.52411546	2.32E-39	postive
ALOX12	AC012366.1	0.50378757	4.94E-36	postive
CPT1B	NFYC-AS1	0.61322008	5.59E-57	postive
D2HGDH	NFYC-AS1	0.56443125	1.13E-46	postive
ALOX12	NFYC-AS1	0.73688145	2.28E-93	postive
CPT1B	AC136475.3	0.52409472	2.33E-39	postive
D2HGDH	AC136475.3	0.51702774	3.56E-38	postive
HSD17B3	AL158834.2	0.52612171	1.06E-39	postive
CPT1B	AL158834.2	0.52249755	4.34E-39	postive
ALOX12	AL158834.2	0.58298945	2.18E-50	postive
ACOT6	AC016590.2	0.71390394	3.81E-85	postive
BMPR1B	AC016590.2	0.5111686	3.24E-37	postive
ELOVL2	AL050327.1	0.73472668	1.46E-92	postive
ACSBG2	AL050327.1	0.56591315	5.84E-47	postive
CYP4B1	AL050327.1	0.76327601	5.95E-104	postive

FASN	AL050327.1	0.74753913	1.75E-97	postive
ALOX12	AC091057.1	0.51201169	2.36E-37	postive
PTGS2	AL391056.1	0.52590918	1.15E-39	postive
ELOVL2	AC008946.1	0.67323809	2.01E-72	postive
ACSBG2	AC008946.1	0.56222569	3.03E-46	postive
CYP4B1	AC008946.1	0.74126797	4.88E-95	postive
FASN	AC008946.1	0.70345779	1.14E-81	postive
D2HGDH	AL590822.1	0.55123502	3.61E-44	postive
FAAH	AL590822.1	0.52769711	5.68E-40	postive
ACSBG2	AL590822.1	0.51338006	1.41E-37	postive
ACOT6	LINC00216	0.66349805	1.13E-69	postive
ALOX12	AC025043.1	0.55594469	4.76E-45	postive
SDHD	AC004870.4	0.50597138	2.22E-36	postive
CPT1B	AL671710.1	0.67070125	1.07E-71	postive
D2HGDH	AL671710.1	0.54689967	2.27E-43	postive
SUCLG1	AP000640.1	0.59925636	7.58E-54	postive
ELOVL2	Z69666.1	0.66588237	2.46E-70	postive
ACSBG2	Z69666.1	0.71275215	9.37E-85	postive
CYP4B1	Z69666.1	0.72787581	4.80E-90	postive
FASN	Z69666.1	0.6484991	1.24E-65	postive
CPT1B	AC003070.1	0.63164283	2.33E-61	postive

D2HGDH	AC003070.1	0.59495093	6.52E-53	postive
ALOX12	AC003070.1	0.68459304	9.15E-76	postive
ALOX12	DCUN1D2-AS	0.59994336	5.36E-54	postive
ALOX12	AP001094.2	0.6591491	1.78E-68	postive
ALOX12	LINC00427	0.54206889	1.70E-42	postive
PTGS2	AC148477.3	0.60073513	3.59E-54	postive
ALOX12	Z98884.1	0.57247405	2.97E-48	postive
ELOVL2	ARF4-AS1	0.67425309	1.03E-72	postive
ACSBG2	ARF4-AS1	0.57960847	1.08E-49	postive
CYP4B1	ARF4-AS1	0.69003212	2.02E-77	postive
FASN	ARF4-AS1	0.66535184	3.46E-70	postive
ELOVL2	AC026474.1	0.81484575	2.86E-129	postive
ACSBG2	AC026474.1	0.67800579	8.30E-74	postive
CYP4B1	AC026474.1	0.8827774	2.53E-178	postive
FASN	AC026474.1	0.8083392	1.17E-125	postive
ACSBG2	AC106795.2	0.50385503	4.82E-36	postive
ACOT8	SNHG11	0.64494651	1.04E-64	postive
ELOVL2	AC024257.3	0.50981103	5.38E-37	postive
ALOX12	AC024257.3	0.62771913	2.12E-60	postive
ACSBG2	AC024257.3	0.66277491	1.80E-69	postive
CYP4B1	AC024257.3	0.58559859	6.27E-51	postive
CPT1B	AC007292.1	0.5961607	3.57E-53	postive

D2HGDH	AC007292.1	0.76956386	1.11E-106	postive
ACADVL	AC007292.1	0.59942309	6.97E-54	postive
ALOX12	AC007292.1	0.56568075	6.49E-47	postive
ACOT6	FLNB-AS1	0.51993122	1.17E-38	postive
ELOVL2	AC011481.1	0.61882855	2.79E-58	postive
ALOX12	AC011481.1	0.56289499	2.25E-46	postive
ACSBG2	AC011481.1	0.65951684	1.41E-68	postive
CYP4B1	AC011481.1	0.6849925	6.93E-76	postive
FASN	AC011481.1	0.61804228	4.26E-58	postive
HSD17B3	AC026333.4	0.52801437	5.01E-40	postive
CPT1B	AC026333.4	0.51832461	2.17E-38	postive
ALOX12	ASH1L-AS1	0.52033841	1.00E-38	postive
ALOX12	AL162274.1	0.61787936	4.65E-58	postive
HSD17B3	LINC00115	0.53044068	1.92E-40	postive
CPT1B	LINC00115	0.61097596	1.83E-56	postive
D2HGDH	LINC00115	0.60310838	1.07E-54	postive
ALOX12	LINC00115	0.63559949	2.45E-62	postive
ALOX12	AC073912.1	0.59341634	1.39E-52	postive
ALOX12	AC018690.1	0.55750332	2.41E-45	postive
HSD17B3	AL513320.1	0.5717509	4.14E-48	postive
CPT1B	AL513320.1	0.59960813	6.34E-54	postive

D2HGDH	AL513320.1	0.67656582	2.19E-73	postive
ACADVL	AL513320.1	0.55400169	1.10E-44	postive
ALOX12	AL513320.1	0.53702564	1.35E-41	postive
ACOT6	SAMD12-AS1	0.71862806	9.07E-87	postive
BMPR1B	SAMD12-AS1	0.92506176	6.33E-228	postive
RDH11	SAMD12-AS1	0.67071725	1.06E-71	postive
ACACB	SAMD12-AS1	0.51004445	4.93E-37	postive
ELOVL2	AL359397.1	0.52837276	4.35E-40	postive
CYP4B1	AL359397.1	0.61549169	1.67E-57	postive
FASN	AL359397.1	0.53790881	9.43E-42	postive
ELOVL2	AL607028.1	0.59855621	1.08E-53	postive
ACSBG2	AL607028.1	0.6425118	4.39E-64	postive
CYP4B1	AL607028.1	0.67111461	8.18E-72	postive
FASN	AL607028.1	0.59110569	4.34E-52	postive
ACAA2	AC020779.2	0.50236883	8.27E-36	postive
ELOVL2	LINC00299	0.53122264	1.40E-40	postive
ALOX12	LINC00299	0.55192555	2.69E-44	postive
ACSBG2	LINC00299	0.61762505	5.33E-58	postive
CYP4B1	LINC00299	0.56758715	2.75E-47	postive
ALOX12	AC025430.1	0.52207433	5.12E-39	postive
ACSBG2	AC025430.1	0.54133299	2.31E-42	postive

ELOVL2	AC009226.1	0.5327100<br>4	7.73E-41	postive
ACSBG2	AC009226.1	0.5755916	7.06E-49	postive
CYP4B1	AC009226.1	0.5405084<br>8	3.25E-42	postive
FASN	AC009226.1	0.5886308<br>6	1.45E-51	postive
ALOX12	LINC00476	0.5225167<br>7	4.31E-39	postive
ACSBG2	LINC00476	0.5301179<br>4	2.18E-40	postive
CYP4B1	LINC00476	0.5478705<br>5	1.51E-43	postive
ALOX12	AC112496.1	0.5653115<br>3	7.65E-47	postive
ACSBG2	AC112496.1	0.5826425<br>6	2.57E-50	postive
HSD17B3	MRPL20-DT	0.5089033<br>7	7.53E-37	postive
CPT1B	MRPL20-DT	0.5779758<br>7	2.32E-49	postive
D2HGDH	MRPL20-DT	0.5735311<br>3	1.83E-48	postive
ALOX12	MRPL20-DT	0.5500126<br>5	6.08E-44	postive
ACOT6	LINC02006	0.9182507<br>4	3.43E-218	postive
BMPR1B	LINC02006	0.7471610<br>7	2.47E-97	postive
ELOVL2	AC007216.3	0.7265207<br>9	1.48E-89	postive
ACSBG2	AC007216.3	0.6743075<br>6	9.90E-73	postive
CYP4B1	AC007216.3	0.7805678<br>9	1.14E-111	postive
FASN	AC007216.3	0.7056321<br>6	2.22E-82	postive
ALOX12	AC118344.1	0.5740478<br>9	1.44E-48	postive
ACOT6	AC118344.1	0.6016721	2.23E-54	postive

CYP4B1	AC027449.1	0.54953763	7.44E-44	postive
ACOT8	UBAC2-AS1	0.52231128	4.67E-39	postive
SDHD	UBAC2-AS1	0.57303921	2.29E-48	postive
PHYH	UBAC2-AS1	0.55675441	3.35E-45	postive
NDUFAB1	UBAC2-AS1	0.50200954	9.42E-36	postive
HACD3	UBAC2-AS1	0.56698281	3.61E-47	postive
HACL1	UBAC2-AS1	0.55228741	2.30E-44	postive
ELOVL4	UBAC2-AS1	0.51673141	3.98E-38	postive
ALOX12	LINC00937	0.51056423	4.06E-37	postive
D2HGDH	AL606760.2	0.5257356	1.23E-39	postive
ALOX12	AL606760.2	0.64164614	7.31E-64	postive
ACSBG2	AL606760.2	0.60329088	9.78E-55	postive
ACSBG2	AC072061.1	0.52381266	2.61E-39	postive
CYP4B1	AC072061.1	0.54911757	8.89E-44	postive
ELOVL2	AC092718.4	0.54831904	1.25E-43	postive
ACSBG2	AC092718.4	0.57712121	3.46E-49	postive
CYP4B1	AC092718.4	0.58153438	4.35E-50	postive
FASN	AC092718.4	0.56977701	1.02E-47	postive
CPT1B	ASB16-AS1	0.5937767	1.17E-52	postive
D2HGDH	ASB16-AS1	0.57598849	5.87E-49	postive
ALOX12	ASB16-AS1	0.5706704	6.79E-48	postive
HSD17B3	LINC01355	0.56448037	1.11E-46	postive

CPT1B	LINC01355	0.5451959	4.64E-43	postive
ALOX12	LINC01355	0.58160594	4.21E-50	postive
HSD17B3	AC125494.2	0.57899633	1.44E-49	postive
D2HGDH	AC125494.2	0.50754833	1.24E-36	postive
PRKAG2	AL031710.1	0.52615697	1.04E-39	postive
ACOT8	AL031710.1	0.59381313	1.15E-52	postive
SDHD	AL031710.1	0.66874763	3.85E-71	postive
PHYH	AL031710.1	0.6437152	2.16E-64	postive
PDHB	AL031710.1	0.50574575	2.41E-36	postive
NDUFAB1	AL031710.1	0.63599023	1.95E-62	postive
HACD3	AL031710.1	0.70022895	1.26E-80	postive
AUH	AL031710.1	0.60802877	8.51E-56	postive
HACL1	AL031710.1	0.52690319	7.77E-40	postive
ELOVL4	AL031710.1	0.72700036	9.93E-90	postive
RDH11	AL031710.1	0.57726555	3.24E-49	postive
GABARAPL1	AL031710.1	0.60671898	1.68E-55	postive
ALOX12	AC068987.2	0.63654321	1.42E-62	postive
ACSBG2	AC068987.2	0.50418601	4.27E-36	postive
ELOVL2	AC103810.5	0.66576063	2.66E-70	postive
ACSBG2	AC103810.5	0.65561835	1.60E-67	postive
CYP4B1	AC103810.5	0.73629132	3.80E-93	postive

FASN	AC103810.5	0.65146875	2.05E-66	postive
PRKAG2	AC118345.1	0.58960381	9.03E-52	postive
PDHB	AC118345.1	0.55804509	1.91E-45	postive
BMPR1B	AC118345.1	0.5198326	1.22E-38	postive
GABARAPL1	AC118345.1	0.77897859	6.25E-111	postive
ACACB	AC118345.1	0.55749924	2.42E-45	postive
ALOX12	AL355297.3	0.5676739	2.65E-47	postive
HSD17B3	AC015802.5	0.57834161	1.96E-49	postive
D2HGDH	AC015802.5	0.51236706	2.07E-37	postive
CPT1B	AC022167.2	0.56612316	5.32E-47	postive
D2HGDH	AC022167.2	0.5821928	3.19E-50	postive
ALOX12	AC022167.2	0.63382534	6.75E-62	postive
ELOVL2	AC105105.4	0.65691448	7.17E-68	postive
ACSBG2	AC105105.4	0.62002713	1.46E-58	postive
CYP4B1	AC105105.4	0.71960043	4.16E-87	postive
FASN	AC105105.4	0.66598397	2.30E-70	postive
ELOVL2	LINC02585	0.80588059	2.50E-124	postive
ACSBG2	LINC02585	0.67546239	4.58E-73	postive
CYP4B1	LINC02585	0.87392963	2.21E-170	postive
FASN	LINC02585	0.81283799	3.86E-128	postive
ALOX12	AC002064.2	0.57398359	1.49E-48	postive
ACSBG2	AC002064.2	0.64338261	2.63E-64	postive

CYP4B1	AC002064.2	0.54305691	1.13E-42	postive
ACOT6	AL035071.2	0.66752339	8.52E-71	postive
ACAA2	LINC01843	0.55608685	4.47E-45	postive
ALOX12	AC027796.1	0.59401803	1.04E-52	postive
ACSBG2	AC027796.1	0.62824426	1.58E-60	postive
CYP4B1	AC027796.1	0.52613947	1.05E-39	postive
ELOVL2	LINC02453	0.81134356	2.62E-127	postive
ACSBG2	LINC02453	0.64208913	5.63E-64	postive
CYP4B1	LINC02453	0.87081868	9.87E-168	postive
FASN	LINC02453	0.81894187	1.28E-131	postive
ELOVL2	AL133551.1	0.74212126	2.29E-95	postive
ACSBG2	AL133551.1	0.7378343	9.95E-94	postive
CYP4B1	AL133551.1	0.79084131	1.35E-116	postive
FASN	AL133551.1	0.74711428	2.58E-97	postive
CPT1B	ARRDC1-AS1	0.67107597	8.39E-72	postive
ELOVL2	AC011825.4	0.77629284	1.07E-109	postive
ACSBG2	AC011825.4	0.65089756	2.90E-66	postive
CYP4B1	AC011825.4	0.84438074	1.15E-147	postive
FASN	AC011825.4	0.76303175	7.56E-104	postive
PRKAG2	GAS6-DT	0.59858869	1.06E-53	postive
SDHD	GAS6-DT	0.5659231	5.82E-47	postive

PHYH	GAS6-DT	0.51589329	5.47E-38	postive
PDHB	GAS6-DT	0.60221498	1.69E-54	postive
GCDH	GAS6-DT	0.51057666	4.04E-37	postive
HACD3	GAS6-DT	0.63410613	5.75E-62	postive
AUH	GAS6-DT	0.6121885	9.65E-57	postive
HACL1	GAS6-DT	0.61137336	1.48E-56	postive
BMPR1B	GAS6-DT	0.67083885	9.80E-72	postive
ELOVL4	GAS6-DT	0.66828753	5.19E-71	postive
RDH11	GAS6-DT	0.68314471	2.49E-75	postive
GABARAPL1	GAS6-DT	0.67680723	1.86E-73	postive
ACACB	GAS6-DT	0.62176902	5.65E-59	postive
ACOT8	AC133552.5	0.50022474	1.80E-35	postive
NDUFAB1	AC133552.5	0.53877413	6.62E-42	postive
CYP4B1	DSG2-AS1	0.57561337	6.99E-49	postive
ACOT6	DSG2-AS1	0.57408287	1.42E-48	postive
BMPR1B	DSG2-AS1	0.55553739	5.67E-45	postive
PRKAA2	AC124854.1	0.50757467	1.23E-36	postive
ELOVL2	ZNF114-AS1	0.60196569	1.92E-54	postive
CYP4B1	ZNF114-AS1	0.55929064	1.10E-45	postive
FASN	ZNF114-AS1	0.57003984	9.05E-48	postive
ELOVL2	AC024941.2	0.59776266	1.60E-53	postive

ACSBG2	AC024941.2	0.5015493	1.11E-35	postive
CYP4B1	AC024941.2	0.62730199	2.67E-60	postive
FASN	AC024941.2	0.5465664	2.61E-43	postive
ELOVL2	AL592301.1	0.7449799	1.78E-96	postive
ACSBG2	AL592301.1	0.69561927	3.68E-79	postive
CYP4B1	AL592301.1	0.80476111	9.92E-124	postive
FASN	AL592301.1	0.73740217	1.45E-93	postive
ALOX12	AC066613.1	0.6872684	1.42E-76	postive
ACOT6	AL353804.2	0.52632741	9.74E-40	postive
ACOT6	AC010333.2	0.7668178	1.77E-105	postive
BMPR1B	AC010333.2	0.734829	1.34E-92	postive
ACACB	AC010333.2	0.52714284	7.07E-40	postive
ALOX12	AC025171.2	0.59692925	2.43E-53	postive
ACOT6	FAM198B-AS1	0.823546	2.50E-134	postive
BMPR1B	FAM198B-AS1	0.80214151	2.41E-122	postive
RDH11	FAM198B-AS1	0.50244041	8.06E-36	postive
ELOVL2	LINC02427	0.51551838	6.31E-38	postive
CYP4B1	LINC02427	0.54544495	4.18E-43	postive
ACSBG1	LINC02427	0.62181013	5.52E-59	postive
ENO3	LINC02427	0.62979479	6.62E-61	postive
PCBD1	LINC02427	0.52503149	1.62E-39	postive
FASN	LINC02427	0.52703186	7.39E-40	postive
ELOVL2	AC025871.2	0.63605098	1.89E-62	postive
ACSBG2	AC025871.2	0.73442929	1.89E-92	postive

CYP4B1	AC025871.2	0.67615703	2.88E-73	postive
FASN	AC025871.2	0.60442719	5.47E-55	postive
HSD17B3	ZNF213-AS1	0.51488701	8.01E-38	postive
CPT1B	ZNF213-AS1	0.64532712	8.29E-65	postive
D2HGDH	ZNF213-AS1	0.69029628	1.67E-77	postive
ALOX12	ZNF213-AS1	0.63981563	2.13E-63	postive
ELOVL2	AL591043.2	0.63714124	1.01E-62	postive
ACSBG2	AL591043.2	0.63039674	4.72E-61	postive
CYP4B1	AL591043.2	0.68427681	1.14E-75	postive
FASN	AL591043.2	0.63927376	2.92E-63	postive
ALOX12	AC018809.2	0.57301467	2.32E-48	postive
ALOX12	AP001020.2	0.55140344	3.36E-44	postive
ACSBG2	AP001020.2	0.61419066	3.34E-57	postive
CYP4B1	AP001020.2	0.52711444	7.15E-40	postive
ALOX12	ACBD3-AS1	0.58344294	1.76E-50	postive
CPT1B	AC008735.1	0.5069106	1.57E-36	postive
ALOX12	AC008735.1	0.70987641	8.70E-84	postive
ACSBG2	AC008735.1	0.52117606	7.25E-39	postive
ACOT6	AC092127.2	0.77814189	1.52E-110	postive
BMPR1B	AC092127.2	0.57336338	1.98E-48	postive
ALOX12	AL606534.1	0.54390371	7.95E-43	postive

ACOT6	AL450384.1	0.72581095	2.66E-89	postive
BMPR1B	AL450384.1	0.50725577	1.38E-36	postive
ACOT6	AC025918.1	0.6145926	2.70E-57	postive
PTGS2	XXYLT1-AS2	0.60350899	8.75E-55	postive
ACADVL	AC015726.1	0.53272296	7.69E-41	postive
ELOVL2	AC004584.1	0.81749055	8.86E-131	postive
ACSBG2	AC004584.1	0.6971816	1.18E-79	postive
CYP4B1	AC004584.1	0.87060412	1.49E-167	postive
FASN	AC004584.1	0.81012008	1.24E-126	postive
ACOT6	AL135787.1	0.58523732	7.45E-51	postive
ALOX12	C9orf106	0.55657969	3.61E-45	postive
ACSBG2	AC010273.2	0.52424311	2.20E-39	postive
ACOT6	OCIAD1-AS1	0.60826334	7.54E-56	postive
ALOX12	AC073912.2	0.58655123	3.96E-51	postive
ALOX12	DLEU2L	0.59391127	1.09E-52	postive
ACOT6	AC016831.4	0.89197357	2.76E-187	postive
BMPR1B	AC016831.4	0.64753997	2.21E-65	postive
DPEP2	AC020658.5	0.52012326	1.09E-38	postive
ALOX12	Z94721.1	0.6553531	1.89E-67	postive
ACSBG2	Z94721.1	0.5478976	1.49E-43	postive
ALOX12	PSMD6-AS2	0.50689231	1.58E-36	postive
ACOT6	PSMD6-AS2	0.51932821	1.48E-38	postive

PRKAG2	AC008691.1	0.62628187	4.71E-60	postive
ACOT8	AC008691.1	0.53813109	8.61E-42	postive
SDHD	AC008691.1	0.63973114	2.24E-63	postive
PHYH	AC008691.1	0.59910169	8.19E-54	postive
PDHB	AC008691.1	0.57713274	3.45E-49	postive
NDUFAB1	AC008691.1	0.51078746	3.74E-37	postive
GCDH	AC008691.1	0.51507906	7.45E-38	postive
HACD3	AC008691.1	0.69893513	3.27E-80	postive
AUH	AC008691.1	0.62891107	1.09E-60	postive
HACL1	AC008691.1	0.55868071	1.44E-45	postive
BMPR1B	AC008691.1	0.59002933	7.34E-52	postive
ELOVL4	AC008691.1	0.64602971	5.45E-65	postive
RDH11	AC008691.1	0.63134743	2.76E-61	postive
GABARAPL1	AC008691.1	0.79474095	1.54E-118	postive
ACACB	AC008691.1	0.58428746	1.17E-50	postive
FASN	LINC01301	0.50900273	7.26E-37	postive
MMAA	AL589745.1	0.51181263	2.55E-37	postive
ACOT6	AC096586.2	0.75397126	4.56E-100	postive
BMPR1B	AC096586.2	0.50035386	1.71E-35	postive
CBR4	AC096586.2	0.54797616	1.44E-43	postive

ALOX12	AC110769.2	0.59824562	1.26E-53	postive
D2HGDH	AL591895.1	0.59864074	1.03E-53	postive
ACADVL	AL591895.1	0.5468816	2.29E-43	postive
ALOX12	AC008737.1	0.54239969	1.49E-42	postive
ACOT6	AC021321.1	0.749551	2.77E-98	postive
BMPR1B	AC021321.1	0.64110246	1.01E-63	postive
ELOVL2	AC126773.3	0.83206948	1.46E-139	postive
ACSBG2	AC126773.3	0.68122909	9.28E-75	postive
CYP4B1	AC126773.3	0.89220141	1.62E-187	postive
FASN	AC126773.3	0.82516465	2.66E-135	postive
DPEP2	AC002091.1	0.56292376	2.22E-46	postive
TBXAS1	AC002091.1	0.69378943	1.38E-78	postive
FAAH	AC087482.1	0.52504777	1.61E-39	postive
ALOX12	C21orf62-AS1	0.50030842	1.74E-35	postive
ACSBG2	C21orf62-AS1	0.51656997	4.23E-38	postive
CPT1B	AP001107.4	0.60123761	2.78E-54	postive
D2HGDH	AP001107.4	0.55299795	1.70E-44	postive
ALOX12	AP001107.4	0.5788654	1.53E-49	postive
ACOT6	AC027544.2	0.72777132	5.23E-90	postive
ELOVL2	AC008050.1	0.69894156	3.25E-80	postive
ACSBG2	AC008050.1	0.54690745	2.26E-43	postive
CYP4B1	AC008050.1	0.78970008	4.93E-116	postive

FASN	AC008050.1	0.64032091	1.59E-63	postive
ALOX12	AC134775.1	0.50249243	7.91E-36	postive
EHHADH	AC063919.1	0.51880582	1.80E-38	postive
ACOT6	AC078842.1	0.59571533	4.46E-53	postive
BMPR1B	AC078842.1	0.9040418	2.30E-200	postive
RDH11	AC078842.1	0.63917657	3.09E-63	postive
ELOVL2	XIAP-AS1	0.51695488	3.66E-38	postive
ACSBG2	XIAP-AS1	0.61638509	1.04E-57	postive
CYP4B1	XIAP-AS1	0.57058642	7.06E-48	postive
CPT1B	FAM13A-AS1	0.52164367	6.05E-39	postive
ALOX12	FAM13A-AS1	0.69611884	2.56E-79	postive
D2HGDH	AC004449.1	0.51273926	1.80E-37	postive
ACOT6	AC007336.1	0.71368478	4.53E-85	postive
BMPR1B	AC007336.1	0.5299929	2.29E-40	postive
ACSBG2	BICRA-AS1	0.53638363	1.75E-41	postive
CPT1B	AC004846.2	0.53968495	4.55E-42	postive
D2HGDH	AC004846.2	0.63539347	2.75E-62	postive
ACADVL	AC004846.2	0.51429606	1.00E-37	postive
ALOX12	AC004846.2	0.58104132	5.50E-50	postive
CPT1B	AC008735.2	0.73093783	3.68E-91	postive
D2HGDH	AC008735.2	0.67102921	8.65E-72	postive
ALOX12	AC008735.2	0.5450191	4.99E-43	postive

ELOVL2	AC133550.3	0.69227869	4.08E-78	postive
ALOX12	AC133550.3	0.53040254	1.94E-40	postive
ACSBG2	AC133550.3	0.72789105	4.74E-90	postive
CYP4B1	AC133550.3	0.76655898	2.30E-105	postive
FASN	AC133550.3	0.70267501	2.04E-81	postive
CBR4	GAS5-AS1	0.54067816	3.03E-42	postive
ELOVL2	AC091114.1	0.5166267	4.14E-38	postive
CYP4B1	AC091114.1	0.57845023	1.86E-49	postive
FASN	AC091114.1	0.57325222	2.08E-48	postive
ALOX12	AC022558.3	0.55415892	1.03E-44	postive
ACSBG2	AC022558.3	0.5663676	4.77E-47	postive
ACOT6	AC022558.3	0.6159405	1.32E-57	postive
HSD17B3	AC087239.1	0.509756	5.49E-37	postive
CPT1B	AC087239.1	0.50722186	1.40E-36	postive
D2HGDH	AC087239.1	0.52588089	1.16E-39	postive
ACOT6	ITGB5-AS1	0.50099118	1.36E-35	postive
ELOVL2	AP000688.1	0.62593738	5.70E-60	postive
ACSBG2	AP000688.1	0.61548218	1.68E-57	postive
CYP4B1	AP000688.1	0.69851853	4.44E-80	postive
FASN	AP000688.1	0.63437285	4.94E-62	postive
ACOT6	AC007014.2	0.53156036	1.23E-40	postive
AUH	LINC02762	0.51148238	2.88E-37	postive

BMPR1B	LINC02762	0.55204285	2.56E-44	postive
ALOX12	GK-AS1	0.58270917	2.49E-50	postive
ACOT6	AC020900.1	0.63777787	6.97E-63	postive
ALOX12	LINC01389	0.54243785	1.46E-42	postive
ELOVL2	AC010531.6	0.78366365	4.00E-113	postive
ACSBG2	AC010531.6	0.63976985	2.19E-63	postive
CYP4B1	AC010531.6	0.84570638	1.40E-148	postive
FASN	AC010531.6	0.79820097	2.69E-120	postive
ACOT6	AL356805.1	0.74839721	8.00E-98	postive
BMPR1B	AL356805.1	0.55526317	6.39E-45	postive
ALOX12	AL512791.1	0.64418065	1.64E-64	postive
ELOVL2	HDHD5-AS1	0.56011077	7.70E-46	postive
ACSBG2	HDHD5-AS1	0.56079054	5.71E-46	postive
CYP4B1	HDHD5-AS1	0.61925524	2.21E-58	postive
FASN	HDHD5-AS1	0.53819261	8.39E-42	postive
ELOVL2	ZFHX2-AS1	0.69069592	1.26E-77	postive
ALOX12	ZFHX2-AS1	0.56229908	2.93E-46	postive
ACSBG2	ZFHX2-AS1	0.71427304	2.85E-85	postive
CYP4B1	ZFHX2-AS1	0.74518756	1.47E-96	postive
FASN	ZFHX2-AS1	0.67198384	4.61E-72	postive

ALOX12	PITRM1-AS1	0.56763617	2.69E-47	postive
CPT1B	HEXA-AS1	0.51277828	1.77E-37	postive
ALOX12	AC087222.1	0.64229996	4.97E-64	postive
ACOT6	AL031667.3	0.57880545	1.58E-49	postive
ACSBG1	AC036108.2	0.72117509	1.17E-87	postive
ENO3	AC036108.2	0.68702804	1.68E-76	postive
PCBD1	AC036108.2	0.63219398	1.71E-61	postive
ALOX12	AP001610.2	0.50412333	4.37E-36	postive
ACSBG1	GAR1-DT	0.78707935	9.28E-115	postive
ENO3	GAR1-DT	0.71123071	3.06E-84	postive
PCBD1	GAR1-DT	0.61901027	2.53E-58	postive
ACACB	AC068580.3	0.5401499	3.76E-42	postive
ALOX12	AC036108.3	0.50031756	1.74E-35	postive
ELOVL2	AC005089.1	0.5705645	7.13E-48	postive
ALOX12	AC005089.1	0.55110687	3.81E-44	postive
ACSBG2	AC005089.1	0.72270604	3.38E-88	postive
CYP4B1	AC005089.1	0.61874173	2.92E-58	postive
FASN	AC005089.1	0.55083357	4.29E-44	postive
ALOX12	AC009113.1	0.53204447	1.01E-40	postive
CPT1B	AC093752.2	0.56482427	9.52E-47	postive
D2HGDH	AC093752.2	0.59502803	6.28E-53	postive

ALOX12	AC093752.2	0.61813555	4.05E-58	postive
ALOX12	AC023510.2	0.5745756	1.13E-48	postive
ALOX12	RUNX3-AS1	0.51325646	1.48E-37	postive
ACOT6	AC139887.4	0.67354727	1.64E-72	postive
ACOT6	AC007494.2	0.73124412	2.84E-91	postive
ELOVL2	HNF4A-AS1	0.67924853	3.58E-74	postive
ACSBG2	HNF4A-AS1	0.60365233	8.13E-55	postive
CYP4B1	HNF4A-AS1	0.75044171	1.22E-98	postive
FASN	HNF4A-AS1	0.69571927	3.42E-79	postive
ACOT6	C1orf195	0.8932016	1.52E-188	postive
BMPR1B	C1orf195	0.67550575	4.45E-73	postive
PRKAG2	AC023158.1	0.6181203	4.08E-58	postive
SDHD	AC023158.1	0.62570828	6.47E-60	postive
PHYH	AC023158.1	0.50723003	1.40E-36	postive
HACD3	AC023158.1	0.60530492	3.48E-55	postive
AUH	AC023158.1	0.55859172	1.50E-45	postive
ACOT6	AC023158.1	0.63309333	1.02E-61	postive
BMPR1B	AC023158.1	0.79978909	4.07E-121	postive
ELOVL4	AC023158.1	0.59369757	1.21E-52	postive
RDH11	AC023158.1	0.6040167	6.75E-55	postive
GABARAPL1	AC023158.1	0.64348454	2.47E-64	postive
ACACB	AC023158.1	0.51337951	1.41E-37	postive

HSD17B3	LINC00653	0.58291318	2.26E-50	postive
CPT1B	LINC00653	0.58365831	1.59E-50	postive
D2HGDH	LINC00653	0.5822662	3.08E-50	postive
ALOX12	LINC00653	0.54590006	3.45E-43	postive
ELOVL2	AC079907.1	0.72592443	2.42E-89	postive
ACSBG2	AC079907.1	0.67747219	1.19E-73	postive
CYP4B1	AC079907.1	0.79071693	1.56E-116	postive
FASN	AC079907.1	0.70972745	9.75E-84	postive
CPT1B	AC007566.1	0.5096234	5.76E-37	postive
D2HGDH	AC007566.1	0.50530885	2.83E-36	postive
ALOX12	AC007566.1	0.73091666	3.75E-91	postive
PON1	LINC02158	0.65361746	5.51E-67	postive
ADH1A	LINC02158	0.6514066	2.13E-66	postive
ADH4	LINC02158	0.65464315	2.93E-67	postive
FABP1	LINC02158	0.60631954	2.06E-55	postive
ADH1C	LINC02158	0.65198899	1.49E-66	postive
CYP1A2	LINC02158	0.53791486	9.40E-42	postive
RDH16	LINC02158	0.64948319	6.84E-66	postive
ADH1B	LINC02158	0.5657993	6.15E-47	postive
CPT1B	AC139100.2	0.56425668	1.23E-46	postive
ALOX12	AC138207.1	0.56567664	6.50E-47	postive
ACACB	LINC00482	0.50833387	9.30E-37	postive

ELOVL2	LINC01703	0.53137379	1.32E-40	postive
FASN	LINC01703	0.55868904	1.44E-45	postive
ELOVL2	AC090061.1	0.77713269	4.43E-110	postive
ACSBG2	AC090061.1	0.65531851	1.93E-67	postive
CYP4B1	AC090061.1	0.82815521	4.01E-137	postive
FASN	AC090061.1	0.78785645	3.90E-115	postive
ALOX12	AC138207.4	0.65265833	9.92E-67	postive
ACOT6	AC022613.3	0.73735601	1.51E-93	postive
ACOT6	AL137782.1	0.62976822	6.72E-61	postive
BMPR1B	AL137782.1	0.57858562	1.75E-49	postive
PRKAG2	GATA2-AS1	0.51507032	7.48E-38	postive
HACD3	GATA2-AS1	0.56028667	7.13E-46	postive
AUH	GATA2-AS1	0.51773329	2.72E-38	postive
HACL1	GATA2-AS1	0.53996769	4.05E-42	postive
BMPR1B	GATA2-AS1	0.66741865	9.12E-71	postive
ELOVL4	GATA2-AS1	0.59433165	8.86E-53	postive
RDH11	GATA2-AS1	0.69353419	1.66E-78	postive
GABARAPL1	GATA2-AS1	0.63264762	1.32E-61	postive
ACACB	GATA2-AS1	0.60850621	6.64E-56	postive
ALOX12	AC012020.1	0.52443523	2.04E-39	postive

CPT1B	ITGB2-AS1	0.51613121	5.00E-38	postive
DPEP2	ITGB2-AS1	0.52863609	3.92E-40	postive
ELOVL2	AL035461.2	0.64082099	1.19E-63	postive
ACSBG2	AL035461.2	0.56298244	2.16E-46	postive
CYP4B1	AL035461.2	0.66693641	1.25E-70	postive
FASN	AL035461.2	0.66445425	6.15E-70	postive
ALOX12	AP000873.1	0.67288861	2.54E-72	postive
ALOX12	ARMC2-AS1	0.51646658	4.40E-38	postive
ACOT6	AC093788.1	0.68690194	1.83E-76	postive
ALOX12	AC074194.1	0.51175038	2.61E-37	postive
ACSBG2	AC074194.1	0.58819995	1.79E-51	postive
ELOVL2	AC000120.1	0.72398495	1.19E-88	postive
ACSBG2	AC000120.1	0.71255376	1.09E-84	postive
CYP4B1	AC000120.1	0.78267354	1.18E-112	postive
FASN	AC000120.1	0.70990326	8.52E-84	postive
CYP1A2	AC021678.2	0.54118948	2.45E-42	postive
HSD17B3	AC024337.2	0.52608807	1.07E-39	postive
D2HGDH	AC024337.2	0.51098178	3.48E-37	postive
ACOT6	AC012467.1	0.67950544	3.00E-74	postive
ALOX12	AL358115.1	0.55038578	5.19E-44	postive
ACSBG2	L3MBTL2-AS1	0.5200114	1.13E-38	postive

CYP4B1	L3MBTL2-AS1	0.54226953	1.57E-42	postive
ACOT6	AC058791.1	0.89615488	1.24E-191	postive
BMPR1B	AC058791.1	0.66342189	1.19E-69	postive
CPT1B	SNHG20	0.54749281	1.77E-43	postive
D2HGDH	SNHG20	0.6217948	5.57E-59	postive
ALOX12	SNHG20	0.70720998	6.70E-83	postive
ACOT6	AC006480.2	0.72531384	4.00E-89	postive
ALOX12	AC021078.1	0.57053045	7.24E-48	postive
GABARAPL1	AL161630.1	0.55834384	1.67E-45	postive
ALOX12	LINC02175	0.60286229	1.22E-54	postive
D2HGDH	AL353622.2	0.58113208	5.27E-50	postive
HSD17B3	C2orf49-DT	0.5098382	5.32E-37	postive
CPT1B	C2orf49-DT	0.64813862	1.54E-65	postive
D2HGDH	C2orf49-DT	0.63400215	6.11E-62	postive
ALOX12	C2orf49-DT	0.61304714	6.13E-57	postive
ALOX12	AC010680.4	0.55108378	3.85E-44	postive
ELOVL2	RABGAP1L-DT	0.8043224	1.70E-123	postive
ACSBG2	RABGAP1L-DT	0.67964339	2.74E-74	postive
CYP4B1	RABGAP1L-DT	0.84570895	1.39E-148	postive
FASN	RABGAP1L-DT	0.79893516	1.13E-120	postive
ELOVL2	ZNF687-AS1	0.76906374	1.85E-106	postive
ACSBG2	ZNF687-AS1	0.59280338	1.89E-52	postive

CYP4B1	ZNF687-AS1	0.82948116	6.07E-138	postive
FASN	ZNF687-AS1	0.80020724	2.47E-121	postive
CYP4B1	F11-AS1	0.54582315	3.57E-43	postive
FASN	F11-AS1	0.55039746	5.16E-44	postive
ALOX12	AC011726.2	0.58185692	3.74E-50	postive
ACOT6	EIF1B-AS1	0.69597827	2.83E-79	postive
BMPR1B	EIF1B-AS1	0.5063731	1.92E-36	postive
ELOVL2	PTPRG-AS1	0.52607975	1.07E-39	postive
ACSBG2	PTPRG-AS1	0.53747571	1.12E-41	postive
CYP4B1	PTPRG-AS1	0.52721434	6.87E-40	postive
FASN	PTPRG-AS1	0.51100843	3.44E-37	postive
ELOVL2	AC120498.3	0.51266095	1.85E-37	postive
ACSBG2	AC120498.3	0.50240763	8.16E-36	postive
CYP4B1	AC120498.3	0.57007629	8.90E-48	postive
FASN	AC120498.3	0.51346281	1.37E-37	postive
HSD17B3	MYG1-AS1	0.62072872	9.95E-59	postive
ALOX12	ZBTB40-IT1	0.60142007	2.54E-54	postive
DPEP2	AC007336.2	0.54685817	2.31E-43	postive
ALOX12	AC139887.1	0.56632362	4.86E-47	postive
ACAA2	AC009053.3	0.52425294	2.19E-39	postive
ACOT6	MAMDC2-AS1	0.52384754	2.57E-39	postive

ELOVL2	AC105020.2	0.82873611	1.76E-137	postive
ACSBG2	AC105020.2	0.65552658	1.70E-67	postive
CYP4B1	AC105020.2	0.89521021	1.23E-190	postive
FASN	AC105020.2	0.82441899	7.49E-135	postive
ACOT6	LINC00571	0.65054326	3.60E-66	postive
BMPR1B	LINC00571	0.82294434	5.70E-134	postive
CBR4	LINC00571	0.5215286	6.32E-39	postive
RDH11	LINC00571	0.54953247	7.45E-44	postive
ACACB	LINC00571	0.53043825	1.92E-40	postive
CPT1B	AC087623.1	0.54886695	9.89E-44	postive
ALOX12	LINC00939	0.64142317	8.33E-64	postive
ELOVL2	AC092809.2	0.81292626	3.44E-128	postive
ACSBG2	AC092809.2	0.6683153	5.10E-71	postive
CYP4B1	AC092809.2	0.87822163	3.71E-174	postive
FASN	AC092809.2	0.81299619	3.15E-128	postive
CPT1B	AP000692.1	0.50920881	6.72E-37	postive
ALOX12	AP000692.1	0.61435255	3.07E-57	postive
ACOT6	MIR100HG	0.5851828	7.65E-51	postive
BMPR1B	MIR100HG	0.59700426	2.35E-53	postive
ACOT6	MAP3K5-AS1	0.63467101	4.17E-62	postive
ELOVL2	AC020917.2	0.8099354	1.57E-126	postive
ACSBG2	AC020917.2	0.72184377	6.81E-88	postive

CYP4B1	AC020917.2	0.86405095	3.38E-162	postive
FASN	AC020917.2	0.79246208	2.13E-117	postive
ALOX12	AL358334.3	0.51666143	4.09E-38	postive
BMPR1B	LINC00588	0.51269809	1.83E-37	postive
ELOVL4	LINC00588	0.53407069	4.47E-41	postive
RDH11	LINC00588	0.68082193	1.23E-74	postive
GABARAPL1	LINC00588	0.5002147	1.80E-35	postive
PRKAA2	AC003984.1	0.51290549	1.69E-37	postive
PRKAG2	ITGA9-AS1	0.51860721	1.95E-38	postive
ACOT6	ITGA9-AS1	0.62805651	1.75E-60	postive
BMPR1B	ITGA9-AS1	0.60248111	1.48E-54	postive
ACACB	ITGA9-AS1	0.59051704	5.79E-52	postive
CPT1B	AL031709.1	0.55073576	4.47E-44	postive
D2HGDH	AL031709.1	0.54059606	3.13E-42	postive
ALOX12	AL031709.1	0.74215006	2.23E-95	postive
ELOVL2	SPIN4-AS1	0.73706011	1.95E-93	postive
ACSBG2	SPIN4-AS1	0.70323673	1.34E-81	postive
CYP4B1	SPIN4-AS1	0.79707084	1.02E-119	postive
FASN	SPIN4-AS1	0.71706439	3.15E-86	postive
ELOVL2	AL359643.2	0.78510981	8.19E-114	postive

ACSBG2	AL359643.2	0.62947073	7.94E-61	postive
CYP4B1	AL359643.2	0.84050531	4.84E-145	postive
FASN	AL359643.2	0.76172024	2.73E-103	postive
ALOX12	AC104109.4	0.55130462	3.51E-44	postive
BMPR1B	AP000282.1	0.70328284	1.30E-81	postive
RDH11	AP000282.1	0.5464886	2.70E-43	postive
GABARAPL1	AP000282.1	0.55673183	3.38E-45	postive
CPT1B	Z97832.2	0.53908304	5.83E-42	postive
D2HGDH	Z97832.2	0.52816926	4.72E-40	postive
ALOX12	Z97832.2	0.75628096	5.14E-101	postive
ACACB	AC006042.1	0.56281738	2.33E-46	postive
D2HGDH	LINC01011	0.50139286	1.18E-35	postive
ALOX12	LINC01011	0.56247626	2.71E-46	postive
AUH	ZNF710-AS1	0.55546698	5.85E-45	postive
GABARAPL1	ZNF710-AS1	0.57868923	1.66E-49	postive
ACACB	ZNF710-AS1	0.53883587	6.45E-42	postive
ELOVL2	LINC02487	0.62896083	1.06E-60	postive
CYP4B1	LINC02487	0.67086695	9.62E-72	postive
FASN	LINC02487	0.55550415	5.76E-45	postive
D2HGDH	AC060780.1	0.51250374	1.97E-37	postive
ALOX12	AC060780.1	0.68355038	1.88E-75	postive

ELOVL2	AC116348.1	0.53636683	1.77E-41	postive
ACSBG2	AC116348.1	0.55391385	1.14E-44	postive
CYP4B1	AC116348.1	0.59448135	8.23E-53	postive
FASN	AC116348.1	0.56027093	7.18E-46	postive
ELOVL2	F10-AS1	0.77624417	1.13E-109	postive
ACSBG2	F10-AS1	0.62132374	7.20E-59	postive
CYP4B1	F10-AS1	0.84391846	2.38E-147	postive
FASN	F10-AS1	0.77012689	6.29E-107	postive
D2HGDH	ERVK9-11	0.55103205	3.94E-44	postive
FAAH	ERVK9-11	0.539079	5.84E-42	postive
ACOT6	AC080013.2	0.600948	3.22E-54	postive
ELOVL2	AP001372.1	0.74458604	2.53E-96	postive
ACSBG2	AP001372.1	0.66082767	6.18E-69	postive
CYP4B1	AP001372.1	0.80316559	6.97E-123	postive
FASN	AP001372.1	0.69717267	1.19E-79	postive
HSD17B3	SNHG17	0.51912938	1.59E-38	postive
KMT5A	SNHG17	0.52718104	6.96E-40	postive
RDH11	AC103760.1	0.51574945	5.78E-38	postive
ACOT6	AC011389.1	0.65513471	2.16E-67	postive
DLST	AC004816.1	0.54430127	6.74E-43	postive
ALOX12	AL049780.2	0.54837288	1.22E-43	postive

ACSBG2	AL049780.2	0.59132417	3.90E-52	postive
CYP4B1	AL049780.2	0.52341744	3.04E-39	postive
ACSBG2	AC063965.2	0.58645903	4.14E-51	postive
CYP4B1	AC063965.2	0.50644165	1.87E-36	postive
ALOX12	AC073367.1	0.51658303	4.21E-38	postive
AUH	AC026992.2	0.52072052	8.64E-39	postive
ACACB	AC026992.2	0.51408768	1.08E-37	postive
PRKAA2	FBXO30-DT	0.50720802	1.41E-36	postive
ELOVL2	U52111.1	0.50337023	5.75E-36	postive
ACSBG2	U52111.1	0.52173868	5.83E-39	postive
FASN	U52111.1	0.54500165	5.03E-43	postive
CPT1B	OGFR-AS1	0.60056711	3.91E-54	postive
D2HGDH	OGFR-AS1	0.56034894	6.94E-46	postive
AUH	AC079848.1	0.53449938	3.76E-41	postive
ELOVL2	LINC01934	0.55020797	5.59E-44	postive
ACSBG2	LINC01934	0.58414526	1.26E-50	postive
CYP4B1	LINC01934	0.58224058	3.12E-50	postive
FASN	LINC01934	0.56072403	5.88E-46	postive
HSD17B3	ATP2C2-AS1	0.53743295	1.14E-41	postive
CPT1B	ATP2C2-AS1	0.53001753	2.27E-40	postive

CPT1B	AL133367.1	0.53246823	8.52E-41	postive
D2HGDH	AL133367.1	0.51119802	3.21E-37	postive
ALOX12	AL133367.1	0.58258915	2.64E-50	postive
CPT1B	AC084018.1	0.65951849	1.41E-68	postive
D2HGDH	AC084018.1	0.5698997	9.65E-48	postive
ALOX12	AC084018.1	0.60912485	4.81E-56	postive
ELOVL2	FALEC	0.76512949	9.54E-105	postive
ACSBG2	FALEC	0.69453688	8.05E-79	postive
CYP4B1	FALEC	0.8091792	4.07E-126	postive
FASN	FALEC	0.77159369	1.41E-107	postive
ELOVL2	AL135790.1	0.81842043	2.58E-131	postive
ACSBG2	AL135790.1	0.64156657	7.66E-64	postive
CYP4B1	AL135790.1	0.88368349	3.59E-179	postive
FASN	AL135790.1	0.81090069	4.61E-127	postive
ALOX12	AL031658.1	0.53380031	4.99E-41	postive
ALOX12	AC026979.1	0.59593284	4.00E-53	postive
ACSBG2	AC026979.1	0.62567726	6.58E-60	postive
CYP4B1	AC026979.1	0.52306818	3.48E-39	postive
ELOVL2	ARHGAP26-AS1	0.54365758	8.81E-43	postive
ACSBG2	ARHGAP26-AS1	0.66681063	1.35E-70	postive
CYP4B1	ARHGAP26-AS1	0.58390368	1.41E-50	postive

FASN	ARHGAP26-AS1	0.51061408	3.99E-37	postive
ALOX12	AGAP11	0.63332168	9.00E-62	postive
ACOT6	AC244453.3	0.66680034	1.36E-70	postive
BMPR1B	AC244453.3	0.6505075	3.68E-66	postive
ALOX12	AP001469.3	0.59656652	2.92E-53	postive
ALOX12	AC010999.1	0.54297598	1.17E-42	postive
ACOT8	AL035420.3	0.51604563	5.17E-38	postive
ALOX12	AC011815.2	0.62576989	6.25E-60	postive
ACSBG2	AC011815.2	0.61449027	2.85E-57	postive
ELOVL2	LINC01376	0.64617481	5.00E-65	postive
ALOX12	LINC01376	0.51590064	5.46E-38	postive
ACSBG2	LINC01376	0.6699619	1.74E-71	postive
CYP4B1	LINC01376	0.7096159	1.06E-83	postive
FASN	LINC01376	0.61688387	7.94E-58	postive
AUH	AL162171.1	0.53797821	9.16E-42	postive
ACBD5	AC005261.3	-0.5003008	1.75E-35	negative
SERINC1	AC005261.3	-0.5158285	5.61E-38	negative
ACOT6	AC026202.2	0.63800181	6.12E-63	postive
HSD17B3	LINC02019	0.51069444	3.87E-37	postive
CPT1B	LINC02019	0.6067041	1.69E-55	postive
SDHD	LINC02608	0.54854329	1.13E-43	postive
PHYH	LINC02608	0.57243611	3.03E-48	postive

HACD3	LINC02608	0.56890134	1.52E-47	postive
AUH	LINC02608	0.53339047	5.88E-41	postive
BMPR1B	LINC02608	0.61005116	2.97E-56	postive
ADH1C	LINC02608	0.50184662	9.99E-36	postive
ELOVL4	LINC02608	0.51125008	3.14E-37	postive
RDH11	LINC02608	0.54051472	3.24E-42	postive
GABARAPL1	LINC02608	0.62750643	2.38E-60	postive
CPT1B	AL035252.3	0.50236871	8.27E-36	postive
ALOX12	KLF3-AS1	0.62036393	1.21E-58	postive
ALOX12	AC002553.2	0.54812284	1.35E-43	postive
HSD17B3	AC020907.4	0.50215966	8.92E-36	postive
CPT1B	AC020907.4	0.64469737	1.21E-64	postive
D2HGDH	AC020907.4	0.60854976	6.49E-56	postive
ALOX12	AC020907.4	0.57733276	3.14E-49	postive
ACOT6	AC068790.4	0.51799035	2.46E-38	postive
AUH	AC005696.1	0.50854858	8.59E-37	postive
ACACB	AC005696.1	0.55204941	2.55E-44	postive
ELOVL2	HCG25	0.67652164	2.25E-73	postive
ALOX12	HCG25	0.58394163	1.39E-50	postive
ACSBG2	HCG25	0.74151066	3.94E-95	postive

CYP4B1	HCG25	0.71478187	1.91E-85	postive
FASN	HCG25	0.68471121	8.43E-76	postive
PDHB	AC097359.2	0.55362617	1.30E-44	postive
GCDH	AC097359.2	0.51807188	2.39E-38	postive
SUCLG1	AC097359.2	0.54907362	9.06E-44	postive
HACL1	AC097359.2	0.55274498	1.89E-44	postive
ENO3	AC097359.2	0.50893506	7.44E-37	postive
PCBD1	AC097359.2	0.50754183	1.25E-36	postive
RDH11	AC097359.2	0.51661832	4.16E-38	postive
GABARAPL1	AC097359.2	0.51257392	1.92E-37	postive
CPT1B	INE1	0.54280701	1.25E-42	postive
ALOX12	INE1	0.58571396	5.93E-51	postive
ALOX12	AC006441.1	0.53445577	3.83E-41	postive
ELOVL2	AL596325.2	0.57442789	1.21E-48	postive
ACSBG2	AL596325.2	0.55533599	6.19E-45	postive
CYP4B1	AL596325.2	0.61211809	1.00E-56	postive
ACOT6	AL596325.2	0.64386431	1.98E-64	postive
FASN	AL596325.2	0.54507276	4.88E-43	postive
ELOVL2	AC008083.3	0.58314689	2.02E-50	postive
ACSBG2	AC008083.3	0.55579191	5.08E-45	postive

CYP4B1	AC008083.3	0.60709583	1.38E-55	postive
FASN	AC008083.3	0.5938536	1.12E-52	postive
ELOVL2	AC007422.1	0.83804239	2.07E-143	postive
ACSBG2	AC007422.1	0.64581493	6.20E-65	postive
CYP4B1	AC007422.1	0.89587398	2.46E-191	postive
FASN	AC007422.1	0.83570027	6.98E-142	postive
ALOX12	AC105206.2	0.52071681	8.65E-39	postive
PRKAG2	LINC00886	0.55846501	1.59E-45	postive
AUH	LINC00886	0.59737799	1.95E-53	postive
GABARAPL1	LINC00886	0.69546913	4.10E-79	postive
ACACB	LINC00886	0.62259398	3.60E-59	postive
HSD17B3	AC016737.1	0.67209018	4.30E-72	postive
CPT1B	AC016737.1	0.54789436	1.49E-43	postive
D2HGDH	AC016737.1	0.57150241	4.64E-48	postive
ALOX12	AC016737.1	0.5278967	5.25E-40	postive
ELOVL2	AC108471.2	0.71200144	1.68E-84	postive
ACSBG2	AC108471.2	0.7073201	6.16E-83	postive
CYP4B1	AC108471.2	0.75625806	5.25E-101	postive
FASN	AC108471.2	0.69670726	1.67E-79	postive
HSD17B3	AC009283.1	0.57894384	1.48E-49	postive
CPT1B	AC009283.1	0.59149837	3.58E-52	postive
D2HGDH	AC009283.1	0.618453	3.41E-58	postive

ACADVL	AC009283.1	0.50587758	2.30E-36	postive
ALOX12	AC009283.1	0.55831267	1.70E-45	postive
ACOT6	AC005392.2	0.73544715	7.87E-93	postive
BMPR1B	AC005392.2	0.56850078	1.82E-47	postive
ACOT6	AC103810.2	0.51862864	1.93E-38	postive
HSD17B3	U62317.1	0.52100217	7.75E-39	postive
CPT1B	U62317.1	0.64396602	1.86E-64	postive
D2HGDH	U62317.1	0.57192628	3.82E-48	postive
ELOVL2	AP000350.5	0.74977126	2.27E-98	postive
ACSBG2	AP000350.5	0.61999758	1.48E-58	postive
CYP4B1	AP000350.5	0.80325892	6.22E-123	postive
FASN	AP000350.5	0.7500274	1.79E-98	postive
ELOVL2	AL121890.5	0.54714329	2.05E-43	postive
ACSBG2	AL121890.5	0.58830072	1.70E-51	postive
CYP4B1	AL121890.5	0.56651066	4.47E-47	postive
FASN	AL121890.5	0.51652989	4.30E-38	postive
HSD17B3	AC027796.4	0.56270961	2.44E-46	postive
CPT1B	AC027796.4	0.5705489	7.18E-48	postive
D2HGDH	AC027796.4	0.54530145	4.44E-43	postive
ACADVL	AC027796.4	0.52235636	4.59E-39	postive
KMT5A	AC027796.4	0.50111626	1.30E-35	postive
CPT1B	AC004812.2	0.5292934	3.02E-40	postive

ACSBG2	AC007622.2	0.52222567	4.83E-39	postive
ACOT6	AC007622.2	0.63866089	4.18E-63	postive
ACOT6	AL157895.1	0.71299348	7.77E-85	postive
BMPR1B	AL157895.1	0.51894579	1.71E-38	postive
ELOVL2	RUNDC3A-AS1	0.51388218	1.17E-37	postive
CYP4B1	RUNDC3A-AS1	0.59436476	8.72E-53	postive
BMPR1B	RUNDC3A-AS1	0.56151678	4.14E-46	postive
FASN	RUNDC3A-AS1	0.53146788	1.27E-40	postive
GABARAPL1	RUNDC3A-AS1	0.54876886	1.03E-43	postive
ACOT6	AC010615.2	0.51861121	1.94E-38	postive
HACD3	AC093904.2	0.52433007	2.13E-39	postive
HACL1	AC093904.2	0.56587943	5.93E-47	postive
ELOVL4	AC093904.2	0.63637705	1.56E-62	postive
RDH11	AC093904.2	0.6860116	3.41E-76	postive
GABARAPL1	AC093904.2	0.51935868	1.46E-38	postive
ACACB	AC093904.2	0.52046249	9.54E-39	postive
HSD17B3	STAG3L5P-PVRIG2P-PILRB	0.59079833	5.04E-52	postive
CPT1B	STAG3L5P-PVRIG2P-PILRB	0.67010152	1.59E-71	postive
D2HGDH	STAG3L5P-PVRIG2P-PILRB	0.72193327	6.34E-88	postive
ACADVL	STAG3L5P-PVRIG2P-PILRB	0.58359383	1.64E-50	postive
ALOX12	STAG3L5P-PVRIG2P-PILRB	0.55216438	2.43E-44	postive

ALOX12	AC004492.1	0.5090044	7.25E-37	postive
ACOT6	AC004492.1	0.50147585	1.14E-35	postive
ELOVL2	AL354989.1	0.54504493	4.94E-43	postive
ALOX12	AL354989.1	0.52578887	1.20E-39	postive
ACSBG2	AL354989.1	0.64803735	1.64E-65	postive
CYP4B1	AL354989.1	0.59416125	9.64E-53	postive
ACOT6	AL354989.1	0.50771803	1.17E-36	postive
FASN	AL354989.1	0.53497209	3.11E-41	postive
ACOT6	AC018529.2	0.66138566	4.34E-69	postive
ELOVL2	AC051619.6	0.68023891	1.82E-74	postive
ACSBG2	AC051619.6	0.62171285	5.82E-59	postive
CYP4B1	AC051619.6	0.76670617	1.98E-105	postive
FASN	AC051619.6	0.64575044	6.44E-65	postive
ELOVL2	AC078777.1	0.83530449	1.26E-141	postive
ACSBG2	AC078777.1	0.66096838	5.65E-69	postive
CYP4B1	AC078777.1	0.89475835	3.68E-190	postive
FASN	AC078777.1	0.83322275	2.71E-140	postive
CPT1B	ERVK13-1	0.53670459	1.54E-41	postive
ALOX12	ERVK13-1	0.69316958	2.15E-78	postive
CPT1B	AL161935.1	0.50088649	1.41E-35	postive
ALOX12	AL161935.1	0.5802583	7.96E-50	postive

ACOT6	ZBTB20-AS5	0.59702658	2.32E-53	postive
ELOVL2	LINC02356	0.81617336	5.03E-130	postive
ACSBG2	LINC02356	0.66370909	9.91E-70	postive
CYP4B1	LINC02356	0.86399945	3.72E-162	postive
FASN	LINC02356	0.80966671	2.20E-126	postive
ALOX12	AP000919.3	0.57258523	2.83E-48	postive
ALOX12	AC105105.1	0.61655049	9.49E-58	postive
ACOT6	AC012557.1	0.59165909	3.31E-52	postive
ALOX12	AL139011.1	0.6628435	1.72E-69	postive
ELOVL2	AP001010.1	0.51831156	2.18E-38	postive
HSD17B3	AP001010.1	0.50062938	1.55E-35	postive
ACSBG2	AP001010.1	0.54315458	1.09E-42	postive
CYP4B1	AP001010.1	0.5200407	1.12E-38	postive
ACAA2	LINC01507	0.62976219	6.74E-61	postive
EHHADH	LINC01507	0.59665536	2.79E-53	postive
PRKAA2	LINC01507	0.5874451	2.57E-51	postive
ADH5	LINC01507	0.58076029	6.28E-50	postive
ACADM	LINC01507	0.54142402	2.22E-42	postive
ETFDH	LINC01507	0.50976082	5.48E-37	postive
ACADL	LINC01507	0.55708786	2.89E-45	postive
ALDH3A2	LINC01507	0.60076551	3.53E-54	postive
ALDH9A1	LINC01507	0.52482248	1.76E-39	postive

HIBCH	LINC01507	0.61500066	2.17E-57	postive
SLC27A2	LINC01507	0.61602602	1.26E-57	postive
CPT2	LINC01507	0.50737572	1.32E-36	postive
ELOVL2	AL355432.1	0.67847753	6.03E-74	postive
ALOX12	AL355432.1	0.52485839	1.73E-39	postive
ACSBG2	AL355432.1	0.71197999	1.71E-84	postive
CYP4B1	AL355432.1	0.72978365	9.73E-91	postive
FASN	AL355432.1	0.65148615	2.03E-66	postive
PRKAA2	AC005034.5	0.63285527	1.17E-61	postive
MMAA	AC005034.5	0.50693445	1.56E-36	postive
HSD17B3	AC132872.2	0.63161846	2.37E-61	postive
CPT1B	AC132872.2	0.55005342	5.97E-44	postive
D2HGDH	AC132872.2	0.58311611	2.05E-50	postive
ALOX12	AC132872.2	0.52495159	1.67E-39	postive
ACOT6	AC073651.1	0.89646225	5.84E-192	postive
BMPR1B	AC073651.1	0.66253421	2.09E-69	postive
CPT1B	AC024361.3	0.58109813	5.35E-50	postive
D2HGDH	AC024361.3	0.57592381	6.05E-49	postive
ALOX12	AC024361.3	0.69877918	3.66E-80	postive
ELOVL2	AC084757.3	0.65447168	3.26E-67	postive

CYP4B1	AC084757.3	0.64547175	7.60E-65	postive
FASN	AC084757.3	0.67941566	3.19E-74	postive
ACOT6	AC120349.1	0.64065326	1.31E-63	postive
ACOT8	LEF1-AS1	0.65353382	5.80E-67	postive
SDHD	LEF1-AS1	0.72643272	1.59E-89	postive
PHYH	LEF1-AS1	0.70327432	1.31E-81	postive
NDUFAB1	LEF1-AS1	0.63422545	5.38E-62	postive
HACD3	LEF1-AS1	0.77586815	1.67E-109	postive
ELOVL4	LEF1-AS1	0.79312627	9.96E-118	postive
ELOVL2	FAM222A-AS1	0.70190539	3.63E-81	postive
ACSBG2	FAM222A-AS1	0.58933038	1.03E-51	postive
CYP4B1	FAM222A-AS1	0.75860384	5.58E-102	postive
FASN	FAM222A-AS1	0.71895224	7.00E-87	postive
PRKAG2	AC138305.1	0.55479549	7.82E-45	postive
HACD3	AC138305.1	0.52814515	4.76E-40	postive
AUH	AC138305.1	0.53080521	1.66E-40	postive
HACL1	AC138305.1	0.57488277	9.80E-49	postive
ACOT6	AC138305.1	0.53750242	1.11E-41	postive
BMPR1B	AC138305.1	0.89284204	3.57E-188	postive
RDH11	AC138305.1	0.68879515	4.84E-77	postive

GABARAPL1	AC138305.1	0.61703538	7.32E-58	postive
ACACB	AC138305.1	0.6005042	4.03E-54	postive
ELOVL2	AC004000.1	0.69879188	3.63E-80	postive
ACSBG2	AC004000.1	0.68168125	6.81E-75	postive
CYP4B1	AC004000.1	0.72579021	2.70E-89	postive
FASN	AC004000.1	0.66428491	6.86E-70	postive
HSD17B3	C3orf35	0.56729485	3.14E-47	postive
ALOX12	C3orf35	0.54329953	1.02E-42	postive
HSD17B3	AC005785.1	0.56206974	3.24E-46	postive
CPT1B	AC005785.1	0.58146218	4.51E-50	postive
D2HGDH	AC005785.1	0.54514442	4.74E-43	postive
SERINC1	AC005785.1	-0.504306	4.09E-36	negative
CPT1B	MMP25-AS1	0.66061726	7.05E-69	postive
D2HGDH	MMP25-AS1	0.62877959	1.17E-60	postive
ALOX12	MMP25-AS1	0.59610506	3.67E-53	postive
ACOT6	LINC00513	0.83642048	2.38E-142	postive
BMPR1B	LINC00513	0.59799119	1.43E-53	postive
ELOVL2	AC099521.1	0.81892469	1.31E-131	postive
ACSBG2	AC099521.1	0.66696476	1.22E-70	postive
CYP4B1	AC099521.1	0.87124481	4.32E-168	postive
FASN	AC099521.1	0.80482797	9.14E-124	postive

CPT1B	SEMA3F-AS1	0.55122799	3.62E-44	postive
D2HGDH	SEMA3F-AS1	0.51318662	1.52E-37	postive
ALOX12	SEMA3F-AS1	0.63653771	1.43E-62	postive
ALOX12	AL596202.1	0.59413161	9.79E-53	postive
CPT1B	AC092171.4	0.62012379	1.38E-58	postive
D2HGDH	AC092171.4	0.57565811	6.84E-49	postive
SDHD	AC008669.1	0.53112066	1.46E-40	postive
PRKAA2	AC008669.1	0.57108577	5.62E-48	postive
CBR4	AC008669.1	0.61943424	2.01E-58	postive
ELOVL4	AC117386.2	0.55362539	1.30E-44	postive
RDH11	AC117386.2	0.50666363	1.72E-36	postive
ALOX12	AC015849.3	0.70414889	6.78E-82	postive
ACSBG2	AC015849.3	0.52533947	1.44E-39	postive
CPT1B	AC145285.6	0.62647325	4.23E-60	postive
D2HGDH	AC145285.6	0.58531442	7.18E-51	postive
ALOX12	AC145285.6	0.51834124	2.15E-38	postive
ELOVL2	AL008726.1	0.62445158	1.30E-59	postive
ACSBG2	AL008726.1	0.59636959	3.22E-53	postive
CYP4B1	AL008726.1	0.65921238	1.71E-68	postive
FASN	AL008726.1	0.65227833	1.25E-66	postive

ACADL	ADORA2A-AS1	0.52821822	4.63E-40	postive
ALDH3A2	ADORA2A-AS1	0.54357127	9.13E-43	postive
ELOVL2	VLDLR-AS1	0.51972709	1.27E-38	postive
CYP4B1	VLDLR-AS1	0.57848264	1.83E-49	postive
BMPR1B	VLDLR-AS1	0.62029251	1.26E-58	postive
FASN	VLDLR-AS1	0.5519914	2.61E-44	postive
RDH11	VLDLR-AS1	0.53169529	1.16E-40	postive
ELOVL2	LINC00449	0.81106033	3.76E-127	postive
ACSBG2	LINC00449	0.64081867	1.19E-63	postive
CYP4B1	LINC00449	0.87297092	1.47E-169	postive
FASN	LINC00449	0.81729618	1.15E-130	postive
D2HGDH	AC078864.1	0.60238749	1.55E-54	postive
FAAH	AC078864.1	0.55444162	9.11E-45	postive
ALOX12	AC078864.1	0.54002703	3.96E-42	postive
ALOX12	TMSB15B-AS1	0.5052014	2.95E-36	postive
ALOX12	RERE-AS1	0.50566241	2.49E-36	postive
ACOT6	RERE-AS1	0.53016818	2.13E-40	postive
RDH11	AC021087.4	0.5241509	2.28E-39	postive
GABARAPL1	AC021087.4	0.63821698	5.40E-63	postive
ACACB	AC021087.4	0.51232499	2.10E-37	postive
ELOVL2	AC011978.1	0.64444885	1.40E-64	postive
ACSBG2	AC011978.1	0.59468014	7.46E-53	postive

CYP4B1	AC011978.1	0.65562345	1.60E-67	postive
FASN	AC011978.1	0.57788266	2.43E-49	postive
HSD17B3	AC245052.4	0.52023722	1.04E-38	postive
CPT1B	AC245052.4	0.6365448	1.42E-62	postive
D2HGDH	AC245052.4	0.6331	1.02E-61	postive
ACADVL	AC245052.4	0.52287245	3.76E-39	postive
ALOX12	AC245052.4	0.68204256	5.31E-75	postive
ELOVL2	AL591848.1	0.82886219	1.47E-137	postive
ACSBG2	AL591848.1	0.63933238	2.83E-63	postive
CYP4B1	AL591848.1	0.89154579	7.51E-187	postive
FASN	AL591848.1	0.82710161	1.77E-136	postive
ACOT6	GPC6-AS2	0.81013173	1.22E-126	postive
BMPR1B	GPC6-AS2	0.57711478	3.47E-49	postive
ALOX12	AC137932.1	0.62268465	3.42E-59	postive
ACOT6	AC025188.1	0.50562961	2.52E-36	postive
ALOX12	AC018410.1	0.50126631	1.23E-35	postive
ELOVL2	AC063943.1	0.59847103	1.12E-53	postive
ACSBG2	AC063943.1	0.6284639	1.40E-60	postive
CYP4B1	AC063943.1	0.69107062	9.66E-78	postive
FASN	AC063943.1	0.58966962	8.75E-52	postive
ACOT6	MYCBP2-AS1	0.59055154	5.69E-52	postive
ACOT6	L3MBTL4-AS1	0.62029062	1.26E-58	postive

ELOVL2	AL133243.1	0.59616512	3.56E-53	postive
ACSBG2	AL133243.1	0.63070091	3.97E-61	postive
CYP4B1	AL133243.1	0.64511138	9.42E-65	postive
ACOT6	AL133243.1	0.50799477	1.05E-36	postive
FASN	AL133243.1	0.56015716	7.55E-46	postive
SDHD	AL161668.4	0.64253753	4.32E-64	postive
PHYH	AL161668.4	0.75750561	1.60E-101	postive
NDUFAB1	AL161668.4	0.50296076	6.67E-36	postive
GCDH	AL161668.4	0.50806658	1.03E-36	postive
HACD3	AL161668.4	0.65806385	3.50E-68	postive
AUH	AL161668.4	0.53298157	6.93E-41	postive
HACL1	AL161668.4	0.50323325	6.04E-36	postive
PON1	AL161668.4	0.6456741	6.74E-65	postive
ADH1A	AL161668.4	0.64667025	3.72E-65	postive
ADH4	AL161668.4	0.65657068	8.88E-68	postive
FABP1	AL161668.4	0.59938911	7.09E-54	postive
ADH1C	AL161668.4	0.67889735	4.54E-74	postive
CYP1A2	AL161668.4	0.52714769	7.06E-40	postive
RDH16	AL161668.4	0.6430319	3.23E-64	postive
ADH1B	AL161668.4	0.57076138	6.51E-48	postive
ELOVL4	AL161668.4	0.59817347	1.31E-53	postive

ACOT6	AC002044.1	0.54896745	9.47E-44	postive
ALOX12	AP001432.1	0.55394176	1.13E-44	postive
HSD17B3	AL645940.1	0.64764878	2.07E-65	postive
CPT1B	AL645940.1	0.50880624	7.81E-37	postive
HSD17B3	AC084876.1	0.63765738	7.47E-63	postive
HSD17B3	AL161669.3	0.53167165	1.17E-40	postive
CPT1B	AL161669.3	0.56004534	7.93E-46	postive
D2HGDH	AL161669.3	0.62129309	7.32E-59	postive
ACADVL	AL161669.3	0.56218278	3.09E-46	postive
ALOX12	AL161669.3	0.50608129	2.13E-36	postive
HSD17B3	SLBP-DT	0.58774063	2.23E-51	postive
CPT1B	SLBP-DT	0.62834322	1.49E-60	postive
D2HGDH	SLBP-DT	0.51575471	5.77E-38	postive
ALOX12	AL133297.1	0.52624785	1.01E-39	postive
ACSBG2	AL133297.1	0.65091163	2.88E-66	postive
CYP4B1	AL133297.1	0.53690717	1.42E-41	postive
ALOX12	SEPTIN7-DT	0.50498028	3.19E-36	postive
PRKAA2	SEPTIN7-DT	0.5123019	2.12E-37	postive
ELOVL2	AC008731.1	0.60784605	9.36E-56	postive
ACSBG2	AC008731.1	0.65233998	1.20E-66	postive
CYP4B1	AC008731.1	0.68031948	1.73E-74	postive

FASN	AC008731.1	0.59908242	8.27E-54	postive
ACOT6	AC025031.3	0.626236	4.83E-60	postive
ACOT6	AC087501.1	0.6046243	4.94E-55	postive
ELOVL2	AL445490.1	0.68908695	3.94E-77	postive
ACSBG2	AL445490.1	0.62379943	1.86E-59	postive
CYP4B1	AL445490.1	0.7278909	4.74E-90	postive
FASN	AL445490.1	0.67415	1.10E-72	postive
ELOVL2	AC137630.1	0.55851846	1.55E-45	postive
ACSBG2	AC137630.1	0.5427827	1.27E-42	postive
CYP4B1	AC137630.1	0.62835215	1.49E-60	postive
FASN	AC137630.1	0.60402644	6.71E-55	postive
ACOT6	AC244093.4	0.56468098	1.01E-46	postive
ALOX12	AC108010.1	0.63791577	6.43E-63	postive
ELOVL2	AC231981.1	0.63132171	2.80E-61	postive
ACSBG2	AC231981.1	0.55251424	2.09E-44	postive
CYP4B1	AC231981.1	0.6839371	1.44E-75	postive
FASN	AC231981.1	0.6290242	1.02E-60	postive
ELOVL2	AL360182.2	0.82348232	2.73E-134	postive
ACSBG2	AL360182.2	0.65417297	3.91E-67	postive
CYP4B1	AL360182.2	0.88913923	1.94E-184	postive
FASN	AL360182.2	0.8139361	9.33E-129	postive
ELOVL2	AC007405.1	0.80169543	4.14E-122	postive
ACSBG2	AC007405.1	0.67385016	1.34E-72	postive
CYP4B1	AC007405.1	0.84651026	3.86E-149	postive

FASN	AC007405.1	0.78727083	7.50E-115	postive
ALOX12	AC087276.1	0.52100994	7.72E-39	postive
ACSBG2	AC087276.1	0.55237248	2.22E-44	postive
ELOVL2	NAV2-AS3	0.76669761	2.00E-105	postive
ACSBG2	NAV2-AS3	0.64599182	5.58E-65	postive
CYP4B1	NAV2-AS3	0.83241396	8.83E-140	postive
FASN	NAV2-AS3	0.75946154	2.44E-102	postive
ELOVL2	UBOX5-AS1	0.7485902	6.70E-98	postive
ACSBG2	UBOX5-AS1	0.75903133	3.70E-102	postive
CYP4B1	UBOX5-AS1	0.80580623	2.74E-124	postive
FASN	UBOX5-AS1	0.73258925	9.09E-92	postive
BMPR1B	AC078788.2	0.74001439	1.48E-94	postive
RDH11	AC078788.2	0.55659198	3.59E-45	postive
HSD17B3	LINC02804	0.54082191	2.85E-42	postive
D2HGDH	LINC02804	0.5244919	2.00E-39	postive
ACADVL	LINC02804	0.51375805	1.23E-37	postive
CPT1B	AC009690.2	0.59784247	1.54E-53	postive
ALOX12	AC009690.2	0.69445317	8.55E-79	postive
ACOT6	KCNQ1OT1	0.59611554	3.65E-53	postive
ELOVL2	AC115102.1	0.72546136	3.54E-89	postive
ACSBG2	AC115102.1	0.68367004	1.73E-75	postive

CYP4B1	AC115102.1	0.78379692	3.46E-113	postive
FASN	AC115102.1	0.70006461	1.42E-80	postive
ELOVL2	AC023389.1	0.70786579	4.06E-83	postive
ACSBG2	AC023389.1	0.69505231	5.55E-79	postive
CYP4B1	AC023389.1	0.77099689	2.59E-107	postive
FASN	AC023389.1	0.69061149	1.34E-77	postive
ALOX12	AC008569.2	0.61540007	1.76E-57	postive
ACSBG2	AC008569.2	0.55046626	5.01E-44	postive
ELOVL2	AC124248.1	0.5960824	3.71E-53	postive
CYP4B1	AC124248.1	0.55678778	3.30E-45	postive
FASN	AC124248.1	0.55814615	1.82E-45	postive
CPT1B	HM13-IT1	0.56273043	2.42E-46	postive
ALOX12	HM13-IT1	0.57681868	3.99E-49	postive
ENO3	LINC02303	0.58837018	1.65E-51	postive
ALOX12	AC020663.3	0.51925634	1.52E-38	postive
ELOVL2	PGM5P4-AS1	0.58584006	5.58E-51	postive
ACSBG2	PGM5P4-AS1	0.52114101	7.34E-39	postive
CYP4B1	PGM5P4-AS1	0.64925187	7.87E-66	postive
FASN	PGM5P4-AS1	0.5944091	8.53E-53	postive
ELOVL2	AC118755.1	0.83790394	2.56E-143	postive
ACSBG2	AC118755.1	0.6633909	1.21E-69	postive
CYP4B1	AC118755.1	0.89617029	1.19E-191	postive

FASN	AC118755.1	0.832391	9.13E-140	postive
HSD17B3	LINC02640	0.50663562	1.74E-36	postive
ELOVL2	AC004951.1	0.63559485	2.45E-62	postive
ALOX12	AC004951.1	0.54194542	1.79E-42	postive
ACSBG2	AC004951.1	0.72325892	2.16E-88	postive
CYP4B1	AC004951.1	0.68625692	2.87E-76	postive
FASN	AC004951.1	0.63008365	5.63E-61	postive
HSD17B3	AL049795.1	0.5613537	4.45E-46	postive
CPT1B	AL049795.1	0.63576181	2.23E-62	postive
D2HGDH	AL049795.1	0.71991314	3.24E-87	postive
ACADVL	AL049795.1	0.56117067	4.83E-46	postive
ALOX12	AL049795.1	0.67309166	2.22E-72	postive
HSD17B3	AL590096.1	0.57232896	3.18E-48	postive
D2HGDH	AL590096.1	0.59692739	2.44E-53	postive
ALOX12	AL590096.1	0.60194578	1.94E-54	postive
NDUFAB1	ENTPD3-AS1	0.54402007	7.58E-43	postive
ELOVL2	AC022211.3	0.60567552	2.88E-55	postive
ALOX12	AC022211.3	0.53469799	3.47E-41	postive
ACSBG2	AC022211.3	0.62496045	9.79E-60	postive
CYP4B1	AC022211.3	0.62553378	7.13E-60	postive
FASN	AC022211.3	0.65615862	1.15E-67	postive

CYP4B1	AL022313.2	0.53882022	6.49E-42	postive
FASN	AL022313.2	0.516969	3.64E-38	postive
ACOT6	FAF1-AS1	0.55522261	6.50E-45	postive
ELOVL2	MDS2	0.75705303	2.46E-101	postive
ACSBG2	MDS2	0.67820621	7.24E-74	postive
CYP4B1	MDS2	0.76989572	7.95E-107	postive
FASN	MDS2	0.70585786	1.87E-82	postive
LGALS1	MAFG-DT	0.52917867	3.16E-40	postive
ELOVL2	AL137779.1	0.70456035	4.98E-82	postive
ACSBG2	AL137779.1	0.70611106	1.54E-82	postive
CYP4B1	AL137779.1	0.75127219	5.66E-99	postive
FASN	AL137779.1	0.67717187	1.46E-73	postive
ALOX12	AC087392.3	0.61682267	8.20E-58	postive
ACSBG2	AC087392.3	0.68820405	7.34E-77	postive
CYP4B1	AC087392.3	0.51698395	3.62E-38	postive
ALOX12	GHRLOS	0.53799012	9.12E-42	postive
ELOVL2	AC011462.3	0.83522464	1.41E-141	postive
ACSBG2	AC011462.3	0.65175454	1.72E-66	postive
CYP4B1	AC011462.3	0.89808503	1.05E-193	postive
FASN	AC011462.3	0.8313859	3.93E-139	postive
ELOVL2	MIR302CHG	0.74274633	1.31E-95	postive

ACSBG2	MIR302CHG	0.69065727	1.30E-77	postive
CYP4B1	MIR302CHG	0.80346146	4.86E-123	postive
FASN	MIR302CHG	0.72653224	1.46E-89	postive
ACOT8	AL355001.2	0.51532587	6.79E-38	postive
NDUFAB1	AL355001.2	0.54753703	1.73E-43	postive
ACSBG2	DHRSX-IT1	0.50298864	6.60E-36	postive
PON1	DCXR-DT	0.99449231	0	postive
ADH1A	DCXR-DT	0.99466974	0	postive
ADH4	DCXR-DT	0.99127476	0	postive
FABP1	DCXR-DT	0.94064681	3.52E-254	postive
ADH1C	DCXR-DT	0.97635748	0	postive
CYP1A2	DCXR-DT	0.73002827	7.92E-91	postive
RDH16	DCXR-DT	0.99293254	0	postive
ADH1B	DCXR-DT	0.88586858	3.00E-181	postive
ACOT6	AC005225.2	0.52391865	2.50E-39	postive
BMPR1B	AC005225.2	0.53212903	9.76E-41	postive
RDH11	AC005225.2	0.52707291	7.27E-40	postive
BMPR1B	LINC00265	0.55817998	1.80E-45	postive
ELOVL4	LINC00265	0.53270968	7.73E-41	postive
RDH11	LINC00265	0.56063301	6.12E-46	postive

GABARAPL1	LINC00265	0.56693121	3.70E-47	postive
ACACB	LINC00265	0.68680493	1.96E-76	postive
ELOVL2	AC114488.1	0.71091816	3.89E-84	postive
ACSBG2	AC114488.1	0.65167229	1.81E-66	postive
CYP4B1	AC114488.1	0.78990497	3.91E-116	postive
FASN	AC114488.1	0.7054887	2.47E-82	postive
HSD17B3	AC022144.1	0.55293734	1.74E-44	postive
D2HGDH	AC022144.1	0.65416882	3.92E-67	postive
ACADVL	AC022144.1	0.57788083	2.43E-49	postive
FAAH	AC022144.1	0.58838685	1.63E-51	postive
ALOX12	AC022144.1	0.54681309	2.35E-43	postive
ADH5	TRAM2-AS1	0.55985216	8.63E-46	postive
SERINC1	TRAM2-AS1	0.61336695	5.17E-57	postive
PRKAA2	AC011912.1	0.53350176	5.62E-41	postive
ACSBG1	AC009041.3	0.62027492	1.27E-58	postive
ENO3	AC009041.3	0.55038108	5.20E-44	postive
ELOVL2	AC135178.4	0.74525801	1.38E-96	postive
ACSBG2	AC135178.4	0.71111868	3.33E-84	postive
CYP4B1	AC135178.4	0.81455835	4.16E-129	postive
FASN	AC135178.4	0.7424694	1.68E-95	postive
ALOX12	ANKRD10-IT1	0.58758457	2.41E-51	postive

ACOT6	ANKRD10-IT1	0.52136279	6.74E-39	postive
ALOX12	AC020612.2	0.571758	4.13E-48	postive
ALOX12	WAKMAR2	0.55039793	5.16E-44	postive
ACSBG2	WAKMAR2	0.51371488	1.25E-37	postive
ACSBG2	AC073534.2	0.5205019	9.40E-39	postive
ALOX12	AC023794.4	0.5383531	7.86E-42	postive
ALOX12	AC015871.3	0.57974459	1.01E-49	postive
CBR4	AC015871.3	0.50525272	2.89E-36	postive
ACACB	AC015871.3	0.53028542	2.04E-40	postive
ALOX12	AC016586.1	0.533789	5.01E-41	postive
ALOX12	AC023632.2	0.52789932	5.25E-40	postive
ACOT6	DIP2A-IT1	0.53349531	5.64E-41	postive
ALOX12	RPL37A-DT	0.50614584	2.08E-36	postive
ACSBG2	RPL37A-DT	0.52243323	4.45E-39	postive
CYP4B1	RPL37A-DT	0.52086632	8.16E-39	postive
ELOVL2	AC112491.1	0.72157774	8.45E-88	postive
ACSBG2	AC112491.1	0.55077012	4.40E-44	postive
CYP4B1	AC112491.1	0.77681765	6.17E-110	postive
FASN	AC112491.1	0.7235836	1.65E-88	postive
ACOT6	AC003681.1	0.67984064	2.39E-74	postive
PRKAA2	RAP2C-AS1	0.58470728	9.61E-51	postive
CBR4	RAP2C-AS1	0.55989242	8.48E-46	postive
ACSBG2	AC034198.2	0.5174577	3.02E-38	postive

CYP4B1	AC034198.2	0.50166034	1.07E-35	postive
CPT1B	AC018638.7	0.52901132	3.38E-40	postive
CPT1B	ARHGEF2-AS2	0.53236694	8.87E-41	postive
ELOVL2	IDH2-DT	0.74731972	2.14E-97	postive
ACSBG2	IDH2-DT	0.58193082	3.61E-50	postive
CYP4B1	IDH2-DT	0.80350615	4.60E-123	postive
FASN	IDH2-DT	0.75480403	2.08E-100	postive
ELOVL2	AC004923.4	0.79981089	3.97E-121	postive
ACSBG2	AC004923.4	0.70066219	9.14E-81	postive
CYP4B1	AC004923.4	0.85897619	3.07E-158	postive
FASN	AC004923.4	0.81181276	1.44E-127	postive
ELOVL2	LIX1L-AS1	0.57949424	1.14E-49	postive
ALOX12	LIX1L-AS1	0.61098249	1.82E-56	postive
ACSBG2	LIX1L-AS1	0.69199946	4.98E-78	postive
CYP4B1	LIX1L-AS1	0.60468591	4.79E-55	postive
FASN	LIX1L-AS1	0.5348419	3.28E-41	postive
FASN	AC078802.1	0.50343198	5.62E-36	postive
ELOVL2	AP001781.1	0.79336359	7.58E-118	postive
ACSBG2	AP001781.1	0.74981688	2.17E-98	postive
CYP4B1	AP001781.1	0.84538454	2.33E-148	postive
FASN	AP001781.1	0.78273698	1.10E-112	postive

ELOVL2	AC027319.1	0.58533892	7.10E-51	postive
CYP4B1	AC027319.1	0.57526167	8.22E-49	postive
FASN	AC027319.1	0.56091979	5.39E-46	postive
HSD17B3	AC005840.2	0.57663782	4.34E-49	postive
CPT1B	AC005840.2	0.53173657	1.14E-40	postive
ACOT6	AL591721.1	0.57561047	7.00E-49	postive
ALOX12	AC138956.2	0.6065692	1.81E-55	postive
ALOX12	LANCL1-AS1	0.51075257	3.79E-37	postive
ALOX12	AP001020.3	0.5000951	1.88E-35	postive
ALOX12	AC079921.2	0.55411347	1.05E-44	postive
SUCLG1	ATP11AUN	0.61826449	3.78E-58	postive
ENO3	ATP11AUN	0.59969399	6.08E-54	postive
PCBD1	ATP11AUN	0.51293874	1.67E-37	postive
ELOVL2	AL133553.1	0.59482301	6.95E-53	postive
ACSBG2	AL133553.1	0.55012874	5.79E-44	postive
CYP4B1	AL133553.1	0.6157506	1.46E-57	postive
FASN	AL133553.1	0.53471205	3.45E-41	postive
ACOT6	AL357078.2	0.60960999	3.73E-56	postive
ACOT6	AC007684.2	0.64148232	8.04E-64	postive
ALOX12	AL512506.1	0.50089534	1.41E-35	postive
ACOT6	AC006064.2	0.65611447	1.18E-67	postive
ELOVL2	AP003555.2	0.79049281	2.01E-116	postive

ACSBG2	AP003555.2	0.58542071	6.83E-51	postive
CYP4B1	AP003555.2	0.83191677	1.82E-139	postive
FASN	AP003555.2	0.77581987	1.76E-109	postive
ELOVL2	AC093249.2	0.53115444	1.44E-40	postive
ACSBG2	AC093249.2	0.60731528	1.23E-55	postive
CYP4B1	AC093249.2	0.60467163	4.82E-55	postive
FASN	AC093249.2	0.55223728	2.35E-44	postive
ACSBG2	AC012358.2	0.55865726	1.46E-45	postive
CPT1B	AC069281.2	0.67640724	2.43E-73	postive
D2HGDH	AC069281.2	0.58917455	1.11E-51	postive
HSD17B3	LINC01089	0.57600896	5.81E-49	postive
CPT1B	LINC01089	0.68820961	7.32E-77	postive
D2HGDH	LINC01089	0.59918663	7.85E-54	postive
ALOX12	LINC01089	0.52678806	8.13E-40	postive
CPT1B	AC129510.1	0.64151645	7.88E-64	postive
D2HGDH	AC129510.1	0.62769677	2.14E-60	postive
ALOX12	AC129510.1	0.68304923	2.66E-75	postive
ELOVL2	AC023983.1	0.81479698	3.05E-129	postive
ACSBG2	AC023983.1	0.67195616	4.70E-72	postive
CYP4B1	AC023983.1	0.88017106	6.42E-176	postive

FASN	AC023983.1	0.81258747	5.32E-128	postive
ACOT6	AC011825.2	0.56938355	1.22E-47	postive
ELOVL2	AC122129.1	0.51464892	8.77E-38	postive
ALOX12	AC122129.1	0.6715979	5.95E-72	postive
ACSBG2	AC122129.1	0.6389611	3.51E-63	postive
CYP4B1	AC122129.1	0.58499225	8.38E-51	postive
FASN	AC122129.1	0.50389376	4.75E-36	postive
ALOX12	AC006064.1	0.69124225	8.55E-78	postive
ACOT6	PCCA-AS1	0.74205429	2.43E-95	postive
BMPR1B	PCCA-AS1	0.50826116	9.55E-37	postive
HSD17B3	AC013731.1	0.56875118	1.63E-47	postive
CPT1B	AC013731.1	0.53710443	1.31E-41	postive
ALOX12	AC013731.1	0.541741	1.95E-42	postive
ELOVL2	AC006238.1	0.67328641	1.95E-72	postive
ACSBG2	AC006238.1	0.54678242	2.38E-43	postive
CYP4B1	AC006238.1	0.75303017	1.10E-99	postive
FASN	AC006238.1	0.66720233	1.05E-70	postive
ELOVL2	AC244093.3	0.80325855	6.22E-123	postive
ACSBG2	AC244093.3	0.69657274	1.84E-79	postive
CYP4B1	AC244093.3	0.86699756	1.44E-164	postive
FASN	AC244093.3	0.79602258	3.47E-119	postive
ACOT6	AC023421.1	0.80285781	1.01E-122	postive

BMPR1B	AC023421.1	0.65892662	2.04E-68	postive
D2HGDH	AP001453.1	0.50638967	1.90E-36	postive
ALOX12	AP001453.1	0.53190334	1.07E-40	postive
ACSBG2	AP001453.1	0.51554363	6.25E-38	postive
ACOT6	CBR3-AS1	0.76212091	1.85E-103	postive
BMPR1B	CBR3-AS1	0.74201004	2.53E-95	postive
RDH11	CBR3-AS1	0.53264994	7.92E-41	postive
HACL1	ST3GAL6-AS1	0.50627508	1.99E-36	postive
RDH11	ST3GAL6-AS1	0.52082364	8.30E-39	postive
GABARAPL1	ST3GAL6-AS1	0.60240961	1.53E-54	postive
ELOVL2	AC006111.2	0.78617063	2.54E-114	postive
ACSBG2	AC006111.2	0.70396541	7.78E-82	postive
CYP4B1	AC006111.2	0.85268393	1.53E-153	postive
FASN	AC006111.2	0.77227568	6.97E-108	postive
ACOT6	AC012213.3	0.62955006	7.60E-61	postive
SDHD	AL135999.3	0.51795817	2.49E-38	postive
HACD3	AL135999.3	0.55498656	7.20E-45	postive
AUH	AL135999.3	0.53493597	3.15E-41	postive
ELOVL4	AL135999.3	0.61916836	2.32E-58	postive
RDH11	AL135999.3	0.50566327	2.49E-36	postive

GABARAPL1	AL135999.3	0.57049587	7.35E-48	postive
SDHD	AL512363.1	0.58248638	2.77E-50	postive
PHYH	AL512363.1	0.62439811	1.33E-59	postive
HACD3	AL512363.1	0.627013	3.14E-60	postive
AUH	AL512363.1	0.58247505	2.79E-50	postive
HACL1	AL512363.1	0.58675247	3.60E-51	postive
BMPR1B	AL512363.1	0.68755751	1.16E-76	postive
ELOVL4	AL512363.1	0.63758059	7.81E-63	postive
RDH11	AL512363.1	0.6993191	2.46E-80	postive
GABARAPL1	AL512363.1	0.55661456	3.56E-45	postive
ELOVL2	AC009404.1	0.50022098	1.80E-35	postive
ALOX12	AC009404.1	0.54554363	4.01E-43	postive
ACSBG2	AC009404.1	0.60060095	3.84E-54	postive
CYP4B1	AC009404.1	0.50890813	7.52E-37	postive
ELOVL2	MIS18A-AS1	0.77524231	3.22E-109	postive
ACSBG2	MIS18A-AS1	0.69798273	6.57E-80	postive
CYP4B1	MIS18A-AS1	0.84056185	4.44E-145	postive
FASN	MIS18A-AS1	0.76275707	9.91E-104	postive
ELOVL2	AC145343.1	0.58534672	7.07E-51	postive
CYP4B1	AC145343.1	0.55022164	5.56E-44	postive
FASN	AC145343.1	0.64006055	1.85E-63	postive

ACOT6	AC037487.2	0.55720183	2.75E-45	postive
ACOT6	AC025917.1	0.8138114	1.10E-128	postive
BMPR1B	AC025917.1	0.59174062	3.18E-52	postive
ELOVL2	AC127024.2	0.58236009	2.94E-50	postive
ACSBG2	AC127024.2	0.65728329	5.70E-68	postive
CYP4B1	AC127024.2	0.64090746	1.13E-63	postive
FASN	AC127024.2	0.58070043	6.46E-50	postive
ACSBG2	PANK2-AS1	0.51727747	3.23E-38	postive
ALOX12	AC008115.3	0.60242798	1.52E-54	postive
CPT1B	AC106782.5	0.52679845	8.10E-40	postive
D2HGDH	AC106782.5	0.56190209	3.49E-46	postive
ALOX12	AC106782.5	0.54641024	2.79E-43	postive
CPT1B	AC244197.2	0.50327084	5.96E-36	postive
D2HGDH	AC244197.2	0.5390789	5.84E-42	postive
ALOX12	AC244197.2	0.50001836	1.93E-35	postive
CPT1B	AC233728.1	0.66288484	1.68E-69	postive
D2HGDH	AC233728.1	0.61644838	1.00E-57	postive
ALOX12	AC233728.1	0.50037813	1.70E-35	postive
KMT5A	AP001453.2	0.51509179	7.41E-38	postive
CYP4B1	LINC01357	0.50845283	8.90E-37	postive
ACOT6	AC024267.5	0.5200681	1.11E-38	postive
CBR4	AC109347.1	0.60919752	4.63E-56	postive

ALOX12	DHDDS-AS1	0.5953096	5.46E-53	postive
CPT1B	AL022328.3	0.63851758	4.54E-63	postive
D2HGDH	AL022328.3	0.56067003	6.02E-46	postive
ALOX12	AL022328.3	0.55976518	8.97E-46	postive
ELOVL2	AC140847.2	0.78161803	3.69E-112	postive
ACSBG2	AC140847.2	0.63719535	9.76E-63	postive
CYP4B1	AC140847.2	0.85623821	3.62E-156	postive
FASN	AC140847.2	0.77522528	3.28E-109	postive
ELOVL2	CDKN2B-AS1	0.52286689	3.76E-39	postive
ACSBG2	CDKN2B-AS1	0.57684673	3.94E-49	postive
ELOVL2	AP001107.1	0.66634853	1.82E-70	postive
ALOX12	AP001107.1	0.63716335	9.94E-63	postive
ACSBG2	AP001107.1	0.71483571	1.83E-85	postive
CYP4B1	AP001107.1	0.71486179	1.80E-85	postive
FASN	AP001107.1	0.64351784	2.42E-64	postive
ELOVL2	AC015914.1	0.67400075	1.21E-72	postive
ACSBG2	AC015914.1	0.64644865	4.24E-65	postive
CYP4B1	AC015914.1	0.73297587	6.54E-92	postive
FASN	AC015914.1	0.65944446	1.48E-68	postive
ALOX12	AC116667.1	0.63930958	2.86E-63	postive
ACSBG2	AC116667.1	0.51337508	1.42E-37	postive

ACOT6	RHOQ-AS1	0.56873748	1.64E-47	postive
ALOX12	AC010761.3	0.56792184	2.37E-47	postive
CPT1B	ZKSCAN2-DT	0.69749566	9.38E-80	postive
D2HGDH	ZKSCAN2-DT	0.61866029	3.05E-58	postive
ALOX12	ZKSCAN2-DT	0.67829588	6.82E-74	postive
HSD17B3	AL008729.2	0.58582838	5.61E-51	postive
EHHADH	AC002401.2	0.54031198	3.52E-42	postive
ELOVL2	APP-DT	0.62099212	8.62E-59	postive
ACSBG2	APP-DT	0.53561223	2.40E-41	postive
CYP4B1	APP-DT	0.70158969	4.59E-81	postive
FASN	APP-DT	0.60145637	2.49E-54	postive
ELOVL2	AC009145.2	0.81478595	3.09E-129	postive
ACSBG2	AC009145.2	0.6806501	1.38E-74	postive
CYP4B1	AC009145.2	0.87955647	2.33E-175	postive
FASN	AC009145.2	0.80749303	3.37E-125	postive
HSD17B3	AL135999.1	0.50159445	1.10E-35	postive
CPT1B	AL135999.1	0.72468159	6.73E-89	postive
D2HGDH	AL135999.1	0.73013075	7.27E-91	postive
ACADVL	AL135999.1	0.56312784	2.03E-46	postive
ALOX12	AL135999.1	0.66663734	1.51E-70	postive
ELOVL2	AC092718.5	0.82348922	2.70E-134	postive

ACSBG2	AC092718.5	0.67381037	1.38E-72	postive
CYP4B1	AC092718.5	0.88804974	2.30E-183	postive
FASN	AC092718.5	0.82361232	2.28E-134	postive
ELOVL2	SRD5A3-AS1	0.72907061	1.77E-90	postive
ACSBG2	SRD5A3-AS1	0.64754322	2.20E-65	postive
CYP4B1	SRD5A3-AS1	0.7033251	1.26E-81	postive
FASN	SRD5A3-AS1	0.67247849	3.33E-72	postive
ELOVL2	AC074135.1	0.78626997	2.28E-114	postive
ACSBG2	AC074135.1	0.6392575	2.95E-63	postive
CYP4B1	AC074135.1	0.8098794	1.68E-126	postive
FASN	AC074135.1	0.7923883	2.32E-117	postive
ELOVL2	MZF1-AS1	0.61875092	2.91E-58	postive
ALOX12	MZF1-AS1	0.54808677	1.38E-43	postive
ACSBG2	MZF1-AS1	0.65612983	1.17E-67	postive
CYP4B1	MZF1-AS1	0.68020459	1.87E-74	postive
FASN	MZF1-AS1	0.6587514	2.28E-68	postive
CPT1B	AC010201.1	0.54216617	1.64E-42	postive
ALOX12	AC010201.1	0.61204123	1.04E-56	postive
ELOVL2	CATIP-AS2	0.5726336	2.76E-48	postive
CYP4B1	CATIP-AS2	0.64881798	1.02E-65	postive
FASN	CATIP-AS2	0.54924937	8.41E-44	postive
D2HGDH	AC007406.1	0.5076459	1.20E-36	postive
ALOX12	AC007406.1	0.50630537	1.96E-36	postive
ACOT6	MALAT1	0.64546529	7.63E-65	postive

ALOX12	LINC02145	0.56632202	4.86E-47	postive
CPT1B	LINC01772	0.51771282	2.74E-38	postive
D2HGDH	LINC01772	0.50745738	1.29E-36	postive
ALOX12	LINC01772	0.72246662	4.11E-88	postive
BMPR1B	KRTAP5-AS1	0.71437415	2.64E-85	postive
ELOVL4	KRTAP5-AS1	0.5190235	1.66E-38	postive
RDH11	KRTAP5-AS1	0.65152098	1.99E-66	postive
GABARAPL1	KRTAP5-AS1	0.63418288	5.51E-62	postive
ACACB	KRTAP5-AS1	0.56952667	1.14E-47	postive
ELOVL2	AC090515.5	0.75457553	2.58E-100	postive
ACSBG2	AC090515.5	0.64513683	9.28E-65	postive
CYP4B1	AC090515.5	0.82210276	1.80E-133	postive
FASN	AC090515.5	0.74126405	4.90E-95	postive
ACOT6	FP325332.1	0.74254053	1.58E-95	postive
BMPR1B	FP325332.1	0.50259074	7.63E-36	postive
ACOT6	AF117829.1	0.603199	1.03E-54	postive
ALOX12	AL021368.2	0.70257856	2.20E-81	postive
HSD17B3	RASGRP3-AS1	0.55016254	5.70E-44	postive
ELOVL2	AC067817.2	0.82374538	1.90E-134	postive
ACSBG2	AC067817.2	0.65459896	3.01E-67	postive
CYP4B1	AC067817.2	0.89582716	2.76E-191	postive

FASN	AC067817.2	0.80671519	8.88E-125	postive
HSD17B3	HNRNPD-DT	0.54210255	1.68E-42	postive
ELOVL2	AC005391.1	0.80138475	6.01E-122	postive
ACSBG2	AC005391.1	0.71592791	7.76E-86	postive
CYP4B1	AC005391.1	0.85285825	1.14E-153	postive
FASN	AC005391.1	0.78671804	1.39E-114	postive
CPT1B	AC009107.2	0.50223598	8.68E-36	postive
ALOX12	AC009107.2	0.56838009	1.92E-47	postive
ELOVL2	AC107959.1	0.69230168	4.01E-78	postive
ACSBG2	AC107959.1	0.54897759	9.43E-44	postive
CYP4B1	AC107959.1	0.74257759	1.53E-95	postive
FASN	AC107959.1	0.67450171	8.70E-73	postive
LGALS1	LINC01711	0.52958991	2.69E-40	postive
ALOX12	GRTP1-AS1	0.59473469	7.26E-53	postive
ALOX12	AP000355.1	0.57308334	2.25E-48	postive
ALOX12	AC034102.6	0.67801016	8.27E-74	postive
ELOVL2	AC012640.1	0.83026414	1.98E-138	postive
ACSBG2	AC012640.1	0.67421978	1.05E-72	postive
CYP4B1	AC012640.1	0.88970163	5.35E-185	postive
FASN	AC012640.1	0.83418909	6.55E-141	postive

CPT1B	AC098479.1	0.51463362	8.82E-38	postive
ACOT8	LINC00239	0.51456075	9.06E-38	postive
ELOVL4	LINC00239	0.58378243	1.50E-50	postive
ALOX12	ELOA-AS1	0.53179092	1.12E-40	postive
ELOVL2	AC005884.2	0.81138022	2.50E-127	postive
ACSBG2	AC005884.2	0.62245628	3.88E-59	postive
CYP4B1	AC005884.2	0.86765231	4.19E-165	postive
FASN	AC005884.2	0.81735001	1.07E-130	postive
ACOT6	AC002066.1	0.60775125	9.83E-56	postive
BMPR1B	AC002066.1	0.53752468	1.10E-41	postive
ALOX12	AC079075.1	0.56723644	3.22E-47	postive
ACSBG2	AC079075.1	0.64621503	4.88E-65	postive
ELOVL2	AC011377.1	0.51340154	1.40E-37	postive
ALOX12	AC011377.1	0.52958994	2.69E-40	postive
ACSBG2	AC011377.1	0.6488203	1.02E-65	postive
CYP4B1	AC011377.1	0.56841513	1.89E-47	postive
ALOX12	AP006623.1	0.6847847	8.01E-76	postive
ACSBG2	AP006623.1	0.58186016	3.73E-50	postive
CYP4B1	AP006623.1	0.50554362	2.60E-36	postive
ELOVL2	RC3H1-IT1	0.5969769	2.38E-53	postive
ACSBG2	RC3H1-IT1	0.65444373	3.31E-67	postive
CYP4B1	RC3H1-IT1	0.65343689	6.15E-67	postive

FASN	RC3H1-IT1	0.5809172	5.83E-50	postive
ELOVL2	AC078785.1	0.72979775	9.62E-91	postive
ACSBG2	AC078785.1	0.59106805	4.42E-52	postive
CYP4B1	AC078785.1	0.78982162	4.30E-116	postive
FASN	AC078785.1	0.7487988	5.54E-98	postive
ALOX12	AL139041.1	0.53613495	1.94E-41	postive
ALOX12	AL117339.3	0.52273358	3.96E-39	postive
HSD17B3	AC114730.3	0.52699398	7.50E-40	postive
CPT1B	AC114730.3	0.72630074	1.77E-89	postive
D2HGDH	AC114730.3	0.75812025	8.88E-102	postive
ACADVL	AC114730.3	0.54356495	9.16E-43	postive
ALOX12	AC114730.3	0.62837977	1.46E-60	postive
ELOVL2	AC138207.7	0.80702163	6.07E-125	postive
ACSBG2	AC138207.7	0.69016491	1.84E-77	postive
CYP4B1	AC138207.7	0.87240743	4.46E-169	postive
FASN	AC138207.7	0.80412216	2.17E-123	postive
ACOT6	DIAPH1-AS1	0.72188155	6.61E-88	postive
ACOT6	AC131182.1	0.69635409	2.16E-79	postive
D2HGDH	AC005264.1	0.52008788	1.10E-38	postive
ALOX12	AP000941.1	0.56974444	1.04E-47	postive
ACAA2	LINC00526	0.62042754	1.17E-58	postive

ALOX12	HLA-F-AS1	0.61609461	1.21E-57	postive
ELOVL2	AL161668.3	0.79645275	2.10E-119	postive
ACSBG2	AL161668.3	0.68741237	1.28E-76	postive
CYP4B1	AL161668.3	0.84699163	1.78E-149	postive
FASN	AL161668.3	0.78831306	2.34E-115	postive
PRKAA2	GNG12-AS1	0.52173605	5.84E-39	postive
ALOX12	AC032044.1	0.58324713	1.93E-50	postive
D2HGDH	AC112484.1	0.53123349	1.40E-40	postive
ACOT6	AL136221.1	0.67720723	1.42E-73	postive
CBR4	AL136221.1	0.50321451	6.08E-36	postive
ACOT6	AL356124.1	0.57178853	4.07E-48	postive
ALOX12	N4BP2L2-IT2	0.50091622	1.40E-35	postive
ACOT6	N4BP2L2-IT2	0.52826799	4.54E-40	postive
ELOVL2	AC008870.4	0.65886414	2.12E-68	postive
ALOX12	AC008870.4	0.52369772	2.73E-39	postive
ACSBG2	AC008870.4	0.7185103	9.97E-87	postive
CYP4B1	AC008870.4	0.71979399	3.56E-87	postive
FASN	AC008870.4	0.65770346	4.39E-68	postive
ELOVL2	AC010761.6	0.73151066	2.27E-91	postive
ACSBG2	AC010761.6	0.66448742	6.02E-70	postive
CYP4B1	AC010761.6	0.79653385	1.91E-119	postive

FASN	AC010761.6	0.75918197	3.20E-102	postive
ELOVL2	LINC02345	0.81850411	2.30E-131	postive
ACSBG2	LINC02345	0.64234696	4.84E-64	postive
CYP4B1	LINC02345	0.86715688	1.06E-164	postive
FASN	LINC02345	0.83082474	8.83E-139	postive
HSD17B3	PVT1	0.53162675	1.19E-40	postive
HSD17B3	AC005840.4	0.53157848	1.22E-40	postive
CPT1B	AC005840.4	0.57505087	9.07E-49	postive
CPT1B	IGBP1-AS1	0.51664782	4.11E-38	postive
ALOX12	IGBP1-AS1	0.56892536	1.50E-47	postive
HSD17B3	AC005332.5	0.56130792	4.54E-46	postive
CPT1B	AC005332.5	0.50045517	1.65E-35	postive
D2HGDH	AC005332.5	0.59188763	2.96E-52	postive
ACADVL	AC005332.5	0.52030648	1.01E-38	postive
ALOX12	AC005332.5	0.51825832	2.22E-38	postive
HSD17B3	AL096701.3	0.61996264	1.51E-58	postive
CPT1B	AL096701.3	0.51027731	4.52E-37	postive
ALOX12	AL096701.3	0.53919296	5.57E-42	postive
ACO2	MIR4435-2HG	-0.5225311	4.29E-39	negative
ACOT6	USP3-AS1	0.65743504	5.19E-68	postive

BMPR1B	USP3-AS1	0.54358546	9.08E-43	postive
ELOVL2	AC106820.4	0.69886627	3.44E-80	postive
ACSBG2	AC106820.4	0.58097958	5.66E-50	postive
CYP4B1	AC106820.4	0.72394882	1.23E-88	postive
FASN	AC106820.4	0.71507013	1.53E-85	postive
ALOX12	XPC-AS1	0.71429567	2.80E-85	postive
ACOT6	AC016949.1	0.65665353	8.44E-68	postive
ALOX12	AC008750.4	0.70454626	5.03E-82	postive
SERINC1	LINC01801	0.54092814	2.73E-42	postive
CPT1B	AC006435.2	0.72797159	4.43E-90	postive
D2HGDH	AC006435.2	0.58326958	1.91E-50	postive
ALOX12	AC006435.2	0.60080893	3.46E-54	postive
ELOVL2	AC079313.1	0.73988388	1.66E-94	postive
ACSBG2	AC079313.1	0.69514799	5.18E-79	postive
CYP4B1	AC079313.1	0.77919591	4.96E-111	postive
FASN	AC079313.1	0.72435661	8.79E-89	postive
ACOT6	AC006213.5	0.5961552	3.58E-53	postive
ACSBG2	AC009996.1	0.59021422	6.71E-52	postive
CYP4B1	AC009996.1	0.5101378	4.76E-37	postive
ACOT6	AC009996.1	0.51515511	7.24E-38	postive
ACAA2	ADAMTS9-AS1	0.50197881	9.53E-36	postive

HSD17B3	AC024060.2	0.52735877	6.49E-40	postive
CPT1B	AC024060.2	0.61777104	4.93E-58	postive
ELOVL2	LINC02340	0.84067243	3.74E-145	postive
ACSBG2	LINC02340	0.68134557	8.57E-75	postive
CYP4B1	LINC02340	0.88944132	9.72E-185	postive
FASN	LINC02340	0.83231756	1.02E-139	postive
SUCLG1	DHRS4-AS1	0.51207558	2.31E-37	postive
MMUT	DHRS4-AS1	0.51473692	8.48E-38	postive
DLST	DHRS4-AS1	0.53087198	1.61E-40	postive
HSD17B3	AC127502.2	0.57214434	3.46E-48	postive
CPT1B	AC127502.2	0.53203058	1.02E-40	postive
SUCLG1	AC008991.1	0.62150896	6.51E-59	postive
PCBD1	AC008991.1	0.51649614	4.35E-38	postive
ALOX12	AL162724.2	0.57952897	1.12E-49	postive
HSD17B3	AP003352.1	0.56893168	1.50E-47	postive
CPT1B	AP003352.1	0.54856712	1.12E-43	postive
ELOVL2	LINC02642	0.51455995	9.07E-38	postive
ACSBG2	LINC02642	0.58593205	5.34E-51	postive
CYP4B1	LINC02642	0.54716934	2.03E-43	postive
FASN	LINC02642	0.50505242	3.11E-36	postive
ELOVL2	ATP2B1-AS1	0.6786788	5.26E-74	postive

ACSBG2	ATP2B1-AS1	0.5153193	6.80E-38	postive
CYP4B1	ATP2B1-AS1	0.73110277	3.20E-91	postive
FASN	ATP2B1-AS1	0.66670814	1.44E-70	postive
ELOVL2	AC124017.1	0.7417136	3.29E-95	postive
ACSBG2	AC124017.1	0.57430702	1.28E-48	postive
CYP4B1	AC124017.1	0.79666192	1.64E-119	postive
FASN	AC124017.1	0.74292732	1.12E-95	postive
ALOX12	FIGNL2-DT	0.5065689	1.78E-36	postive
ALOX12	LINC00641	0.50841188	9.03E-37	postive
ACOT6	AC068700.1	0.85504596	2.81E-155	postive
BMPR1B	AC068700.1	0.79579376	4.54E-119	postive
PRKAG2	AC055720.2	0.53839064	7.74E-42	postive
PDHB	AC055720.2	0.57580507	6.39E-49	postive
GABARAPL1	AC055720.2	0.66966874	2.11E-71	postive
ACACB	AC055720.2	0.56051095	6.46E-46	postive
ALOX12	LINC01402	0.54881478	1.01E-43	postive
RDH11	AC087045.2	0.54051089	3.24E-42	postive
GABARAPL1	AC087045.2	0.51368471	1.26E-37	postive
ALOX12	AL731566.2	0.6128943	6.64E-57	postive
ELOVL2	AC011481.2	0.55163371	3.05E-44	postive
CYP4B1	AC011481.2	0.60050374	4.04E-54	postive
FASN	AC011481.2	0.51004445	4.93E-37	postive
ALOX12	LINC01320	0.5046075	3.66E-36	postive

ACOT6	AC068792.1	0.50164783	1.07E-35	postive
ELOVL2	SCAT1	0.61121215	1.61E-56	postive
CYP4B1	SCAT1	0.64071867	1.26E-63	postive
FASN	SCAT1	0.65025877	4.28E-66	postive
ACOT6	AC090948.1	0.66813403	5.73E-71	postive
ACOT6	AC011511.5	0.69402825	1.16E-78	postive
BMPR1B	AC011511.5	0.65184284	1.63E-66	postive
ELOVL2	AC022167.3	0.82840495	2.81E-137	postive
ACSBG2	AC022167.3	0.6584062	2.83E-68	postive
CYP4B1	AC022167.3	0.89070365	5.32E-186	postive
FASN	AC022167.3	0.83052091	1.37E-138	postive
ELOVL2	TUBA3FP	0.77316668	2.78E-108	postive
ACSBG2	TUBA3FP	0.64297348	3.34E-64	postive
CYP4B1	TUBA3FP	0.82309243	4.66E-134	postive
FASN	TUBA3FP	0.75084085	8.44E-99	postive
ELOVL2	AL512274.1	0.61237607	8.74E-57	postive
ACSBG2	AL512274.1	0.50229433	8.50E-36	postive
CYP4B1	AL512274.1	0.65656687	8.91E-68	postive
FASN	AL512274.1	0.66063683	6.97E-69	postive
ACOT6	AC009237.15	0.65691479	7.17E-68	postive
BMPR1B	AC009237.15	0.50351714	5.45E-36	postive

HSD17B3	KIF1C-AS1	0.5278021	5.45E-40	postive
CPT1B	KIF1C-AS1	0.55249934	2.10E-44	postive
ALOX12	KIF1C-AS1	0.54533239	4.38E-43	postive
ALDH3A2	LINC01550	0.5717789	4.09E-48	postive
ACOT6	AC106028.2	0.66383496	9.14E-70	postive
HSD17B3	AC005387.2	0.5175976	2.86E-38	postive
CPT1B	AC005387.2	0.59317152	1.57E-52	postive
D2HGDH	AC005387.2	0.55781434	2.11E-45	postive
ALOX12	AC005387.2	0.66824686	5.33E-71	postive
CBR4	AL078581.2	0.54804228	1.40E-43	postive
HSD17B3	NALT1	0.55853972	1.53E-45	postive
CPT1B	NALT1	0.51443618	9.50E-38	postive
D2HGDH	NALT1	0.55882306	1.36E-45	postive
ELOVL2	AL451085.1	0.79952604	5.57E-121	postive
ACSBG2	AL451085.1	0.64604919	5.39E-65	postive
CYP4B1	AL451085.1	0.86991027	5.69E-167	postive
FASN	AL451085.1	0.79112095	9.86E-117	postive
ACOT6	TCEAL3-AS1	0.73117215	3.02E-91	postive
ELOVL2	AC005775.1	0.80008453	2.86E-121	postive
ACSBG2	AC005775.1	0.65643241	9.68E-68	postive
CYP4B1	AC005775.1	0.8515017	1.11E-152	postive
FASN	AC005775.1	0.80000347	3.15E-121	postive

ELOVL2	LINC02749	0.65688253	7.32E-68	postive
CYP4B1	LINC02749	0.61632098	1.07E-57	postive
FASN	LINC02749	0.6282745	1.55E-60	postive
ACOT6	PLAC4	0.91866319	9.35E-219	postive
BMPR1B	PLAC4	0.71399104	3.56E-85	postive
HSD17B3	FSIP2-AS1	0.58698217	3.22E-51	postive
ALOX12	FSIP2-AS1	0.55461772	8.45E-45	postive
ALOX12	LINC00663	0.59723952	2.08E-53	postive
D2HGDH	AC127521.1	0.50406547	4.46E-36	postive
ALOX12	AC127521.1	0.60341742	9.17E-55	postive
ACSBG2	AC127521.1	0.51740374	3.08E-38	postive
CPT1B	AL031705.1	0.55101494	3.97E-44	postive
D2HGDH	AL031705.1	0.59147598	3.62E-52	postive
ALOX12	AL031705.1	0.71018094	6.88E-84	postive
ACOT6	AL035448.1	0.60299326	1.14E-54	postive
ACOT6	FARP1-AS1	0.53668813	1.55E-41	postive
HSD17B3	AC008875.1	0.54669449	2.47E-43	postive
CPT1B	AC008875.1	0.55968631	9.28E-46	postive
DLST	AC004825.2	0.51921465	1.54E-38	postive
ALDH3A2	MAPT-AS1	0.54166851	2.01E-42	postive
ELOVL2	AC002550.2	0.79726859	8.06E-120	postive

ACSBG2	AC002550.2	0.69536182	4.43E-79	postive
CYP4B1	AC002550.2	0.8552827	1.87E-155	postive
FASN	AC002550.2	0.77982572	2.53E-111	postive
PRKAG2	LINC02343	0.51426986	1.01E-37	postive
AUH	LINC02343	0.50237826	8.24E-36	postive
ACOT6	LINC02343	0.63674653	1.26E-62	postive
BMPR1B	LINC02343	0.88565762	4.78E-181	postive
RDH11	LINC02343	0.70186763	3.73E-81	postive
GABARAPL1	LINC02343	0.59377848	1.17E-52	postive
ACACB	LINC02343	0.5850185	8.28E-51	postive
ELOVL2	LINC02147	0.82063082	1.33E-132	postive
ACSBG2	LINC02147	0.66247398	2.18E-69	postive
CYP4B1	LINC02147	0.89089627	3.40E-186	postive
FASN	LINC02147	0.80745575	3.53E-125	postive
ELOVL2	AL513477.2	0.64855938	1.20E-65	postive
ACSBG2	AL513477.2	0.67183723	5.08E-72	postive
CYP4B1	AL513477.2	0.71529957	1.27E-85	postive
FASN	AL513477.2	0.64068881	1.28E-63	postive
CPT1B	AC109460.2	0.58870027	1.40E-51	postive
D2HGDH	AC109460.2	0.54293737	1.19E-42	postive
ALOX12	AC109460.2	0.79220017	2.88E-117	postive

ELOVL2	AC090229.1	0.83241903	8.76E-140	postive
ACSBG2	AC090229.1	0.64169632	7.09E-64	postive
CYP4B1	AC090229.1	0.8760118	3.39E-172	postive
FASN	AC090229.1	0.82262139	8.87E-134	postive
ALOX12	TH2LCRR	0.50141153	1.17E-35	postive
ACOT6	TH2LCRR	0.52248968	4.36E-39	postive
HSD17B3	AL049780.1	0.56565934	6.55E-47	postive
ALOX12	AL049780.1	0.55308392	1.64E-44	postive
ACSBG2	AL049780.1	0.52126153	7.01E-39	postive
ELOVL2	AC026904.2	0.78386182	3.22E-113	postive
ACSBG2	AC026904.2	0.58087461	5.95E-50	postive
CYP4B1	AC026904.2	0.84286413	1.25E-146	postive
FASN	AC026904.2	0.75597081	6.90E-101	postive
ALOX12	AL157392.4	0.55227958	2.31E-44	postive
ACOT6	AC069029.1	0.70181714	3.88E-81	postive
ELOVL2	HCG14	0.79886997	1.22E-120	postive
ACSBG2	HCG14	0.68303266	2.69E-75	postive
CYP4B1	HCG14	0.86340828	1.09E-161	postive
FASN	HCG14	0.78897958	1.11E-115	postive
CYP4B1	AC007681.1	0.52484664	1.74E-39	postive
ALOX12	AC127024.4	0.65595113	1.30E-67	postive

ACSBG2	AC127024.4	0.59893063	8.93E-54	postive
ACOT6	SNHG14	0.55930311	1.10E-45	postive
CBR4	SNHG14	0.54067052	3.04E-42	postive
ALOX12	CRYZL2P-SEC16B	0.69023214	1.75E-77	postive
ALOX12	AL450998.2	0.71672996	4.11E-86	postive
ACSBG2	AL450998.2	0.54146582	2.19E-42	postive
ALOX12	AL390208.1	0.53933253	5.26E-42	postive
ELOVL2	AC091180.5	0.83537539	1.13E-141	postive
ACSBG2	AC091180.5	0.67903534	4.13E-74	postive
CYP4B1	AC091180.5	0.88786069	3.52E-183	postive
FASN	AC091180.5	0.82946816	6.19E-138	postive
ELOVL2	AC007098.1	0.67075367	1.04E-71	postive
ALOX12	AC007098.1	0.5149879	7.71E-38	postive
ACSBG2	AC007098.1	0.66363762	1.04E-69	postive
CYP4B1	AC007098.1	0.69883302	3.52E-80	postive
FASN	AC007098.1	0.63088992	3.57E-61	postive
CYP4B1	LINC01521	0.51041076	4.30E-37	postive
FASN	LINC01521	0.51169688	2.66E-37	postive
ALOX12	AL162727.2	0.50051886	1.61E-35	postive
ALOX12	ALG13-AS1	0.54590186	3.45E-43	postive
BMPR1B	AC116351.1	0.52002088	1.13E-38	postive

GABARAPL1	AC116351.1	0.50240169	8.17E-36	postive
ACACB	AC116351.1	0.53084852	1.63E-40	postive
CPT1B	ASMTL-AS1	0.6185632	3.22E-58	postive
D2HGDH	ASMTL-AS1	0.57956709	1.10E-49	postive
ELOVL2	AC002116.2	0.70951279	1.15E-83	postive
ACSBG2	AC002116.2	0.59015423	6.91E-52	postive
CYP4B1	AC002116.2	0.74069422	8.11E-95	postive
FASN	AC002116.2	0.71910262	6.21E-87	postive
HSD17B3	AC074117.1	0.56812511	2.16E-47	postive
CPT1B	AC074117.1	0.64481586	1.12E-64	postive
D2HGDH	AC074117.1	0.5919696	2.84E-52	postive
ALOX12	AC074117.1	0.6129976	6.29E-57	postive
ACOT6	AL109761.1	0.55261138	2.00E-44	postive
ELOVL2	AC008543.3	0.81985491	3.78E-132	postive
ACSBG2	AC008543.3	0.68893766	4.38E-77	postive
CYP4B1	AC008543.3	0.88607047	1.92E-181	postive
FASN	AC008543.3	0.82061508	1.36E-132	postive
ACSBG2	CXXC5-AS1	0.50121392	1.26E-35	postive
ALOX12	CACNA1C-AS1	0.56927581	1.28E-47	postive
ACOT6	AC097382.1	0.92132216	1.80E-222	postive
BMPR1B	AC097382.1	0.71550075	1.09E-85	postive
ACOT6	AC006059.1	0.59093038	4.73E-52	postive

BMPR1B	AC006059.1	0.50469366	3.55E-36	postive
ELOVL2	AC004839.2	0.78100183	7.17E-112	postive
ACSBG2	AC004839.2	0.67306069	2.26E-72	postive
CYP4B1	AC004839.2	0.85451329	6.96E-155	postive
FASN	AC004839.2	0.77181152	1.12E-107	postive
ACOT6	AC072039.2	0.6074056	1.18E-55	postive
D2HGDH	AC018653.3	0.57703073	3.61E-49	postive
ACADVL	AC018653.3	0.52538998	1.41E-39	postive
ALOX12	AC018653.3	0.6002495	4.59E-54	postive
ELOVL2	AL591178.1	0.81585933	7.60E-130	postive
ACSBG2	AL591178.1	0.67423112	1.04E-72	postive
CYP4B1	AL591178.1	0.88108709	9.32E-177	postive
FASN	AL591178.1	0.81873349	1.70E-131	postive
ALOX12	AC005332.7	0.61451733	2.81E-57	postive
ELOVL2	AC104653.1	0.6317426	2.21E-61	postive
ACSBG2	AC104653.1	0.51559429	6.13E-38	postive
CYP4B1	AC104653.1	0.64754389	2.20E-65	postive
FASN	AC104653.1	0.62941754	8.18E-61	postive
ACOT6	AC037198.1	0.75440591	3.03E-100	postive
ELOVL2	AC091965.1	0.68909926	3.91E-77	postive
ACSBG2	AC091965.1	0.57777725	2.55E-49	postive
CYP4B1	AC091965.1	0.7663501	2.83E-105	postive

FASN	AC091965.1	0.69277874	2.85E-78	postive
ALOX12	AL109659.2	0.60151352	2.42E-54	postive
ELOVL2	RHOA-IT1	0.66593069	2.38E-70	postive
ALOX12	RHOA-IT1	0.53285689	7.29E-41	postive
ACSBG2	RHOA-IT1	0.71599384	7.36E-86	postive
CYP4B1	RHOA-IT1	0.72027249	2.43E-87	postive
FASN	RHOA-IT1	0.65159901	1.89E-66	postive
ACOT6	TRHDE-AS1	0.60209157	1.80E-54	postive
BMPR1B	TRHDE-AS1	0.5073293	1.35E-36	postive
CPT1B	ADIRF-AS1	0.61163867	1.29E-56	postive
D2HGDH	ADIRF-AS1	0.5540703	1.07E-44	postive
ACOT6	AC020978.1	0.55150943	3.21E-44	postive
ACSBG1	LINC01606	0.79236923	2.37E-117	postive
ENO3	LINC01606	0.74851808	7.16E-98	postive
PCBD1	LINC01606	0.6588299	2.17E-68	postive
ACOT6	AC012085.2	0.55710195	2.88E-45	postive
ELOVL2	AC078883.2	0.62085621	9.28E-59	postive
ACSBG2	AC078883.2	0.55058081	4.77E-44	postive
CYP4B1	AC078883.2	0.68166561	6.88E-75	postive
ACOT6	AC078883.2	0.61797395	4.42E-58	postive
BMPR1B	AC078883.2	0.53714607	1.29E-41	postive
FASN	AC078883.2	0.60381424	7.48E-55	postive

ACSBG2	AL358472.3	0.51474372	8.46E-38	postive
D2HGDH	UICLM	0.54953243	7.46E-44	postive
ALOX12	AC010531.5	0.59135891	3.83E-52	postive
ACOT6	MIR646HG	0.53564145	2.37E-41	postive
BMPR1B	MIR646HG	0.64702488	3.01E-65	postive
ALOX12	AC010168.2	0.59452484	8.05E-53	postive
ACOT6	AC010168.2	0.59380931	1.15E-52	postive
ACOT6	AC008883.1	0.55710195	2.88E-45	postive
ACSBG2	LINC01285	0.55302857	1.67E-44	postive
ELOVL2	AC025171.3	0.62463285	1.17E-59	postive
ACSBG2	AC025171.3	0.60984865	3.30E-56	postive
CYP4B1	AC025171.3	0.6502682	4.25E-66	postive
FASN	AC025171.3	0.59990051	5.47E-54	postive
ELOVL2	Z97200.1	0.80708279	5.62E-125	postive
ACSBG2	Z97200.1	0.62480032	1.07E-59	postive
CYP4B1	Z97200.1	0.84813082	2.82E-150	postive
FASN	Z97200.1	0.81062483	6.54E-127	postive
ACOT6	MAGI1-IT1	0.89893401	1.26E-194	postive
BMPR1B	MAGI1-IT1	0.66611124	2.12E-70	postive
ACOT6	AC016292.1	0.83701873	9.71E-143	postive
BMPR1B	AC016292.1	0.61428033	3.19E-57	postive

ACOT6	AC090579.1	0.69532911	4.54E-79	postive
BMPR1B	AC090579.1	0.51509357	7.41E-38	postive
CPT1B	AC002128.1	0.55777557	2.14E-45	postive
ALOX12	AC002128.1	0.64800174	1.67E-65	postive
DPEP2	PTPRN2-AS1	0.59031926	6.37E-52	postive
TBXAS1	PTPRN2-AS1	0.77981365	2.56E-111	postive
ALOX12	AC126118.1	0.61973425	1.71E-58	postive
ACSBG2	AC126118.1	0.52890725	3.52E-40	postive
ELOVL2	AC025048.4	0.81272932	4.44E-128	postive
ACSBG2	AC025048.4	0.65274756	9.39E-67	postive
CYP4B1	AC025048.4	0.87247505	3.91E-169	postive
FASN	AC025048.4	0.82521244	2.49E-135	postive
ALOX12	TPM1-AS	0.5144359	9.50E-38	postive
ACOT6	NADK2-AS1	0.66240679	2.27E-69	postive
ALOX12	AC021205.3	0.58730472	2.76E-51	postive
ELOVL2	AC008759.2	0.52974107	2.53E-40	postive
ALOX12	AC008759.2	0.55451169	8.84E-45	postive
ACSBG2	AC008759.2	0.61990816	1.55E-58	postive
CYP4B1	AC008759.2	0.59253045	2.16E-52	postive
CPT1B	AC103706.1	0.53344253	5.76E-41	postive
CPT1B	PKD1P6-NPIPP1	0.50893282	7.45E-37	postive

D2HGDH	PKD1P6-NPIPP1	0.62288667	3.06E-59	postive
ALOX12	PKD1P6-NPIPP1	0.53625707	1.85E-41	postive
ELOVL2	AC093206.1	0.83396905	9.05E-141	postive
ACSBG2	AC093206.1	0.66425467	6.99E-70	postive
CYP4B1	AC093206.1	0.89376462	3.99E-189	postive
FASN	AC093206.1	0.82861097	2.10E-137	postive
PON1	LINC01146	0.68787686	9.24E-77	postive
ADH1A	LINC01146	0.68733335	1.35E-76	postive
ADH4	LINC01146	0.68118405	9.57E-75	postive
FABP1	LINC01146	0.63339124	8.65E-62	postive
ADH1C	LINC01146	0.6602446	8.92E-69	postive
RDH16	LINC01146	0.69284052	2.73E-78	postive
ADH1B	LINC01146	0.59659101	2.88E-53	postive
ALOX12	AL683813.1	0.61799638	4.37E-58	postive
ACSBG2	AL683813.1	0.56800288	2.28E-47	postive
BMPR1B	AL118522.1	0.66939008	2.53E-71	postive
RDH11	AL118522.1	0.51186089	2.50E-37	postive
ALOX12	TBC1D8-AS1	0.5009961	1.36E-35	postive
ACOT6	AP000786.1	0.5613989	4.36E-46	postive
HSD17B3	SCGB1B2P	0.57410491	1.40E-48	postive
ELOVL2	PCAT1	0.73251789	9.66E-92	postive
ACSBG2	PCAT1	0.67801093	8.27E-74	postive

CYP4B1	PCAT1	0.79582613	4.37E-119	postive
FASN	PCAT1	0.70223633	2.84E-81	postive
ELOVL2	AL731569.1	0.54535759	4.33E-43	postive
ALOX12	AL731569.1	0.62196548	5.07E-59	postive
ACSBG2	AL731569.1	0.64770753	2.00E-65	postive
CYP4B1	AL731569.1	0.60195461	1.93E-54	postive
FASN	AL731569.1	0.55619702	4.26E-45	postive
ACSBG1	FAM27E3	0.69032625	1.64E-77	postive
ENO3	FAM27E3	0.69872446	3.81E-80	postive
PCBD1	FAM27E3	0.53466102	3.52E-41	postive
ELOVL2	AC074032.1	0.56066569	6.03E-46	postive
ACSBG2	AC074032.1	0.62083436	9.40E-59	postive
CYP4B1	AC074032.1	0.62839625	1.45E-60	postive
FASN	AC074032.1	0.50891648	7.49E-37	postive
ALOX12	COL4A2-AS1	0.52493445	1.68E-39	postive
TBXAS1	AL161785.1	0.54919154	8.62E-44	postive
ELOVL2	AC105020.5	0.78879367	1.37E-115	postive
ACSBG2	AC105020.5	0.70244423	2.43E-81	postive
CYP4B1	AC105020.5	0.86588838	1.14E-163	postive
FASN	AC105020.5	0.77201543	9.11E-108	postive

ELOVL2	AL391097.2	0.83001447	2.83E-138	postive
ACSBG2	AL391097.2	0.66915852	2.94E-71	postive
CYP4B1	AL391097.2	0.89151767	8.01E-187	postive
FASN	AL391097.2	0.82461671	5.70E-135	postive
ACOT6	TSC22D1-AS1	0.66491999	4.57E-70	postive
BMPR1B	TSC22D1-AS1	0.53238394	8.81E-41	postive
ACOT6	AC003682.1	0.55078695	4.37E-44	postive
ALOX12	AL590729.1	0.57375419	1.65E-48	postive
ACOT6	AC090181.2	0.57290319	2.44E-48	postive
ELOVL2	AC005730.3	0.72501533	5.12E-89	postive
ACSBG2	AC005730.3	0.67540589	4.76E-73	postive
CYP4B1	AC005730.3	0.79042808	2.16E-116	postive
FASN	AC005730.3	0.71082072	4.20E-84	postive
CPT1B	AC004951.4	0.55557008	5.59E-45	postive
ALOX12	AC004951.4	0.51602979	5.20E-38	postive
SDHD	AP003068.2	0.57855805	1.77E-49	postive
HACD3	AP003068.2	0.55365742	1.28E-44	postive
AUH	AP003068.2	0.6064206	1.96E-55	postive
ACOT6	AL158163.2	0.54011878	3.81E-42	postive
ACOT6	AC009716.1	0.52484766	1.74E-39	postive
ELOVL2	AL162430.2	0.59569725	4.50E-53	postive

ACSBG2	AL162430.2	0.65088722	2.92E-66	postive
CYP4B1	AL162430.2	0.65127636	2.31E-66	postive
FASN	AL162430.2	0.57856873	1.76E-49	postive
SDHD	AL157911.1	0.56890291	1.52E-47	postive
HACD3	AL157911.1	0.55502201	7.09E-45	postive
AUH	AL157911.1	0.5290288	3.36E-40	postive
ACOT6	AL157911.1	0.61464634	2.62E-57	postive
BMPR1B	AL157911.1	0.76411658	2.60E-104	postive
ELOVL4	AL157911.1	0.66145357	4.16E-69	postive
RDH11	AL157911.1	0.64820462	1.48E-65	postive
GABARAPL1	AL157911.1	0.58597795	5.22E-51	postive
ACACB	AL157911.1	0.54396724	7.75E-43	postive
ALOX12	AC110015.1	0.52452974	1.97E-39	postive
ACOT6	AL158832.1	0.65813407	3.35E-68	postive
HSD17B3	AC073896.4	0.52220928	4.86E-39	postive
SERINC1	AC073896.4	-0.5167934	3.89E-38	negative
CPT1B	VPS9D1-AS1	0.57876222	1.61E-49	postive
CBR4	RBM26-AS1	0.55673621	3.37E-45	postive
ALOX12	AC008655.2	0.58265044	2.56E-50	postive
ACSBG2	AC008655.2	0.57020053	8.41E-48	postive
CYP4B1	AC008655.2	0.50821738	9.71E-37	postive

ELOVL2	AC060766.5	0.63268354	1.29E-61	postive
ACSBG2	AC060766.5	0.58074866	6.31E-50	postive
CYP4B1	AC060766.5	0.68691712	1.81E-76	postive
FASN	AC060766.5	0.60486037	4.38E-55	postive
CYP4B1	LINC01750	0.54658588	2.59E-43	postive
ACOT6	MIR99AHG	0.60536206	3.38E-55	postive
BMPR1B	MIR99AHG	0.50765627	1.19E-36	postive
CBR4	MIR99AHG	0.50396966	4.62E-36	postive
ALOX12	AL513190.1	0.63124639	2.92E-61	postive
ELOVL2	AC024267.3	0.6577139	4.36E-68	postive
ALOX12	AC024267.3	0.54453327	6.12E-43	postive
ACSBG2	AC024267.3	0.68671779	2.08E-76	postive
CYP4B1	AC024267.3	0.73190338	1.63E-91	postive
FASN	AC024267.3	0.66881631	3.68E-71	postive
PRKAA2	LINC00271	0.54356182	9.17E-43	postive
ACOT6	AC005034.2	0.68927936	3.44E-77	postive
BMPR1B	AC005034.2	0.52651884	9.04E-40	postive
D2HGDH	AC020558.2	0.507985	1.06E-36	postive
PRKAG2	SCAMP1-AS1	0.56191508	3.47E-46	postive
ACOT8	SCAMP1-AS1	0.54031355	3.52E-42	postive
SDHD	SCAMP1-AS1	0.57804032	2.26E-49	postive
PHYH	SCAMP1-AS1	0.5185384	2.00E-38	postive

PDHB	SCAMP1-AS1	0.50184293	1.00E-35	postive
HSD17B8	SCAMP1-AS1	0.50561161	2.53E-36	postive
GCDH	SCAMP1-AS1	0.52332798	3.15E-39	postive
HACD3	SCAMP1-AS1	0.61428859	3.17E-57	postive
AUH	SCAMP1-AS1	0.60141093	2.55E-54	postive
HACL1	SCAMP1-AS1	0.56592691	5.81E-47	postive
ALOX12	RUFY1-AS1	0.70329893	1.28E-81	postive
ELOVL2	AC135178.6	0.56128134	4.60E-46	postive
ACSBG2	AC135178.6	0.57819892	2.09E-49	postive
CYP4B1	AC135178.6	0.5721027	3.53E-48	postive
FASN	AC135178.6	0.57821601	2.08E-49	postive
ALOX12	AL132780.1	0.56614589	5.26E-47	postive
ACSBG2	AL132780.1	0.50034676	1.72E-35	postive
ALOX12	AP001793.1	0.61309308	5.98E-57	postive
ELOVL2	PPP1R12A-AS1	0.62870492	1.22E-60	postive
ACSBG2	PPP1R12A-AS1	0.58157206	4.28E-50	postive
CYP4B1	PPP1R12A-AS1	0.71116692	3.21E-84	postive
FASN	PPP1R12A-AS1	0.58161065	4.20E-50	postive
CBR4	BDNF-AS	0.51636176	4.58E-38	postive
DPEP2	LINC02285	0.54570757	3.74E-43	postive
TBXAS1	LINC02285	0.72839873	3.10E-90	postive

AUH	AC069224.1	0.50204751	9.29E-36	postive
BMPR1B	AC069224.1	0.61422859	3.28E-57	postive
GABARAPL1	AC069224.1	0.55334077	1.46E-44	postive
ACACB	AC069224.1	0.60130696	2.69E-54	postive
ALOX12	AL080317.2	0.51291234	1.69E-37	postive
ALOX12	AC116552.1	0.58902619	1.20E-51	postive
ACOT6	AC109361.2	0.90606939	9.89E-203	postive
BMPR1B	AC109361.2	0.73455498	1.70E-92	postive
ACOT6	AL445309.1	0.50443382	3.90E-36	postive
D2HGDH	LINC01277	0.54726712	1.94E-43	postive
CPT1B	AC015961.2	0.55232627	2.26E-44	postive
ALOX12	AC084824.3	0.51629579	4.70E-38	postive
ACOT6	AC084824.3	0.66371061	9.90E-70	postive
ACOT6	AC136424.2	0.50379915	4.92E-36	postive
ELOVL2	AC023043.3	0.64936466	7.35E-66	postive
ACSBG2	AC023043.3	0.51197865	2.39E-37	postive
CYP4B1	AC023043.3	0.65944936	1.47E-68	postive
FASN	AC023043.3	0.71163149	2.24E-84	postive
ALOX12	AC005899.1	0.6015896	2.33E-54	postive
ACSBG2	AC005899.1	0.61095687	1.84E-56	postive
ELOVL2	AL391095.3	0.7913727	7.40E-117	postive

ACSBG2	AL391095.3	0.74839358	8.02E-98	postive
CYP4B1	AL391095.3	0.83824317	1.53E-143	postive
FASN	AL391095.3	0.78617344	2.53E-114	postive
ELOVL2	AC011498.1	0.83625608	3.04E-142	postive
ACSBG2	AC011498.1	0.67303922	2.30E-72	postive
CYP4B1	AC011498.1	0.894425	8.20E-190	postive
FASN	AC011498.1	0.83048842	1.43E-138	postive
ELOVL2	LINC02572	0.76027072	1.12E-102	postive
ACSBG2	LINC02572	0.60989616	3.22E-56	postive
CYP4B1	LINC02572	0.81032816	9.53E-127	postive
FASN	LINC02572	0.72285019	3.01E-88	postive
ALOX12	AC007292.2	0.61170767	1.24E-56	postive
ACOT6	AC026124.2	0.59732915	1.99E-53	postive
ALOX12	AC027020.1	0.5264261	9.37E-40	postive
ACSBG2	AC027020.1	0.53366114	5.27E-41	postive
ACADL	IQCA1-AS1	0.52576483	1.22E-39	postive
HSD17B3	PARD3-AS1	0.51521042	7.09E-38	postive
ALOX12	AC096992.2	0.60113187	2.94E-54	postive
ALOX12	AC011447.3	0.53310131	6.61E-41	postive
ACSBG2	AC011447.3	0.56321341	1.95E-46	postive
ELOVL2	AC135050.4	0.70801483	3.62E-83	postive

ACSBG2	AC135050.4	0.72417184	1.02E-88	postive
CYP4B1	AC135050.4	0.75652902	4.06E-101	postive
FASN	AC135050.4	0.71899858	6.75E-87	postive
ACOT6	AL450263.1	0.77995806	2.20E-111	postive
BMPR1B	AL450263.1	0.79466765	1.68E-118	postive
RDH11	AL450263.1	0.53764362	1.05E-41	postive
GABARAPL1	AL450263.1	0.53221473	9.43E-41	postive
ACACB	AL450263.1	0.62980169	6.59E-61	postive
CYP4B1	AC011472.2	0.51667136	4.07E-38	postive
ELOVL2	AC067930.2	0.65134635	2.21E-66	postive
ACSBG2	AC067930.2	0.71027622	6.39E-84	postive
CYP4B1	AC067930.2	0.68251131	3.85E-75	postive
FASN	AC067930.2	0.64221743	5.22E-64	postive
PRKAG2	PART1	0.65657729	8.85E-68	postive
SDHD	PART1	0.65760257	4.67E-68	postive
PHYH	PART1	0.60876416	5.81E-56	postive
PDHB	PART1	0.51839085	2.11E-38	postive
HACD3	PART1	0.70581524	1.93E-82	postive
AUH	PART1	0.52328344	3.20E-39	postive
HACL1	PART1	0.56087213	5.51E-46	postive

BMPR1B	PART1	0.69937566	2.36E-80	postive
ELOVL4	PART1	0.67506273	5.98E-73	postive
RDH11	PART1	0.66581534	2.57E-70	postive
GABARAPL1	PART1	0.648208	1.48E-65	postive
ACACB	PART1	0.52719617	6.92E-40	postive
CPT1B	MIR3936HG	0.54035068	3.46E-42	postive
D2HGDH	MIR3936HG	0.60933535	4.31E-56	postive
ALOX12	MIR3936HG	0.6573749	5.39E-68	postive
CPT1B	AC109460.3	0.6719835	4.62E-72	postive
D2HGDH	AC109460.3	0.66648232	1.67E-70	postive
ALOX12	AC109460.3	0.59603651	3.80E-53	postive
ACOT6	AL355102.1	0.77814129	1.52E-110	postive
BMPR1B	AL355102.1	0.67012873	1.56E-71	postive
ACOT6	AC106037.3	0.9099577	2.00E-207	postive
BMPR1B	AC106037.3	0.78739793	6.51E-115	postive
D2HGDH	AC093915.1	0.59511933	6.00E-53	postive
ALOX12	AC093915.1	0.51990744	1.18E-38	postive
ELOVL2	AC135050.1	0.82396689	1.40E-134	postive
ACSBG2	AC135050.1	0.68436884	1.07E-75	postive
CYP4B1	AC135050.1	0.8766575	9.15E-173	postive
FASN	AC135050.1	0.83341943	2.03E-140	postive
ACOT6	AL158207.2	0.64290306	3.49E-64	postive

CPT1B	AC092301.1	0.56237226	2.84E-46	postive
ALOX12	AC092301.1	0.62002219	1.46E-58	postive
ACOT6	ACAP2-IT1	0.63660764	1.37E-62	postive
ALOX12	AC079684.1	0.50400175	4.57E-36	postive
ACSBG2	AC079684.1	0.6175427	5.57E-58	postive
PRKAA2	AP001486.2	0.61149103	1.39E-56	postive
CBR4	AP001486.2	0.50601884	2.18E-36	postive
MMAA	AP001486.2	0.55854622	1.53E-45	postive
ALOX12	AC105105.3	0.51595563	5.34E-38	postive
ALOX12	AL117336.2	0.5199014	1.18E-38	postive
ACACB	UCKL1-AS1	0.61179185	1.19E-56	postive
ALOX12	AC011461.1	0.58083568	6.06E-50	postive
ACSBG2	AC011461.1	0.52899087	3.41E-40	postive
ALOX12	AC004381.1	0.5558586	4.94E-45	postive
ELOVL2	REV3L-IT1	0.82053882	1.50E-132	postive
ACSBG2	REV3L-IT1	0.68503268	6.74E-76	postive
CYP4B1	REV3L-IT1	0.88591918	2.68E-181	postive
FASN	REV3L-IT1	0.81194734	1.21E-127	postive
CPT1B	AL139099.2	0.51575545	5.77E-38	postive
ALOX12	AL139099.2	0.68267912	3.43E-75	postive
ALOX12	AC105339.3	0.61759037	5.43E-58	postive
ACSBG2	AC105339.3	0.55975472	9.01E-46	postive

SDHD	AP000894.4	0.51986732	1.20E-38	postive
AUH	AP000894.4	0.59054239	5.71E-52	postive
ALOX12	AC009336.1	0.50765108	1.20E-36	postive
ACOT6	ATP1B3-AS1	0.67412321	1.12E-72	postive
ALOX12	AL353801.2	0.52131746	6.86E-39	postive
ELOVL2	AC007619.1	0.69287404	2.66E-78	postive
ACSBG2	AC007619.1	0.68015795	1.93E-74	postive
CYP4B1	AC007619.1	0.74464529	2.40E-96	postive
FASN	AC007619.1	0.66823628	5.36E-71	postive
ALOX12	AL049840.3	0.52824807	4.57E-40	postive
BMPR1B	LINC01213	0.63292852	1.13E-61	postive
ELOVL4	LINC01213	0.56051152	6.46E-46	postive
RDH11	LINC01213	0.64514562	9.23E-65	postive
GABARAPL1	LINC01213	0.50298901	6.60E-36	postive
PRKAG2	AC010501.2	0.58571098	5.94E-51	postive
SDHD	AC010501.2	0.5901319	6.98E-52	postive
PHYH	AC010501.2	0.58872164	1.39E-51	postive
PDHB	AC010501.2	0.54194865	1.79E-42	postive
GCDH	AC010501.2	0.51946393	1.40E-38	postive
HACD3	AC010501.2	0.63227432	1.63E-61	postive
AUH	AC010501.2	0.60826411	7.53E-56	postive

HACL1	AC010501.2	0.61965904	1.78E-58	postive
BMPR1B	AC010501.2	0.70640467	1.23E-82	postive
ELOVL4	AC010501.2	0.70343245	1.16E-81	postive
RDH11	AC010501.2	0.72327718	2.12E-88	postive
GABARAPL1	AC010501.2	0.77217347	7.75E-108	postive
ACACB	AC010501.2	0.61363758	4.48E-57	postive
HACD3	RALGPS2-AS1	0.508892	7.56E-37	postive
BMPR1B	RALGPS2-AS1	0.52993934	2.34E-40	postive
ELOVL2	AC106028.3	0.53435514	3.99E-41	postive
ACSBG2	AC106028.3	0.61737476	6.10E-58	postive
CYP4B1	AC106028.3	0.56417154	1.27E-46	postive
FASN	AC106028.3	0.53374954	5.09E-41	postive
ACOT6	AC092828.1	0.5110389	3.40E-37	postive
ACSBG2	AL136115.2	0.5232703	3.22E-39	postive
ACOT6	AL136115.2	0.60990405	3.20E-56	postive
ALOX12	AL512656.1	0.52240936	4.50E-39	postive
CPT1B	AC011472.1	0.50767042	1.19E-36	postive
D2HGDH	AC011472.1	0.6028008	1.26E-54	postive
ALOX12	AC011472.1	0.58996732	7.57E-52	postive
ACOT6	AP000787.1	0.57901868	1.43E-49	postive
BMPR1B	AP000787.1	0.66803654	6.11E-71	postive
ACACB	AP000787.1	0.56094336	5.34E-46	postive

HSD17B3	AC012645.4	0.56234773	2.87E-46	postive
CPT1B	AC012645.4	0.57182462	4.01E-48	postive
D2HGDH	AC012645.4	0.56427342	1.22E-46	postive
ALOX12	AC012645.4	0.53976727	4.40E-42	postive
HSD17B3	AL354836.1	0.53121624	1.41E-40	postive
CPT1B	AL354836.1	0.67569535	3.92E-73	postive
D2HGDH	AL354836.1	0.60182143	2.07E-54	postive
ACADVL	AL354836.1	0.50537418	2.76E-36	postive
CYP4B1	AC002451.1	0.5112111	3.19E-37	postive
ALOX12	AP002340.1	0.53409043	4.44E-41	postive
ELOVL2	LINC00862	0.6243452	1.37E-59	postive
CYP4B1	LINC00862	0.58413346	1.26E-50	postive
FASN	LINC00862	0.5968482	2.54E-53	postive
ACAA2	AL161782.1	0.50264025	7.50E-36	postive
PRKAA2	AL161782.1	0.60663577	1.75E-55	postive
ALDH3A2	AL161782.1	0.51917565	1.56E-38	postive
HIBCH	AL161782.1	0.58294527	2.23E-50	postive
SLC27A2	AL161782.1	0.52629056	9.89E-40	postive
ELOVL2	FAM215A	0.79300552	1.14E-117	postive
ACSBG2	FAM215A	0.65605829	1.22E-67	postive
CYP4B1	FAM215A	0.85763345	3.23E-157	postive
FASN	FAM215A	0.80159688	4.66E-122	postive

ELOVL2	AP001029.1	0.50622507	2.02E-36	postive
HSD17B3	AP001029.1	0.51884711	1.77E-38	postive
ALOX12	AP001029.1	0.50997273	5.06E-37	postive
ACSBG2	AP001029.1	0.58462173	1.00E-50	postive
CYP4B1	AP001029.1	0.5343785	3.95E-41	postive
ALOX12	COX10-AS1	0.55013831	5.76E-44	postive
ACSBG2	COX10-AS1	0.52002887	1.13E-38	postive
ELOVL2	AC090970.1	0.82699297	2.07E-136	postive
ACSBG2	AC090970.1	0.67444385	9.04E-73	postive
CYP4B1	AC090970.1	0.89030046	1.35E-185	postive
FASN	AC090970.1	0.81974268	4.39E-132	postive
HSD17B3	H1-10-AS1	0.53583951	2.19E-41	postive
CPT1B	H1-10-AS1	0.51973887	1.26E-38	postive
D2HGDH	H1-10-AS1	0.59710226	2.23E-53	postive
SLC27A3	H1-10-AS1	0.52289822	3.72E-39	postive
ACACB	AP000866.1	0.50554758	2.59E-36	postive
ELOVL2	LINC01593	0.53823215	8.26E-42	postive
ACSBG2	LINC01593	0.52175173	5.80E-39	postive
CYP4B1	LINC01593	0.61204549	1.04E-56	postive
FASN	LINC01593	0.54236619	1.51E-42	postive
SERINC1	LINC01637	-0.5119014	2.46E-37	negative

ALOX12	LAMC1-AS1	0.57390195	1.54E-48	postive
ACOT6	CACNA1C-AS4	0.55665954	3.49E-45	postive
ALOX12	AC004918.3	0.5556753	5.35E-45	postive
ACSBG2	FGF14-IT1	0.57372133	1.68E-48	postive
ELOVL2	AC005911.1	0.73630358	3.76E-93	postive
ACSBG2	AC005911.1	0.65366776	5.34E-67	postive
CYP4B1	AC005911.1	0.79105608	1.06E-116	postive
FASN	AC005911.1	0.73930443	2.75E-94	postive
ELOVL2	AP000944.1	0.82990682	3.30E-138	postive
ACSBG2	AP000944.1	0.64955479	6.55E-66	postive
CYP4B1	AP000944.1	0.89457286	5.75E-190	postive
FASN	AP000944.1	0.82669895	3.12E-136	postive
CPT1B	AC092910.3	0.50073931	1.49E-35	postive
ALOX12	AC092910.3	0.62673278	3.67E-60	postive
CBR4	AC092910.3	0.52019155	1.06E-38	postive
ALOX12	AP001458.1	0.57437668	1.24E-48	postive
ALOX12	AL035587.1	0.5166811	4.06E-38	postive
ELOVL2	AL109741.1	0.71105834	3.49E-84	postive
CYP4B1	AL109741.1	0.79303221	1.11E-117	postive
FASN	AL109741.1	0.637066	1.05E-62	postive
ACSBG2	AC055811.3	0.55344075	1.40E-44	postive
ACOT6	AC055811.3	0.54751174	1.75E-43	postive

ELOVL2	AC006160.1	0.76202537	2.03E-103	postive
ACSBG2	AC006160.1	0.71707125	3.14E-86	postive
CYP4B1	AC006160.1	0.81815472	3.67E-131	postive
FASN	AC006160.1	0.74723012	2.32E-97	postive
ALOX12	AL020997.3	0.52643951	9.32E-40	postive
SDHD	AC105094.2	0.50905625	7.11E-37	postive
ACOT6	AC105094.2	0.69523952	4.84E-79	postive
BMPR1B	AC105094.2	0.72621621	1.90E-89	postive
ELOVL4	AC105094.2	0.68086743	1.19E-74	postive
ELOVL2	AC079322.1	0.76346405	4.95E-104	postive
ACSBG2	AC079322.1	0.70997136	8.08E-84	postive
CYP4B1	AC079322.1	0.83760222	4.03E-143	postive
FASN	AC079322.1	0.75430807	3.32E-100	postive
ACOT6	AC108693.2	0.65701704	6.73E-68	postive
BMPR1B	AC108693.2	0.75082604	8.56E-99	postive
ACACB	AC108693.2	0.50676576	1.66E-36	postive
ELOVL2	AP002993.1	0.81257114	5.44E-128	postive
ACSBG2	AP002993.1	0.6892948	3.40E-77	postive
CYP4B1	AP002993.1	0.86773189	3.60E-165	postive
FASN	AP002993.1	0.79401775	3.57E-118	postive
ELOVL2	ABCC5-AS1	0.80697862	6.40E-125	postive

ACSBG2	ABCC5-AS1	0.65406721	4.18E-67	postive
CYP4B1	ABCC5-AS1	0.87202341	9.46E-169	postive
FASN	ABCC5-AS1	0.800104	2.80E-121	postive
CYP4B1	AL109976.1	0.51348659	1.36E-37	postive
RDH11	AL109976.1	0.56853803	1.79E-47	postive
D2HGDH	GEMIN7-AS1	0.5099489	5.11E-37	postive
ALOX12	GEMIN7-AS1	0.69265355	3.12E-78	postive
ACSBG2	GEMIN7-AS1	0.63133834	2.77E-61	postive
ALOX12	SLFNL1-AS1	0.58872688	1.38E-51	postive
ELOVL2	KLHL7-DT	0.51016439	4.71E-37	postive
ELOVL2	AL023881.1	0.70209697	3.15E-81	postive
ACSBG2	AL023881.1	0.60167281	2.23E-54	postive
CYP4B1	AL023881.1	0.77415061	1.00E-108	postive
FASN	AL023881.1	0.6819176	5.79E-75	postive
ELOVL2	AC005532.1	0.5170469	3.53E-38	postive
RDH11	AP006287.2	0.51326267	1.48E-37	postive
ELOVL2	AC079336.5	0.75367016	6.04E-100	postive
ACSBG2	AC079336.5	0.75190136	3.15E-99	postive
CYP4B1	AC079336.5	0.7918325	4.38E-117	postive
FASN	AC079336.5	0.7330359	6.21E-92	postive
SERINC1	AC008555.1	0.54751363	1.75E-43	postive
HSD17B3	AC008105.1	0.55422001	1.00E-44	postive
CPT1B	AC008105.1	0.52262486	4.14E-39	postive

ELOVL2	AC005363.2	0.75885754	4.37E-102	postive
ACSBG2	AC005363.2	0.662272	2.47E-69	postive
CYP4B1	AC005363.2	0.80312071	7.36E-123	postive
FASN	AC005363.2	0.75527234	1.34E-100	postive
ACOT6	AC036214.2	0.70343715	1.16E-81	postive
BMPR1B	AC036214.2	0.61480608	2.41E-57	postive
D2HGDH	AL096870.2	0.54403906	7.52E-43	postive
ALOX12	AL096870.2	0.62615935	5.04E-60	postive
ELOVL2	SIRLNT	0.56360233	1.64E-46	postive
CYP4B1	SIRLNT	0.51960409	1.33E-38	postive
FASN	SIRLNT	0.50519681	2.95E-36	postive
CPT1B	CACTIN-AS1	0.52761988	5.86E-40	postive
D2HGDH	CACTIN-AS1	0.54009316	3.85E-42	postive
ALOX12	CACTIN-AS1	0.52610884	1.06E-39	postive
ALOX12	AC097641.2	0.58332496	1.86E-50	postive
ELOVL2	AL158825.2	0.61785783	4.70E-58	postive
ACSBG2	AL158825.2	0.62414097	1.54E-59	postive
CYP4B1	AL158825.2	0.67024233	1.45E-71	postive
ACOT6	AL158825.2	0.53574741	2.27E-41	postive
FASN	AL158825.2	0.59768187	1.67E-53	postive
ACSBG2	AL359232.1	0.50965784	5.69E-37	postive

ALOX12	AC027097.2	0.57955544	1.11E-49	postive
ACSBG2	AC027097.2	0.5215615	6.24E-39	postive
ACOT6	AC008937.3	0.52369045	2.73E-39	postive
ACOT6	ARAP1-AS2	0.76949786	1.19E-106	postive
BMPR1B	ARAP1-AS2	0.60540139	3.31E-55	postive
CYP4B1	LINC01506	0.54367639	8.74E-43	postive
ACSBG1	ITPR1-DT	0.5015889	1.10E-35	postive
ACOT6	AC012435.3	0.62079031	9.62E-59	postive
ALOX12	AL135960.1	0.51921077	1.54E-38	postive
ALOX12	AP001636.3	0.52145531	6.50E-39	postive
ELOVL2	AC092919.1	0.66341956	1.19E-69	postive
ACSBG2	AC092919.1	0.53056718	1.82E-40	postive
CYP4B1	AC092919.1	0.68608449	3.24E-76	postive
FASN	AC092919.1	0.66622734	1.97E-70	postive
ACOT6	AC108159.1	0.91614429	2.37E-215	postive
BMPR1B	AC108159.1	0.705572	2.32E-82	postive
PRKAG2	LINC00706	0.54441035	6.44E-43	postive
ACOT8	LINC00706	0.59130883	3.93E-52	postive
ELOVL2	NDUFB2-AS1	0.77704772	4.84E-110	postive
ACSBG2	NDUFB2-AS1	0.65193662	1.54E-66	postive
CYP4B1	NDUFB2-AS1	0.83460224	3.56E-141	postive
FASN	NDUFB2-AS1	0.81837553	2.73E-131	postive

ELOVL2	AP002812.5	0.64788212	1.80E-65	postive
ACSBG2	AP002812.5	0.73157112	2.16E-91	postive
CYP4B1	AP002812.5	0.68740557	1.29E-76	postive
FASN	AP002812.5	0.60654943	1.83E-55	postive
ALDH3A2	LINC01671	0.5179586	2.49E-38	postive
ELOVL2	AC084026.2	0.82280122	6.94E-134	postive
ACSBG2	AC084026.2	0.64302245	3.25E-64	postive
CYP4B1	AC084026.2	0.86559319	1.97E-163	postive
FASN	AC084026.2	0.80856146	8.86E-126	postive
ELOVL2	AC130324.1	0.76932854	1.41E-106	postive
ACSBG2	AC130324.1	0.7353739	8.38E-93	postive
CYP4B1	AC130324.1	0.82026705	2.17E-132	postive
FASN	AC130324.1	0.73839088	6.13E-94	postive
HSD17B3	AC107081.1	0.54800423	1.42E-43	postive
ALOX12	AC107081.1	0.52988235	2.39E-40	postive
ACSBG2	AC107081.1	0.63612896	1.80E-62	postive
CYP4B1	AC107081.1	0.50638184	1.91E-36	postive
ELOVL2	FGF12-AS2	0.76999666	7.18E-107	postive
ACSBG2	FGF12-AS2	0.54922772	8.48E-44	postive
CYP4B1	FGF12-AS2	0.81355931	1.52E-128	postive
FASN	FGF12-AS2	0.74789705	1.26E-97	postive

ELOVL2	AC007993.2	0.75993833	1.54E-102	postive
ACSBG2	AC007993.2	0.61448501	2.86E-57	postive
CYP4B1	AC007993.2	0.83321262	2.75E-140	postive
FASN	AC007993.2	0.77590687	1.61E-109	postive
ALOX12	AL356481.1	0.50233377	8.38E-36	postive
ELOVL2	AC008667.1	0.70487048	3.94E-82	postive
ACSBG2	AC008667.1	0.65125418	2.34E-66	postive
CYP4B1	AC008667.1	0.73585709	5.53E-93	postive
FASN	AC008667.1	0.67160602	5.92E-72	postive
ELOVL2	AL022316.1	0.64897166	9.32E-66	postive
ACSBG2	AL022316.1	0.62910045	9.78E-61	postive
CYP4B1	AL022316.1	0.68529699	5.61E-76	postive
FASN	AL022316.1	0.65999911	1.04E-68	postive
CPT1B	THBS3-AS1	0.59656618	2.92E-53	postive
D2HGDH	THBS3-AS1	0.56432795	1.19E-46	postive
ALOX12	THBS3-AS1	0.5911759	4.19E-52	postive
ELOVL2	AP005203.1	0.83233805	9.86E-140	postive
ACSBG2	AP005203.1	0.64152015	7.87E-64	postive
CYP4B1	AP005203.1	0.89572088	3.57E-191	postive
FASN	AP005203.1	0.82926427	8.28E-138	postive
HSD17B3	AC068620.2	0.53981916	4.31E-42	postive

CPT1B	AC068620.2	0.58990079	7.82E-52	postive
ACOT6	GSEC	0.58618662	4.72E-51	postive
BMPR1B	GSEC	0.58247153	2.79E-50	postive
D2HGDH	BCRP3	0.57013382	8.67E-48	postive
GABARAPL1	AL031726.1	0.54192504	1.81E-42	postive
ELOVL2	AC015849.4	0.7267386	1.23E-89	postive
ACSBG2	AC015849.4	0.70138347	5.35E-81	postive
CYP4B1	AC015849.4	0.7930593	1.08E-117	postive
FASN	AC015849.4	0.71381327	4.09E-85	postive
ACOT6	AC098869.2	0.72001475	2.98E-87	postive
BMPR1B	AC098869.2	0.51247366	1.99E-37	postive
ELOVL2	HLX-AS1	0.58417438	1.24E-50	postive
CYP4B1	HLX-AS1	0.59256111	2.12E-52	postive
FASN	HLX-AS1	0.55417256	1.02E-44	postive
ELOVL2	AP003555.1	0.84040685	5.63E-145	postive
ACSBG2	AP003555.1	0.63458576	4.37E-62	postive
CYP4B1	AP003555.1	0.87639349	1.56E-172	postive
FASN	AP003555.1	0.82034788	1.94E-132	postive
CPT1B	AC010327.6	0.56928885	1.27E-47	postive
D2HGDH	AC010327.6	0.55855122	1.53E-45	postive
ALOX12	AC010327.6	0.5065484	1.80E-36	postive
ELOVL2	AC087501.4	0.60698287	1.46E-55	postive

ACSBG2	AC087501.4	0.59720372	2.12E-53	postive
CYP4B1	AC087501.4	0.67705879	1.57E-73	postive
FASN	AC087501.4	0.58463114	9.97E-51	postive
ELOVL2	AC016027.2	0.6575121	4.94E-68	postive
ACSBG2	AC016027.2	0.69142144	7.52E-78	postive
CYP4B1	AC016027.2	0.70840137	2.70E-83	postive
FASN	AC016027.2	0.63042849	4.63E-61	postive
CPT1B	LINC00893	0.67274415	2.79E-72	postive
D2HGDH	LINC00893	0.6291782	9.36E-61	postive
ALOX12	LINC00893	0.63297028	1.10E-61	postive
ELOVL2	AC010463.3	0.58453831	1.04E-50	postive
ALOX12	AC010463.3	0.53838386	7.76E-42	postive
ACSBG2	AC010463.3	0.66896796	3.33E-71	postive
CYP4B1	AC010463.3	0.61819965	3.91E-58	postive
FASN	AC010463.3	0.58028296	7.86E-50	postive
ACSBG1	LINC01484	0.50351555	5.45E-36	postive
ENO3	LINC01484	0.64401384	1.81E-64	postive
PCBD1	LINC01484	0.53091919	1.58E-40	postive
PHYH	AC013472.1	0.50831059	9.38E-37	postive
HACL1	AC013472.1	0.57121445	5.30E-48	postive
BMPR1B	AC013472.1	0.67137289	6.90E-72	postive

ELOVL4	AC013472.1	0.66132561	4.51E-69	postive
RDH11	AC013472.1	0.75485345	1.99E-100	postive
ACACB	AC013472.1	0.50912393	6.94E-37	postive
ALOX12	AL158196.1	0.53592471	2.11E-41	postive
ELOVL2	PCOLCE-AS1	0.5527346	1.90E-44	postive
ACSBG2	PCOLCE-AS1	0.50306748	6.42E-36	postive
CYP4B1	PCOLCE-AS1	0.58108413	5.39E-50	postive
FASN	PCOLCE-AS1	0.54251697	1.42E-42	postive
ELOVL2	AC022960.1	0.8239954	1.34E-134	postive
ACSBG2	AC022960.1	0.68094268	1.13E-74	postive
CYP4B1	AC022960.1	0.88710132	1.93E-182	postive
FASN	AC022960.1	0.81439177	5.17E-129	postive
CPT1B	AL021707.8	0.59741684	1.91E-53	postive
ALOX12	AL021707.8	0.5532865	1.50E-44	postive
ACOT6	TEX41	0.62618532	4.97E-60	postive
ELOVL2	USP2-AS1	0.64266676	4.01E-64	postive
ACSBG2	USP2-AS1	0.55103352	3.94E-44	postive
CYP4B1	USP2-AS1	0.68299896	2.75E-75	postive
FASN	USP2-AS1	0.6622764	2.47E-69	postive
BMPR1B	GPRC5D-AS1	0.5405937	3.13E-42	postive
ACOT6	AC004466.3	0.54913646	8.82E-44	postive
ALOX12	LINC01534	0.6955674	3.82E-79	postive
CPT1B	AC023908.3	0.61426269	3.22E-57	postive

D2HGDH	AC023908.3	0.53954665	4.82E-42	postive
ALOX12	AC023908.3	0.63939821	2.72E-63	postive
ELOVL2	AL121890.2	0.83530404	1.26E-141	postive
ACSBG2	AL121890.2	0.69064933	1.30E-77	postive
CYP4B1	AL121890.2	0.88710119	1.93E-182	postive
FASN	AL121890.2	0.81954231	5.75E-132	postive
ACOT6	MFF-DT	0.74250717	1.63E-95	postive
BMPR1B	MFF-DT	0.55639355	3.91E-45	postive
ACOT6	TET2-AS1	0.64135888	8.65E-64	postive
ACOT8	SPINT1-AS1	0.54091848	2.74E-42	postive
SDHD	SPINT1-AS1	0.65049765	3.70E-66	postive
PHYH	SPINT1-AS1	0.69854705	4.35E-80	postive
NDUFAB1	SPINT1-AS1	0.70768208	4.67E-83	postive
HACD3	SPINT1-AS1	0.63794961	6.31E-63	postive
ELOVL4	SPINT1-AS1	0.71667045	4.31E-86	postive
ELOVL2	AP001381.1	0.70254943	2.25E-81	postive
ACSBG2	AP001381.1	0.67304847	2.28E-72	postive
CYP4B1	AP001381.1	0.75132685	5.38E-99	postive
FASN	AP001381.1	0.67434206	9.67E-73	postive
ELOVL2	AL512791.2	0.79962742	4.94E-121	postive

ACSBG2	AL512791.2	0.62984878	6.42E-61	postive
CYP4B1	AL512791.2	0.85192048	5.50E-153	postive
FASN	AL512791.2	0.78121056	5.72E-112	postive
ELOVL2	AL139289.1	0.63559164	2.46E-62	postive
ACSBG2	AL139289.1	0.5500692	5.93E-44	postive
CYP4B1	AL139289.1	0.68989802	2.22E-77	postive
FASN	AL139289.1	0.65136713	2.18E-66	postive
HSD17B3	AC011468.1	0.56569647	6.44E-47	postive
HSD17B3	ZNF436-AS1	0.56102086	5.16E-46	postive
ALOX12	ZNF337-AS1	0.55454512	8.72E-45	postive
ELOVL2	AL031722.1	0.70111313	6.54E-81	postive
ACSBG2	AL031722.1	0.66560662	2.94E-70	postive
CYP4B1	AL031722.1	0.78233876	1.69E-112	postive
FASN	AL031722.1	0.67119438	7.76E-72	postive
ALOX12	AC048382.2	0.52684957	7.94E-40	postive
ACOT6	AC048382.2	0.63946077	2.62E-63	postive
HSD17B3	AC004148.1	0.5599824	8.15E-46	postive
CPT1B	AC004148.1	0.67990438	2.29E-74	postive
D2HGDH	AC004148.1	0.63407214	5.87E-62	postive
ACADVL	AC004148.1	0.50820564	9.75E-37	postive
ALOX12	AC004148.1	0.69414043	1.07E-78	postive

ACOT6	AC253576.2	0.53433048	4.03E-41	postive
ACBD5	OIP5-AS1	0.56880249	1.59E-47	postive
PRKAA2	OIP5-AS1	0.69034509	1.62E-77	postive
CBR4	OIP5-AS1	0.61588706	1.35E-57	postive
MMAA	OIP5-AS1	0.57727477	3.22E-49	postive
CPT1B	AL360181.2	0.57001954	9.13E-48	postive
D2HGDH	AL360181.2	0.64575003	6.44E-65	postive
ACADVL	AL360181.2	0.50091191	1.40E-35	postive
ALOX12	AL360181.2	0.652817	9.00E-67	postive
ALOX12	AC004223.4	0.52241787	4.48E-39	postive
CPT1B	AC090527.3	0.51403601	1.10E-37	postive
D2HGDH	AC090527.3	0.51224861	2.16E-37	postive
ALOX12	AC090527.3	0.65985483	1.14E-68	postive
ACOT6	AC004461.2	0.593304	1.47E-52	postive
CPT1B	LINC01004	0.60090828	3.29E-54	postive
ACOT6	LINC01004	0.56027589	7.16E-46	postive
ELOVL2	AL592071.1	0.7883634	2.21E-115	postive
ACSBG2	AL592071.1	0.61304108	6.15E-57	postive
CYP4B1	AL592071.1	0.84039711	5.72E-145	postive
FASN	AL592071.1	0.81529105	1.60E-129	postive
ELOVL2	AC025176.1	0.80675484	8.46E-125	postive
ACSBG2	AC025176.1	0.68131567	8.74E-75	postive

CYP4B1	AC025176.1	0.84727427	1.13E-149	postive
FASN	AC025176.1	0.81048049	7.86E-127	postive
HSD17B3	LENG8-AS1	0.53597505	2.07E-41	postive
CPT1B	LENG8-AS1	0.6620067	2.93E-69	postive
D2HGDH	LENG8-AS1	0.68135663	8.50E-75	postive
ALOX12	LENG8-AS1	0.70079893	8.26E-81	postive
ELOVL2	BMPR1B-DT	0.73894419	3.78E-94	postive
ACSBG2	BMPR1B-DT	0.55447681	8.98E-45	postive
CYP4B1	BMPR1B-DT	0.7633695	5.43E-104	postive
FASN	BMPR1B-DT	0.74123286	5.04E-95	postive
ELOVL2	AC005332.1	0.81991365	3.49E-132	postive
ACSBG2	AC005332.1	0.68678499	1.99E-76	postive
CYP4B1	AC005332.1	0.87869927	1.38E-174	postive
FASN	AC005332.1	0.80958899	2.43E-126	postive
ALOX12	LINC02614	0.6290347	1.01E-60	postive
ELOVL2	AC010336.2	0.60254003	1.43E-54	postive
ACSBG2	AC010336.2	0.59768582	1.67E-53	postive
CYP4B1	AC010336.2	0.70998339	8.01E-84	postive
FASN	AC010336.2	0.62075343	9.82E-59	postive
ELOVL2	AC012485.2	0.68441296	1.04E-75	postive
ACSBG2	AC012485.2	0.63876094	3.94E-63	postive
CYP4B1	AC012485.2	0.76912302	1.74E-106	postive

FASN	AC012485.2	0.68027209	1.78E-74	postive
ELOVL2	LINC01117	0.54532965	4.38E-43	postive
CYP4B1	LINC01117	0.57044963	7.51E-48	postive
ACSBG1	AC024270.1	0.68137839	8.38E-75	postive
ENO3	AC024270.1	0.89547911	6.43E-191	postive
PCBD1	AC024270.1	0.60151825	2.41E-54	postive
ELOVL2	AL732314.4	0.80316601	6.97E-123	postive
ACSBG2	AL732314.4	0.71670744	4.19E-86	postive
CYP4B1	AL732314.4	0.87685772	6.08E-173	postive
FASN	AL732314.4	0.78950088	6.17E-116	postive
ALOX12	AC006272.1	0.51258577	1.91E-37	postive
ALOX12	AC019205.1	0.56199655	3.35E-46	postive
ACSBG2	AC019205.1	0.52718159	6.96E-40	postive
ACACB	AL121906.2	0.50471693	3.52E-36	postive
HSD17B3	HOXB-AS1	0.54300544	1.16E-42	postive
ALOX12	HOXB-AS1	0.53007542	2.21E-40	postive
ACOT6	AC131971.1	0.71532388	1.25E-85	postive
ELOVL2	LINC00337	0.83585179	5.57E-142	postive
ACSBG2	LINC00337	0.64876699	1.05E-65	postive
CYP4B1	LINC00337	0.88687143	3.23E-182	postive

FASN	LINC00337	0.84344374	5.02E-147	postive
HSD17B3	LINC00174	0.55843718	1.61E-45	postive
CPT1B	LINC00174	0.70832928	2.85E-83	postive
D2HGDH	LINC00174	0.6863208	2.75E-76	postive
ALOX12	LINC00174	0.63238097	1.54E-61	postive
CPT1B	AC022150.2	0.59661988	2.84E-53	postive
D2HGDH	AC022150.2	0.60875049	5.85E-56	postive
ALOX12	AC022150.2	0.61753649	5.59E-58	postive
HSD17B3	LINC01176	0.63917209	3.10E-63	postive
CPT1B	LINC01176	0.58503095	8.23E-51	postive
D2HGDH	LINC01176	0.64149705	7.97E-64	postive
ACADVL	LINC01176	0.59014562	6.94E-52	postive
ALOX12	LINC01176	0.5366527	1.57E-41	postive
ELOVL2	RBM38-AS1	0.79435291	2.42E-118	postive
ACSBG2	RBM38-AS1	0.67181903	5.14E-72	postive
CYP4B1	RBM38-AS1	0.8357187	6.79E-142	postive
FASN	RBM38-AS1	0.79482	1.41E-118	postive
ACOT6	AC084048.1	0.91313635	2.01E-211	postive
BMPR1B	AC084048.1	0.74641715	4.85E-97	postive
HSD17B3	AC108673.3	0.57059393	7.03E-48	postive
SERINC1	AC108673.3	-0.5308281	1.64E-40	negative
ELOVL2	SKAP1-AS1	0.76171037	2.76E-103	postive

ACSBG2	SKAP1-AS1	0.70392189	8.04E-82	postive
CYP4B1	SKAP1-AS1	0.81979967	4.07E-132	postive
FASN	SKAP1-AS1	0.7445371	2.65E-96	postive
ACOT6	AC010300.1	0.79625058	2.66E-119	postive
BMPR1B	AC010300.1	0.58568773	6.00E-51	postive
ACOT6	RERG-AS1	0.75873511	4.92E-102	postive
BMPR1B	RERG-AS1	0.60416362	6.26E-55	postive
ELOVL2	AL139260.1	0.54052167	3.23E-42	postive
CYP4B1	AL139260.1	0.57061681	6.96E-48	postive
FASN	AL139260.1	0.55650809	3.72E-45	postive
HSD17B3	AC010883.1	0.65015668	4.55E-66	postive
CPT1B	AC010883.1	0.56061259	6.18E-46	postive
D2HGDH	AC010883.1	0.66300606	1.55E-69	postive
ACADVL	AC010883.1	0.52780766	5.44E-40	postive
ALOX12	AC010883.1	0.51419288	1.04E-37	postive
ELOVL2	AC017006.2	0.8338743	1.04E-140	postive
ACSBG2	AC017006.2	0.66272949	1.85E-69	postive
CYP4B1	AC017006.2	0.89616468	1.21E-191	postive
FASN	AC017006.2	0.82935236	7.30E-138	postive
ELOVL2	GNA14-AS1	0.7776666	2.52E-110	postive
ACSBG2	GNA14-AS1	0.69513411	5.23E-79	postive
CYP4B1	GNA14-AS1	0.83565637	7.45E-142	postive

FASN	GNA14-AS1	0.75479153	2.10E-100	postive
HSD17B3	AC027271.1	0.57469745	1.07E-48	postive
ACOT6	AC106037.2	0.69989312	1.61E-80	postive
BMPR1B	AC106037.2	0.78704489	9.64E-115	postive
RDH11	AC106037.2	0.51471687	8.54E-38	postive
ACACB	AC106037.2	0.54339131	9.84E-43	postive
D2HGDH	AC109460.1	0.53396133	4.67E-41	postive
ALOX12	AC109460.1	0.59370371	1.21E-52	postive
ACOT6	AC114947.2	0.57393826	1.52E-48	postive
ELOVL2	AC093752.1	0.78239296	1.59E-112	postive
ACSBG2	AC093752.1	0.68729842	1.39E-76	postive
CYP4B1	AC093752.1	0.85360289	3.26E-154	postive
FASN	AC093752.1	0.77788785	1.99E-110	postive
ACOT6	TSPAN9-IT1	0.65463401	2.95E-67	postive
ELOVL2	AC008747.1	0.81735121	1.07E-130	postive
ACSBG2	AC008747.1	0.70211696	3.10E-81	postive
CYP4B1	AC008747.1	0.86558271	2.01E-163	postive
FASN	AC008747.1	0.81534268	1.50E-129	postive
ACOT6	AP001439.1	0.52875368	3.74E-40	postive
ACOT6	AC011503.2	0.5050392	3.13E-36	postive
BMPR1B	AC011503.2	0.54480285	5.47E-43	postive

CPT1B	AL442128.2	0.55362731	1.29E-44	postive
ALOX12	AL442128.2	0.56438609	1.16E-46	postive
HSD17B3	AC008610.1	0.52213074	5.01E-39	postive
CPT1B	AC008610.1	0.53022359	2.09E-40	postive
CPT1B	AL158151.4	0.50811955	1.01E-36	postive
ACOT6	EP300-AS1	0.72123242	1.12E-87	postive
CPT1B	AC087500.2	0.50621096	2.03E-36	postive
D2HGDH	AC087500.2	0.57307341	2.26E-48	postive
ALOX12	AC087500.2	0.56463348	1.04E-46	postive
CBR4	UBR5-AS1	0.55282008	1.83E-44	postive
HSD17B3	AC093249.6	0.55528422	6.33E-45	postive
D2HGDH	AC093249.6	0.54228384	1.56E-42	postive
ACSBG2	AP002336.2	0.56245188	2.74E-46	postive
ACOT6	AP002336.2	0.69026339	1.71E-77	postive
ELOVL2	DAPK1-IT1	0.60088928	3.32E-54	postive
ACSBG2	DAPK1-IT1	0.63669972	1.30E-62	postive
CYP4B1	DAPK1-IT1	0.66586041	2.49E-70	postive
FASN	DAPK1-IT1	0.58881805	1.32E-51	postive
HSD17B3	SCAT2	0.72070025	1.72E-87	postive
CPT1B	SCAT2	0.50383605	4.85E-36	postive

ACOT6	AC073569.1	0.60699779	1.45E-55	postive
ELOVL2	AP000915.2	0.82184418	2.56E-133	postive
ACSBG2	AP000915.2	0.63719927	9.74E-63	postive
CYP4B1	AP000915.2	0.88791816	3.09E-183	postive
FASN	AP000915.2	0.81871406	1.74E-131	postive
ELOVL2	AC007216.2	0.66442006	6.29E-70	postive
ACSBG2	AC007216.2	0.65610911	1.18E-67	postive
CYP4B1	AC007216.2	0.71852501	9.85E-87	postive
FASN	AC007216.2	0.64494592	1.04E-64	postive
CPT1B	AL022328.2	0.71991342	3.24E-87	postive
D2HGDH	AL022328.2	0.76859954	2.96E-106	postive
ACADVL	AL022328.2	0.57097385	5.91E-48	postive
ALOX12	AL022328.2	0.68015066	1.94E-74	postive
ACSBG1	SDK1-AS1	0.537749	1.01E-41	postive
ENO3	SDK1-AS1	0.68721498	1.47E-76	postive
PCBD1	SDK1-AS1	0.52605357	1.09E-39	postive
ACOT6	AL133243.2	0.58406101	1.31E-50	postive
ALOX12	AF129075.1	0.58670753	3.68E-51	postive
ELOVL2	AC073311.1	0.70911068	1.57E-83	postive
ACSBG2	AC073311.1	0.58789403	2.07E-51	postive
CYP4B1	AC073311.1	0.781121	6.30E-112	postive

FASN	AC073311.1	0.7054202<br>5	2.60E-82	postive
