# Supplementary material for: Fatty acid metabolism-related lncRNAs are potential biomarkers for survival prediction in clear cell renal cell carcinoma
Source: Medicine (Baltimore). 2024 Feb 23;103(8):e37207. doi: 10.1097/MD.0000000000037207 (PMC11309608; doi:10.1097/MD.0000000000037207)
Supplement: Supplementary file 5 [file medi-103-e37207-s005.pdf]

lncRNA	conMean	treatMean	logFC	pValue	fdr
FGD5-AS1	45.09595	20.723099	-1.12175802	2.81E-37	1.01E-35
AC092296.2	0.06069	0.1904742	1.650056849	2.67E-18	1.00E-17
AC092794.2	0.026992	0.1412895	2.388044271	1.07E-10	1.99E-10
AC005746.1	0.053648	0.1482186	1.466141566	6.73E-08	1.00E-07
AC009119.1	0.103827	0.3436353	1.726696674	8.64E-28	7.92E-27
AC011899.2	0.041003	0.3170172	2.950773756	7.39E-32	9.90E-31
AC005776.2	0.069658	0.3305428	2.246485371	2.49E-20	1.09E-19
AP000866.6	0.174192	0.5227621	1.585480119	1.32E-10	2.44E-10
AL162586.1	0.582169	1.3607713	1.224915286	1.08E-09	1.84E-09
AC010463.2	0.027958	0.4600381	4.040439048	6.05E-21	2.80E-20
STPG3-AS1	0.204214	0.678807	1.732917494	1.09E-05	1.40E-05
AC008543.4	0.073127	0.1629353	1.155824206	9.90E-08	1.46E-07
CTBP1-AS	0.113337	0.4575036	2.013159016	2.28E-23	1.35E-22
AP001893.1	0.142671	0.3733874	1.38798407	1.55E-09	2.63E-09
AC007036.1	0.141953	0.3001297	1.080170247	0.004693524	0.005068
AP000907.2	0.099393	0.4069252	2.033551699	2.52E-13	5.86E-13
LINC02626	0.026686	0.1547098	2.535422149	1.11E-09	1.89E-09
AC008764.8	0.129768	0.5103445	1.975540138	3.45E-20	1.50E-19
LINCR-0001	0.137811	0.5049498	1.873448842	1.54E-15	4.36E-15

AC107294.1	0.067726	0.2058469	1.603787064	0.00080869	0.000925
LINC00885	0.74654	0.1579099	-2.24111893	1.68E-36	4.60E-35
AC004771.4	0.16658	0.3431032	1.042427709	7.45E-09	1.19E-08
MED8-AS1	0.164707	0.4651444	1.497776729	3.35E-14	8.44E-14
AP000345.2	0.320649	0.6791	1.082626958	0.014434704	0.014975
LINC00881	0.014348	0.2077196	3.855721507	5.09E-31	6.36E-30
MIR31HG	0.818213	0.3128173	-1.38715574	2.45E-17	8.26E-17
AC010618.2	0.094518	0.4333807	2.196980565	1.73E-15	4.87E-15
AC009133.1	0.809226	2.5178964	1.637605056	4.33E-32	5.90E-31
RCCD1-AS1	0.088005	0.2018848	1.197880186	4.05E-08	6.14E-08
CYTOR	1.491337	5.177978	1.795782597	3.00E-30	3.55E-29
AC100830.1	0.042349	0.1652222	1.963994944	2.76E-19	1.11E-18
COMETT	8.574225	2.0845403	-2.04027695	3.14E-09	5.17E-09
AC005519.1	0.195832	0.7354911	1.909089849	9.31E-14	2.24E-13
LINC02027	3.339308	1.4366993	-1.21679085	4.03E-07	5.68E-07
AC027796.5	0.033385	0.1117711	1.74325626	9.50E-07	1.31E-06
AC044840.1	0.034367	0.3643741	3.406318272	4.00E-13	9.13E-13
AC103769.1	0.048124	0.1989401	2.047505178	0.008720267	0.009215
ASTN2-AS1	0.006987	0.1134485	4.021296634	5.12E-06	6.73E-06
AL133215.1	0.073067	0.2222445	1.60484763	1.66E-09	2.80E-09

AC011479.2	0.052126	0.2685893	2.365324462	5.12E-18	1.86E-17
AC008555.2	0.03492	0.195704	2.486543275	1.19E-31	1.56E-30
AC078962.1	0.0139	0.17129	3.623295287	2.56E-09	4.27E-09
AL513218.1	0.142618	0.5959384	2.063012068	4.51E-15	1.22E-14
ETV7-AS1	0.129546	0.4555852	1.814252707	5.74E-19	2.26E-18
AC027682.4	0.057835	0.1727855	1.578976344	3.38E-10	6.02E-10
AC022431.1	0.650883	0.1096651	-2.56929456	5.75E-26	4.37E-25
AC027373.1	0.04278	0.2099992	2.29537566	6.25E-22	3.23E-21
AL035416.1	0.05295	0.141572	1.41883357	6.13E-10	1.07E-09
AC134407.1	0.029999	0.1470703	2.293530085	7.69E-12	1.57E-11
C22orf34	0.182434	0.6165393	1.756818501	3.48E-27	3.04E-26
MIR583HG	0.045814	0.161184	1.814862308	1.38E-11	2.75E-11
AC092802.2	0.098923	0.2730207	1.464635902	2.41E-13	5.62E-13
AC008764.6	0.158823	0.4793553	1.593677583	8.50E-20	3.57E-19
AC025811.1	0.417126	0.0873488	-2.25562405	4.16E-34	7.69E-33
LINC01252	0.097695	0.2513291	1.363220606	6.12E-08	9.14E-08
AC079793.1	0.013316	0.1762295	3.726179721	6.77E-19	2.65E-18
AC121247.1	0.049855	0.1651179	1.727671741	3.69E-13	8.46E-13
HIF1A-AS3	0.328371	6.1482154	4.226767539	1.10E-35	2.59E-34
MACORIS	0.23563	0.9332328	1.985714205	7.29E-29	7.58E-28

AC013549.1	0.033858	0.1811694	2.419780241	2.39E-06	3.21E-06
AC079035.1	0.020126	0.1244728	2.628708544	0.00042793	0.000499
AC087379.2	7.334647	1.378487	-2.41164194	6.60E-26	4.97E-25
AC007272.1	0.021619	0.1595462	2.88359071	2.05E-15	5.73E-15
AC009120.2	0.52105	1.7162326	1.719751613	1.15E-17	4.01E-17
AC026471.4	1.584315	3.7271877	1.234228365	4.03E-18	1.48E-17
AC025271.4	0.866757	0.1785207	-2.27953556	1.05E-35	2.52E-34
TRIM7-AS1	0.033313	0.1129836	1.761957891	5.70E-13	1.29E-12
AL157838.1	0.225541	0.5575609	1.305741177	2.69E-11	5.20E-11
AL035661.1	29.66428	2.5557825	-3.53688975	1.54E-36	4.48E-35
AC010525.1	0.065975	0.2766799	2.068222426	1.26E-08	1.97E-08
AL157871.2	0.095815	0.5338258	2.478048389	5.99E-16	1.73E-15
AL021707.4	0.165215	0.3736765	1.177444157	6.12E-08	9.14E-08
AC005740.3	0.062671	0.2849443	2.18481453	1.00E-14	2.64E-14
AC010422.4	0.154836	0.3549139	1.196724952	0.000481215	0.000559
Z99916.1	0.043563	0.1912638	2.134387319	1.68E-15	4.72E-15
C1RL-AS1	1.378692	4.2381054	1.620119367	1.84E-21	9.01E-21
SEMA3B-AS1	6.095147	0.8214482	-2.89141955	1.08E-36	3.38E-35
AC138956.1	0.266952	0.9036003	1.759106642	7.10E-17	2.27E-16
LINC00906	0.010717	0.1244301	3.537362568	3.86E-14	9.63E-14

MUC20-OT1	0.875011	2.6294906	1.587410824	5.48E-14	1.35E-13
AC005828.4	0.057749	0.1196825	1.051336634	0.004228625	0.004588
AC245884.10	0.03032	0.2392262	2.980038786	1.42E-16	4.39E-16
AC010519.1	0.056487	0.4130895	2.870467823	7.44E-24	4.58E-23
AC087741.1	0.311494	1.1678361	1.906561364	1.35E-13	3.21E-13
RNF207-AS1	1.477751	0.6101628	-1.27613696	4.73E-26	3.63E-25
AC024145.1	0.145718	0.5355623	1.877873108	1.25E-14	3.26E-14
LINC00551	1.44306	0.1258467	-3.51939144	1.62E-36	4.49E-35
AC018695.4	0.120265	0.4306016	1.840141841	4.80E-16	1.40E-15
AC130650.1	0.018639	0.1333689	2.839009566	4.23E-07	5.96E-07
AC084824.5	0.602468	1.4835019	1.30004933	8.09E-18	2.85E-17
RFPL3S	0.051009	0.1580456	1.631515239	4.36E-12	9.10E-12
AC092574.2	0.053175	0.1526589	1.521482034	0.01697178	0.017518
AC002563.1	0.19993	0.4429174	1.147542246	0.032405425	0.032823
AC090164.2	0.014077	0.2619955	4.218164449	5.84E-23	3.30E-22
AC104984.4	3.682574	0.4629255	-2.99186271	3.54E-38	1.49E-36
AC092375.2	0.072113	0.2796189	1.95512255	3.10E-14	7.82E-14
AC022558.1	0.122883	0.3631507	1.56327738	1.47E-12	3.18E-12
AL589739.1	0.094179	0.2447547	1.377853315	3.71E-05	4.60E-05
AL132639.2	0.9525	0.3688917	-1.36852156	1.36E-28	1.36E-27

SMAD9-IT1	0.124197	0.4092042	1.720195086	1.89E-10	3.45E-10
AC018557.2	0.028434	0.1461722	2.361960947	1.16E-06	1.60E-06
AL662797.1	0.164724	0.3876087	1.234548116	2.08E-20	9.22E-20
GUSBP11	0.133024	0.3121791	1.230691631	2.32E-10	4.20E-10
AC093690.1	0.061226	0.2616587	2.095466973	2.48E-21	1.20E-20
AC087071.2	0.507167	0.2406502	-1.07552324	9.85E-21	4.51E-20
BRWD1-AS2	1.017552	0.3313094	-1.61885184	4.43E-28	4.20E-27
AC004076.2	0.098978	0.2776805	1.488249227	4.48E-21	2.10E-20
LINC01976	1.911128	0.0595122	-5.00509435	6.44E-51	1.41E-48
AP001160.1	0.194225	0.6556059	1.755102056	4.58E-16	1.34E-15
AL731577.1	0.013662	0.1139011	3.059569606	0.001104829	0.001254
AP000346.1	0.090431	0.2074262	1.197700593	0.000269144	0.000318
AC011337.1	0.034082	0.1792948	2.395270662	1.29E-28	1.30E-27
AL121852.1	0.030914	0.1289348	2.06029587	1.05E-11	2.10E-11
LACTB2-AS1	0.044512	0.1869805	2.070616013	6.71E-29	7.05E-28
SEMA6A-AS1	0.144724	0.7403733	2.354945353	1.72E-27	1.53E-26
AC132872.3	0.365956	2.0192892	2.464106861	1.40E-21	6.94E-21
AC093484.4	0.161109	0.5577652	1.791620263	6.04E-13	1.36E-12
AC020661.3	0.026554	0.1228195	2.209551514	2.04E-05	2.57E-05
KDM4A-AS1	0.134701	0.3676344	1.448511866	1.92E-15	5.38E-15

CASC2	0.802866	0.3756325	-1.09583748	8.16E-34	1.45E-32
BMS1P4	0.072141	0.187184	1.375561713	3.46E-17	1.14E-16
AC090630.1	0.0523	0.1217311	1.218821531	2.42E-05	3.04E-05
AC116903.2	0.045263	0.108652	1.263306461	3.79E-05	4.70E-05
AC004817.3	0.008064	0.2230261	4.789660139	1.65E-29	1.82E-28
AP001029.2	0.017651	0.1201975	2.767597709	9.62E-26	7.09E-25
AC011443.1	0.027757	0.1689573	2.605721427	0.003178901	0.003474
DUSP5-DT	0.077911	0.2118772	1.44332463	4.38E-08	6.63E-08
AC114939.1	0.127972	0.4399291	1.781440857	6.48E-14	1.59E-13
AC087164.1	0.024305	0.1372253	2.497208812	4.77E-29	5.06E-28
AC026470.2	0.087653	0.2343489	1.418783257	0.000395277	0.000462
AC073842.2	0.198958	0.483188	1.280124256	3.13E-08	4.79E-08
AC010618.3	0.27074	0.6184897	1.191840638	4.93E-13	1.12E-12
MORF4L2-AS1	0.154218	0.312002	1.016585321	1.18E-05	1.51E-05
AP001429.1	0.076358	0.2703415	1.823927903	1.73E-06	2.35E-06
PDC-AS1	0.109025	0.2499271	1.196844422	1.38E-08	2.15E-08
AC116345.1	0.34711	1.9785326	2.51096651	5.36E-07	7.50E-07
CR936218.1	0.259281	1.0939316	2.076935709	9.57E-22	4.84E-21
AP001350.1	0.045162	0.1204962	1.41579707	1.44E-08	2.26E-08
AC015911.3	0.100497	0.8217785	3.031599665	7.92E-31	9.75E-30

AC087392.4	0.049665	0.148209	1.577320244	5.06E-11	9.62E-11
LINC01018	1.287663	0.2571716	-2.32395194	1.66E-32	2.50E-31
AC005253.1	0.241345	0.8270647	1.776902267	4.04E-20	1.74E-19
DNAJC3-DT	3.549417	1.3964732	-1.34579423	1.95E-28	1.88E-27
AC010273.3	0.79562	0.1425047	-2.48107019	1.22E-27	1.11E-26
AP001628.1	0.081387	0.3470573	2.092296004	1.03E-11	2.07E-11
AC010997.4	0.675294	0.2979196	-1.18059325	4.23E-26	3.25E-25
AC009495.2	0.078292	0.4410911	2.494133762	1.51E-16	4.65E-16
AC010261.1	0.041591	0.3321782	2.997617183	3.21E-17	1.07E-16
AP001099.1	0.020376	0.1141903	2.486508611	7.47E-10	1.29E-09
AC078993.1	0.075041	0.3069142	2.032087202	1.28E-07	1.86E-07
AL162171.2	0.045587	0.1364718	1.581894389	1.13E-06	1.55E-06
AC025580.3	0.051429	0.7980728	3.955854985	3.67E-32	5.17E-31
PABPC4-AS1	0.092717	0.2732786	1.559461839	2.47E-06	3.32E-06
AP003774.2	0.049876	0.3130732	2.650069621	1.90E-25	1.35E-24
AC068790.6	0.076556	0.3034911	1.987066804	1.74E-08	2.71E-08
C9orf139	0.037343	0.1949966	2.384530176	1.24E-26	1.01E-25
AL031714.1	0.188508	0.7984534	2.082583583	1.83E-24	1.20E-23
AC009318.4	0.18669	0.4402339	1.237625433	2.14E-05	2.70E-05
AC087289.2	0.107233	0.2929995	1.450151506	5.98E-15	1.60E-14

AC124312.2	2.647263	1.1861536	-1.15821073	6.40E-25	4.36E-24
AC103563.7	5.173696	0.5979776	-3.11303212	1.51E-36	4.42E-35
AC007996.1	2.845347	0.8783425	-1.69574892	2.25E-34	4.35E-33
AC104564.3	0.177313	0.7809168	2.138872329	7.16E-18	2.55E-17
LINC01788	1.625362	0.4287701	-1.92248532	2.63E-26	2.07E-25
MPPED2-AS1	0.935705	0.0759365	-3.62318757	4.36E-41	3.32E-39
AL445222.1	0.208555	0.7307264	1.808902031	3.59E-29	3.87E-28
LINC01983	2.205006	0.0351349	-5.97173233	1.14E-53	3.48E-51
AC103770.1	0.021518	0.1198986	2.478210815	3.07E-08	4.71E-08
AC103719.1	0.011957	0.1176052	3.297996354	2.02E-20	8.98E-20
AC016722.2	0.087689	0.2505847	1.514830009	1.58E-14	4.09E-14
AC016590.1	0.045762	0.121692	1.411026331	6.85E-10	1.19E-09
AC040977.1	2.882707	8.4777683	1.556260425	5.30E-33	8.50E-32
PDXDC2P-NPIPB14P	0.252385	0.7960805	1.657288316	5.66E-16	1.64E-15
AP006621.2	0.441335	1.8541614	2.07082005	3.74E-14	9.36E-14
AC025766.1	0.122196	0.5453796	2.158065523	8.07E-18	2.85E-17
IRF1-AS1	0.570751	1.4444243	1.339559996	4.01E-26	3.09E-25
AC027601.1	0.052105	0.2829591	2.44108754	1.49E-24	9.81E-24
LINC02048	0.024105	1.2853804	5.736728137	3.02E-36	8.01E-35
AC104971.3	0.075359	0.1679419	1.156110614	0.001211446	0.001372

AP006545.2	0.081058	0.1997157	1.300926279	6.78E-06	8.85E-06
AC117500.2	0.035268	0.221652	2.651874151	3.44E-15	9.50E-15
AC138393.2	0.186657	0.3890957	1.059736961	0.001242173	0.001403
AL162274.2	0.75212	1.5829928	1.073619219	1.05E-14	2.75E-14
AC015818.2	0.009768	0.1141707	3.547013978	1.18E-12	2.58E-12
AL359715.3	1.319979	0.5559961	-1.2473682	4.50E-33	7.27E-32
AC091212.1	0.061828	0.1428644	1.208309037	0.000622892	0.000719
AC092809.4	0.057034	0.3393619	2.572933819	9.38E-34	1.66E-32
UBE2Q1-AS1	0.096731	0.4314416	2.157117404	6.45E-19	2.53E-18
RRN3P2	0.173627	0.5307573	1.612060513	1.28E-20	5.80E-20
AL137244.1	0.103612	0.228633	1.141845825	9.17E-06	1.19E-05
AC005498.2	0.264467	0.0922028	-1.52020513	1.15E-30	1.39E-29
ZNF503-AS2	2.694724	0.8388525	-1.68364828	8.20E-35	1.72E-33
AC009095.1	0.066934	0.2233319	1.738385576	2.95E-17	9.85E-17
AL358334.2	0.353967	0.1292785	-1.45313081	1.50E-13	3.56E-13
AC008663.3	0.030585	0.1977239	2.692603885	7.42E-15	1.97E-14
LINC00987	0.771258	1.5508702	1.007792675	3.34E-18	1.24E-17
PKP4-AS1	0.06376	0.1710543	1.423721497	2.42E-13	5.64E-13
AC092436.2	0.009227	0.1388197	3.911192155	1.06E-16	3.33E-16
AL662844.3	0.115057	0.9892065	3.103921854	2.20E-28	2.12E-27

AC141002.1	0.183338	0.5411744	1.561590365	6.20E-14	1.52E-13
AC135050.3	0.160602	1.4822466	3.206222849	1.68E-30	2.00E-29
LINC00954	0.065128	0.1841028	1.499162683	9.18E-09	1.46E-08
BCL2L1-AS1	0.074774	0.5031648	2.750417358	2.70E-14	6.85E-14
AC002553.1	0.484197	1.3757977	1.506602615	2.07E-18	7.86E-18
AL355916.2	0.144584	0.4240471	1.552314952	6.72E-12	1.38E-11
AP002907.1	0.159508	0.5459742	1.775199932	6.88E-15	1.84E-14
AC022154.1	0.068216	0.1641545	1.266869456	2.66E-10	4.78E-10
AL022328.1	0.488095	1.2782961	1.388989173	3.64E-15	1.00E-14
AL033397.2	0.065993	0.2915479	2.14334442	7.60E-18	2.70E-17
AL049552.1	0.211944	0.7042798	1.732464473	2.94E-14	7.42E-14
RNF139-AS1	0.128615	0.2586035	1.007679802	7.27E-06	9.48E-06
PTPRJ-AS1	0.030316	0.5564866	4.198216407	8.05E-05	9.83E-05
AC135178.2	0.07089	0.2967742	2.065721327	4.47E-12	9.33E-12
AL391832.2	0.10832	0.2187696	1.01411719	1.09E-13	2.62E-13
AL049840.6	0.282956	0.8653662	1.612733299	2.81E-13	6.50E-13
AC002059.1	0.089059	0.5298917	2.572863073	2.61E-20	1.14E-19
AKT3-IT1	0.039741	0.2280903	2.520910496	1.27E-09	2.16E-09
EDRF1-AS1	0.04138	0.1198054	1.533683702	2.91E-10	5.20E-10
AL049840.2	0.645291	1.8459844	1.516367431	2.55E-12	5.41E-12

AF111169.3	0.075762	0.4555298	2.588000322	4.35E-25	3.02E-24
AC004837.2	0.080246	0.2703659	1.752411872	1.10E-12	2.42E-12
C15orf56	0.905765	0.0963104	-3.23337308	4.24E-36	1.10E-34
AC027514.2	0.124013	0.3173804	1.355716231	3.51E-07	4.97E-07
AL928921.1	0.6653	0.2293122	-1.53669276	1.13E-36	3.50E-35
AC103858.1	0.034524	0.1881759	2.446428137	2.32E-16	7.04E-16
AGBL5-AS1	0.063257	0.1340567	1.083551003	1.29E-05	1.64E-05
AC108860.2	1.185898	0.0623401	-4.24967682	2.31E-40	1.54E-38
AC073487.1	0.133786	0.6040393	2.174715204	2.05E-19	8.36E-19
AC007406.2	2.072276	8.0508497	1.957924662	4.38E-18	1.61E-17
AC138028.4	0.342257	1.2580709	1.878062281	2.75E-17	9.18E-17
AL133330.1	0.043471	0.2411238	2.471649156	8.02E-15	2.12E-14
TP73-AS3	0.040003	0.3667755	3.196732746	1.62E-15	4.58E-15
LINC00342	0.591509	3.1522182	2.41389617	6.71E-27	5.62E-26
AC110285.2	0.56244	1.8149495	1.690158581	1.44E-05	1.82E-05
AC107021.2	0.34737	2.4428883	2.814042716	7.27E-36	1.81E-34
AC004223.2	0.024311	0.1397041	2.522679173	1.72E-09	2.90E-09
ZNF775-AS1	0.083602	0.1779632	1.08997414	0.000400941	0.000468
AC007220.1	0.133485	0.3010954	1.173541918	2.34E-07	3.36E-07
AC127024.5	0.722971	1.5413852	1.092217304	3.93E-11	7.52E-11

AL078604.2	0.034753	0.3554627	3.354469884	4.46E-29	4.77E-28
AL355001.1	0.067684	0.1616887	1.256337907	0.002263487	0.002506
AC092142.1	0.419778	0.1947079	-1.108315	1.20E-20	5.46E-20
AC023830.3	0.042602	0.171932	2.012861335	3.03E-13	7.00E-13
AC112722.1	0.090854	0.3281007	1.852524232	5.18E-15	1.40E-14
PRR7-AS1	0.038296	0.3823219	3.319507799	1.99E-33	3.36E-32
AC099811.4	0.039752	0.2524644	2.666977847	2.65E-10	4.77E-10
AP001893.3	0.012142	0.1296466	3.416512357	7.85E-22	4.04E-21
AL022322.1	0.196393	1.3763847	2.809065905	8.38E-24	5.11E-23
AC245884.9	0.020239	0.1191442	2.557472994	4.35E-11	8.29E-11
LINC01571	3.224633	0.0249142	-7.01602114	1.75E-48	2.43E-46
AC005342.2	0.017222	0.1186012	2.783820397	7.60E-15	2.02E-14
AC092118.2	0.199201	0.5601697	1.491639115	1.05E-09	1.79E-09
AP003419.3	0.174522	0.562228	1.687748789	8.50E-12	1.73E-11
AP003170.3	0.120059	0.4457109	1.892365774	7.08E-10	1.22E-09
ZBTB20-AS1	0.029178	0.1703416	2.545499515	1.53E-11	3.01E-11
AC138932.4	0.194845	0.5692178	1.546654359	1.64E-13	3.88E-13
PTOV1-AS2	0.642868	2.7714832	2.108063257	1.53E-18	5.85E-18
AC004817.2	0.052552	0.1807525	1.782186072	0.003776694	0.004107
AC005096.1	0.011495	0.135933	3.563877842	1.38E-10	2.55E-10

AC005277.2	0.051543	0.1778766	1.787029363	0.004500971	0.004866
AC021755.2	0.231235	0.0970429	-1.25266642	1.54E-23	9.30E-23
AC011466.1	0.095968	0.2433419	1.342352346	1.00E-10	1.87E-10
TRG-AS1	0.110214	0.712891	2.693379826	1.82E-35	4.23E-34
AC026369.3	0.02899	0.4192243	3.854071334	1.62E-28	1.60E-27
AP003071.4	1.116454	0.1698233	-2.7168173	1.67E-33	2.87E-32
AC005899.7	0.089889	0.3738358	2.056190186	4.24E-15	1.15E-14
SNHG25	0.347611	1.3105749	1.914655259	1.77E-16	5.41E-16
AC091180.4	0.047962	0.2231867	2.218280616	3.70E-13	8.46E-13
AC022400.4	0.224677	0.5819521	1.373050099	2.67E-16	8.00E-16
AC016737.2	0.131868	0.3152271	1.257292265	3.57E-07	5.06E-07
AL021707.6	0.457954	1.9203098	2.068065251	1.75E-18	6.66E-18
AC079210.1	0.087124	0.2699103	1.631333621	0.000139362	0.000167
AC023906.5	0.126821	0.4766351	1.910092886	8.17E-20	3.44E-19
LINC02656	0.23962	0.5308931	1.147672924	3.15E-09	5.19E-09
AC103563.2	1.603528	0.289783	-2.46820526	2.54E-32	3.66E-31
AL139275.2	0.793857	0.1413515	-2.48959114	1.60E-36	4.49E-35
AP005899.1	0.151139	0.820446	2.4405323	3.40E-27	2.98E-26
AP002490.1	0.115635	0.3054644	1.401422713	1.63E-15	4.60E-15
AC104365.2	0.015279	0.1236628	3.016769478	2.59E-14	6.58E-14

AL031186.1	0.224112	0.606647	1.436637513	1.47E-11	2.91E-11
AL161909.2	0.011165	0.1155852	3.371955072	2.18E-08	3.37E-08
AC245884.8	0.222197	1.30575	2.554966392	1.03E-21	5.20E-21
AC012409.1	2.697268	1.2780882	-1.07751124	1.20E-18	4.64E-18
AL034550.1	0.13433	0.3414894	1.346058719	2.62E-10	4.72E-10
AL390728.5	2.316115	6.033091	1.38119062	3.64E-22	1.92E-21
AL353801.3	0.088063	0.4010323	2.187102768	1.87E-14	4.79E-14
AL356652.1	0.030682	0.2199969	2.842024733	3.82E-23	2.20E-22
AL031848.1	0.119281	0.4000934	1.745974264	0.000576926	0.000667
NARF-IT1	0.095803	0.4236715	2.144801807	1.37E-17	4.74E-17
AC006064.3	0.165555	1.1096419	2.744709501	6.22E-32	8.38E-31
AC090607.1	0.07131	0.1872114	1.392498003	1.46E-08	2.29E-08
AL359220.1	0.091878	0.2272155	1.306269068	3.82E-12	8.02E-12
AC116914.2	0.23694	1.3745031	2.536315191	1.69E-22	9.23E-22
AC009054.1	0.015236	0.1117916	2.875215185	5.26E-07	7.36E-07
AC079384.1	0.015391	0.2019082	3.713494014	1.64E-14	4.23E-14
PCCA-DT	5.936277	2.4436757	-1.28050561	2.04E-32	2.98E-31
AC004241.3	0.600067	0.2797122	-1.10118105	1.54E-25	1.11E-24
LINC02115	0.034956	0.1729772	2.306975861	3.73E-06	4.94E-06
AL136295.2	0.186265	0.643394	1.78834447	2.34E-18	8.83E-18

ITCH-IT1	0.03145	0.2208173	2.81172792	1.64E-07	2.37E-07
LINC02481	0.106716	0.3037438	1.509082232	5.79E-21	2.70E-20
AL021707.3	0.332287	0.8957846	1.430720634	9.77E-17	3.08E-16
AC025627.1	0.300878	1.3725032	2.189557791	1.03E-12	2.28E-12
AC002558.3	0.015094	0.1235472	3.032986086	3.13E-09	5.17E-09
AC024592.1	0.016305	0.1263337	2.953869168	3.68E-09	6.02E-09
LINC01359	0.064567	0.292444	2.17928178	2.26E-17	7.64E-17
AC010809.2	0.181628	0.4193779	1.207268079	1.50E-08	2.34E-08
SUCLA2-AS1	0.831176	0.367383	-1.17786918	8.57E-28	7.91E-27
AC069257.1	0.036757	0.1241524	1.756040735	4.87E-09	7.90E-09
AC020913.3	0.074073	0.6179734	3.060524133	1.27E-13	3.03E-13
CCDC144NL-AS1	0.074735	0.159614	1.094725353	5.43E-08	8.17E-08
GTSE1-DT	0.813555	0.2902593	-1.4868987	1.57E-26	1.27E-25
LINC01094	0.27725	1.9636313	2.824262357	6.62E-37	2.24E-35
Z99289.2	0.011043	0.1181151	3.418965834	3.69E-21	1.75E-20
AC072022.1	0.064347	0.2116255	1.717562686	4.19E-11	8.01E-11
LINC01160	0.086835	0.2262753	1.381735906	1.33E-11	2.64E-11
AC010320.3	0.103724	0.2784603	1.424727294	4.27E-10	7.52E-10
AC087742.1	0.072591	0.1963902	1.435867152	5.14E-08	7.75E-08
AL807752.5	0.076073	0.1914603	1.331580265	2.53E-13	5.89E-13

AL512652.1	0.070395	0.2550797	1.857411388	8.37E-14	2.03E-13
AC008467.1	0.029967	0.1270019	2.083415378	5.53E-20	2.35E-19
OSMR-AS1	0.209446	0.4993029	1.253333934	4.20E-17	1.38E-16
AC007390.1	0.228447	0.4705402	1.042458206	4.31E-11	8.22E-11
LMNTD2-AS1	0.309567	4.0846171	3.721875897	7.15E-25	4.84E-24
AC145285.3	0.06464	0.2624114	2.021330616	3.06E-12	6.47E-12
AC073575.2	0.140452	0.3990354	1.506435415	1.12E-18	4.34E-18
AC090772.3	0.244647	0.5070359	1.051383628	1.22E-12	2.65E-12
AC015727.1	0.117865	0.4039734	1.77711929	1.10E-12	2.40E-12
HCG27	0.202245	1.5784507	2.964331308	3.72E-32	5.21E-31
AC136475.5	0.110998	0.2428601	1.129587996	1.77E-09	2.98E-09
AC091729.2	0.0932	0.4957612	2.411242531	1.39E-08	2.17E-08
MRPS9-AS2	0.662566	0.096932	-2.77301985	2.30E-40	1.54E-38
AC006270.1	0.035903	0.3785366	3.398237181	2.08E-09	3.49E-09
AC104463.2	0.134376	0.4470721	1.734233873	1.44E-10	2.64E-10
GK-IT1	0.043121	0.2574258	2.57769854	3.06E-09	5.05E-09
AC020978.7	0.055781	0.1639405	1.555327963	4.66E-16	1.36E-15
AC004494.1	0.020401	0.1641213	3.008051789	1.55E-27	1.39E-26
MIR3945HG	0.04707	0.1575961	1.743353587	4.21E-12	8.81E-12
LINC01138	0.624318	1.3889183	1.153608964	1.65E-20	7.42E-20

AL031123.2	0.850461	0.2290174	-1.89278737	2.88E-32	4.11E-31
Z97353.2	0.04886	0.1522135	1.639377242	4.38E-06	5.77E-06
AL359710.1	0.197531	0.0879591	-1.16717345	8.85E-27	7.32E-26
AC005529.1	0.021017	0.114406	2.444532569	6.16E-06	8.06E-06
AL021707.7	0.12328	0.576657	2.225772099	5.35E-17	1.73E-16
SH3BP5-AS1	0.638037	1.6592061	1.378781397	2.75E-13	6.37E-13
AC011498.6	0.140206	0.65071	2.214471433	1.51E-22	8.27E-22
AC090510.2	0.083298	0.438558	2.396421106	5.35E-25	3.70E-24
LINC00894	0.117141	0.5450075	2.218029182	2.86E-15	7.93E-15
LINC01842	0.049247	0.1919204	1.962394692	9.63E-13	2.14E-12
AD001527.1	0.127594	0.5760039	2.174521488	4.91E-13	1.11E-12
HMGA1P4	0.423841	0.9523607	1.167984015	0.000448726	0.000522
AL133410.1	0.245267	0.5274864	1.10478223	1.29E-05	1.64E-05
AC105339.2	0.0388	0.1440825	1.892761381	5.46E-15	1.47E-14
AC017104.1	0.252636	0.6570473	1.378937501	2.51E-23	1.48E-22
AL121672.1	0.502227	0.2208465	-1.18529571	4.91E-23	2.82E-22
AC010524.1	0.05379	0.153941	1.516975675	0.000169151	0.000202
AC009084.1	0.064694	2.0126708	4.959327639	1.71E-26	1.36E-25
AL590666.1	0.102628	0.2552596	1.314539992	2.03E-06	2.74E-06
ANKRD44-IT1	0.017022	0.2049556	3.589839711	1.88E-12	4.03E-12

AC008870.2	0.140459	0.6086958	2.115569829	5.25E-22	2.75E-21
LINC01290	0.046307	0.1168244	1.335037459	3.80E-05	4.71E-05
AC013553.3	0.125077	0.4928339	1.978283978	9.77E-22	4.93E-21
AL354863.1	0.911144	0.0399158	-4.5126465	2.48E-49	4.04E-47
DPP9-AS1	0.027381	0.2538331	3.212652398	3.88E-32	5.37E-31
AC100830.2	0.236398	0.9966195	2.075824686	1.22E-17	4.24E-17
AC078788.1	0.023599	0.2124053	3.170031185	3.06E-07	4.35E-07
AC009159.2	0.089244	0.7322489	3.036512356	8.60E-28	7.92E-27
AC009754.1	0.038447	0.2149766	2.483222821	1.73E-09	2.92E-09
Z93403.1	0.530354	0.0739218	-2.84288368	7.53E-33	1.17E-31
AL451050.2	0.083934	0.2466725	1.55526598	5.27E-09	8.49E-09
AL008582.1	0.135248	0.5773018	2.093721998	2.57E-16	7.73E-16
MMP2-AS1	0.020772	0.6203472	4.900386568	9.41E-32	1.25E-30
LINC02298	0.892068	1.9234697	1.10848516	3.67E-16	1.09E-15
LINC01985	0.096907	0.2242121	1.210187054	3.32E-06	4.41E-06
LINC01126	0.137912	0.5203587	1.915759354	1.16E-19	4.82E-19
NUCB1-AS1	0.023257	0.1190301	2.355596779	1.13E-10	2.10E-10
AC006033.2	0.108627	0.5429317	2.321384119	3.91E-33	6.44E-32
AC112220.2	2.461206	1.1672216	-1.07628689	6.85E-33	1.07E-31
Z99572.1	1.842449	0.321199	-2.52008522	1.21E-37	4.55E-36

AC098851.1	0.254308	0.5167571	1.02290903	9.91E-05	0.00012
AL031717.1	0.122615	0.5913043	2.269763454	2.41E-19	9.73E-19
AC007000.3	0.085741	0.3097089	1.852856288	1.03E-08	1.62E-08
AC073592.1	0.04509	0.1793514	1.991899965	1.09E-08	1.72E-08
INSYN1-AS1	1.006203	0.2721727	-1.8863276	4.63E-38	1.88E-36
AC104237.2	3.063654	0.1492802	-4.35915917	3.58E-48	4.59E-46
AC093864.1	0.063302	0.1755071	1.47120709	9.15E-10	1.57E-09
AC012186.2	0.147331	0.4608843	1.645342923	1.54E-14	3.99E-14
AC078883.1	0.372244	1.5894112	2.094170549	6.83E-39	3.20E-37
AL807757.2	0.068583	0.2409213	1.812631913	1.06E-08	1.67E-08
AC012409.3	0.499785	1.0575937	1.081406657	1.45E-06	1.98E-06
AL121999.1	0.487652	0.1392037	-1.80865508	1.33E-06	1.82E-06
AL391095.1	0.044279	0.1597257	1.850915252	1.59E-12	3.43E-12
AC093801.1	0.048228	0.184352	1.934516138	5.44E-08	8.18E-08
IQCH-AS1	1.797153	0.8848284	-1.02224361	3.25E-33	5.39E-32
RNF157-AS1	1.457353	0.3275461	-2.15358051	1.15E-30	1.39E-29
MCF2L-AS1	2.411878	0.4982379	-2.27525044	1.59E-36	4.49E-35
AC023510.1	0.033368	0.1537129	2.203698273	5.48E-11	1.04E-10
AC145098.1	0.2015	1.1406318	2.500985012	5.77E-34	1.03E-32
LINC01543	6.519592	0.0630675	-6.69174063	3.21E-39	1.66E-37

LINC02154	0.023894	0.3376052	3.820607984	1.13E-16	3.52E-16
AL049539.1	0.044403	0.2368896	2.415473789	1.08E-07	1.59E-07
AC145423.2	0.344615	0.7568021	1.134931976	1.05E-06	1.44E-06
AC113143.1	0.057174	0.2224126	1.959803224	0.00033073	0.000389
AC009090.1	0.253848	0.8138726	1.680837638	3.89E-14	9.71E-14
AC000068.1	0.584852	0.1841715	-1.6670217	1.63E-34	3.25E-33
AC012531.1	0.218905	0.4632674	1.081540451	1.10E-06	1.51E-06
AC003965.2	0.221813	0.0984997	-1.17115088	8.16E-33	1.26E-31
AC004039.1	0.054409	0.1218442	1.163119788	1.30E-07	1.89E-07
AC004930.1	0.025189	0.3164043	3.650881072	6.34E-13	1.42E-12
AC074029.3	0.117005	0.3402184	1.539887375	3.21E-11	6.17E-11
MIR210HG	1.204069	11.550133	3.261919102	8.61E-37	2.80E-35
AC011939.2	0.008456	0.2406384	4.830662877	7.92E-11	1.48E-10
AC092338.1	0.111578	0.4283013	1.9405703	1.49E-13	3.54E-13
AC023590.1	0.025094	0.1115327	2.152068464	3.71E-06	4.92E-06
AC138230.1	0.183758	0.4840327	1.397295506	1.32E-08	2.07E-08
AP000757.1	8.871118	1.183178	-2.90644883	1.56E-36	4.49E-35
AC006960.2	1.009432	0.0438581	-4.52455677	1.77E-28	1.73E-27
AL355488.1	0.557591	1.8250681	1.710670434	1.18E-16	3.68E-16
AL357874.2	0.056267	0.1964578	1.803854621	7.43E-11	1.40E-10

LINC02038	2.40652	0.2329672	-3.36874935	5.50E-41	4.06E-39
AC012676.4	0.038374	0.1312086	1.773678526	1.31E-09	2.23E-09
AL731563.3	0.124385	0.3310013	1.412027437	2.65E-20	1.16E-19
AL354733.3	0.578837	1.5041608	1.377730187	1.09E-12	2.39E-12
AC132192.2	0.393124	0.861512	1.131885175	8.01E-06	1.04E-05
PHKA2-AS1	0.171816	1.2164396	2.823727095	4.87E-35	1.06E-33
AL355075.2	0.189105	0.7201101	1.929032464	1.15E-13	2.75E-13
LINC02568	1.679223	0.1775667	-3.24136033	6.99E-38	2.70E-36
LINC01012	0.022768	0.1132307	2.314177616	2.01E-23	1.20E-22
AC129507.4	1.895806	0.5397255	-1.81251374	2.09E-24	1.36E-23
AC067852.3	0.16725	0.6092041	1.864921874	6.88E-18	2.46E-17
AC130650.2	0.128327	0.2768416	1.109230817	1.01E-08	1.60E-08
LINC01827	0.016488	0.1555967	3.238279995	5.72E-18	2.06E-17
AL162400.2	0.93915	0.0464667	-4.33708715	6.92E-51	1.41E-48
FRY-AS1	0.254731	0.106377	-1.25978876	9.93E-27	8.18E-26
AL360091.1	0.128206	0.3193164	1.31652507	8.21E-05	0.0001
AC107993.1	0.04247	0.1964854	2.209900276	2.64E-13	6.12E-13
AC099811.1	0.031934	0.1381475	2.113039761	9.76E-12	1.97E-11
SRI-AS1	0.052158	0.1094867	1.069782585	0.000724965	0.000832
AP006621.4	0.235821	1.0013697	2.086210739	3.25E-12	6.86E-12

AC021851.1	0.139903	0.3149452	1.17067871	5.95E-15	1.60E-14
AC005324.4	0.044988	0.1849046	2.03918272	2.09E-14	5.35E-14
AC091185.1	0.171909	0.8467357	2.300264953	7.20E-24	4.44E-23
AL034417.2	0.07024	0.1680736	1.258736753	0.000748843	0.000858
AP001107.8	0.0654	0.3976672	2.604207188	1.52E-12	3.28E-12
FAM182B	0.09904	0.2362448	1.254200809	1.43E-16	4.41E-16
AL109947.1	0.07728	0.2453541	1.666697062	3.25E-06	4.31E-06
ERVE-1	3.415182	0.0986345	-5.11372556	1.27E-46	1.55E-44
AC087289.5	0.111148	0.2966922	1.416490382	8.88E-18	3.11E-17
AP003086.1	0.034427	0.1264241	1.87664144	8.78E-06	1.14E-05
AC015813.1	0.572768	1.8607186	1.699837752	2.02E-16	6.15E-16
LINC02416	0.018016	0.1963616	3.44612476	6.71E-13	1.50E-12
AC021739.3	0.031734	0.135748	2.096811671	2.77E-06	3.70E-06
AL078587.1	0.073166	0.2779549	1.925612263	6.17E-10	1.07E-09
AC103691.1	0.388934	1.3329466	1.777021941	8.26E-16	2.37E-15
KANSL1L-AS1	0.221807	0.6241899	1.492679205	6.21E-14	1.52E-13
LINC01607	0.626987	0.24928	-1.33066865	2.97E-20	1.29E-19
RUSC1-AS1	0.482382	1.4609419	1.598649659	1.31E-16	4.06E-16
AC044849.1	0.096538	0.3126911	1.695575773	1.10E-16	3.45E-16
LINC01841	0.035595	0.1326518	1.897905291	1.04E-12	2.30E-12

AC021491.4	0.481505	0.0614338	-2.97044504	1.12E-24	7.48E-24
AL365330.1	0.731326	1.4941226	1.030711211	8.46E-11	1.58E-10
AC106873.1	0.005577	0.1835756	5.040782323	1.04E-09	1.79E-09
AC099552.3	0.953525	0.0467393	-4.35056392	7.92E-65	9.66E-62
AC036103.1	0.050782	0.1771838	1.802854755	2.36E-13	5.52E-13
AC009163.4	0.029372	0.1097659	1.901920429	4.38E-09	7.12E-09
AC107057.1	4.639346	0.0660372	-6.13449854	3.24E-60	2.63E-57
AC040174.1	0.357148	0.1668186	-1.09824258	6.02E-29	6.35E-28
ST7-OT4	0.049004	0.1345977	1.457677204	0.014152204	0.014701
AC011306.1	0.048217	0.1400729	1.53855728	2.06E-08	3.19E-08
CD44-AS1	0.069181	0.302844	2.130121104	4.75E-10	8.34E-10
FMR1-IT1	0.277187	0.7782142	1.4893058	1.35E-11	2.69E-11
GATA3-AS1	1.868654	0.0745238	-4.64815465	1.15E-42	1.10E-40
AC087301.1	0.131415	0.4250572	1.693528084	5.88E-09	9.43E-09
AL353803.5	0.148096	0.5041786	1.76740163	3.02E-09	4.98E-09
AP002807.1	0.311514	0.8487591	1.446059058	3.83E-09	6.25E-09
AC073257.2	0.125383	1.1725035	3.225181748	1.84E-33	3.13E-32
AC040162.3	0.08376	0.3906182	2.221425885	2.31E-25	1.63E-24
AC010976.1	0.072017	0.2419028	1.748014088	2.63E-15	7.31E-15
AC009159.1	0.021385	0.2736199	3.677481398	1.31E-24	8.75E-24

AL158163.1	0.162387	0.5376035	1.727110652	1.97E-21	9.59E-21
AP003390.1	0.138547	0.2894356	1.062865929	1.19E-07	1.73E-07
AC139720.1	0.027729	0.1314863	2.245469743	1.23E-09	2.10E-09
AP001207.3	0.655183	0.0292419	-4.48579064	2.14E-49	3.73E-47
AC068790.5	0.149479	0.657822	2.13775464	5.98E-22	3.11E-21
GARS1-DT	0.361001	1.1718959	1.698768013	2.32E-24	1.49E-23
SPON1-AS1	0.090234	0.264997	1.55423695	1.65E-05	2.09E-05
AL121782.1	0.072199	0.2532259	1.81036756	5.62E-15	1.51E-14
AC092123.1	0.197117	0.4717184	1.258874697	1.88E-07	2.72E-07
AC018450.1	0.106236	0.3670074	1.788539709	6.44E-19	2.53E-18
AC020978.3	0.139304	0.5884763	2.078751949	2.66E-16	7.98E-16
LINC01230	2.447813	0.7488235	-1.70879572	2.60E-39	1.38E-37
AL139349.1	0.288177	1.6520009	2.519185503	6.87E-17	2.20E-16
AC005837.4	0.045822	0.3192157	2.800411871	1.59E-10	2.92E-10
AL592211.1	0.077969	0.2400841	1.622567022	1.34E-07	1.95E-07
AC004918.1	1.559503	4.3103743	1.466727123	6.28E-24	3.91E-23
AC084809.1	0.275561	0.1212071	-1.18489769	6.31E-21	2.91E-20
AC073263.1	0.052208	0.1182054	1.178952805	0.000202811	0.000241
AC008982.2	0.147223	0.6042206	2.037070751	1.18E-17	4.09E-17
LINC02798	0.23489	0.0974994	-1.26852159	6.22E-08	9.28E-08

AC025178.1	0.0835	0.3437007	2.04129719	2.56E-20	1.12E-19
FO393418.1	0.299395	0.1098768	-1.44616062	1.13E-07	1.66E-07
AC010422.2	0.079125	0.2656223	1.747174153	1.59E-11	3.13E-11
U47924.3	0.101494	0.4370193	2.106298217	6.76E-17	2.17E-16
AC010205.1	0.468296	0.1423253	-1.71822768	2.98E-21	1.43E-20
AL035658.1	0.029867	0.1488892	2.317623516	1.45E-10	2.67E-10
AC011442.1	0.083727	0.2079124	1.312216736	8.41E-05	0.000102
TSPOAP1-AS1	0.087638	0.2615575	1.577494515	1.14E-21	5.69E-21
AC027607.1	0.160421	0.4892096	1.608588473	5.02E-19	1.98E-18
AC008514.1	0.58541	0.2651995	-1.1423688	3.72E-23	2.15E-22
SEC62-AS1	0.119915	0.2912898	1.280444064	2.01E-08	3.11E-08
AC009831.3	0.043643	0.1515192	1.795664068	6.49E-08	9.67E-08
AL117379.1	0.369185	1.1014655	1.57700877	3.60E-14	9.02E-14
AP001767.2	0.207632	0.5624149	1.437602566	6.03E-12	1.24E-11
AC034102.5	0.063965	0.1824188	1.51190331	1.17E-07	1.71E-07
LINC02604	0.866312	2.6341926	1.604401693	8.71E-18	3.06E-17
AL110115.2	0.063728	0.1568645	1.299526161	2.38E-06	3.21E-06
LINC02157	0.149541	0.3697553	1.306033742	2.60E-06	3.49E-06
AC096733.2	1.7752	0.632468	-1.48891728	1.26E-32	1.90E-31
U91328.3	0.178702	0.4272658	1.257580769	3.44E-07	4.87E-07

AC074138.1	0.12254	0.2800148	1.192247227	2.08E-08	3.22E-08
AC022973.4	0.035347	0.1989955	2.493061297	0.003202694	0.003498
AL359504.1	0.218444	0.5835939	1.417700333	3.07E-10	5.48E-10
AC091180.2	0.040414	0.1160628	1.521991059	8.53E-11	1.60E-10
AL158151.2	0.030333	0.1780593	2.55341975	3.48E-13	7.99E-13
AC073957.3	0.336634	0.7942598	1.238431712	2.20E-07	3.16E-07
AC245060.6	0.071961	0.2027941	1.494726332	7.29E-13	1.63E-12
CR559946.2	0.029141	0.1812766	2.637094435	3.47E-16	1.03E-15
GLIS3-AS1	0.332751	0.1056615	-1.65499483	8.35E-16	2.39E-15
AL158847.1	0.52395	0.0454721	-3.52637564	1.92E-39	1.09E-37
AL731571.1	0.207636	0.6382088	1.619971064	1.56E-21	7.69E-21
AC012404.2	0.034537	0.490434	3.827864677	2.12E-13	4.98E-13
AC018521.5	1.243989	0.5791761	-1.10289943	2.72E-32	3.90E-31
IBA57-DT	0.121534	0.3373489	1.472877982	1.02E-11	2.06E-11
CARMN	0.107672	0.4094897	1.927180789	5.49E-12	1.14E-11
LINC00664	0.022162	0.1394249	2.653309921	1.45E-21	7.19E-21
DTX2P1-UPK3BP1-PMS2P11	0.082123	0.2893769	1.817090211	2.98E-26	2.32E-25
AC012157.2	0.030719	0.1561695	2.345914701	3.58E-16	1.06E-15
AC009159.3	1.631307	3.9933577	1.291574009	1.95E-13	4.59E-13
LINC01356	0.041419	0.1659363	2.002269156	1.09E-25	8.03E-25

AC010261.2	0.041847	0.2262961	2.435029342	2.85E-15	7.92E-15
LINC02334	0.334425	0.1639156	-1.02872991	0.000400909	0.000468
IGFL2-AS1	0.021451	0.82744	5.269534248	2.01E-07	2.90E-07
LINC00106	0.595311	2.7358534	2.200276242	1.03E-11	2.07E-11
LINC00861	0.089858	0.728956	3.020109843	1.93E-31	2.48E-30
AC093110.1	0.407162	1.0899281	1.42055886	1.34E-13	3.19E-13
AL513327.2	0.086487	0.2317885	1.422248417	1.46E-07	2.12E-07
AC104984.5	0.010871	0.2195726	4.336191516	1.54E-11	3.04E-11
AC011603.2	0.040794	0.1386276	1.76476814	4.04E-19	1.60E-18
AGAP2-AS1	1.581781	5.3620998	1.76124845	4.16E-27	3.59E-26
AL021154.1	0.173318	0.441543	1.34913377	3.20E-11	6.15E-11
AL139123.1	0.08277	0.3608587	2.124249391	1.48E-14	3.83E-14
AC104806.2	0.021919	0.1124808	2.359428683	7.08E-17	2.27E-16
AC138811.1	0.061049	0.174943	1.518854207	3.59E-05	4.47E-05
AC243964.3	2.639446	0.7277028	-1.85881381	7.81E-25	5.28E-24
AC092653.1	0.136185	0.3872628	1.507749554	7.96E-18	2.82E-17
AC015849.1	0.02149	0.1267407	2.560174871	1.13E-15	3.24E-15
AC004263.1	0.039762	0.2664266	2.744287861	2.39E-16	7.21E-16
AC131097.2	0.29592	0.0905917	-1.70775525	6.22E-21	2.87E-20
AL391840.1	0.46562	0.1348763	-1.78751578	7.98E-19	3.11E-18

LINC02803	0.058948	0.2497262	2.08283551	1.45E-15	4.09E-15
PACERR	0.055873	0.1686521	1.593815897	2.60E-05	3.25E-05
AC211433.1	0.022372	0.1503258	2.748343739	4.23E-14	1.05E-13
AC073517.1	0.075615	0.1854669	1.294423737	2.07E-08	3.20E-08
AL359697.1	0.174768	0.3988581	1.190436976	5.82E-06	7.64E-06
AC144548.1	0.082715	0.2505578	1.59891567	3.59E-13	8.24E-13
AC138150.1	0.152175	0.3246704	1.093245412	0.001695939	0.001901
AC090559.2	0.006759	0.1990202	4.880022329	7.74E-12	1.58E-11
LINC01928	0.663338	0.0588461	-3.4947256	1.09E-17	3.79E-17
OPA1-AS1	0.062135	0.2373332	1.933431861	7.76E-08	1.15E-07
PRC1-AS1	0.026583	0.1157148	2.121977354	8.73E-22	4.45E-21
CTD-3080P12.3	0.535036	0.2517787	-1.08748113	0.000574411	0.000664
AL158212.2	0.213801	0.4367912	1.030677373	0.001096527	0.001245
PSPC1-AS2	0.382395	0.8394383	1.134359426	1.44E-10	2.66E-10
AL354977.1	0.059999	0.1830212	1.60900645	3.73E-09	6.10E-09
LINC00921	0.279117	0.7797676	1.482173951	1.09E-30	1.33E-29
AC243585.1	0.40758	0.1543613	-1.40077232	4.27E-39	2.08E-37
AP001160.4	0.155974	0.5426801	1.798800433	1.73E-10	3.17E-10
PSORS1C3	1.595967	15.219311	3.253400412	2.98E-29	3.23E-28
AC006017.1	0.16542	0.4629885	1.484841076	7.41E-09	1.18E-08

MIAT	0.033592	0.73477	4.451086939	1.54E-34	3.11E-33
NFE4	0.027847	0.4833421	4.117441328	9.97E-13	2.21E-12
MIRLET7BHG	0.171123	0.4486074	1.390419279	3.96E-10	7.00E-10
YEATS2-AS1	0.080668	0.4547808	2.495106773	4.10E-30	4.76E-29
AC023669.1	0.304972	1.77907	2.544376546	0.002890134	0.003167
AC023825.2	0.015867	0.2600491	4.034644769	8.88E-25	5.96E-24
AC009087.1	0.079819	0.2213577	1.47156736	7.38E-09	1.18E-08
AC090971.2	0.026189	0.13486	2.364437526	1.22E-10	2.26E-10
LINC02097	0.022045	0.116114	2.39703137	8.40E-20	3.53E-19
AP003096.1	0.059405	0.1949818	1.714693305	1.68E-17	5.76E-17
ITGA6-AS1	0.155974	1.2313549	2.980870457	1.15E-35	2.69E-34
AC010998.2	0.070062	0.2212186	1.658761135	3.84E-10	6.79E-10
AL133406.2	0.080592	0.3005023	1.898667243	9.32E-22	4.72E-21
MELTF-AS1	0.693802	1.4261351	1.039515551	2.13E-07	3.06E-07
RN7SL832P	0.713109	0.2489295	-1.51838456	2.11E-24	1.37E-23
AC004034.1	0.060675	0.2861627	2.237653531	5.28E-18	1.92E-17
AL353699.1	0.92555	0.3166657	-1.54734997	8.29E-43	8.42E-41
AF131215.4	0.016819	0.1332039	2.985454889	9.94E-09	1.57E-08
AC068888.2	0.066524	0.282295	2.085252357	8.44E-14	2.04E-13
AC010326.3	1.614871	3.3665718	1.059861302	2.50E-16	7.54E-16

LINC02828	0.02952	0.1352125	2.195459832	1.98E-19	8.07E-19
AC144831.1	3.273022	0.5531224	-2.56495268	6.58E-35	1.41E-33
SNHG4	0.220818	0.5063117	1.197170756	1.52E-12	3.28E-12
ZEB2-AS1	0.19662	0.4841965	1.300179803	3.30E-18	1.22E-17
UFL1-AS1	0.043733	0.1261731	1.528620402	2.91E-05	3.64E-05
AC084357.2	0.185891	0.3796295	1.030131785	6.95E-09	1.11E-08
MIR194-2HG	0.091248	0.1840954	1.012588695	1.78E-05	2.25E-05
AC007405.3	4.075373	1.9808413	-1.04081887	2.07E-20	9.18E-20
AC093462.1	0.063382	0.1429491	1.173349201	6.32E-11	1.19E-10
LINC01311	0.167619	0.4811944	1.52143558	1.17E-14	3.06E-14
AL513327.1	0.253827	1.0529853	2.052570356	1.35E-24	8.93E-24
CAMTA1-DT	1.149879	0.3640719	-1.65918706	1.48E-26	1.20E-25
HEXD-IT1	0.052582	0.1530463	1.541317045	2.31E-10	4.18E-10
AC048382.1	0.041465	0.1167179	1.493050195	4.89E-10	8.58E-10
AC010761.1	0.345711	1.0655608	1.623973459	4.06E-19	1.61E-18
LINC01237	0.068312	0.196611	1.525141607	8.27E-14	2.00E-13
PLCG1-AS1	0.09488	0.3356659	1.822852587	2.19E-22	1.18E-21
AC099518.1	0.077685	0.2108239	1.440336875	3.45E-19	1.38E-18
NEAT1	11.05923	34.952943	1.660162709	2.89E-09	4.78E-09
ATP6V0E2-AS1	1.195025	0.4983747	-1.26173815	1.62E-26	1.30E-25

AC087284.1	0.018044	0.253645	3.813194254	4.37E-19	1.73E-18
LINC01397	0.012149	0.1242805	3.354669212	5.53E-08	8.30E-08
AC023794.2	1.396664	0.4642876	-1.58889427	6.92E-28	6.47E-27
AC002128.2	0.123364	0.3036524	1.299493708	8.21E-06	1.07E-05
FSIP2-AS2	0.052628	0.189185	1.845884965	9.96E-08	1.47E-07
AC016747.3	0.119159	0.3788486	1.668737169	6.32E-13	1.42E-12
AC005546.1	0.045259	0.1821871	2.009146465	1.30E-10	2.41E-10
AC019197.1	2.144548	0.3313308	-2.6943294	2.06E-35	4.66E-34
AC078980.1	0.497171	0.0631349	-2.97723303	2.47E-41	1.94E-39
AC007546.1	0.176553	0.5919824	1.745455337	1.03E-11	2.07E-11
AC012358.1	0.057363	0.1639793	1.515315681	2.66E-07	3.79E-07
AC026401.3	1.351287	3.5911978	1.410130625	1.23E-32	1.88E-31
AC015853.1	0.031769	0.1420165	2.160354687	9.92E-13	2.20E-12
AL133371.3	0.025707	0.1929757	2.908211748	2.55E-17	8.56E-17
AC147067.1	0.549674	1.1943095	1.119527518	9.27E-18	3.24E-17
AC009950.1	0.130414	0.3562197	1.449664841	6.21E-27	5.22E-26
AC132192.1	0.047848	0.2107762	2.139167422	3.42E-12	7.21E-12
YTHDF3-AS1	1.56728	0.7614016	-1.04153365	3.32E-22	1.77E-21
CEROX1	0.184571	0.6345882	1.7816455	0.002127931	0.002362
AC092143.3	0.028851	0.2170734	2.911510262	3.91E-19	1.56E-18

AC073655.2	0.134649	0.5100422	1.921409625	2.04E-20	9.09E-20
AC025287.3	0.256912	0.6156125	1.26074675	5.68E-11	1.08E-10
AP002812.2	0.047056	0.1756276	1.900068983	5.78E-13	1.30E-12
AC005954.1	0.041963	0.2111444	2.331046014	1.65E-14	4.26E-14
AC009955.3	0.026959	0.1170337	2.118069085	1.74E-08	2.71E-08
TMED2-DT	0.153333	0.3963658	1.37015946	4.88E-11	9.28E-11
AATBC	0.06668	0.1517641	1.186512281	9.53E-12	1.93E-11
AC025154.2	1.175534	0.0635944	-4.2082726	4.31E-50	8.09E-48
AC023813.3	0.048855	0.1860136	1.92882527	2.67E-18	1.01E-17
AC008781.2	0.107345	0.4144238	1.948853766	3.07E-16	9.15E-16
AC010149.1	0.040862	0.1257159	1.621341837	1.75E-09	2.94E-09
AC012653.2	0.323576	0.1597649	-1.01815488	1.55E-26	1.26E-25
AC109597.2	0.015806	0.1233161	2.96382909	1.68E-11	3.29E-11
AP001001.1	0.122396	0.3251271	1.409448256	3.53E-15	9.73E-15
LINC01857	0.14918	1.1763159	2.979148424	1.11E-26	9.14E-26
AC016747.2	0.095318	0.2524805	1.405352369	7.22E-08	1.07E-07
AC002091.2	0.206906	0.7034304	1.765431618	1.35E-20	6.09E-20
AC008147.2	0.05562	0.1477801	1.409786992	5.65E-05	6.94E-05
LCMT1-AS1	0.4188	0.1743824	-1.2640069	1.73E-22	9.44E-22
AC145207.8	0.097559	0.2448868	1.327767589	2.10E-08	3.25E-08

AL031600.1	0.195918	0.8200758	2.065507474	1.04E-12	2.28E-12
PRDM16-DT	13.2686	0.3164715	-5.38979658	2.97E-40	1.91E-38
AL161452.1	0.058054	0.2460434	2.08345325	1.59E-19	6.54E-19
LINC00323	1.036566	0.4251409	-1.28579841	5.19E-21	2.43E-20
WWTR1-IT1	0.081703	0.3881668	2.248220407	6.69E-09	1.07E-08
LMO7-AS1	3.65925	0.6812401	-2.4253129	3.78E-32	5.27E-31
AC105429.1	0.036551	0.1541219	2.076087349	1.40E-12	3.03E-12
AC027279.1	0.038991	0.1898645	2.283748294	4.55E-12	9.47E-12
AC104072.1	2.087981	0.1088295	-4.26196751	5.45E-59	3.32E-56
AC087645.2	0.129728	0.3555412	1.454528593	8.90E-19	3.46E-18
AC010531.3	0.102832	0.2600908	1.338727238	2.07E-07	2.98E-07
ZNF32-AS2	0.396491	0.9356654	1.238704136	8.30E-13	1.85E-12
AL021707.1	0.103824	0.347081	1.741137606	2.82E-14	7.14E-14
GAS8-AS1	0.156197	0.4120573	1.399478298	3.79E-07	5.36E-07
AC104794.2	0.260624	0.102399	-1.34776619	1.57E-19	6.49E-19
LINC02541	0.043189	0.2677042	2.631912573	7.20E-21	3.30E-20
AC055855.1	0.133915	0.5112564	1.932729565	9.50E-19	3.70E-18
LINC01612	3.150561	0.3382029	-3.21964787	2.61E-46	3.02E-44
AC092119.2	0.153225	0.6594992	2.105717364	8.33E-15	2.20E-14
AC097504.2	0.646769	0.2543496	-1.3464368	5.17E-27	4.43E-26

AL139287.1	2.075307	5.4869493	1.402679568	2.36E-14	6.00E-14
NPTN-IT1	0.19857	0.6072576	1.612660825	3.49E-12	7.34E-12
LINC02435	0.049406	0.1580311	1.677451104	0.001935882	0.00216
AC005393.1	0.202	0.4360247	1.110057762	4.26E-09	6.93E-09
TCL6	1.450817	0.2365542	-2.61662262	5.37E-20	2.29E-19
AC012645.3	0.047133	0.4280872	3.183092883	1.21E-29	1.36E-28
LINC01415	0.08676	0.1952932	1.170535307	1.31E-11	2.62E-11
SLC16A1-AS1	0.065275	0.610393	3.225135982	4.00E-40	2.50E-38
Z93241.1	0.051342	0.2810701	2.452716201	2.29E-11	4.48E-11
WDFY3-AS2	5.034594	2.2706684	-1.14875851	1.95E-28	1.88E-27
AC020931.1	0.070789	0.1783782	1.333336073	1.50E-08	2.33E-08
AC010973.1	0.092603	0.2257186	1.285396402	2.14E-06	2.89E-06
AC012593.2	1.673591	16.694444	3.31834897	1.78E-29	1.95E-28
AC026992.1	1.142241	0.4717337	-1.27582267	3.93E-19	1.56E-18
LINC01485	0.914877	0.2240607	-2.02968768	3.48E-30	4.10E-29
AL121899.1	0.289443	0.7904843	1.449457537	1.87E-11	3.66E-11
AL139022.1	0.057926	0.1924375	1.732099248	7.10E-12	1.46E-11
AL049555.1	3.540425	0.3702575	-3.25732163	5.29E-36	1.36E-34
AC090589.3	0.409017	1.3573234	1.730530137	4.99E-20	2.14E-19
PCED1B-AS1	0.477539	3.126144	2.710694017	7.88E-36	1.94E-34

ASAP1-IT2	0.025077	0.2077372	3.050303956	8.05E-24	4.93E-23
AC136469.1	0.023199	0.1530045	2.721416256	7.45E-10	1.29E-09
AC005674.1	0.268052	0.760606	1.50463571	2.70E-11	5.22E-11
AC108727.1	0.20218	0.4709565	1.219954509	5.45E-07	7.62E-07
AC010319.4	0.21186	0.4242705	1.001871605	1.54E-09	2.61E-09
AP4B1-AS1	0.070946	0.2453093	1.789811644	3.36E-15	9.27E-15
LHX1-DT	5.958082	0.3431248	-4.11804246	6.11E-41	4.38E-39
AC026471.5	0.01761	0.1140998	2.69584285	1.07E-12	2.36E-12
AC012615.6	0.180508	0.822616	2.188152647	7.62E-23	4.25E-22
AC017083.1	0.119154	0.283289	1.249445736	8.72E-07	1.21E-06
AC131571.1	0.021295	0.1540464	2.854805241	1.26E-05	1.61E-05
AP001178.1	0.078271	0.3168563	2.017275182	7.84E-12	1.60E-11
LINC01738	0.10075	1.5165006	3.911898475	6.73E-30	7.70E-29
AC008735.4	0.226318	0.5727656	1.339596457	1.99E-14	5.08E-14
AC010530.1	0.079	0.2868948	1.860601677	1.46E-14	3.77E-14
AC099684.2	1.919926	0.4853093	-1.98407397	1.38E-15	3.91E-15
BTG3-AS1	0.419474	0.161276	-1.37905059	3.56E-32	5.05E-31
AC025569.1	0.140288	0.3404662	1.279119466	3.62E-08	5.51E-08
AL928654.2	0.738447	2.173389	1.557379182	1.36E-11	2.70E-11
AL022238.2	0.099237	0.3798083	1.936322843	3.04E-14	7.68E-14

AC004233.3	0.064633	0.1698643	1.39405063	3.37E-08	5.14E-08
AC011462.4	0.384346	1.6669392	2.11672363	2.50E-18	9.45E-18
MSC-AS1	1.220437	3.6993263	1.599864437	5.20E-25	3.60E-24
AC087588.1	0.090828	0.2783392	1.61563471	2.84E-06	3.80E-06
TNFRSF14-AS1	0.585105	1.9184314	1.713159473	2.19E-21	1.06E-20
AC009269.2	0.018447	0.1889746	3.356715802	2.36E-09	3.94E-09
AL161669.1	0.218977	1.0545529	2.267779012	1.76E-17	6.00E-17
HPN-AS1	0.212642	0.700172	1.719281214	4.27E-08	6.46E-08
AC090617.4	0.046086	0.1111035	1.269518805	0.000150516	0.000181
AC108134.3	0.476817	1.2391801	1.377879764	4.65E-11	8.86E-11
AC025265.1	0.078609	1.2509251	3.992158274	3.70E-34	6.94E-33
AC079584.2	0.008977	0.116172	3.693948125	8.69E-22	4.44E-21
AC016026.1	0.033277	0.2385467	2.841695114	1.33E-29	1.48E-28
LINC00173	0.193863	1.9690118	3.344363366	7.19E-27	6.00E-26
AC016027.3	0.016729	0.1858368	3.473599936	8.51E-07	1.18E-06
AC103739.2	0.049492	0.186344	1.912706845	2.61E-16	7.86E-16
AP000553.2	0.09515	0.4203888	2.143453573	2.72E-10	4.88E-10
AC087289.1	0.039581	0.1490263	1.912697185	3.68E-15	1.01E-14
MAP3K2-DT	0.367758	0.8370858	1.186620146	2.38E-10	4.31E-10
Z84485.1	0.192813	0.4936491	1.356284748	1.18E-09	2.01E-09

AC022146.2	0.071907	0.3651031	2.344100573	4.75E-08	7.16E-08
AL354760.1	0.153249	0.3762239	1.295710498	2.75E-11	5.31E-11
AC010148.1	0.064392	0.1518513	1.237705979	0.025428878	0.025896
AC008635.1	0.032849	0.1161108	1.821586462	1.37E-12	2.97E-12
AL583810.1	0.087105	0.1764024	1.018050585	0.000582104	0.000673
AL512306.2	0.120465	0.3582058	1.572172021	2.49E-07	3.55E-07
AL023803.2	0.075842	0.1739055	1.197235593	1.32E-05	1.68E-05
ZDHHC20-IT1	0.097636	0.2862619	1.551854989	2.66E-14	6.75E-14
AC087623.2	0.582914	0.2767704	-1.07459357	2.96E-20	1.29E-19
AC124852.1	0.341303	0.0950178	-1.84478363	5.50E-27	4.67E-26
AC020978.4	0.763127	0.1903947	-2.00292996	5.80E-36	1.47E-34
AL031320.2	0.087116	0.1808135	1.053488135	4.20E-09	6.85E-09
AC007342.4	1.316498	0.1735468	-2.92330849	9.65E-37	3.09E-35
AC027228.1	0.011362	0.1209522	3.412090706	9.41E-09	1.49E-08
AC025576.2	0.018176	0.1201341	2.724560516	5.23E-09	8.44E-09
LINC00989	0.26825	0.6748653	1.331021367	5.72E-14	1.41E-13
AC026771.1	0.052694	0.1521231	1.529520965	7.94E-08	1.18E-07
LINC02747	0.151633	11.753542	6.276364246	6.46E-31	7.99E-30
AC010201.2	0.34136	1.0241455	1.585055524	1.02E-12	2.24E-12
AC112715.1	0.016286	0.137784	3.080681434	3.56E-10	6.32E-10

AL450384.2	0.302826	1.0526237	1.7974268	2.02E-20	8.98E-20
Z83843.1	0.546551	1.6092966	1.558003366	1.02E-12	2.24E-12
AC232271.1	0.332768	0.9066358	1.44600629	4.86E-14	1.20E-13
LINC01786	0.196841	1.0398644	2.40129068	1.06E-18	4.11E-18
GABPB1-AS1	0.459668	1.1762027	1.355473471	2.55E-09	4.25E-09
AC091057.3	0.044417	0.1299861	1.549180611	3.23E-08	4.94E-08
SNHG3	1.434007	2.8743193	1.003168247	1.78E-08	2.76E-08
AC026412.3	0.159989	0.5012501	1.647556287	6.40E-25	4.36E-24
AC007637.1	2.486031	1.1129964	-1.1593954	1.88E-21	9.18E-21
AL121987.2	0.068106	0.2347779	1.785439457	9.19E-22	4.67E-21
AC009120.3	0.490427	1.3948183	1.507967136	1.20E-12	2.61E-12
AL136115.1	0.055235	0.1758374	1.670579804	5.65E-05	6.94E-05
LINC02528	0.014541	0.2828967	4.28206208	1.82E-22	9.88E-22
LINC02544	0.078982	0.3347813	2.083627672	7.73E-13	1.73E-12
AC007292.3	0.036328	0.1806854	2.314315701	6.70E-10	1.16E-09
AC004908.2	0.510547	1.04339	1.031163008	6.00E-08	8.98E-08
AC007342.5	0.902257	0.1558438	-2.53343741	3.76E-36	9.85E-35
AC010976.2	0.32506	1.1171775	1.781078359	1.13E-07	1.65E-07
LINC01014	0.576123	0.1661499	-1.79389206	7.05E-25	4.79E-24
AC010333.1	0.122505	0.2948617	1.267194888	0.025695602	0.026146

AC119396.1	0.191576	0.4743039	1.30789828	4.11E-07	5.79E-07
LINC02012	0.415989	0.0895098	-2.21642758	3.75E-34	6.97E-33
AC104564.1	0.114588	0.3409778	1.573215724	7.54E-10	1.30E-09
AC006441.4	1.586704	0.082427	-4.26677216	1.17E-14	3.06E-14
AL590764.1	0.15393	0.7534717	2.2912772	3.26E-31	4.12E-30
SLC25A5-AS1	1.90629	0.4655861	-2.03364778	7.05E-37	2.36E-35
LINC00871	0.639447	0.0466929	-3.77555137	8.52E-33	1.31E-31
LINC01150	0.228777	1.1519394	2.332053248	1.24E-29	1.39E-28
SPATA13-AS1	0.014917	0.1191085	2.997275401	2.42E-07	3.46E-07
AC007991.4	0.011749	0.3844236	5.0321197	4.48E-29	4.77E-28
AL109936.2	0.824016	1.7756483	1.10760111	5.19E-12	1.08E-11
AC007038.1	0.503582	1.4099641	1.485360658	5.12E-16	1.49E-15
GRK5-IT1	0.045153	0.1824484	2.014591875	0.005970451	0.006409
AC010307.4	0.716867	0.149844	-2.25824395	2.29E-32	3.32E-31
AC100778.2	0.152945	0.4233248	1.468748408	1.10E-10	2.04E-10
AC004921.1	0.13765	0.7042399	2.355066514	2.66E-34	5.02E-33
AP003471.1	0.0475	0.1494722	1.653888867	2.32E-09	3.89E-09
AC093797.1	0.130757	0.4366112	1.739464049	2.20E-17	7.46E-17
MYCBP2-AS2	0.038175	0.1364904	1.838111292	0.003560126	0.003878
AC005281.1	1.762453	0.2623594	-2.74796886	8.05E-35	1.71E-33

HOXB-AS3	5.586956	0.9409673	-2.56984612	6.02E-36	1.51E-34
SSBP3-AS1	0.110795	0.2579855	1.219391029	2.55E-10	4.60E-10
AP001972.4	0.011158	0.143188	3.681758528	1.13E-11	2.27E-11
AC007743.1	0.341308	0.95531	1.484896751	3.06E-16	9.13E-16
AC020911.1	0.066274	0.1612105	1.282433852	1.65E-07	2.38E-07
AL138689.1	0.114618	0.3586026	1.645547555	4.00E-08	6.07E-08
AL163051.1	0.564559	0.2440772	-1.20978675	4.06E-24	2.57E-23
AC123768.1	0.096442	0.2644014	1.455000807	7.14E-14	1.74E-13
AC004253.1	0.148081	0.8312917	2.488964014	5.64E-22	2.94E-21
CARNMT1-AS1	0.085444	0.175896	1.041674515	0.032447145	0.032851
AC080162.1	0.252462	0.6074921	1.26680023	1.01E-11	2.04E-11
AC093001.1	0.011144	0.9659695	6.437666202	2.11E-10	3.84E-10
AC009509.4	0.625893	1.3696034	1.129770094	3.24E-15	8.97E-15
AL356299.2	0.079288	0.2784882	1.812439895	1.63E-12	3.51E-12
AP003498.1	0.051974	0.1634997	1.653422867	7.14E-07	9.92E-07
NRIR	0.057481	0.3212698	2.482618048	1.20E-20	5.48E-20
AC087683.2	0.179873	0.3674024	1.030379568	0.002219625	0.002461
ZNF346-IT1	0.137309	0.3624305	1.400277323	4.18E-08	6.34E-08
AC007216.4	0.146963	0.4099948	1.480152899	7.05E-14	1.72E-13
AC104667.2	1.149316	0.4009368	-1.51932912	1.63E-26	1.30E-25

AC137932.3	0.086061	0.3846045	2.159939014	8.46E-22	4.33E-21
LHFPL3-AS2	8.787694	3.6688625	-1.26015184	1.08E-23	6.56E-23
AC005332.2	0.068233	0.2035805	1.577060328	3.79E-18	1.40E-17
LINC02532	9.27065	19.121209	1.044431369	1.07E-15	3.06E-15
AL162725.2	0.83921	0.0257877	-5.0242788	1.15E-28	1.16E-27
AC005104.1	0.290904	1.2309542	2.081161976	1.63E-19	6.69E-19
LINC02041	0.352964	4.1619788	3.55967475	2.33E-29	2.54E-28
AL117209.1	0.194589	0.6088993	1.645770104	3.34E-11	6.42E-11
MIR3659HG	0.044445	0.1364285	1.618039092	2.68E-10	4.81E-10
AL031733.2	0.019916	0.1338402	2.748507109	2.56E-22	1.37E-21
AL035530.1	0.028797	0.1433181	2.315228415	5.14E-05	6.33E-05
AL132989.1	0.684699	1.9313721	1.496083554	3.45E-13	7.94E-13
AP001269.2	0.094897	0.2092746	1.140956332	8.41E-06	1.09E-05
AC130469.1	0.057492	0.584919	3.346803571	2.14E-19	8.70E-19
AP001619.1	0.054498	0.1207701	1.147976227	0.000160352	0.000192
LINC01266	0.327627	0.1326509	-1.30441983	1.96E-26	1.55E-25
AC005899.6	0.32995	1.1537522	1.806016266	2.64E-15	7.35E-15
SUCLG2-AS1	1.044463	0.3561458	-1.55222102	4.52E-34	8.29E-33
AC011379.1	0.02855	0.1271589	2.15508959	0.001229819	0.00139
AC018761.3	0.048591	0.1830219	1.913249317	5.49E-08	8.24E-08

AC016957.2	0.274332	0.8876587	1.694081638	9.53E-29	9.76E-28
AC148477.4	5.974009	0.8036776	-2.89401067	2.37E-34	4.55E-33
AC093535.1	0.209319	0.5609246	1.422105756	3.46E-14	8.71E-14
Z98884.2	0.493667	1.243942	1.333309659	1.53E-21	7.55E-21
AC008760.1	0.424249	0.8677897	1.032432638	7.19E-07	9.98E-07
AC004771.1	0.063788	0.2016105	1.660214224	1.65E-10	3.02E-10
PLA2G4C-AS1	0.027953	0.1682872	2.589868017	1.11E-22	6.15E-22
LNX1-AS2	0.329619	0.0908826	-1.8587247	5.57E-25	3.84E-24
DNAJB5-DT	0.079374	0.278804	1.812512	6.24E-12	1.29E-11
AC010997.2	0.04822	0.1107454	1.199556506	0.000147112	0.000177
AP001021.2	0.089277	0.2561224	1.520475938	1.79E-08	2.78E-08
AL157871.5	0.134548	0.3443129	1.355600857	1.22E-07	1.78E-07
AL031123.1	10.58834	0.9061741	-3.54654487	1.09E-39	6.46E-38
AC083841.1	0.457586	0.0969643	-2.23851737	3.82E-16	1.13E-15
ARHGAP27P1-BPTFP1-KPNA2P3	0.317314	1.367695	2.107762859	9.89E-23	5.49E-22
AL360181.1	0.225065	0.5998714	1.414310096	1.05E-06	1.45E-06
AC244093.5	0.034705	0.1574041	2.181274303	6.34E-18	2.28E-17
AC026495.1	0.340591	0.0832465	-2.03258007	1.85E-05	2.34E-05
WASHC5-AS1	0.111217	0.2859716	1.362499052	2.05E-11	4.01E-11
MID1IP1-AS1	2.16467	0.8286851	-1.38525107	1.48E-30	1.78E-29

LIF-AS1	0.015509	0.1274318	3.038594373	9.61E-09	1.52E-08
COLCA1	3.252071	1.0552545	-1.62376764	7.27E-28	6.76E-27
AL662884.1	0.034128	0.174126	2.351118573	2.54E-17	8.54E-17
AC018766.1	0.122397	0.2951185	1.269726826	7.59E-05	9.27E-05
AC010973.2	0.170749	0.7750834	2.18247336	4.60E-24	2.89E-23
AP002812.3	0.037168	0.1916614	2.366442464	2.60E-15	7.24E-15
AL357874.1	0.06758	0.2286624	1.758548419	2.96E-18	1.11E-17
AL354893.2	0.051581	0.1371336	1.4106815	1.30E-06	1.78E-06
AC020915.1	0.373869	0.9579789	1.357460967	2.89E-11	5.57E-11
AL356740.1	0.896658	0.1679948	-2.4161411	3.00E-36	8.01E-35
AC008147.1	0.072067	0.2064059	1.518081692	0.001132503	0.001284
AC253536.3	0.402784	0.8440684	1.067354586	0.000392266	0.000458
AC104964.3	0.191484	0.8183046	2.095412122	2.36E-17	7.95E-17
NCBP2-AS1	0.067544	0.2347962	1.797507636	5.07E-14	1.25E-13
MROCKI	0.417986	2.241826	2.423148982	9.01E-29	9.27E-28
AL118556.1	0.317884	0.1566541	-1.02091744	1.43E-19	5.93E-19
AL031846.2	0.165473	0.6098767	1.881925646	2.26E-19	9.16E-19
AC025171.4	0.167366	0.9802199	2.550103042	2.23E-27	1.97E-26
AL133371.2	0.693637	1.8223814	1.393573168	1.24E-17	4.29E-17
LINC01569	1.178833	2.3640112	1.003877437	5.50E-15	1.48E-14

LINC00472	4.81864	1.3292535	-1.85800964	2.14E-35	4.78E-34
AL359921.1	0.201957	0.5518249	1.450158897	2.66E-17	8.93E-17
AL121929.2	0.063449	0.1976228	1.639087688	1.43E-07	2.07E-07
WT1-AS	0.39422	0.0722961	-2.44701063	2.56E-28	2.45E-27
HCFC1-AS1	0.065302	0.1684038	1.36671904	2.92E-08	4.48E-08
AC011005.4	0.158056	0.4385135	1.472185729	1.38E-07	2.00E-07
AC048341.2	0.786541	2.5226267	1.681332982	1.06E-11	2.13E-11
AC009148.1	0.032591	0.1303314	1.999632594	2.06E-13	4.85E-13
AL022311.1	0.054954	0.1405374	1.354649189	3.86E-18	1.42E-17
AL354809.1	0.054962	0.1435362	1.384917503	1.96E-14	5.03E-14
AC015660.3	0.089216	0.267706	1.585281614	6.92E-08	1.03E-07
LINC00511	0.158663	0.411296	1.374210526	1.02E-07	1.50E-07
AC013724.1	0.017703	0.1820538	3.362326805	3.54E-24	2.25E-23
AP000350.6	0.104888	0.2167182	1.046973464	0.001511412	0.001699
PGM5-AS1	0.80392	0.2911787	-1.46514802	2.52E-22	1.35E-21
AL139246.3	0.304543	0.6785553	1.155821416	1.64E-10	3.00E-10
AL354892.3	0.166187	0.372114	1.162941367	3.18E-09	5.23E-09
AC104316.2	0.035199	0.5133543	3.866361184	6.29E-28	5.92E-27
AP003392.1	0.517678	1.7596502	1.765160747	2.38E-14	6.06E-14
AC005154.4	0.110776	0.4193017	1.920348458	1.74E-16	5.33E-16

MIR34AHG	0.087719	0.2762892	1.655210607	3.29E-21	1.57E-20
AL512770.1	0.08684	0.3747463	2.109490387	1.06E-21	5.33E-21
AL358075.1	0.040664	0.1970879	2.277027726	3.38E-09	5.56E-09
AC010245.2	0.416246	0.9367254	1.170188229	3.39E-23	1.97E-22
PLS3-AS1	0.710488	0.1107892	-2.68099187	4.88E-38	1.95E-36
AC078906.1	0.016379	0.341561	4.382241873	5.99E-32	8.12E-31
AC009119.2	0.062631	0.3148963	2.329917942	1.53E-16	4.69E-16
AC245014.3	0.1307	0.3099881	1.245950685	2.20E-06	2.97E-06
AC096642.1	0.071197	0.3100149	2.122456862	6.13E-20	2.60E-19
AC100793.2	0.035987	0.1392014	1.951638149	1.01E-05	1.30E-05
AL031670.1	0.274297	0.7104352	1.37296499	1.41E-11	2.79E-11
AL122125.1	0.05373	0.3987305	2.891625423	1.22E-22	6.73E-22
LINC02432	1.300242	0.1807467	-2.84673887	4.60E-34	8.37E-33
AC008105.2	0.051766	0.8257179	3.995576939	4.20E-33	6.88E-32
AC005616.1	1.283192	0.0231641	-5.79170488	6.70E-26	5.02E-25
AC004832.6	0.021249	0.2144271	3.335017926	1.63E-26	1.30E-25
GTF3C2-AS1	0.03134	0.1264902	2.012948516	1.86E-14	4.77E-14
AL024508.1	0.603268	0.1764536	-1.77350983	9.03E-35	1.87E-33
AC005387.1	0.085842	0.450949	2.393208241	1.84E-18	6.98E-18
ZNF793-AS1	1.437711	0.6513474	-1.14227456	9.76E-29	9.95E-28

MIR200CHG	2.760587	0.4764736	-2.53450684	1.27E-41	1.07E-39
STARD4-AS1	0.414539	1.4781269	1.834191181	3.24E-24	2.07E-23
MRTFA-AS1	0.015996	0.1334654	3.06070773	1.24E-14	3.23E-14
AC006111.3	0.076031	0.2026315	1.414199724	0.007874242	0.008355
AC005306.1	0.060827	0.3605728	2.567508101	2.68E-18	1.01E-17
AC091887.1	0.039847	0.2393763	2.586738989	5.46E-23	3.10E-22
AC022973.5	0.086002	0.2444648	1.507188922	1.02E-11	2.05E-11
MECOM-AS1	0.65666	0.1473812	-2.15559278	1.07E-37	4.07E-36
LINC01780	0.850136	0.2441539	-1.79990243	2.65E-19	1.07E-18
AC012404.1	0.017146	0.4991504	4.863492381	1.40E-08	2.19E-08
MRPS9-AS1	0.156212	0.3992217	1.353688919	6.00E-12	1.24E-11
SAP30L-AS1	0.154535	0.3614505	1.225864654	5.90E-11	1.12E-10
AP000873.3	0.131117	0.5064921	1.949679772	4.73E-18	1.72E-17
Z98885.3	0.039882	0.1552902	1.961144165	6.00E-15	1.61E-14
RNF216-IT1	0.026168	0.1845897	2.818426588	1.61E-11	3.18E-11
AL391261.1	0.365092	0.1153254	-1.66255063	6.88E-11	1.30E-10
AC117490.2	0.088641	0.1915852	1.111942607	4.62E-05	5.70E-05
AC064801.1	0.731524	0.2763365	-1.40447874	6.00E-24	3.75E-23
AC090907.2	0.048422	0.1608422	1.731904679	2.33E-16	7.04E-16
AP000763.3	0.034723	0.2454922	2.821708289	7.35E-18	2.62E-17

LINC02812	0.03749	0.1267516	1.757442656	5.53E-09	8.90E-09
AL357497.1	0.025087	0.1625939	2.696254059	1.43E-12	3.10E-12
EDIL3-DT	0.342326	0.7994735	1.223680218	1.53E-06	2.08E-06
AC053513.2	0.216829	0.652253	1.588871896	1.20E-12	2.62E-12
DOCK8-AS1	7.224884	3.4520724	-1.06551166	0.006028647	0.006466
AL353622.1	0.656716	1.3663408	1.056975485	0.000336192	0.000395
AC012366.1	0.322841	0.1440295	-1.16446006	1.83E-14	4.72E-14
AC136475.3	0.856646	13.489178	3.976959486	5.75E-18	2.07E-17
AL158834.2	0.063919	0.2808665	2.135572554	4.32E-22	2.27E-21
AC016590.2	0.192845	0.5110994	1.406161557	1.75E-06	2.37E-06
AC015845.2	1.708304	0.8361629	-1.0307089	1.83E-31	2.37E-30
AL050327.1	0.234495	0.0965186	-1.28067508	5.42E-28	5.12E-27
AC010247.2	0.075203	0.2443919	1.700325763	2.77E-24	1.78E-23
AC091057.1	0.079307	0.2974232	1.907004825	4.39E-33	7.13E-32
AL391056.1	0.074862	0.3145053	2.070778189	1.59E-10	2.91E-10
AL590822.1	0.088636	0.7686049	3.11627426	3.93E-12	8.23E-12
LINC00216	0.123241	0.2948062	1.258288944	0.003633931	0.003957
AC025043.1	0.060719	0.202106	1.734901947	2.27E-13	5.32E-13
AC004870.4	0.575458	0.063893	-3.17098244	3.79E-43	4.01E-41
Z69666.1	0.033659	0.1822492	2.436866987	2.77E-15	7.68E-15

AC003070.1	0.283999	1.3904841	2.291631363	2.55E-23	1.50E-22
AP001094.2	0.090902	0.6436571	2.823910085	2.00E-32	2.94E-31
LINC00427	0.026243	0.1322591	2.333355616	1.65E-10	3.02E-10
AC148477.3	0.3359	0.0963604	-1.80152047	5.78E-27	4.89E-26
AC106795.2	0.507669	0.1801536	-1.49466118	1.29E-11	2.57E-11
AC024257.3	0.057168	0.122996	1.105322601	0.00143792	0.001618
AC007292.1	0.441183	0.9971495	1.176430995	2.88E-13	6.67E-13
FLNB-AS1	0.17571	0.4082509	1.216262203	9.90E-07	1.36E-06
AC011481.1	0.207535	0.7763332	1.903323906	2.38E-11	4.63E-11
AC026333.4	0.077934	0.25753	1.724416971	7.47E-15	1.98E-14
AL162274.1	0.05967	0.4558717	2.933558609	7.55E-30	8.60E-29
LINC00115	0.300086	0.7421117	1.306260229	3.84E-10	6.79E-10
AC073912.1	0.053622	0.23055	2.104177423	1.20E-12	2.61E-12
AL513320.1	0.326675	0.9444547	1.531627626	4.18E-06	5.52E-06
SAMD12-AS1	0.321015	0.141294	-1.18393906	4.07E-23	2.34E-22
AL359397.1	1.086089	0.3429911	-1.66289899	5.40E-22	2.82E-21
AL009178.2	0.554803	0.2426907	-1.19285617	1.81E-25	1.30E-24
AL607028.1	0.488225	0.1199994	-2.02451771	1.32E-29	1.48E-28
AC020779.2	1.299146	0.3131162	-2.05279315	5.32E-27	4.53E-26
LINC00299	0.009167	0.1687565	4.202376694	5.08E-37	1.77E-35

AC025430.1	0.080782	0.2388646	1.564095421	2.87E-09	4.76E-09
AC009226.1	0.042835	0.1234516	1.527090479	2.69E-16	8.07E-16
AC112496.1	0.192408	0.5804406	1.592979941	1.88E-11	3.69E-11
MRPL20-DT	0.419782	1.0923502	1.379723225	9.00E-22	4.58E-21
AC007216.3	0.062909	0.2939953	2.224453923	1.83E-16	5.59E-16
AC118344.1	0.153617	0.3073501	1.000545009	0.000155205	0.000186
UBAC2-AS1	0.894846	0.3889744	-1.2019637	1.83E-31	2.37E-30
LINC00937	0.047729	0.1141478	1.257967497	1.83E-09	3.07E-09
LINC01355	0.246017	1.1261337	2.194550435	1.08E-20	4.92E-20
AL031710.1	10.23424	1.6976578	-2.59178596	5.68E-33	9.04E-32
AC068987.2	0.112252	0.266406	1.246883712	3.92E-06	5.19E-06
AC103810.5	0.067524	0.1843597	1.44905709	6.60E-05	8.08E-05
AC022167.2	0.263507	0.6599885	1.324599634	3.27E-26	2.53E-25
AC105105.4	0.004283	0.1165594	4.766448521	4.26E-24	2.69E-23
LINC02585	0.269199	0.702718	1.384273217	8.69E-11	1.62E-10
AC002064.2	0.292878	0.8622095	1.557738462	8.61E-09	1.37E-08
AL035071.2	0.128561	0.2689395	1.064825682	0.002466291	0.002724
LINC01843	0.734497	2.9352872	1.998672442	5.23E-23	3.00E-22
AC027796.1	0.080156	0.2306508	1.524832402	1.59E-05	2.01E-05
LINC02453	0.537581	0.2368778	-1.18233915	1.74E-27	1.55E-26

GAS6-DT	2.265057	0.9002566	-1.33113883	8.73E-25	5.88E-24
AC133552.5	3.985295	1.8055994	-1.14220866	1.85E-25	1.32E-24
AC124854.1	1.089441	5.830667	2.420073464	5.35E-12	1.11E-11
ZNF114-AS1	0.013468	0.1624524	3.5924214	1.59E-08	2.47E-08
AC024941.2	0.628825	0.2814626	-1.15971636	4.22E-21	1.99E-20
AL592301.1	0.048523	0.1588504	1.710933162	3.81E-07	5.38E-07
LINC00924	6.000021	2.2159173	-1.43706354	6.24E-22	3.23E-21
AC066613.1	0.195762	0.5978878	1.610775067	3.26E-17	1.08E-16
AL353804.2	0.036085	0.2969917	3.040965532	2.15E-16	6.53E-16
AC010333.2	0.091908	0.2282738	1.312508611	0.015290585	0.015836
AC025171.2	0.447332	1.2457298	1.477573643	4.99E-26	3.82E-25
FAM198B-AS1	0.162115	0.3577866	1.142078232	3.01E-10	5.38E-10
LINC02427	0.454838	0.1725568	-1.39828108	2.88E-16	8.61E-16
AC025871.2	0.086559	0.195968	1.178859334	6.56E-05	8.04E-05
AP001020.2	0.032547	0.121128	1.895932314	1.89E-12	4.05E-12
ACBD3-AS1	0.139406	0.4209009	1.594185619	5.49E-15	1.48E-14
AC008735.1	0.061577	0.3716715	2.593554069	1.34E-18	5.14E-18
AL606534.1	0.058092	0.2259376	1.959513447	7.38E-18	2.62E-17
AL450384.1	0.08107	0.3499336	2.109848854	3.21E-15	8.88E-15
AC025918.1	0.048265	0.1426309	1.56322711	1.52E-05	1.93E-05

XXYLT1-AS2	0.062973	0.1865922	1.567080598	9.47E-09	1.50E-08
AC004584.1	0.03865	0.2080543	2.428425272	1.18E-14	3.09E-14
AL135787.1	0.074772	0.1575174	1.074938984	0.008178483	0.008665
OCIAD1-AS1	0.353832	0.8800555	1.314528312	5.72E-15	1.53E-14
AC073912.2	0.050981	0.2773274	2.443567859	1.59E-13	3.75E-13
DLEU2L	0.071455	0.1831356	1.357805284	1.22E-10	2.26E-10
AC016831.4	0.019026	0.3614435	4.247752188	3.09E-06	4.11E-06
AC020658.5	0.136236	0.4216229	1.629849012	8.67E-17	2.75E-16
Z94721.1	0.119882	0.8669265	2.85429131	7.31E-29	7.58E-28
PSMD6-AS2	0.151898	0.3106828	1.032342307	6.43E-06	8.41E-06
AL589745.1	0.527171	1.3998298	1.408909644	2.68E-09	4.47E-09
AC008737.1	0.105067	0.5328596	2.342446314	6.62E-16	1.91E-15
AC126773.3	0.049273	0.2373962	2.268424268	2.93E-08	4.49E-08
AC002091.1	0.230789	0.802367	1.797686525	4.34E-25	3.02E-24
AC087482.1	3.536526	14.458272	2.031490313	1.04E-21	5.25E-21
AP001107.4	0.200963	0.7623557	1.923533699	5.62E-13	1.27E-12
AC027544.2	0.048217	0.1543118	1.678241267	6.38E-07	8.87E-07
AC008050.1	0.076284	0.3044156	1.996596505	4.99E-15	1.35E-14
AC134775.1	0.054695	0.1640333	1.584501144	2.16E-06	2.91E-06
AC078842.1	0.011426	0.1209638	3.404223838	1.76E-09	2.96E-09

AC116407.1	0.612525	0.223749	-1.45288765	3.85E-27	3.34E-26
XIAP-AS1	0.010923	0.1210685	3.470443328	9.60E-08	1.41E-07
FAM13A-AS1	0.216505	1.2417919	2.519948539	1.42E-29	1.57E-28
AC007336.1	0.058289	0.1343513	1.2047214	0.036989599	0.037265
AC008735.2	0.609316	3.2372101	2.409489428	7.61E-20	3.21E-19
AC022558.3	0.118206	0.3837566	1.698885953	3.16E-16	9.43E-16
AC087239.1	0.123115	0.4288982	1.800627315	6.87E-14	1.68E-13
ITGB5-AS1	0.033017	0.1138959	1.786434824	9.79E-06	1.26E-05
AC007014.2	0.085437	0.2401647	1.491097069	0.001873956	0.002092
LINC02762	0.432109	2.4466324	2.50132992	2.13E-33	3.56E-32
GK-AS1	0.188141	0.6392103	1.764475086	2.78E-14	7.04E-14
AC020900.1	0.029425	0.1569944	2.41558355	5.51E-11	1.05E-10
LINC01389	0.156305	0.5226152	1.741381137	4.61E-16	1.35E-15
AL512791.1	0.665798	1.9159161	1.52487838	3.98E-13	9.08E-13
ZFHX2-AS1	0.067245	0.2554848	1.925744353	4.35E-16	1.28E-15
PITRM1-AS1	0.087118	0.3609925	2.050930206	2.19E-34	4.34E-33
HEXA-AS1	0.307399	0.1507878	-1.02759308	4.70E-12	9.76E-12
AC087222.1	0.103054	0.6591914	2.677294261	1.79E-21	8.82E-21
AC036108.2	0.026728	0.1556946	2.542297429	8.05E-17	2.56E-16
AC005180.2	0.283016	0.5719108	1.014908771	8.13E-08	1.20E-07

AP001610.2	0.052368	0.11542	1.140127236	0.000290389	0.000343
GAR1-DT	0.29075	0.1304367	-1.15643053	6.43E-16	1.86E-15
AC068580.3	0.83055	1.8484688	1.154192231	4.64E-16	1.36E-15
AC036108.3	0.124982	0.2780683	1.153714743	4.21E-18	1.55E-17
AC005089.1	0.038032	0.1212184	1.672324876	3.89E-07	5.49E-07
AC023510.2	0.389697	0.822939	1.078433692	2.93E-09	4.84E-09
FOXC2-AS1	0.099379	0.7241795	2.865337776	9.22E-13	2.05E-12
RUNX3-AS1	0.010271	0.1414347	3.783530793	1.25E-26	1.02E-25
USP30-AS1	0.394299	2.0726222	2.394093688	1.15E-28	1.16E-27
AC007494.2	0.014971	0.1180696	2.979425776	7.26E-07	1.01E-06
HNF4A-AS1	2.914971	0.1443293	-4.3360455	2.24E-20	9.90E-20
C1orf195	0.079158	0.4057137	2.357652935	0.000103589	0.000125
AC079907.1	0.148504	0.4614244	1.635591769	3.38E-16	1.01E-15
AC007566.1	0.641806	2.0954509	1.707051033	6.93E-18	2.48E-17
AC139100.2	0.276793	0.9894776	1.837861649	5.48E-17	1.77E-16
AC138207.1	0.03083	0.1941205	2.654550407	3.96E-23	2.28E-22
LINC00482	0.27033	0.674346	1.31876626	0.000133698	0.000161
AC138207.4	0.656098	1.428084	1.122096807	3.91E-17	1.28E-16
AC008957.1	0.088073	0.3274787	1.894634857	4.74E-17	1.54E-16
AC022613.3	0.035438	0.1376931	1.958099251	2.28E-09	3.82E-09

AC024022.1	1.229373	0.0482313	-4.67180982	1.12E-44	1.25E-42
AC012020.1	0.038838	0.1215269	1.645739399	7.37E-14	1.80E-13
ITGB2-AS1	0.223036	1.6652707	2.900407133	2.01E-29	2.20E-28
AP000873.1	0.325321	0.664424	1.030240111	6.83E-12	1.40E-11
ARMC2-AS1	0.128188	0.3284766	1.357531081	9.19E-06	1.19E-05
AC093788.1	0.138316	0.58497	2.080399152	3.62E-19	1.45E-18
AC074194.1	0.066666	0.1846762	1.469970645	3.61E-09	5.92E-09
AC000120.1	0.06098	0.3138255	2.363550397	5.11E-17	1.66E-16
AL358115.1	0.038182	0.1404905	1.879505685	3.95E-15	1.08E-14
AC058791.1	0.041256	0.4293428	3.379445378	0.000454747	0.000529
SNHG20	1.119142	2.6134299	1.223551863	3.19E-25	2.24E-24
AC006480.2	0.235591	0.6253989	1.408495004	7.04E-09	1.13E-08
AC021078.1	0.830893	2.9498583	1.827911397	9.41E-28	8.59E-27
HCP5	9.270478	22.609893	1.286238478	6.51E-28	6.11E-27
AC010680.4	0.109835	0.3282688	1.579542941	1.29E-10	2.38E-10
RABGAP1L-DT	0.229666	0.0984757	-1.22169787	1.98E-22	1.07E-21
F11-AS1	2.071853	0.1084195	-4.2562263	1.54E-38	6.81E-37
AC011726.2	0.127549	0.2880077	1.175053952	3.15E-06	4.19E-06
AC120498.3	0.066387	0.3524396	2.40839377	2.15E-05	2.71E-05
MYG1-AS1	0.30304	1.4393744	2.247862162	1.96E-35	4.46E-34

AL353152.1	2.991248	0.1704737	-4.1331261	2.50E-39	1.35E-37
ZBTB40-IT1	0.11062	0.3689596	1.737853822	8.30E-13	1.85E-12
AC007336.2	0.006159	0.2084706	5.081017148	7.90E-24	4.85E-23
AC139887.1	0.426193	0.9573185	1.167493282	3.46E-10	6.15E-10
AC009053.3	1.313678	3.6138149	1.459910691	1.67E-11	3.29E-11
AC105020.2	0.020862	0.116985	2.487374427	2.38E-07	3.41E-07
AC087623.1	0.184244	0.6700138	1.862569606	1.27E-12	2.76E-12
LINC00939	0.042956	0.1640899	1.933560203	1.31E-12	2.84E-12
AC092809.2	0.144748	0.3532545	1.28716557	4.43E-10	7.79E-10
AP000692.1	0.192447	0.7162327	1.895969287	2.00E-18	7.58E-18
MAP3K5-AS1	0.128652	0.2892492	1.168840808	4.34E-06	5.72E-06
AC020917.2	0.009041	0.119145	3.720138015	7.61E-12	1.56E-11
AL358334.3	0.012758	0.1636711	3.681304477	1.99E-23	1.19E-22
AC003984.1	0.236369	1.190646	2.332632942	2.89E-11	5.57E-11
AL031709.1	0.140393	0.5403656	1.944465722	5.01E-15	1.36E-14
SPIN4-AS1	0.032408	0.1178705	1.86276598	2.59E-06	3.48E-06
AL353751.1	2.1768	0.9785511	-1.15348985	4.62E-19	1.82E-18
AC104109.4	0.053793	0.1472482	1.452756909	3.02E-11	5.81E-11
ZNF710-AS1	14.87384	5.8648206	-1.34261783	6.37E-24	3.96E-23
LINC02487	0.073159	0.2429015	1.731273966	6.11E-21	2.83E-20

AC116348.1	0.115887	0.4977464	2.102696189	0.001806322	0.002017
F10-AS1	0.2324	0.1073059	-1.11488244	1.65E-27	1.48E-26
ERVK9-11	0.705372	2.3693006	1.748004825	8.26E-17	2.62E-16
AC080013.2	0.089548	0.2966402	1.727987556	6.32E-10	1.10E-09
AP003392.3	0.065708	0.1432839	1.124745348	1.65E-06	2.24E-06
SNHG17	1.621331	3.3534641	1.048473285	5.03E-21	2.36E-20
AC011389.1	0.047	0.2748702	2.548003375	1.73E-09	2.92E-09
AC004816.1	1.608547	0.5151327	-1.64274172	4.64E-34	8.38E-33
AC063965.2	0.111037	0.7058994	2.668422844	2.30E-17	7.78E-17
AC073367.1	0.071901	0.2117664	1.558380872	2.87E-08	4.41E-08
AC026992.2	1.618297	0.5678035	-1.51101298	8.47E-26	6.29E-25
U52111.1	0.035439	0.1789234	2.335933251	1.15E-21	5.73E-21
OGFR-AS1	0.148352	0.3854622	1.377566786	3.70E-15	1.01E-14
AC079848.1	1.393803	0.3901185	-1.83704226	3.51E-35	7.71E-34
LINC01934	0.028407	0.2098864	2.885286754	6.79E-23	3.80E-22
ATP2C2-AS1	0.04127	0.1151333	1.480133003	2.10E-09	3.52E-09
AC084018.1	0.995772	2.8275342	1.505656712	2.43E-09	4.05E-09
AL049629.1	2.086014	0.4067843	-2.35841323	2.06E-33	3.46E-32
AL031658.1	0.081079	0.2910219	1.843731899	1.25E-13	2.99E-13
AC026979.1	0.029582	0.1224137	2.048949646	3.72E-10	6.58E-10

ARHGAP26-AS1	0.01325	0.1807621	3.770046461	1.34E-20	6.04E-20
AGAP11	0.039721	0.1870495	2.235429124	4.46E-16	1.31E-15
AL390294.1	0.681516	0.0231488	-4.87973813	6.85E-78	1.67E-74
AC010999.1	0.081503	0.2559473	1.650924608	2.02E-13	4.74E-13
AL035420.3	0.613909	0.2527729	-1.28018391	1.17E-20	5.35E-20
AC011815.2	0.058864	0.187196	1.669090658	5.54E-12	1.14E-11
AL355922.1	0.079088	0.1969944	1.316631473	1.58E-09	2.68E-09
AL162171.1	2.443813	0.767423	-1.67103993	9.84E-29	9.99E-28
AC005261.3	1.385184	3.062945	1.144841391	7.04E-29	7.36E-28
LINC02019	0.149048	0.3566217	1.258612924	4.05E-17	1.33E-16
LINC02608	0.46624	0.1823719	-1.3541911	6.39E-23	3.59E-22
AC002553.2	0.371952	1.0355013	1.477142068	8.72E-14	2.11E-13
AC020907.4	0.134378	1.0945452	3.025966707	1.54E-25	1.11E-24
AC068790.4	0.142137	0.4640439	1.706981613	4.53E-12	9.43E-12
INE1	0.721407	1.4986795	1.054806796	8.66E-11	1.62E-10
AC006441.1	0.071102	0.2444562	1.78161267	8.52E-10	1.46E-09
AL596325.2	0.317326	0.875754	1.46455887	8.41E-09	1.34E-08
AC008083.3	0.134397	0.3550637	1.401575373	2.27E-13	5.32E-13
AC016737.1	0.114971	0.3461301	1.590050102	4.40E-10	7.74E-10
AC005392.2	0.033902	0.124141	1.872543673	5.07E-07	7.10E-07

AC103810.2	0.07005	0.187691	1.421910915	6.73E-06	8.80E-06
U62317.1	1.890257	6.762733	1.83902388	1.62E-28	1.60E-27
AL121890.5	0.1058	0.2338567	1.144286473	1.51E-06	2.06E-06
AC027796.4	0.224249	1.0666274	2.249880365	1.74E-17	5.94E-17
AL157895.1	0.052018	0.1237874	1.250769006	4.12E-13	9.39E-13
AC010615.2	0.102957	0.2851072	1.469458752	1.84E-20	8.24E-20
AC093904.2	0.508899	0.0756005	-2.75091134	1.41E-52	3.82E-50
STAG3L5P-PVRIG2P-PILRB	0.259378	0.9894548	1.931579379	1.11E-18	4.32E-18
AC004492.1	0.445941	0.9184086	1.042282509	4.23E-12	8.85E-12
AL354989.1	0.067014	0.2189149	1.707835865	2.68E-11	5.20E-11
AC018529.2	0.090611	0.2112499	1.221186165	5.70E-10	9.93E-10
AC078777.1	0.05395	0.1764759	1.709768896	6.29E-13	1.41E-12
AL161935.1	0.041823	0.2856335	2.771811414	2.26E-24	1.46E-23
ZBTB20-AS5	0.006843	0.1187164	4.116728717	2.94E-08	4.51E-08
LINC02356	0.11466	0.233015	1.023065847	7.21E-06	9.40E-06
AP000919.3	0.113054	0.2681246	1.245891854	5.06E-12	1.05E-11
AC105105.1	0.036781	0.4055138	3.46270863	3.24E-26	2.51E-25
AP001010.1	0.038194	0.3065829	3.004842553	2.03E-24	1.32E-23
AL355432.1	0.061171	0.1755398	1.520881591	1.27E-06	1.74E-06
LINC01896	1.292712	0.279952	-2.20714966	1.22E-36	3.71E-35

AC132872.2	0.044853	0.1519352	1.760194969	1.43E-11	2.84E-11
AC073651.1	0.016418	0.4114366	4.647297375	4.39E-05	5.41E-05
AC024361.3	0.04583	0.3562378	2.95848249	5.51E-20	2.35E-19
AC120349.1	0.091198	0.4610798	2.337948953	2.76E-06	3.69E-06
AC138305.1	0.883565	0.3303007	-1.41955575	9.71E-32	1.29E-30
AC004000.1	0.040752	0.3312183	3.022851173	1.36E-16	4.21E-16
C3orf35	0.059996	0.1959458	1.707523371	1.06E-16	3.33E-16
AC005785.1	0.121053	0.610457	2.334250551	9.01E-29	9.27E-28
MMP25-AS1	0.315379	1.680745	2.413940449	8.09E-36	1.97E-34
SEMA3F-AS1	0.254401	0.5476931	1.106265731	2.61E-11	5.07E-11
AC092171.4	0.198324	0.7101688	1.840302991	3.82E-11	7.32E-11
AL033397.1	0.541005	0.2239701	-1.27233694	1.03E-08	1.63E-08
AC015849.3	0.466717	1.7109363	1.874165713	3.75E-20	1.62E-19
AC078864.1	1.264192	9.3010774	2.879181992	8.13E-27	6.76E-26
TMSB15B-AS1	0.050258	0.1930037	1.941199145	1.59E-25	1.14E-24
RERE-AS1	0.15892	0.4216853	1.407869632	4.29E-12	8.97E-12
AC021087.4	0.123777	1.8391055	3.893193326	1.72E-07	2.49E-07
AC245052.4	0.090431	0.2618742	1.533989773	4.43E-15	1.20E-14
GPC6-AS2	0.014395	0.1152765	3.001454156	1.39E-10	2.55E-10
AC025188.1	0.039277	0.1123923	1.516770731	1.41E-09	2.40E-09

AC018410.1	0.078972	0.3471416	2.136112314	6.72E-18	2.41E-17
L3MBTL4-AS1	0.056628	0.4122465	2.863918331	6.77E-38	2.66E-36
AL133243.1	0.041805	0.1370017	1.712451192	1.78E-07	2.58E-07
AL161668.4	1.775058	0.1037349	-4.09689249	4.24E-39	2.08E-37
AC002044.1	0.077712	0.5552333	2.836879322	4.49E-12	9.36E-12
AL645940.1	0.101614	0.3175357	1.643820696	1.13E-11	2.26E-11
AC084876.1	0.082949	0.5723143	2.786509562	1.30E-24	8.65E-24
AL161669.3	0.768424	2.1373905	1.475875513	5.44E-07	7.60E-07
SLBP-DT	0.107834	0.6779468	2.652364628	1.23E-20	5.56E-20
AC008731.1	0.129553	0.4240717	1.710766888	2.96E-10	5.29E-10
AC025031.3	0.04121	0.1956537	2.24723617	0.013223037	0.013753
AC087501.1	0.029671	0.1603807	2.434386626	1.68E-11	3.30E-11
AL445490.1	0.031834	0.117068	1.878722379	3.44E-12	7.24E-12
AC244093.4	0.046138	0.12025	1.381995299	1.04E-07	1.53E-07
AC108010.1	0.899406	1.8248846	1.02076062	4.00E-10	7.05E-10
AL360182.2	0.007236	0.175289	4.598400899	1.48E-25	1.07E-24
AC087276.1	0.195805	0.8331663	2.089187201	2.06E-16	6.25E-16
NAV2-AS3	0.451975	0.0847807	-2.41443423	2.48E-38	1.06E-36
UBOX5-AS1	0.050899	0.1830019	1.846162356	5.40E-16	1.57E-15
LINC02804	0.069057	0.3067942	2.151404602	1.14E-16	3.57E-16

AC009690.2	0.175133	0.4758164	1.441954039	4.45E-18	1.63E-17
KCNQ1OT1	0.072325	0.1992395	1.46193733	0.000109148	0.000132
AC115102.1	0.032895	0.1210311	1.879456798	5.61E-07	7.83E-07
AC023389.1	0.060652	0.2070789	1.771548318	2.56E-06	3.44E-06
AC008569.2	0.056333	0.2510629	2.156008769	1.51E-19	6.23E-19
AC124248.1	0.270992	0.1189991	-1.18730153	3.93E-11	7.52E-11
HM13-IT1	0.271599	1.359948	2.324002186	6.03E-26	4.57E-25
LINC02303	2.518065	0.6428118	-1.96984727	1.08E-36	3.38E-35
AC020663.3	0.035596	0.1462775	2.038931543	4.46E-09	7.24E-09
AC118755.1	0.035305	0.3408437	3.271176861	1.21E-05	1.54E-05
AC004951.1	0.077232	0.1734944	1.167614524	7.66E-12	1.57E-11
AL049795.1	0.071238	0.2141956	1.588203394	9.01E-12	1.83E-11
ENTPD3-AS1	2.16655	0.9901151	-1.12973164	5.77E-33	9.08E-32
FAF1-AS1	0.019628	0.1632712	3.056259524	7.89E-07	1.09E-06
MDS2	0.031174	0.1505304	2.271636369	1.42E-28	1.41E-27
LINC01929	0.041636	0.4866201	3.546877398	3.06E-27	2.70E-26
AL137779.1	0.069526	0.1803775	1.375403886	0.00018296	0.000219
AC087392.3	0.049367	0.1440452	1.544915222	1.43E-11	2.84E-11
AC011462.3	0.0063	0.2491448	5.305468081	3.67E-10	6.51E-10
MIR302CHG	0.079944	0.1708025	1.095262609	8.19E-08	1.21E-07

DCXR-DT	0.659761	0.0785793	-3.06972124	2.57E-34	4.90E-33
LINC00265	0.589547	1.2344607	1.066202486	1.47E-07	2.13E-07
AC022144.1	1.099056	5.979007	2.443641642	1.59E-28	1.57E-27
AC009041.3	0.198565	0.4318086	1.12077722	5.20E-16	1.51E-15
ANKRD10-IT1	2.534113	7.5905934	1.582731823	1.90E-20	8.49E-20
WAKMAR2	0.132084	0.7041631	2.414452114	1.07E-39	6.46E-38
AC023794.4	0.049257	0.1137966	1.20805622	0.000106138	0.000128
AC016586.1	0.119979	0.3172535	1.402859743	2.41E-08	3.70E-08
DIP2A-IT1	0.033077	0.122331	1.886872765	7.41E-06	9.65E-06
AC112491.1	0.3593	1.3739302	1.935046842	3.83E-21	1.81E-20
AC003681.1	0.042604	0.1119572	1.393874725	1.66E-12	3.58E-12
RAP2C-AS1	1.250434	0.5543201	-1.17363746	1.23E-33	2.12E-32
AC018638.7	0.248297	0.7305138	1.556844319	1.12E-12	2.45E-12
ARHGEF2-AS2	0.092308	0.3155439	1.77331026	8.94E-14	2.15E-13
IDH2-DT	1.404986	0.2017966	-2.79958156	1.61E-38	6.99E-37
AC004923.4	0.157851	0.649031	2.039723581	3.03E-22	1.62E-21
AC078802.1	0.369984	0.098802	-1.90484943	6.43E-26	4.85E-25
AP001781.1	0.05991	0.1765718	1.559374836	2.79E-10	5.00E-10
AC005840.2	0.190085	0.7931563	2.060961767	1.82E-22	9.88E-22
AL591721.1	0.078147	0.173579	1.151328904	0.004363049	0.004728

AC138956.2	0.503211	1.6506222	1.713775709	7.23E-17	2.31E-16
AP001020.3	0.033136	0.1270832	1.939312732	3.97E-14	9.88E-14
SSTR5-AS1	0.928594	0.0605901	-3.9378953	1.50E-36	4.42E-35
AL133553.1	0.016984	0.1141528	2.748757404	2.15E-12	4.58E-12
AL357078.2	0.043064	0.2643837	2.618063575	2.08E-17	7.05E-17
AL512506.1	0.057904	0.1922546	1.73128593	2.45E-10	4.43E-10
AP003555.2	0.088634	0.2329567	1.394125804	3.49E-08	5.33E-08
AC069281.2	0.371931	0.7652294	1.040857033	6.78E-09	1.09E-08
AC129510.1	0.363975	1.0390353	1.513332836	1.12E-11	2.26E-11
AC023983.1	0.05103	0.1234189	1.274137693	0.013449887	0.013983
AC011825.2	0.128157	0.4122887	1.685748105	5.16E-11	9.80E-11
AC006064.1	0.039086	0.2232744	2.514091756	3.13E-19	1.26E-18
PCCA-AS1	0.046708	0.1942352	2.056073083	0.002897183	0.003173
AC244093.3	0.013341	0.1321888	3.308705813	4.47E-09	7.25E-09
AC023421.1	10.33512	0.109044	-6.56650061	2.25E-34	4.35E-33
ST3GAL6-AS1	2.028862	0.5560749	-1.86731925	1.75E-31	2.28E-30
AC012213.3	0.060652	0.2132014	1.813594105	1.99E-17	6.76E-17
AL135999.3	1.545612	0.458942	-1.75179438	9.49E-39	4.37E-37
AL512363.1	0.58254	0.0807829	-2.85023511	2.21E-55	1.08E-52
AC037487.2	0.013522	0.2125474	3.974399533	4.72E-07	6.63E-07

FAM242C	0.707949	0.0735671	-3.26651238	3.00E-37	1.06E-35
AC025917.1	0.206883	0.6155895	1.573153707	6.52E-14	1.59E-13
AC127024.2	0.027137	0.1143178	2.074736343	2.76E-10	4.95E-10
AC008115.3	0.325154	1.1593943	1.83417502	5.75E-24	3.61E-23
AC106782.5	0.595329	1.199866	1.011114461	3.18E-17	1.06E-16
AC244197.2	0.120919	0.6641607	2.457493079	1.09E-12	2.40E-12
AC233728.1	0.234389	0.5055387	1.108915099	1.53E-06	2.08E-06
LINC01357	0.030277	0.3181871	3.393601371	3.25E-31	4.12E-30
AC024267.5	0.027299	0.1150492	2.075357684	1.30E-08	2.04E-08
DHDDS-AS1	0.137908	0.4048232	1.553586168	1.08E-14	2.82E-14
CDKN2B-AS1	0.008285	0.1723205	4.378497468	2.34E-40	1.54E-38
AP001107.1	0.113968	0.2867884	1.331363097	1.38E-16	4.28E-16
AC015914.1	0.027078	0.1281485	2.242618682	1.22E-05	1.56E-05
AC116667.1	0.225577	0.5940429	1.396949427	1.63E-13	3.86E-13
RHOQ-AS1	0.076464	0.2951662	1.948666822	1.41E-16	4.38E-16
AC010761.3	0.182289	0.6530502	1.840966868	7.63E-14	1.86E-13
ZKSCAN2-DT	0.240281	0.8576706	1.835700866	1.06E-16	3.32E-16
AL008729.2	0.05902	0.1198511	1.02197652	0.000585415	0.000676
APP-DT	0.783941	0.3735932	-1.0692778	6.12E-23	3.44E-22
AL135999.1	0.211246	0.8930687	2.07984678	7.64E-17	2.43E-16

AC092718.5	0.028691	0.1802731	2.651537601	2.61E-12	5.53E-12
SRD5A3-AS1	0.097739	0.246246	1.333101145	7.86E-30	8.91E-29
AC010201.1	0.285731	0.6857273	1.262976276	4.87E-07	6.83E-07
CATIP-AS2	1.148342	0.37883	-1.59992939	4.61E-30	5.32E-29
AC007406.1	0.39816	1.270502	1.673978076	2.41E-05	3.03E-05
MALAT1	15.83501	71.623804	2.177321674	3.77E-10	6.67E-10
LINC02145	0.05309	0.180561	1.765976398	1.36E-11	2.70E-11
LINC01772	0.180998	0.4818122	1.412495657	1.42E-19	5.92E-19
AC090515.5	0.041296	0.149194	1.853121175	0.002592524	0.002857
FP325332.1	0.047655	0.1127604	1.242553067	4.69E-05	5.78E-05
AF117829.1	0.30926	0.7210535	1.221288031	1.23E-18	4.75E-18
RASGRP3-AS1	0.211983	0.6138098	1.533844276	2.93E-12	6.20E-12
AC067817.2	0.250253	0.9930109	1.988419434	3.68E-11	7.05E-11
AC005391.1	0.057954	0.1764679	1.60643381	0.011954517	0.012487
AP000355.1	0.43931	1.5907842	1.856427571	1.11E-12	2.43E-12
AC034102.6	0.05554	0.1527824	1.459885685	8.86E-14	2.14E-13
AC012640.1	0.019688	0.1261565	2.67984809	2.23E-07	3.20E-07
AC005884.2	0.048838	0.1413894	1.533608174	1.41E-05	1.79E-05
AC002066.1	0.043866	0.1362647	1.635237039	1.22E-13	2.93E-13
AC079075.1	0.07484	0.1743581	1.220172961	9.44E-05	0.000115

AC011377.1	0.162167	0.3565179	1.136498208	0.000299069	0.000352
AP006623.1	0.196933	0.9383628	2.252443251	3.04E-20	1.32E-19
AL139041.1	0.042387	0.2075145	2.29150884	1.05E-16	3.32E-16
AC114730.3	0.105112	0.5002546	2.250729087	1.02E-14	2.67E-14
AC138207.7	0.052719	0.1840391	1.803618545	6.60E-08	9.83E-08
DIAPH1-AS1	0.004286	0.1137636	4.730188607	2.41E-12	5.13E-12
AC005082.1	6.499593	0.7917328	-3.03726384	1.00E-34	2.06E-33
LBX2-AS1	3.087291	7.8776563	1.351425176	3.70E-28	3.52E-27
AC005264.1	0.014622	0.2371624	4.019617418	1.96E-35	4.46E-34
AP000941.1	0.154208	0.3968613	1.363758858	2.63E-09	4.38E-09
AL391261.2	0.32087	0.126621	-1.34147355	5.17E-13	1.17E-12
HLA-F-AS1	0.333382	1.1396087	1.773292269	2.12E-39	1.18E-37
AL161668.3	0.037297	0.2160622	2.534319858	1.47E-19	6.09E-19
AL121974.1	0.50943	0.1091753	-2.22223747	1.18E-31	1.55E-30
AC032044.1	0.070081	0.2293699	1.710580412	1.13E-12	2.46E-12
AC112484.1	0.149921	0.3300244	1.138371061	4.56E-12	9.48E-12
AL136221.1	0.077339	0.1597135	1.046219933	0.035301696	0.035667
AL356124.1	0.068565	0.2053168	1.582309619	6.82E-06	8.90E-06
N4BP2L2-IT2	0.146034	0.7729454	2.404065212	1.96E-27	1.74E-26
AC008870.4	0.038804	0.1663081	2.099574725	1.16E-15	3.30E-15

AC010761.6	0.529955	0.2311806	-1.19685101	3.16E-18	1.18E-17
LINC02345	0.03134	0.1135534	1.857284381	8.19E-18	2.88E-17
PVT1	0.124391	3.1310986	4.653718144	3.04E-42	2.74E-40
AC105118.1	0.02291	0.2828461	3.625970834	4.69E-07	6.59E-07
IGBP1-AS1	0.096277	0.3778404	1.972520455	7.08E-17	2.27E-16
AC005332.5	0.789173	1.8978625	1.265961511	5.79E-27	4.89E-26
AL096701.3	0.087899	0.3051768	1.795731479	2.57E-16	7.74E-16
MIR4435-2HG	1.056914	4.0434246	1.935720061	5.52E-37	1.90E-35
XPC-AS1	0.209245	0.4505307	1.106434754	1.27E-12	2.76E-12
AC016949.1	0.090029	0.3433061	1.93103273	3.61E-17	1.19E-16
AC008750.4	0.081667	0.2775603	1.764974087	1.53E-13	3.62E-13
LINC01801	0.279713	0.5771739	1.045059762	0.000624417	0.00072
AC006435.2	0.201304	0.9377882	2.219883789	2.41E-15	6.74E-15
AC079313.1	0.050809	0.4032343	2.988472793	3.32E-23	1.93E-22
AC009996.1	0.121246	0.3695115	1.607683527	2.82E-10	5.06E-10
LINC02340	0.080075	0.1927393	1.267218657	5.71E-05	7.02E-05
DHRS4-AS1	14.17352	6.9185014	-1.03466683	4.02E-29	4.31E-28
AC127502.2	0.607824	2.1793951	1.842202374	6.78E-26	5.07E-25
AC008991.1	0.021533	0.4094077	4.248911549	3.46E-13	7.96E-13
AL162724.2	0.207477	1.8920046	3.188893103	9.77E-34	1.71E-32

AP003352.1	0.458921	1.3351527	1.540685777	2.28E-24	1.47E-23
LINC02642	0.021493	0.1506248	2.808993533	1.92E-23	1.15E-22
ATP2B1-AS1	1.03156	0.475863	-1.11620982	8.78E-27	7.28E-26
LINC00315	0.057702	0.1355281	1.23190947	4.71E-17	1.54E-16
AC124017.1	4.182311	0.042685	-6.61442719	1.43E-41	1.16E-39
LINC01402	0.190616	0.5144136	1.432257445	1.06E-08	1.66E-08
AC087045.2	0.076892	0.1573467	1.033035413	0.002261971	0.002506
AC011481.2	0.173587	0.4976536	1.519483694	5.88E-23	3.32E-22
LINC01320	9.481668	24.130597	1.347650804	1.16E-18	4.47E-18
AC068792.1	0.22652	0.576237	1.347022838	5.95E-21	2.76E-20
SCAT1	0.011205	0.1956313	4.125983303	2.03E-26	1.61E-25
AC011511.5	0.043544	0.1090858	1.324920989	2.12E-14	5.42E-14
AL512274.1	0.598685	0.2882589	-1.05443272	2.33E-11	4.55E-11
AC009237.15	1.808554	0.679508	-1.41227457	2.71E-17	9.08E-17
KIF1C-AS1	0.128573	0.5341268	2.054598368	1.76E-23	1.06E-22
AC106028.2	0.066488	0.1729944	1.379567483	8.04E-06	1.04E-05
AC005387.2	0.076183	0.4880785	2.679565987	2.08E-23	1.23E-22
NALT1	0.153687	0.6417205	2.061954168	2.63E-12	5.57E-12
AL451085.1	0.208405	0.0934744	-1.15674892	1.92E-21	9.38E-21
TCEAL3-AS1	0.050803	0.1526396	1.587137947	0.004360952	0.004727

FSIP2-AS1	0.074799	0.2827154	1.918266582	1.93E-14	4.94E-14
AL031705.1	0.052757	0.2219742	2.072944324	3.04E-13	7.02E-13
AL035448.1	0.085688	0.191144	1.157491617	0.000187733	0.000224
FARP1-AS1	0.064995	0.1367334	1.072968414	0.001648414	0.00185
AC008875.1	0.045316	0.3073095	2.761587171	5.88E-25	4.04E-24
MAPT-AS1	0.118243	0.2661383	1.170426203	0.003325584	0.003629
AC002550.2	0.41261	0.9884433	1.260379313	1.49E-18	5.71E-18
LINC02343	1.984955	0.0847745	-4.549332	2.74E-54	1.11E-51
AC016405.3	0.288724	1.6550081	2.519076294	2.74E-23	1.61E-22
AL513477.2	0.355741	0.8391567	1.238113241	3.71E-11	7.11E-11
AC109460.2	0.216085	0.7905386	1.871240094	7.14E-16	2.05E-15
TH2LCRR	0.059107	0.2507007	2.084572469	9.11E-17	2.88E-16
AL049780.1	0.141856	0.5707986	2.008557181	4.22E-14	1.05E-13
AC026904.2	0.269604	0.128471	-1.0694004	5.44E-23	3.10E-22
AL157392.4	0.134044	0.5661377	2.078442644	1.63E-15	4.58E-15
AC127024.4	0.250425	1.055793	2.075876334	3.21E-17	1.07E-16
CRYZL2P-SEC16B	0.100537	0.3370873	1.745390507	9.08E-20	3.80E-19
AL450998.2	0.095521	0.3329246	1.80130452	8.38E-17	2.66E-16
AL390208.1	0.176573	0.4730335	1.421680546	2.12E-13	4.98E-13
AC091180.5	0.044587	0.1951527	2.129899886	2.67E-11	5.19E-11

AC007098.1	0.106151	0.5725074	2.431174731	2.41E-28	2.32E-27
AL162727.2	0.121848	0.3357024	1.462102484	4.40E-17	1.44E-16
ALG13-AS1	0.229273	1.0596642	2.208467224	3.30E-21	1.57E-20
AC116351.1	0.526054	1.512762	1.523901555	0.000264044	0.000312
ASMTL-AS1	0.500564	3.2758993	2.710265897	2.21E-15	6.19E-15
LINC02332	0.66096	0.0822169	-3.00705647	7.70E-37	2.54E-35
AL109761.1	0.044748	0.1112412	1.313789919	0.002582087	0.002847
AC008543.3	0.060493	0.2214769	1.872304729	2.84E-05	3.55E-05
AC129492.1	0.27562	0.6983061	1.341179894	1.81E-09	3.04E-09
CACNA1C-AS1	0.032255	0.1133715	1.813483018	6.17E-18	2.22E-17
AC097382.1	0.015315	0.1734493	3.501533307	1.37E-13	3.26E-13
AL591178.1	0.064919	0.1646439	1.342639246	0.00946513	0.009981
AC005332.7	0.130387	0.3944775	1.597144562	6.28E-17	2.02E-16
AC091965.1	0.680509	0.1258974	-2.43436678	1.44E-37	5.33E-36
AL109659.2	0.084746	0.3250274	1.939340254	1.99E-19	8.11E-19
RHOA-IT1	0.121514	0.4256076	1.808396865	2.02E-12	4.32E-12
AC020978.1	0.048963	0.1597632	1.706168565	5.47E-08	8.22E-08
LINC01606	2.060511	0.214273	-3.26548042	7.24E-42	6.31E-40
AC010531.5	0.043424	0.2447244	2.494582931	4.56E-18	1.67E-17
AC010168.2	0.390522	0.9006339	1.205538809	9.29E-12	1.89E-11

LINC01285	0.075287	0.1980515	1.395397307	3.28E-16	9.76E-16
AC025171.3	0.089515	0.2955422	1.723165528	1.28E-18	4.91E-18
Z97200.1	0.014978	0.1736135	3.534917783	1.22E-17	4.24E-17
XIST	0.997847	3.0594206	1.616367425	4.48E-08	6.77E-08
AC002128.1	0.204417	0.6661215	1.704269735	6.57E-12	1.35E-11
PTPRN2-AS1	0.091457	0.624373	2.771240842	2.25E-34	4.35E-33
AC126118.1	0.105057	0.3414381	1.700446903	2.42E-11	4.71E-11
TPM1-AS	0.14679	0.4720974	1.68532978	1.26E-16	3.90E-16
NADK2-AS1	0.21976	0.4723405	1.103896559	3.58E-08	5.46E-08
PRKAR1B-AS2	5.260377	11.133919	1.081723242	3.19E-06	4.24E-06
AC008759.2	0.111313	0.2527536	1.183108556	0.00020717	0.000246
AC103706.1	0.269836	0.7556605	1.485656467	7.65E-18	2.71E-17
PKD1P6-NPIPP1	0.151197	0.4625608	1.613217427	2.94E-25	2.07E-24
AC093206.1	0.015785	0.1319579	3.063406673	6.33E-08	9.45E-08
LINC01146	0.037904	0.209535	2.466779243	4.67E-27	4.01E-26
AL118522.1	0.404389	0.1138432	-1.8286946	1.87E-20	8.39E-20
AC110995.1	0.19448	0.6507647	1.742511823	3.35E-21	1.59E-20
AC015660.1	0.568763	0.2152636	-1.40172353	6.30E-20	2.67E-19
AP000786.1	0.150444	0.3789292	1.332704116	2.95E-07	4.19E-07
SCGB1B2P	0.423333	1.0358653	1.290971734	4.10E-16	1.21E-15

AL731569.1	0.145928	0.3714743	1.348001154	3.27E-10	5.83E-10
COL4A2-AS1	0.140709	0.5531671	1.974997548	1.78E-16	5.45E-16
AC105020.5	0.289828	1.0554078	1.864532643	2.15E-21	1.04E-20
AC003682.1	0.027536	0.1098347	1.995928054	5.04E-09	8.16E-09
AC090181.2	0.200373	0.5030103	1.327902058	3.91E-09	6.37E-09
AC243829.4	0.009532	0.255827	4.746209504	1.09E-33	1.91E-32
AC005730.3	0.060561	0.2482962	2.035596	7.69E-10	1.32E-09
RTCA-AS1	5.171182	1.6997108	-1.60520471	4.96E-35	1.07E-33
AC004951.4	0.067101	0.1551524	1.209272142	2.61E-13	6.06E-13
AL158163.2	0.135785	0.3884662	1.516468016	8.84E-20	3.70E-19
AL162430.2	0.068273	0.3076356	2.171826369	9.24E-18	3.23E-17
AC110015.1	0.044358	0.276912	2.642170055	5.82E-23	3.30E-22
VPS9D1-AS1	0.229626	0.755629	1.718389148	5.67E-11	1.07E-10
AC060766.5	0.235001	0.5308464	1.175627853	2.54E-12	5.39E-12
MIR99AHG	1.903442	0.763132	-1.3186063	1.95E-31	2.48E-30
AL513190.1	0.110531	0.3315729	1.584880954	5.47E-14	1.35E-13
AC024267.3	0.145109	0.3909014	1.429664407	1.08E-10	2.01E-10
LINC00271	0.905164	0.2800971	-1.69225301	1.89E-36	5.12E-35
AC005034.2	0.100467	0.2361289	1.232858839	7.18E-07	9.97E-07
AC020558.2	0.191131	0.5663177	1.56705033	9.48E-12	1.92E-11

RUFY1-AS1	0.075976	0.3646309	2.262818606	1.94E-22	1.05E-21
AC135178.6	0.300389	0.7127197	1.246504364	5.99E-22	3.11E-21
AL132780.1	1.3424	0.4065074	-1.72346114	3.87E-25	2.70E-24
AP001793.1	0.144976	0.3441543	1.247239316	8.56E-11	1.60E-10
BDNF-AS	1.609536	0.7946724	-1.01821254	1.92E-32	2.84E-31
LINC02285	0.100156	0.3657214	1.868502037	1.37E-27	1.24E-26
AC116552.1	0.065841	0.3241342	2.29952536	3.53E-14	8.86E-14
TMEM246-AS1	3.836072	1.0680662	-1.84462874	6.19E-12	1.28E-11
AC015961.2	0.192925	0.4976271	1.367024334	1.05E-09	1.79E-09
AC023669.2	0.277618	1.9037758	2.777691057	0.000737398	0.000846
AC084824.3	0.189595	0.5323445	1.489440289	3.43E-18	1.27E-17
AC136424.2	0.007058	0.1902367	4.75240563	6.97E-09	1.11E-08
AC005899.1	0.026454	0.1973724	2.899351733	1.27E-19	5.29E-19
AL391095.3	0.04357	0.1626693	1.90053265	3.60E-08	5.49E-08
AC007292.2	0.124418	0.317285	1.350578179	3.92E-24	2.49E-23
IQCA1-AS1	0.151347	0.339075	1.1637415	8.94E-07	1.23E-06
PARD3-AS1	0.513022	1.1480932	1.162146654	5.76E-09	9.25E-09
AC096992.2	0.557621	1.1493122	1.043413668	6.90E-24	4.27E-23
AC135050.4	0.036195	0.1435884	1.988082473	1.51E-11	3.00E-11
AC011472.2	0.118749	0.4016846	1.758150205	6.77E-24	4.20E-23

AC067930.2	0.083263	0.1775795	1.092717506	0.000355528	0.000417
AC109460.3	0.038774	0.1975304	2.348909111	6.75E-23	3.79E-22
AC106037.3	0.045408	0.1468961	1.693774728	0.020645432	0.021184
AC093915.1	0.040442	0.5125292	3.663700167	3.90E-20	1.68E-19
AC135050.1	0.055687	0.1208223	1.117464994	1.56E-15	4.41E-15
AC092301.1	0.067194	0.2419838	1.84850221	3.75E-15	1.03E-14
ACAP2-IT1	0.180357	0.4439009	1.299385849	6.70E-08	9.97E-08
AC079684.1	0.17104	0.5814924	1.765424875	6.02E-17	1.94E-16
AC105105.3	0.027003	0.3181649	3.558592714	1.40E-24	9.26E-24
UCKL1-AS1	0.062514	0.2057938	1.718952939	5.51E-12	1.14E-11
AL033523.1	0.348488	0.1087195	-1.6804987	0.005617805	0.006039
AC011461.1	0.062127	0.2889015	2.217279957	4.07E-13	9.28E-13
REV3L-IT1	0.07923	0.2290123	1.531298318	0.024309171	0.024797
AL139099.2	0.086351	0.199143	1.205516838	1.53E-06	2.09E-06
AC105339.3	0.067399	0.2455511	1.865222008	9.69E-10	1.66E-09
AP007216.2	0.039121	0.4250486	3.441605314	1.23E-27	1.12E-26
AP000894.4	3.705652	1.1440809	-1.69553835	4.01E-32	5.53E-31
AC009336.1	0.459099	0.2119756	-1.11490816	1.86E-17	6.32E-17
AL353801.2	0.327163	0.103046	-1.6667206	1.77E-21	8.71E-21
AC007619.1	0.200898	0.447549	1.155579738	3.62E-07	5.12E-07

LINC01213	0.634512	0.2106998	-1.59045875	1.28E-36	3.85E-35
AC010501.2	1.006588	0.185455	-2.44033245	6.04E-39	2.89E-37
AC106028.3	0.127531	0.2692991	1.078358976	5.42E-10	9.47E-10
AC092828.1	0.045911	0.1653619	1.848709189	0.00018881	0.000226
AL136115.2	0.075702	0.2712644	1.841303926	3.51E-06	4.66E-06
AL512656.1	0.088754	0.2177583	1.294837801	1.20E-05	1.53E-05
AC011472.1	0.705543	2.1372534	1.598951349	2.40E-13	5.59E-13
AC012645.4	0.05853	0.3387184	2.532829417	1.68E-17	5.76E-17
AL354836.1	1.377484	4.8285571	1.809556314	5.64E-18	2.04E-17
AP002340.1	0.120918	0.3104515	1.360344862	2.27E-08	3.50E-08
LINC00862	0.027372	0.1598312	2.545780887	9.74E-07	1.34E-06
FAM215A	0.54183	0.0527688	-3.36008417	1.44E-34	2.94E-33
AP001029.1	0.054675	0.5442145	3.315235152	3.22E-17	1.07E-16
AC090970.1	0.044209	0.2858828	2.693018233	0.011194352	0.011733
H1-10-AS1	0.215542	0.4680428	1.118671145	3.54E-09	5.80E-09
CDC37L1-DT	1.998021	0.9579126	-1.06060552	6.12E-25	4.19E-24
LAMC1-AS1	0.227818	0.6013051	1.400214085	5.07E-14	1.25E-13
AC004918.3	1.140033	2.3043055	1.015256246	8.81E-15	2.32E-14
FGF14-IT1	0.025693	0.1164321	2.180033528	0.008316348	0.0088
AP000944.1	0.055861	0.1651477	1.563848002	0.00019504	0.000232

AP001458.1	0.077069	0.5226792	2.761697772	8.78E-14	2.12E-13
AC055811.3	0.019071	0.1122157	2.556799222	4.94E-10	8.65E-10
AC006160.1	0.112002	0.3930502	1.8111937	1.99E-17	6.76E-17
AC105094.2	0.450829	0.0586034	-2.94352474	1.26E-25	9.19E-25
AC079322.1	0.100086	0.4294852	2.101366119	2.01E-21	9.77E-21
ABCC5-AS1	0.015874	0.1575017	3.310649543	6.46E-06	8.45E-06
AL109976.1	0.754935	0.1842133	-2.03497401	1.31E-13	3.13E-13
AC129507.2	3.994117	1.9174237	-1.0587076	3.00E-21	1.44E-20
LINC02613	0.291461	0.0994771	-1.55086703	2.87E-26	2.24E-25
SLFNL1-AS1	0.079034	0.3619547	2.19527098	1.53E-22	8.38E-22
AL023881.1	0.177254	0.5362157	1.596997937	1.68E-18	6.40E-18
AC005532.1	0.084927	0.173071	1.027077323	0.000238228	0.000283
AC079336.5	0.031069	0.1350432	2.119894218	8.39E-18	2.95E-17
AC008555.1	0.381395	0.9449552	1.308960112	3.70E-10	6.56E-10
AC005363.2	0.06288	0.1455206	1.21055027	0.002507569	0.002768
SIRLNT	1.948539	0.0464893	-5.38934974	7.57E-49	1.15E-46
CACTIN-AS1	0.082132	0.1911531	1.218710314	1.46E-14	3.77E-14
AC097641.2	0.191838	0.4485731	1.225454418	3.47E-11	6.66E-11
AL731567.1	0.103841	0.8318235	3.001897284	1.53E-25	1.11E-24
ARAP1-AS2	0.034522	0.2599715	2.91274613	2.66E-23	1.56E-22

ITPR1-DT	1.062181	0.4628427	-1.1984356	2.39E-19	9.70E-19
AC012435.3	0.125945	0.2727253	1.114655401	1.14E-08	1.79E-08
AL135960.1	0.063128	0.2441645	1.951505561	1.49E-10	2.72E-10
AC091563.1	12.35485	5.5905881	-1.14400565	1.69E-32	2.53E-31
AP001636.3	0.054285	0.2368974	2.125642589	3.60E-30	4.21E-29
AC092919.1	0.069027	0.1616556	1.227698347	0.02327634	0.023764
AP002812.5	0.068617	0.360179	2.392082509	1.68E-22	9.21E-22
AC084026.2	0.029254	0.1807514	2.627293183	1.76E-17	6.01E-17
AC107081.1	0.067362	0.3096229	2.200498363	1.85E-19	7.55E-19
FGF12-AS2	0.024005	0.1381503	2.524811142	8.70E-18	3.06E-17
AC007993.2	2.418381	0.0439577	-5.78178114	1.79E-48	2.43E-46
AC008667.1	0.047834	0.2616894	2.451739154	4.44E-17	1.45E-16
AL022316.1	0.202244	0.5850425	1.53244659	7.35E-16	2.11E-15
AC068620.2	0.194321	0.4840511	1.316716337	4.62E-09	7.49E-09
AL031726.1	2.981812	0.0691651	-5.43000182	4.13E-39	2.08E-37
AC015849.4	0.035562	0.1679831	2.239895792	8.97E-12	1.82E-11
AC098869.2	0.186935	0.4548152	1.282746741	2.37E-07	3.40E-07
AP003555.1	0.027965	0.4375928	3.967890202	8.79E-35	1.83E-33
AC016027.2	0.008835	0.1157364	3.711474436	3.43E-21	1.63E-20
LINC00893	0.168803	0.8009611	2.246392173	9.65E-17	3.04E-16

AC010463.3	0.118364	0.3148205	1.411298076	7.37E-11	1.39E-10
AL158196.1	0.078931	0.2361042	1.580764241	7.08E-11	1.33E-10
AC022960.1	0.056337	0.312443	2.471442185	4.97E-09	8.04E-09
AL021707.8	0.398046	1.5027434	1.916593327	1.98E-16	6.03E-16
TEX41	0.328871	0.8575109	1.382631174	0.000921537	0.001052
AC004466.3	0.166599	0.4826354	1.534555804	7.54E-07	1.04E-06
AL121890.2	0.023261	0.1222294	2.393613251	1.25E-09	2.13E-09
TET2-AS1	0.034576	0.1494921	2.112212953	2.89E-06	3.86E-06
AL139280.1	9.836986	0.0894417	-6.78112552	1.18E-42	1.10E-40
SPINT1-AS1	9.524573	3.9585282	-1.26669026	1.02E-30	1.25E-29
AP001381.1	0.026086	0.2291534	3.134985871	8.46E-13	1.88E-12
AC011468.1	0.622301	1.6587847	1.414442651	3.06E-24	1.96E-23
ZNF436-AS1	0.579545	1.2940336	1.158882753	4.77E-18	1.74E-17
AC048382.2	0.179139	0.644482	1.847061011	2.58E-15	7.20E-15
AC004148.1	0.510153	1.863831	1.869270302	1.47E-17	5.07E-17
AC253576.2	0.224225	0.5107993	1.187807136	3.60E-05	4.47E-05
AL360181.2	0.624783	2.1603908	1.789864886	2.09E-18	7.89E-18
AC090527.3	0.098437	0.2211899	1.168007489	4.18E-05	5.16E-05
AC004461.2	0.0573	0.2029839	1.824747914	4.14E-15	1.13E-14
LINC01004	0.64816	1.7181849	1.406462868	7.52E-11	1.41E-10

AL592071.1	0.047303	0.1750512	1.88775899	6.65E-08	9.89E-08
AC025176.1	0.039396	0.2134501	2.43776066	4.17E-13	9.48E-13
LENG8-AS1	1.052021	3.3027486	1.650503708	3.37E-22	1.78E-21
BMPR1B-DT	1.455254	0.0541696	-4.7476438	4.55E-52	1.11E-49
AC005332.1	0.054997	0.3069935	2.480781643	6.54E-21	3.01E-20
LINC02614	0.149907	0.378057	1.334536067	8.01E-15	2.12E-14
AC010336.2	0.754045	0.2492611	-1.59699254	1.16E-10	2.16E-10
AC024270.1	0.033704	0.1358367	2.010895008	5.74E-08	8.60E-08
AC006272.1	0.036271	0.2514555	2.793423651	1.37E-24	9.07E-24
HOXB-AS1	0.841339	2.1179722	1.331925075	4.21E-18	1.55E-17
AC131971.1	0.038572	0.2413086	2.645260984	1.26E-09	2.15E-09
LINC00174	0.635957	1.8560507	1.545235793	1.25E-17	4.31E-17
LINC01176	0.548287	2.119977	1.951044831	2.84E-21	1.37E-20
RBM38-AS1	0.036888	0.1151248	1.641967328	1.17E-16	3.64E-16
AC084048.1	0.016599	0.2546492	3.939371846	0.031486334	0.031905
AC108673.3	1.135738	2.3381435	1.041733665	2.45E-10	4.43E-10
SKAP1-AS1	0.044616	0.1747538	1.969685303	3.54E-05	4.40E-05
AC010300.1	0.08118	0.2171806	1.419704714	1.89E-07	2.73E-07
AC010883.1	0.578507	1.5098988	1.384045382	1.65E-12	3.56E-12
AC017006.2	0.01799	0.1878668	3.384450576	1.06E-05	1.37E-05

GNA14-AS1	0.03679	0.1532323	2.058324097	0.004936546	0.005323
AC027271.1	0.160882	0.4021009	1.321550234	1.41E-14	3.66E-14
AC106037.2	0.084257	0.2081786	1.304950688	2.15E-05	2.70E-05
AC109460.1	0.127675	0.3390552	1.409040657	1.39E-11	2.76E-11
AC093752.1	0.034979	0.137897	1.979032454	5.64E-10	9.84E-10
AL442128.2	0.096116	0.22603	1.233660113	3.08E-08	4.71E-08
AC008610.1	0.648958	1.7859403	1.460486715	1.03E-11	2.07E-11
AL158151.4	0.03579	0.3077053	3.103924958	1.48E-20	6.66E-20
EP300-AS1	0.400995	0.8602054	1.101096414	2.14E-13	5.01E-13
AC087500.2	0.323474	0.6818753	1.075858281	1.74E-09	2.93E-09
DAPK1-IT1	0.008797	0.174362	4.308910475	5.81E-08	8.70E-08
SCAT2	0.178514	0.6079674	1.767957062	4.71E-08	7.11E-08
AC073569.1	0.038545	0.1468305	1.929543877	0.027008585	0.027425
AC007216.2	0.066103	0.2314647	1.807998404	4.26E-09	6.93E-09
AL022328.2	0.6408	2.0636389	1.687243669	2.97E-17	9.90E-17
