# Supplementary material for: Fatty acid metabolism-related lncRNAs are potential biomarkers for survival prediction in clear cell renal cell carcinoma
Source: Medicine (Baltimore). 2024 Feb 23;103(8):e37207. doi: 10.1097/MD.0000000000037207 (PMC11309608; doi:10.1097/MD.0000000000037207)
Supplement: Supplementary file 6 [file medi-103-e37207-s006.pdf]

TFs	lncRNA	cor	pvalue	Regulation
ZSCAN30	BMS1P4	0.678805	7.85E-73	postive
ZSCAN30	PDXDC2P-NPIPB14P	0.607169	1.06E-54	postive
ZSCAN30	LINC00894	0.611161	1.37E-55	postive
ZSCAN30	GABPB1-AS1	0.627584	2.19E-59	postive
ZSCAN30	SSBP3-AS1	0.620298	1.13E-57	postive
ZSCAN30	ARHGAP27P1-BPTFP1-KPNA2P3	0.673758	2.19E-71	postive
ZSCAN30	FAM13A-AS1	0.610653	1.78E-55	postive
ZSCAN30	SNHG20	0.612462	6.98E-56	postive
ZSCAN30	INE1	0.641438	9.05E-63	postive
ZSCAN30	N4BP2L2-IT2	0.626504	3.95E-59	postive
ZSCAN30	LINC00893	0.624658	1.08E-58	postive
ZSCAN30	LINC00174	0.623407	2.13E-58	postive
ZNF692	RFPL3S	0.755429	5.22E-99	postive
ZNF692	GUSBP11	0.745279	5.26E-95	postive
ZNF692	BMS1P4	0.693189	4.08E-77	postive
ZNF692	C9orf139	0.69952	4.41E-79	postive
ZNF692	PDXDC2P-NPIPB14P	0.781915	########	postive
ZNF692	RRN3P2	0.611943	9.14E-56	postive
ZNF692	LINC02656	0.647116	3.30E-64	postive
ZNF692	HCG27	0.738121	2.70E-92	postive
ZNF692	LINC00894	0.719903	8.81E-86	postive
ZNF692	DPP9-AS1	0.72334	5.70E-87	postive
ZNF692	IBA57-DT	0.679163	6.18E-73	postive
ZNF692	DTX2P1-UPK3BP1-PMS2P11	0.648241	1.70E-64	postive
ZNF692	LINC00173	0.699365	4.93E-79	postive
ZNF692	GABPB1-AS1	0.626676	3.60E-59	postive
ZNF692	SNHG3	0.734955	4.00E-91	postive
ZNF692	ARHGAP27P1-BPTFP1-KPNA2P3	0.821938	########	postive
ZNF692	LINC00115	0.788242	########	postive
ZNF692	FAM13A-AS1	0.808568	########	postive
ZNF692	SNHG20	0.738284	2.35E-92	postive
ZNF692	SNHG17	0.60586	2.07E-54	postive
ZNF692	INE1	0.658181	4.23E-67	postive
ZNF692	C3orf35	0.739804	6.34E-93	postive
ZNF692	MALAT1	0.643704	2.43E-63	postive
ZNF692	N4BP2L2-IT2	0.725028	1.46E-87	postive
ZNF692	PVT1	0.658564	3.34E-67	postive
ZNF692	ASMTL-AS1	0.807324	########	postive

ZNF692	SCGB1B2P	0.642686	4.39E-63	postive
ZNF692	H1-10-AS1	0.740552	3.32E-93	postive
ZNF692	LINC00893	0.78266	########	postive
ZNF692	LINC00174	0.841492	########	postive
ZNF682	RFPL3S	0.669088	4.49E-70	postive
ZNF682	GUSBP11	0.611118	1.40E-55	postive
ZNF682	BMS1P4	0.698406	9.86E-79	postive
ZNF682	C9orf139	0.669255	4.04E-70	postive
ZNF682	PDXDC2P-NPIPB14P	0.696844	3.03E-78	postive
ZNF682	LINC02656	0.650622	4.12E-65	postive
ZNF682	HCG27	0.64708	3.37E-64	postive
ZNF682	LINC00894	0.660831	8.22E-68	postive
ZNF682	DPP9-AS1	0.663752	1.33E-68	postive
ZNF682	IBA57-DT	0.656891	9.32E-67	postive
ZNF682	GABPB1-AS1	0.672196	6.05E-71	postive
ZNF682	SNHG3	0.612735	6.06E-56	postive
ZNF682	ARHGAP27P1-BPTFP1-KPNA2P3	0.765889	########	postive
ZNF682	LINC00115	0.643831	2.26E-63	postive
ZNF682	FAM13A-AS1	0.70172	8.89E-80	postive
ZNF682	SNHG20	0.631518	2.49E-60	postive
ZNF682	INE1	0.618375	3.14E-57	postive
ZNF682	C3orf35	0.696483	3.93E-78	postive
ZNF682	N4BP2L2-IT2	0.692195	8.22E-77	postive
ZNF682	ASMTL-AS1	0.652936	1.03E-65	postive
ZNF682	H1-10-AS1	0.611884	9.43E-56	postive
ZNF682	LINC00893	0.680094	3.32E-73	postive
ZNF682	LINC00174	0.737206	5.91E-92	postive
ZNF326	BMS1P4	0.63239	1.53E-60	postive
ZNF326	ARHGAP27P1-BPTFP1-KPNA2P3	0.641908	6.89E-63	postive
ZNF326	INE1	0.617789	4.28E-57	postive
ZNF326	N4BP2L2-IT2	0.61978	1.49E-57	postive
AKAP8L	RFPL3S	0.708095	7.88E-82	postive
AKAP8L	GUSBP11	0.697304	2.18E-78	postive
AKAP8L	BMS1P4	0.652617	1.25E-65	postive
AKAP8L	C9orf139	0.629644	7.05E-60	postive
AKAP8L	PDXDC2P-NPIPB14P	0.772593	########	postive
AKAP8L	HCG27	0.665334	4.90E-69	postive
AKAP8L	LINC00894	0.736188	1.41E-91	postive
AKAP8L	DPP9-AS1	0.663102	2.00E-68	postive

AKAP8L	IBA57-DT	0.6309	3.52E-60	postive
AKAP8L	DTX2P1-UPK3BP1-PMS2P11	0.611524	1.14E-55	postive
AKAP8L	GABPB1-AS1	0.604815	3.51E-54	postive
AKAP8L	SNHG3	0.638171	5.89E-62	postive
AKAP8L	ARHGAP27P1-BPTFP1-KPNA2P3	0.761296	########	postive
AKAP8L	LINC00115	0.77903	########	postive
AKAP8L	FAM13A-AS1	0.693381	3.56E-77	postive
AKAP8L	SNHG20	0.699088	6.03E-79	postive
AKAP8L	INE1	0.701646	9.38E-80	postive
AKAP8L	C3orf35	0.678403	1.03E-72	postive
AKAP8L	LINC00265	0.611753	1.01E-55	postive
AKAP8L	MALAT1	0.63536	2.90E-61	postive
AKAP8L	N4BP2L2-IT2	0.690908	2.03E-76	postive
AKAP8L	ASMTL-AS1	0.796887	########	postive
AKAP8L	SCGB1B2P	0.609117	3.92E-55	postive
AKAP8L	H1-10-AS1	0.744862	7.61E-95	postive
AKAP8L	LINC00893	0.779577	########	postive
AKAP8L	LINC00174	0.779948	########	postive
ZNF580	SCGB1B2P	0.638382	5.22E-62	postive
ZNF580	H1-10-AS1	0.665458	4.53E-69	postive
FOXP3	PCED1B-AS1	0.705727	4.63E-81	postive
ZNF8	INE1	0.634873	3.82E-61	postive
ZBTB48	GUSBP11	0.713141	1.71E-83	postive
ZBTB48	C9orf139	0.652008	1.80E-65	postive
ZBTB48	PDXDC2P-NPIPB14P	0.656148	1.47E-66	postive
ZBTB48	LINC02656	0.607779	7.79E-55	postive
ZBTB48	HCG27	0.73188	5.28E-90	postive
ZBTB48	DPP9-AS1	0.618718	2.62E-57	postive
ZBTB48	IBA57-DT	0.604483	4.15E-54	postive
ZBTB48	LINC00173	0.681587	1.22E-73	postive
ZBTB48	ARHGAP27P1-BPTFP1-KPNA2P3	0.722287	1.32E-86	postive
ZBTB48	LINC00115	0.717072	8.14E-85	postive
ZBTB48	FAM13A-AS1	0.713454	1.34E-83	postive
ZBTB48	SNHG20	0.707413	1.31E-81	postive
ZBTB48	N4BP2L2-IT2	0.6412	1.04E-62	postive
ZBTB48	ASMTL-AS1	0.671125	1.21E-70	postive
ZBTB48	H1-10-AS1	0.67192	7.24E-71	postive
ZBTB48	LINC00893	0.671584	9.00E-71	postive
ZBTB48	LINC00174	0.705241	6.64E-81	postive

TIGD1	RFPL3S	0.618564	2.84E-57	postive
TIGD1	GUSBP11	0.620451	1.04E-57	postive
TIGD1	HCG27	0.668042	8.78E-70	postive
TIGD1	LINC00894	0.655611	2.04E-66	postive
TIGD1	LINC00115	0.649613	7.52E-65	postive
TIGD1	LINC00893	0.636974	1.16E-61	postive
TIGD1	LINC00174	0.673106	3.35E-71	postive
ZNF337	RFPL3S	0.64804	1.91E-64	postive
ZNF337	GUSBP11	0.631216	2.95E-60	postive
ZNF337	BMS1P4	0.745589	3.99E-95	postive
ZNF337	C9orf139	0.654594	3.77E-66	postive
ZNF337	PDXDC2P-NPIPB14P	0.758223	########	postive
ZNF337	LINC02656	0.671182	1.17E-70	postive
ZNF337	HCG27	0.696932	2.85E-78	postive
ZNF337	LINC00894	0.730685	1.43E-89	postive
ZNF337	DPP9-AS1	0.687562	2.07E-75	postive
ZNF337	IBA57-DT	0.641083	1.11E-62	postive
ZNF337	DTX2P1-UPK3BP1-PMS2P11	0.633046	1.06E-60	postive
ZNF337	LINC00173	0.67611	4.68E-72	postive
ZNF337	GABPB1-AS1	0.734729	4.84E-91	postive
ZNF337	SNHG3	0.608492	5.41E-55	postive
ZNF337	SSBP3-AS1	0.677716	1.62E-72	postive
ZNF337	ARHGAP27P1-BPTFP1-KPNA2P3	0.785293	########	postive
ZNF337	LINC00115	0.699932	3.27E-79	postive
ZNF337	FAM13A-AS1	0.792368	########	postive
ZNF337	SNHG20	0.711394	6.49E-83	postive
ZNF337	INE1	0.750487	4.91E-97	postive
ZNF337	C3orf35	0.637765	7.42E-62	postive
ZNF337	MALAT1	0.686986	3.08E-75	postive
ZNF337	N4BP2L2-IT2	0.750721	3.97E-97	postive
ZNF337	ASMTL-AS1	0.720529	5.36E-86	postive
ZNF337	LINC00893	0.755793	3.72E-99	postive
ZNF337	LINC00174	0.804468	########	postive
HOMEZ	DHRS4-AS1	0.639094	3.48E-62	postive
ZNF431	LINC00265	0.724538	2.18E-87	postive
PBX4	RFPL3S	0.612047	8.66E-56	postive
PBX4	C9orf139	0.603239	7.76E-54	postive
PBX4	DPP9-AS1	0.620284	1.14E-57	postive
PBX4	SNHG3	0.681264	1.51E-73	postive
PBX4	LINC00115	0.608933	4.31E-55	postive

PBX4	FAM13A-AS1	0.630473	4.46E-60	postive
PBX4	PVT1	0.632966	1.11E-60	postive
PBX4	LINC00174	0.629151	9.25E-60	postive
ZNF446	SCGB1B2P	0.613356	4.39E-56	postive
ZNF808	BMS1P4	0.613498	4.08E-56	postive
ZNF808	LINC00894	0.615697	1.29E-56	postive
ZNF808	GABPB1-AS1	0.605408	2.60E-54	postive
ZNF808	INE1	0.625031	8.82E-59	postive
ZNF808	LINC00265	0.61285	5.71E-56	postive
ZNF808	N4BP2L2-IT2	0.609532	3.17E-55	postive
BATF2	PCED1B-AS1	0.617543	4.88E-57	postive
BCL11B	PCED1B-AS1	0.778868	########	postive
ZNF266	BMS1P4	0.632847	1.19E-60	postive
ZNF266	C9orf139	0.609826	2.73E-55	postive
ZNF266	PDXDC2P-NPIPB14P	0.649932	6.22E-65	postive
ZNF266	HCG27	0.611033	1.46E-55	postive
ZNF266	LINC00894	0.600566	2.95E-53	postive
ZNF266	ARHGAP27P1-BPTFP1-KPNA2P3	0.754146	1.72E-98	postive
ZNF266	LINC00115	0.616405	8.89E-57	postive
ZNF266	FAM13A-AS1	0.661729	4.71E-68	postive
ZNF266	SNHG20	0.638057	6.28E-62	postive
ZNF266	INE1	0.666163	2.90E-69	postive
ZNF266	N4BP2L2-IT2	0.677762	1.57E-72	postive
ZNF266	ASMTL-AS1	0.669786	2.87E-70	postive
ZNF266	H1-10-AS1	0.647725	2.30E-64	postive
ZNF266	LINC00893	0.693915	2.44E-77	postive
ZNF266	LINC00174	0.647101	3.33E-64	postive
STAT5A	PCED1B-AS1	0.645872	6.85E-64	postive
ZNF814	RFPL3S	0.630118	5.42E-60	postive
ZNF814	BMS1P4	0.644173	1.85E-63	postive
ZNF814	C9orf139	0.620371	1.09E-57	postive
ZNF814	PDXDC2P-NPIPB14P	0.704009	1.65E-80	postive
ZNF814	LINC00894	0.654611	3.74E-66	postive
ZNF814	DPP9-AS1	0.699321	5.09E-79	postive
ZNF814	IBA57-DT	0.632174	1.73E-60	postive
ZNF814	MIRLET7BHG	0.606031	1.89E-54	postive
ZNF814	GABPB1-AS1	0.658372	3.76E-67	postive
ZNF814	ARHGAP27P1-BPTFP1-KPNA2P3	0.714633	5.41E-84	postive
ZNF814	LINC00115	0.628788	1.13E-59	postive

ZNF814	FAM13A-AS1	0.650987	3.31E-65	postive
ZNF814	SNHG20	0.619033	2.21E-57	postive
ZNF814	INE1	0.668668	5.88E-70	postive
ZNF814	C3orf35	0.646742	4.11E-64	postive
ZNF814	MALAT1	0.630092	5.50E-60	postive
ZNF814	N4BP2L2-IT2	0.671074	1.25E-70	postive
ZNF814	ASMTL-AS1	0.671466	9.71E-71	postive
ZNF814	H1-10-AS1	0.640637	1.43E-62	postive
ZNF814	LINC00893	0.686851	3.38E-75	postive
ZNF814	LINC00174	0.705969	3.86E-81	postive
AC008770.3	BMS1P4	0.657191	7.76E-67	postive
AC008770.3	GABPB1-AS1	0.641959	6.69E-63	postive
AC008770.3	INE1	0.638107	6.11E-62	postive
MTERF2	GUSBP11	0.650553	4.29E-65	postive
MTERF2	PDXDC2P-NPIPB14P	0.650083	5.68E-65	postive
MTERF2	HCG27	0.601573	1.79E-53	postive
MTERF2	LINC00894	0.609231	3.70E-55	postive
MTERF2	LINC00173	0.625647	6.31E-59	postive
MTERF2	ARHGAP27P1-BPTFP1-KPNA2P3	0.634066	6.01E-61	postive
MTERF2	FAM13A-AS1	0.626933	3.13E-59	postive
MTERF2	SNHG20	0.612237	7.85E-56	postive
MTERF2	ASMTL-AS1	0.602433	1.16E-53	postive
MTERF2	LINC00893	0.655585	2.07E-66	postive
MTERF2	LINC00174	0.607031	1.14E-54	postive
GTF2IRD2B	LINC00173	0.604463	4.19E-54	postive
GTF2IRD2B	SNHG20	0.600792	2.64E-53	postive
GTF2IRD2B	LINC00174	0.609032	4.10E-55	postive
ZNF226	PDXDC2P-NPIPB14P	0.617276	5.62E-57	postive
ZNF226	LINC00894	0.620345	1.10E-57	postive
ZNF226	GABPB1-AS1	0.600601	2.90E-53	postive
ZNF226	INE1	0.622824	2.91E-58	postive
ZNF226	N4BP2L2-IT2	0.623849	1.67E-58	postive
ZNF226	LINC00893	0.642532	4.80E-63	postive
ZNF891	BMS1P4	0.642225	5.74E-63	postive
ZNF891	GABPB1-AS1	0.643018	3.62E-63	postive
ZNF891	N4BP2L2-IT2	0.603112	8.27E-54	postive
ZNF513	GUSBP11	0.603059	8.49E-54	postive
ZNF513	ARHGAP27P1-BPTFP1-KPNA2P3	0.602511	1.12E-53	postive
ZNF513	ASMTL-AS1	0.629927	6.03E-60	postive

ZNF133	PDXDC2P-NPIPB14P	0.654657	3.63E-66	postive
ZNF133	HCG27	0.626364	4.27E-59	postive
ZNF133	IBA57-DT	0.612423	7.13E-56	postive
ZNF133	ARHGAP27P1-BPTFP1-KPNA2P3	0.691661	1.20E-76	postive
ZNF133	LINC00115	0.668014	8.93E-70	postive
ZNF133	FAM13A-AS1	0.633237	9.56E-61	postive
ZNF133	SNHG20	0.665346	4.86E-69	postive
ZNF133	C3orf35	0.6304	4.64E-60	postive
ZNF133	N4BP2L2-IT2	0.65366	6.64E-66	postive
ZNF133	ASMTL-AS1	0.673392	2.78E-71	postive
ZNF133	H1-10-AS1	0.653643	6.71E-66	postive
ZNF133	LINC00893	0.657338	7.09E-67	postive
ZNF133	LINC00174	0.707573	1.17E-81	postive
ZBED2	PCED1B-AS1	0.704816	9.10E-81	postive
ZNF169	RFPL3S	0.617724	4.43E-57	postive
ZNF169	GUSBP11	0.633341	9.02E-61	postive
ZNF169	BMS1P4	0.811369	########	postive
ZNF169	C9orf139	0.71817	3.45E-85	postive
ZNF169	PDXDC2P-NPIPB14P	0.757819	########	postive
ZNF169	HCG27	0.733015	2.05E-90	postive
ZNF169	LINC00894	0.762457	########	postive
ZNF169	DPP9-AS1	0.669177	4.24E-70	postive
ZNF169	LINC01126	0.655554	2.11E-66	postive
ZNF169	IBA57-DT	0.619683	1.57E-57	postive
ZNF169	DTX2P1-UPK3BP1-PMS2P11	0.650304	4.98E-65	postive
ZNF169	SNHG4	0.614945	1.91E-56	postive
ZNF169	LINC00173	0.649858	6.50E-65	postive
ZNF169	GABPB1-AS1	0.774284	########	postive
ZNF169	SSBP3-AS1	0.674819	1.09E-71	postive
ZNF169	ARHGAP27P1-BPTFP1-KPNA2P3	0.775094	########	postive
ZNF169	LINC00115	0.710363	1.42E-82	postive
ZNF169	FAM13A-AS1	0.711641	5.38E-83	postive
ZNF169	SNHG20	0.730293	1.97E-89	postive
ZNF169	INE1	0.752169	1.06E-97	postive
ZNF169	MALAT1	0.675778	5.82E-72	postive
ZNF169	N4BP2L2-IT2	0.768234	########	postive
ZNF169	ASMTL-AS1	0.675551	6.76E-72	postive
ZNF169	H1-10-AS1	0.601131	2.23E-53	postive
ZNF169	LINC00893	0.766802	########	postive

ZNF169	LINC00174	0.729984	2.55E-89	postive
ZNF419	PDXDC2P-NPIPB14P	0.613167	4.84E-56	postive
ZNF419	GABPB1-AS1	0.602971	8.88E-54	postive
ZNF419	ARHGAP27P1-BPTFP1-KPNA2P3	0.628438	1.37E-59	postive
ZNF419	LINC00893	0.614168	2.87E-56	postive
HINFP	PDXDC2P-NPIPB14P	0.621323	6.52E-58	postive
HINFP	HCG27	0.711058	8.39E-83	postive
HINFP	LINC00173	0.649937	6.20E-65	postive
HINFP	ARHGAP27P1-BPTFP1-KPNA2P3	0.605524	2.45E-54	postive
HINFP	LINC00115	0.665433	4.60E-69	postive
HINFP	SNHG20	0.68274	5.59E-74	postive
HINFP	H1-10-AS1	0.606289	1.66E-54	postive
HINFP	LINC00893	0.669584	3.27E-70	postive
HINFP	LINC00174	0.605588	2.37E-54	postive
E4F1	RFPL3S	0.653878	5.82E-66	postive
E4F1	GUSBP11	0.65017	5.40E-65	postive
E4F1	PDXDC2P-NPIPB14P	0.697301	2.18E-78	postive
E4F1	LINC00894	0.636708	1.35E-61	postive
E4F1	DPP9-AS1	0.643718	2.41E-63	postive
E4F1	IBA57-DT	0.610439	1.99E-55	postive
E4F1	ARHGAP27P1-BPTFP1-KPNA2P3	0.677553	1.80E-72	postive
E4F1	LINC00115	0.717944	4.12E-85	postive
E4F1	FAM13A-AS1	0.602835	9.50E-54	postive
E4F1	SNHG20	0.62831	1.47E-59	postive
E4F1	SNHG17	0.650383	4.75E-65	postive
E4F1	C3orf35	0.634536	4.61E-61	postive
E4F1	ASMTL-AS1	0.737911	3.23E-92	postive
E4F1	SCGB1B2P	0.652822	1.10E-65	postive
E4F1	H1-10-AS1	0.714949	4.24E-84	postive
E4F1	LINC00893	0.68114	1.64E-73	postive
E4F1	LINC00174	0.703776	1.96E-80	postive
ZNF655	BMS1P4	0.63677	1.31E-61	postive
ZNF655	GABPB1-AS1	0.60545	2.54E-54	postive
ZNF655	INE1	0.613293	4.53E-56	postive
IKZF1	PCED1B-AS1	0.796598	########	postive
ZNF335	RFPL3S	0.679357	5.43E-73	postive
ZNF335	GUSBP11	0.620166	1.21E-57	postive
ZNF335	BMS1P4	0.666819	1.91E-69	postive

ZNF335	C9orf139	0.660502	1.01E-67	postive
ZNF335	PDXDC2P-NPIPB14P	0.762255	########	postive
ZNF335	HCG27	0.619381	1.84E-57	postive
ZNF335	LINC00894	0.643878	2.20E-63	postive
ZNF335	DPP9-AS1	0.658031	4.63E-67	postive
ZNF335	IBA57-DT	0.606172	1.76E-54	postive
ZNF335	DTX2P1-UPK3BP1-PMS2P11	0.654441	4.14E-66	postive
ZNF335	SNHG3	0.657667	5.80E-67	postive
ZNF335	ARHGAP27P1-BPTFP1-KPNA2P3	0.759252	########	postive
ZNF335	LINC00115	0.708873	4.39E-82	postive
ZNF335	FAM13A-AS1	0.665485	4.45E-69	postive
ZNF335	SNHG20	0.67116	1.18E-70	postive
ZNF335	INE1	0.721869	1.85E-86	postive
ZNF335	C3orf35	0.609521	3.19E-55	postive
ZNF335	LINC00265	0.603324	7.43E-54	postive
ZNF335	N4BP2L2-IT2	0.632819	1.21E-60	postive
ZNF335	ASMTL-AS1	0.75507	7.29E-99	postive
ZNF335	H1-10-AS1	0.689312	6.17E-76	postive
ZNF335	LINC00893	0.733098	1.91E-90	postive
ZNF335	LINC00174	0.734102	8.21E-91	postive
IKZF3	PCED1B-AS1	0.825415	########	postive
ZNF708	BMS1P4	0.623272	2.29E-58	postive
MEF2B	RFPL3S	0.690433	2.83E-76	postive
MEF2B	BMS1P4	0.609544	3.15E-55	postive
MEF2B	C9orf139	0.673782	2.16E-71	postive
MEF2B	PDXDC2P-NPIPB14P	0.622997	2.65E-58	postive
MEF2B	RRN3P2	0.603556	6.62E-54	postive
MEF2B	LINC00894	0.610918	1.55E-55	postive
MEF2B	DPP9-AS1	0.629277	8.63E-60	postive
MEF2B	SNHG3	0.615735	1.26E-56	postive
MEF2B	ARHGAP27P1-BPTFP1-KPNA2P3	0.671636	8.70E-71	postive
MEF2B	LINC00115	0.658279	3.98E-67	postive
MEF2B	C3orf35	0.648498	1.46E-64	postive
MEF2B	N4BP2L2-IT2	0.617281	5.60E-57	postive
MEF2B	ASMTL-AS1	0.667085	1.61E-69	postive
MEF2B	H1-10-AS1	0.707851	9.46E-82	postive
MEF2B	LINC00893	0.622375	3.71E-58	postive
MEF2B	LINC00174	0.668141	8.24E-70	postive
ZNF251	RFPL3S	0.630918	3.48E-60	postive

ZNF251	GUSBP11	0.613515	4.04E-56	postive
ZNF251	BMS1P4	0.676842	2.89E-72	postive
ZNF251	C9orf139	0.608378	5.73E-55	postive
ZNF251	PDXDC2P-NPIPB14P	0.710608	1.18E-82	postive
ZNF251	LINC02656	0.661298	6.15E-68	postive
ZNF251	HCG27	0.67018	2.23E-70	postive
ZNF251	LINC00894	0.651108	3.08E-65	postive
ZNF251	IBA57-DT	0.624751	1.03E-58	postive
ZNF251	DTX2P1-UPK3BP1-PMS2P11	0.602525	1.11E-53	postive
ZNF251	GABPB1-AS1	0.612414	7.16E-56	postive
ZNF251	SNHG3	0.676362	3.96E-72	postive
ZNF251	SSBP3-AS1	0.62218	4.12E-58	postive
ZNF251	ARHGAP27P1-BPTFP1-KPNA2P3	0.766076	########	postive
ZNF251	LINC00115	0.738707	1.63E-92	postive
ZNF251	FAM13A-AS1	0.741422	1.56E-93	postive
ZNF251	SNHG20	0.659053	2.47E-67	postive
ZNF251	INE1	0.636206	1.80E-61	postive
ZNF251	C3orf35	0.66049	1.02E-67	postive
ZNF251	N4BP2L2-IT2	0.707386	1.34E-81	postive
ZNF251	ASMTL-AS1	0.674214	1.62E-71	postive
ZNF251	H1-10-AS1	0.675571	6.67E-72	postive
ZNF251	LINC00893	0.703952	1.72E-80	postive
ZNF251	LINC00174	0.704259	1.37E-80	postive
ZBTB17	ARHGAP27P1-BPTFP1-KPNA2P3	0.624413	1.23E-58	postive
ZBTB17	LINC00115	0.660567	9.69E-68	postive
ZBTB17	SNHG20	0.613354	4.39E-56	postive
ZBTB17	ASMTL-AS1	0.614047	3.06E-56	postive
ZBTB17	H1-10-AS1	0.683984	2.40E-74	postive
ZNF831	PCED1B-AS1	0.766694	########	postive
CREBZF	BMS1P4	0.721807	1.94E-86	postive
CREBZF	PDXDC2P-NPIPB14P	0.692125	8.63E-77	postive
CREBZF	HCG27	0.685938	6.33E-75	postive
CREBZF	LINC00894	0.67625	4.27E-72	postive
CREBZF	LINC01126	0.656713	1.04E-66	postive
CREBZF	DTX2P1-UPK3BP1-PMS2P11	0.608931	4.32E-55	postive
CREBZF	LINC00173	0.617733	4.41E-57	postive
CREBZF	GABPB1-AS1	0.671237	1.13E-70	postive
CREBZF	SSBP3-AS1	0.60349	6.84E-54	postive

CREBZF	ARHGAP27P1-BPTFP1-KPNA2P3	0.68893	8.04E-76	postive
CREBZF	LINC00115	0.642381	5.24E-63	postive
CREBZF	FAM13A-AS1	0.667286	1.42E-69	postive
CREBZF	SNHG20	0.668494	6.57E-70	postive
CREBZF	INE1	0.682309	7.48E-74	postive
CREBZF	MALAT1	0.65971	1.65E-67	postive
CREBZF	N4BP2L2-IT2	0.706412	2.78E-81	postive
CREBZF	ASMTL-AS1	0.61171	1.03E-55	postive
CREBZF	LINC00893	0.701681	9.14E-80	postive
CREBZF	LINC00174	0.627996	1.75E-59	postive
ZNF7	BMS1P4	0.65714	8.01E-67	postive
ZNF7	PDXDC2P-NPIPB14P	0.699124	5.87E-79	postive
ZNF7	LINC00894	0.605039	3.13E-54	postive
ZNF7	GABPB1-AS1	0.608987	4.20E-55	postive
ZNF7	SNHG3	0.683562	3.20E-74	postive
ZNF7	ARHGAP27P1-BPTFP1-KPNA2P3	0.695721	6.77E-78	postive
ZNF7	LINC00115	0.678833	7.70E-73	postive
ZNF7	FAM13A-AS1	0.662362	3.17E-68	postive
ZNF7	SNHG20	0.666006	3.20E-69	postive
ZNF7	INE1	0.629015	9.97E-60	postive
ZNF7	N4BP2L2-IT2	0.645607	8.01E-64	postive
ZNF7	ASMTL-AS1	0.628466	1.35E-59	postive
ZNF7	H1-10-AS1	0.618493	2.95E-57	postive
ZNF7	LINC00893	0.661472	5.52E-68	postive
ZNF7	LINC00174	0.608798	4.62E-55	postive
ZNF160	BMS1P4	0.630589	4.18E-60	postive
ZNF160	PDXDC2P-NPIPB14P	0.647014	3.50E-64	postive
ZNF160	LINC02656	0.610227	2.22E-55	postive
ZNF160	HCG27	0.62263	3.23E-58	postive
ZNF160	SSBP3-AS1	0.663505	1.55E-68	postive
ZNF160	ARHGAP27P1-BPTFP1-KPNA2P3	0.696883	2.95E-78	postive
ZNF160	FAM13A-AS1	0.656569	1.13E-66	postive
ZNF160	SNHG20	0.668598	6.15E-70	postive
ZNF160	INE1	0.663825	1.27E-68	postive
ZNF160	MALAT1	0.612186	8.06E-56	postive
ZNF160	N4BP2L2-IT2	0.678567	9.20E-73	postive
ZNF160	LINC00893	0.6237	1.81E-58	postive
POU2F2	PCED1B-AS1	0.753041	4.75E-98	postive

ZBTB25	BMS1P4	0.653102	9.31E-66	postive
ZBTB25	PDXDC2P-NPIPB14P	0.628664	1.21E-59	postive
ZBTB25	HCG27	0.646175	5.74E-64	postive
ZBTB25	LINC00173	0.64724	3.07E-64	postive
ZBTB25	GABPB1-AS1	0.668282	7.53E-70	postive
ZBTB25	SSBP3-AS1	0.603942	5.45E-54	postive
ZBTB25	ARHGAP27P1-BPTFP1-KPNA2P3	0.640021	2.04E-62	postive
ZBTB25	LINC00115	0.639947	2.13E-62	postive
ZBTB25	FAM13A-AS1	0.654802	3.33E-66	postive
ZBTB25	SNHG20	0.664614	7.72E-69	postive
ZBTB25	INE1	0.614007	3.13E-56	postive
ZBTB25	N4BP2L2-IT2	0.706776	2.12E-81	postive
ZBTB25	LINC00174	0.605209	2.87E-54	postive
IRF3	RFPL3S	0.682075	8.76E-74	postive
IRF3	C9orf139	0.651341	2.68E-65	postive
IRF3	PDXDC2P-NPIPB14P	0.659872	1.49E-67	postive
IRF3	DPP9-AS1	0.666465	2.39E-69	postive
IRF3	SNHG3	0.659368	2.03E-67	postive
IRF3	ARHGAP27P1-BPTFP1-KPNA2P3	0.655707	1.92E-66	postive
IRF3	LINC00115	0.665263	5.12E-69	postive
IRF3	FAM13A-AS1	0.610172	2.28E-55	postive
IRF3	SNHG20	0.605384	2.63E-54	postive
IRF3	SNHG17	0.616555	8.21E-57	postive
IRF3	C3orf35	0.623077	2.54E-58	postive
IRF3	ASMTL-AS1	0.692949	4.84E-77	postive
IRF3	SCGB1B2P	0.62871	1.18E-59	postive
IRF3	H1-10-AS1	0.678654	8.68E-73	postive
IRF3	LINC00893	0.651583	2.32E-65	postive
IRF3	LINC00174	0.668428	6.86E-70	postive
STAT4	RRN3P2	0.627154	2.77E-59	postive
STAT4	PCED1B-AS1	0.701777	8.52E-80	postive
ZBTB32	RRN3P2	0.629787	6.51E-60	postive
ZBTB32	MIAT	0.618527	2.90E-57	postive
PROX2	SSBP3-AS1	0.603818	5.80E-54	postive
PROX2	N4BP2L2-IT2	0.613673	3.72E-56	postive
ZNF605	BMS1P4	0.60452	4.07E-54	postive
MEIS3	AGAP2-AS1	0.600683	2.78E-53	postive
MEIS3	MIR4435-2HG	0.629671	6.95E-60	postive
STAT2	BMS1P4	0.637335	9.48E-62	postive

STAT2	C9orf139	0.619888	1.40E-57	postive
ZNF10	PDXDC2P-NPIPB14P	0.607631	8.39E-55	postive
ZNF10	INE1	0.600748	2.70E-53	postive
ZNF10	LINC00265	0.600863	2.55E-53	postive
ZGLP1	RFPL3S	0.70805	8.15E-82	postive
ZGLP1	GUSBP11	0.621097	7.36E-58	postive
ZGLP1	C9orf139	0.671638	8.69E-71	postive
ZGLP1	PDXDC2P-NPIPB14P	0.642837	4.02E-63	postive
ZGLP1	LINC00894	0.62573	6.03E-59	postive
ZGLP1	DPP9-AS1	0.696416	4.12E-78	postive
ZGLP1	IBA57-DT	0.619192	2.03E-57	postive
ZGLP1	SNHG3	0.637014	1.14E-61	postive
ZGLP1	ARHGAP27P1-BPTFP1-KPNA2P3	0.705497	5.49E-81	postive
ZGLP1	LINC00115	0.683927	2.50E-74	postive
ZGLP1	FAM13A-AS1	0.606134	1.80E-54	postive
ZGLP1	SNHG17	0.618084	3.66E-57	postive
ZGLP1	C3orf35	0.658525	3.42E-67	postive
ZGLP1	N4BP2L2-IT2	0.607038	1.14E-54	postive
ZGLP1	ASMTL-AS1	0.710221	1.58E-82	postive
ZGLP1	H1-10-AS1	0.719268	1.45E-85	postive
ZGLP1	LINC00893	0.661638	4.98E-68	postive
ZGLP1	LINC00174	0.701596	9.73E-80	postive
ANKZF1	RFPL3S	0.640756	1.34E-62	postive
ANKZF1	GUSBP11	0.695958	5.71E-78	postive
ANKZF1	BMS1P4	0.714526	5.88E-84	postive
ANKZF1	C9orf139	0.689778	4.46E-76	postive
ANKZF1	PDXDC2P-NPIPB14P	0.711312	6.91E-83	postive
ANKZF1	LINC02656	0.664372	8.99E-69	postive
ANKZF1	HCG27	0.732523	3.09E-90	postive
ANKZF1	LINC00894	0.659902	1.46E-67	postive
ANKZF1	DPP9-AS1	0.640602	1.46E-62	postive
ANKZF1	LINC01126	0.613537	3.99E-56	postive
ANKZF1	DTX2P1-UPK3BP1-PMS2P11	0.631278	2.85E-60	postive
ANKZF1	LINC00173	0.667858	9.86E-70	postive
ANKZF1	GABPB1-AS1	0.60776	7.86E-55	postive
ANKZF1	SNHG3	0.653618	6.82E-66	postive
ANKZF1	ARHGAP27P1-BPTFP1-KPNA2P3	0.753666	2.67E-98	postive
ANKZF1	LINC00115	0.727235	2.44E-88	postive
ANKZF1	FAM13A-AS1	0.758217	########	postive

ANKZF1	SNHG20	0.689658	4.85E-76	postive
ANKZF1	INE1	0.609625	3.02E-55	postive
ANKZF1	C3orf35	0.646739	4.12E-64	postive
ANKZF1	N4BP2L2-IT2	0.681972	9.39E-74	postive
ANKZF1	PVT1	0.606349	1.61E-54	postive
ANKZF1	ASMTL-AS1	0.711839	4.63E-83	postive
ANKZF1	H1-10-AS1	0.607296	9.96E-55	postive
ANKZF1	LINC00893	0.717413	6.24E-85	postive
ANKZF1	LINC00174	0.793437	########	postive
ZNF558	FAM13A-AS1	0.614498	2.42E-56	postive
ZNF558	LINC00174	0.60497	3.24E-54	postive
ZNF785	BMS1P4	0.643068	3.52E-63	postive
ZNF785	PDXDC2P-NPIPB14P	0.715828	2.14E-84	postive
ZNF785	HCG27	0.690459	2.77E-76	postive
ZNF785	LINC00894	0.657093	8.24E-67	postive
ZNF785	LINC01126	0.604615	3.88E-54	postive
ZNF785	LINC00173	0.64936	8.74E-65	postive
ZNF785	GABPB1-AS1	0.663412	1.64E-68	postive
ZNF785	ARHGAP27P1-BPTFP1-KPNA2P3	0.682258	7.74E-74	postive
ZNF785	LINC00115	0.644643	1.41E-63	postive
ZNF785	FAM13A-AS1	0.635464	2.73E-61	postive
ZNF785	SNHG20	0.707586	1.15E-81	postive
ZNF785	INE1	0.620229	1.17E-57	postive
ZNF785	N4BP2L2-IT2	0.624303	1.31E-58	postive
ZNF785	ASMTL-AS1	0.622845	2.88E-58	postive
ZNF785	LINC00893	0.685346	9.50E-75	postive
ZNF785	LINC00174	0.678834	7.70E-73	postive
ZNF26	RFPL3S	0.757533	########	postive
ZNF26	GUSBP11	0.638213	5.75E-62	postive
ZNF26	BMS1P4	0.765102	########	postive
ZNF26	C9orf139	0.674097	1.75E-71	postive
ZNF26	PDXDC2P-NPIPB14P	0.816386	########	postive
ZNF26	RRN3P2	0.61886	2.43E-57	postive
ZNF26	LINC02656	0.650643	4.07E-65	postive
ZNF26	HCG27	0.645399	9.05E-64	postive
ZNF26	LINC00894	0.727893	1.42E-88	postive
ZNF26	DPP9-AS1	0.757424	########	postive
ZNF26	IBA57-DT	0.663561	1.50E-68	postive
ZNF26	DTX2P1-UPK3BP1-PMS2P11	0.690309	3.08E-76	postive
ZNF26	SNHG4	0.61866	2.70E-57	postive

ZNF26	LINC00173	0.652938	1.03E-65	postive
ZNF26	GABPB1-AS1	0.713573	1.23E-83	postive
ZNF26	SNHG3	0.721562	2.36E-86	postive
ZNF26	SSBP3-AS1	0.616522	8.36E-57	postive
ZNF26	ARHGAP27P1-BPTFP1-KPNA2P3	0.807215	########	postive
ZNF26	LINC00115	0.750393	5.35E-97	postive
ZNF26	FAM13A-AS1	0.779963	########	postive
ZNF26	SNHG20	0.68065	2.29E-73	postive
ZNF26	INE1	0.723	7.49E-87	postive
ZNF26	C3orf35	0.781109	########	postive
ZNF26	CDKN2B-AS1	0.612125	8.32E-56	postive
ZNF26	MALAT1	0.678746	8.16E-73	postive
ZNF26	N4BP2L2-IT2	0.770594	########	postive
ZNF26	ASMTL-AS1	0.744184	1.38E-94	postive
ZNF26	H1-10-AS1	0.686503	4.29E-75	postive
ZNF26	LINC00893	0.760545	########	postive
ZNF26	LINC00174	0.798533	########	postive
ZNF594	HCG27	0.621089	7.39E-58	postive
ZNF594	GABPB1-AS1	0.606747	1.32E-54	postive
ZNF594	SNHG20	0.631336	2.76E-60	postive
ZNF846	BMS1P4	0.622356	3.75E-58	postive
ZNF846	PDXDC2P-NPIPB14P	0.605419	2.58E-54	postive
ZNF846	GABPB1-AS1	0.619863	1.42E-57	postive
ZNF846	ARHGAP27P1-BPTFP1-KPNA2P3	0.647189	3.16E-64	postive
ZNF846	N4BP2L2-IT2	0.621268	6.72E-58	postive
ZNF846	LINC00893	0.613347	4.41E-56	postive
ZNF234	GABPB1-AS1	0.624073	1.48E-58	postive
SP140	MIAT	0.626427	4.12E-59	postive
SP140	PCED1B-AS1	0.868283	########	postive
MYSM1	BMS1P4	0.713175	1.66E-83	postive
MYSM1	PDXDC2P-NPIPB14P	0.625604	6.46E-59	postive
MYSM1	GABPB1-AS1	0.649976	6.06E-65	postive
MYSM1	SSBP3-AS1	0.626263	4.51E-59	postive
MYSM1	ARHGAP27P1-BPTFP1-KPNA2P3	0.650998	3.29E-65	postive
MYSM1	FAM13A-AS1	0.635173	3.22E-61	postive
MYSM1	INE1	0.647656	2.40E-64	postive
MYSM1	MALAT1	0.604517	4.08E-54	postive
MYSM1	N4BP2L2-IT2	0.668668	5.88E-70	postive

MYSM1	LINC00893	0.603437	7.02E-54	postive
ZNF621	BMS1P4	0.639951	2.13E-62	postive
ZNF621	PDXDC2P-NPIPB14P	0.605673	2.27E-54	postive
ZNF621	LINC00894	0.629119	9.42E-60	postive
ZNF621	GABPB1-AS1	0.627876	1.87E-59	postive
ZNF621	INE1	0.628575	1.27E-59	postive
ZNF621	LINC00265	0.614388	2.56E-56	postive
ZNF621	LINC00893	0.611663	1.06E-55	postive
ZNF528	BMS1P4	0.66535	4.85E-69	postive
ZNF528	PDXDC2P-NPIPB14P	0.617837	4.17E-57	postive
ZNF528	LINC00894	0.605564	2.40E-54	postive
ZNF528	GABPB1-AS1	0.620787	8.69E-58	postive
ZNF528	SSBP3-AS1	0.611001	1.49E-55	postive
ZNF528	ARHGAP27P1-BPTFP1-KPNA2P3	0.639011	3.65E-62	postive
ZNF528	FAM13A-AS1	0.61903	2.22E-57	postive
ZNF528	INE1	0.67606	4.84E-72	postive
ZNF528	N4BP2L2-IT2	0.637214	1.01E-61	postive
ZNF528	LINC00893	0.656957	8.95E-67	postive
ZNF528	LINC00174	0.615746	1.26E-56	postive
SAFB2	GUSBP11	0.6503	4.99E-65	postive
SAFB2	SNHG20	0.628853	1.09E-59	postive
ZNF80	RRN3P2	0.613388	4.32E-56	postive
SATB2	LHFPL3-AS2	0.643363	2.97E-63	postive
SNAI3	PCED1B-AS1	0.699212	5.51E-79	postive
ZNF546	BMS1P4	0.638993	3.68E-62	postive
ZNF546	HCG27	0.627793	1.95E-59	postive
ZNF546	LINC00173	0.605787	2.14E-54	postive
ZNF546	GABPB1-AS1	0.647396	2.80E-64	postive
ZNF546	ARHGAP27P1-BPTFP1-KPNA2P3	0.613404	4.28E-56	postive
ZNF546	FAM13A-AS1	0.642889	3.91E-63	postive
ZNF546	N4BP2L2-IT2	0.659748	1.61E-67	postive
EOMES	PCED1B-AS1	0.772308	########	postive
NEUROD1	WT1-AS	0.669754	2.93E-70	postive
ZNF841	RFPL3S	0.653241	8.55E-66	postive
ZNF841	BMS1P4	0.732149	4.22E-90	postive
ZNF841	PDXDC2P-NPIPB14P	0.766508	########	postive
ZNF841	LINC02656	0.643737	2.39E-63	postive
ZNF841	HCG27	0.637304	9.64E-62	postive
ZNF841	LINC00894	0.70998	1.90E-82	postive

ZNF841	DTX2P1-UPK3BP1-PMS2P11	0.637	1.15E-61	postive
ZNF841	GABPB1-AS1	0.682989	4.72E-74	postive
ZNF841	SNHG3	0.60201	1.44E-53	postive
ZNF841	SSBP3-AS1	0.729568	3.59E-89	postive
ZNF841	ARHGAP27P1-BPTFP1-KPNA2P3	0.729374	4.22E-89	postive
ZNF841	LINC00115	0.675049	9.40E-72	postive
ZNF841	FAM13A-AS1	0.701677	9.17E-80	postive
ZNF841	SNHG20	0.674584	1.28E-71	postive
ZNF841	INE1	0.802006	########	postive
ZNF841	C3orf35	0.620431	1.05E-57	postive
ZNF841	MALAT1	0.701289	1.22E-79	postive
ZNF841	N4BP2L2-IT2	0.744637	9.28E-95	postive
ZNF841	ASMTL-AS1	0.690007	3.80E-76	postive
ZNF841	H1-10-AS1	0.611408	1.21E-55	postive
ZNF841	LINC00893	0.730527	1.63E-89	postive
ZNF841	LINC00174	0.678753	8.13E-73	postive
SKOR1	GUSBP11	0.617196	5.86E-57	postive
SKOR1	PDXDC2P-NPIPB14P	0.623295	2.26E-58	postive
SKOR1	HCG27	0.600194	3.55E-53	postive
SKOR1	ARHGAP27P1-BPTFP1-KPNA2P3	0.699083	6.05E-79	postive
SKOR1	FAM13A-AS1	0.62272	3.08E-58	postive
SKOR1	SNHG20	0.633763	7.12E-61	postive
SKOR1	ASMTL-AS1	0.604513	4.08E-54	postive
SKOR1	H1-10-AS1	0.640645	1.43E-62	postive
SKOR1	LINC00893	0.619469	1.76E-57	postive
SKOR1	LINC00174	0.626487	3.99E-59	postive
ZC3H8	BMS1P4	0.68358	3.16E-74	postive
ZC3H8	PDXDC2P-NPIPB14P	0.666125	2.97E-69	postive
ZC3H8	HCG27	0.609743	2.85E-55	postive
ZC3H8	LINC00894	0.649225	9.47E-65	postive
ZC3H8	GABPB1-AS1	0.686666	3.84E-75	postive
ZC3H8	ARHGAP27P1-BPTFP1-KPNA2P3	0.652217	1.58E-65	postive
ZC3H8	LINC00115	0.623602	1.91E-58	postive
ZC3H8	FAM13A-AS1	0.624403	1.24E-58	postive
ZC3H8	SNHG20	0.654311	4.48E-66	postive
ZC3H8	INE1	0.616084	1.05E-56	postive
ZC3H8	N4BP2L2-IT2	0.659083	2.43E-67	postive
ZC3H8	LINC00893	0.640361	1.68E-62	postive

ZC3H8	LINC00174	0.637772	7.39E-62	postive
POU5F1	HCG27	0.684329	1.90E-74	postive
POU5F1	ARHGAP27P1-BPTFP1-KPNA2P3	0.636674	1.38E-61	postive
POU5F1	FAM13A-AS1	0.687879	1.66E-75	postive
POU5F1	SNHG20	0.613327	4.46E-56	postive
POU5F1	LINC00174	0.621533	5.83E-58	postive
SPI1	PCED1B-AS1	0.823255	########	postive
ZNF316	LINC00174	0.63675	1.32E-61	postive
AKNA	RRN3P2	0.606889	1.22E-54	postive
AKNA	PCED1B-AS1	0.794752	########	postive
ZSCAN9	GUSBP11	0.624141	1.43E-58	postive
ZSCAN9	BMS1P4	0.616052	1.07E-56	postive
ZSCAN9	PDXDC2P-NPIPB14P	0.626887	3.21E-59	postive
ZSCAN9	HCG27	0.610906	1.56E-55	postive
ZSCAN9	LINC00894	0.61235	7.41E-56	postive
ZSCAN9	ARHGAP27P1-BPTFP1-KPNA2P3	0.73702	6.92E-92	postive
ZSCAN9	SNHG20	0.660761	8.59E-68	postive
ZSCAN9	INE1	0.618834	2.46E-57	postive
ZSCAN9	N4BP2L2-IT2	0.615637	1.33E-56	postive
ZSCAN9	H1-10-AS1	0.62861	1.25E-59	postive
ZSCAN9	LINC00893	0.643242	3.18E-63	postive
ZSCAN9	LINC00174	0.61598	1.11E-56	postive
ZNF707	C9orf139	0.625934	5.40E-59	postive
ZNF707	ARHGAP27P1-BPTFP1-KPNA2P3	0.61369	3.69E-56	postive
ZNF707	H1-10-AS1	0.610371	2.06E-55	postive
DMRTA1	WDFY3-AS2	0.656549	1.15E-66	postive
ZNF783	BMS1P4	0.627898	1.84E-59	postive
ZNF783	PDXDC2P-NPIPB14P	0.657856	5.16E-67	postive
ZNF783	SNHG3	0.67489	1.04E-71	postive
ZNF783	ARHGAP27P1-BPTFP1-KPNA2P3	0.666936	1.77E-69	postive
ZNF783	LINC00115	0.64476	1.31E-63	postive
ZNF783	FAM13A-AS1	0.637247	9.96E-62	postive
ZNF783	N4BP2L2-IT2	0.621603	5.61E-58	postive
ZNF783	LINC00893	0.601712	1.67E-53	postive
ZNF783	LINC00174	0.671598	8.92E-71	postive
PPARA	CYTOR	-0.62179	5.07E-58	negative
ZNF75A	PDXDC2P-NPIPB14P	0.607588	8.58E-55	postive

ZNF333	BMS1P4	0.736054	1.58E-91	postive
ZNF333	PDXDC2P-NPIPB14P	0.688086	1.44E-75	postive
ZNF333	LINC02656	0.61136	1.24E-55	postive
ZNF333	HCG27	0.643391	2.92E-63	postive
ZNF333	LINC00894	0.677849	1.48E-72	postive
ZNF333	IBA57-DT	0.636153	1.85E-61	postive
ZNF333	LINC00173	0.612496	6.86E-56	postive
ZNF333	GABPB1-AS1	0.725558	9.54E-88	postive
ZNF333	SSBP3-AS1	0.669569	3.30E-70	postive
ZNF333	ARHGAP27P1-BPTFP1-KPNA2P3	0.788674	########	postive
ZNF333	LINC00115	0.648567	1.40E-64	postive
ZNF333	FAM13A-AS1	0.665198	5.34E-69	postive
ZNF333	SNHG20	0.741572	1.37E-93	postive
ZNF333	INE1	0.722244	1.37E-86	postive
ZNF333	MALAT1	0.634652	4.32E-61	postive
ZNF333	N4BP2L2-IT2	0.713941	9.23E-84	postive
ZNF333	ASMTL-AS1	0.634189	5.60E-61	postive
ZNF333	H1-10-AS1	0.674918	1.02E-71	postive
ZNF333	LINC00893	0.708011	8.39E-82	postive
ZNF333	LINC00174	0.656602	1.11E-66	postive
TAL2	WDFY3-AS2	0.652845	1.09E-65	postive
TAL2	LHFPL3-AS2	0.669966	2.56E-70	postive
NHLH1	GUSBP11	0.626748	3.46E-59	postive
ZNF302	HCG27	0.619351	1.87E-57	postive
ZNF417	GABPB1-AS1	0.604992	3.21E-54	postive
ZNF417	INE1	0.633191	9.81E-61	postive
ZNF397	GABPB1-AS1	0.611192	1.35E-55	postive
ZNF397	SNHG20	0.624812	9.93E-59	postive
ASCL2	PCED1B-AS1	0.7089	4.30E-82	postive
IRF1	PCED1B-AS1	0.679103	6.43E-73	postive
GFI1	RRN3P2	0.641208	1.03E-62	postive
GFI1	MIAT	0.638545	4.76E-62	postive
GFI1	PCED1B-AS1	0.816143	########	postive
ZNF529	BMS1P4	0.656243	1.38E-66	postive
ZNF529	PDXDC2P-NPIPB14P	0.662402	3.09E-68	postive
ZNF529	LINC00894	0.685742	7.24E-75	postive
ZNF529	GABPB1-AS1	0.696285	4.52E-78	postive
ZNF529	ARHGAP27P1-BPTFP1-KPNA2P3	0.650456	4.55E-65	postive
ZNF529	FAM13A-AS1	0.605997	1.93E-54	postive

ZNF529	SNHG20	0.618462	3.00E-57	postive
ZNF529	INE1	0.66156	5.23E-68	postive
ZNF529	MALAT1	0.630139	5.36E-60	postive
ZNF529	N4BP2L2-IT2	0.679119	6.36E-73	postive
ZNF529	LINC00893	0.666276	2.70E-69	postive
ZNF529	LINC00174	0.625627	6.38E-59	postive
ZNF121	RFPL3S	0.659775	1.58E-67	postive
ZNF121	BMS1P4	0.7218	1.95E-86	postive
ZNF121	PDXDC2P-NPIPB14P	0.718813	2.08E-85	postive
ZNF121	HCG27	0.614994	1.87E-56	postive
ZNF121	LINC00894	0.713221	1.61E-83	postive
ZNF121	DPP9-AS1	0.650729	3.87E-65	postive
ZNF121	GABPB1-AS1	0.705395	5.92E-81	postive
ZNF121	SNHG3	0.633535	8.09E-61	postive
ZNF121	ARHGAP27P1-BPTFP1-KPNA2P3	0.735651	2.22E-91	postive
ZNF121	LINC00115	0.64049	1.56E-62	postive
ZNF121	FAM13A-AS1	0.713088	1.78E-83	postive
ZNF121	SNHG20	0.647997	1.96E-64	postive
ZNF121	INE1	0.718917	1.92E-85	postive
ZNF121	C3orf35	0.614857	2.00E-56	postive
ZNF121	MALAT1	0.697593	1.77E-78	postive
ZNF121	N4BP2L2-IT2	0.757483	########	postive
ZNF121	ASMTL-AS1	0.651208	2.90E-65	postive
ZNF121	LINC00893	0.709217	3.38E-82	postive
ZNF121	LINC00174	0.687518	2.14E-75	postive
TBX19	RFPL3S	0.601366	1.98E-53	postive
TBX19	HCG27	0.628075	1.67E-59	postive
TBX19	DPP9-AS1	0.604026	5.22E-54	postive
TBX19	ARHGAP27P1-BPTFP1-KPNA2P3	0.632412	1.52E-60	postive
TBX19	FAM13A-AS1	0.663407	1.65E-68	postive
TBX19	PVT1	0.652061	1.74E-65	postive
TBX19	LINC00174	0.626137	4.83E-59	postive
IRF4	MIAT	0.607882	7.38E-55	postive
IRF4	PCED1B-AS1	0.742206	7.86E-94	postive
ZNF276	RFPL3S	0.750322	5.70E-97	postive
ZNF276	GUSBP11	0.746608	1.61E-95	postive
ZNF276	BMS1P4	0.805442	########	postive
ZNF276	C9orf139	0.75178	1.51E-97	postive
ZNF276	PDXDC2P-NPIPB14P	0.866548	########	postive

ZNF276	RRN3P2	0.730819	1.28E-89	postive
ZNF276	LINC02656	0.676929	2.73E-72	postive
ZNF276	HCG27	0.769339	########	postive
ZNF276	LINC00894	0.794159	########	postive
ZNF276	DPP9-AS1	0.738724	1.61E-92	postive
ZNF276	LINC01126	0.661316	6.08E-68	postive
ZNF276	IBA57-DT	0.662213	3.48E-68	postive
ZNF276	DTX2P1-UPK3BP1-PMS2P11	0.739528	8.04E-93	postive
ZNF276	LINC00173	0.656827	9.69E-67	postive
ZNF276	GABPB1-AS1	0.69212	8.67E-77	postive
ZNF276	SNHG3	0.709057	3.82E-82	postive
ZNF276	SSBP3-AS1	0.663899	1.21E-68	postive
ZNF276	ARHGAP27P1-BPTFP1-KPNA2P3	0.874978	########	postive
ZNF276	LINC00115	0.81341	########	postive
ZNF276	LINC00937	0.619039	2.21E-57	postive
ZNF276	FAM13A-AS1	0.794641	########	postive
ZNF276	SNHG20	0.759931	########	postive
ZNF276	INE1	0.7687	########	postive
ZNF276	C3orf35	0.690644	2.44E-76	postive
ZNF276	LINC00265	0.6267	3.55E-59	postive
ZNF276	MALAT1	0.716343	1.44E-84	postive
ZNF276	N4BP2L2-IT2	0.801097	########	postive
ZNF276	ASMTL-AS1	0.840853	########	postive
ZNF276	H1-10-AS1	0.727693	1.68E-88	postive
ZNF276	LINC00893	0.842272	########	postive
ZNF276	LINC00174	0.841422	########	postive
KMT2B	BMS1P4	0.601506	1.85E-53	postive
KMT2B	PDXDC2P-NPIPB14P	0.661445	5.61E-68	postive
KMT2B	ARHGAP27P1-BPTFP1-KPNA2P3	0.658212	4.15E-67	postive
KMT2B	LINC00115	0.616913	6.80E-57	postive
KMT2B	SNHG20	0.643431	2.85E-63	postive
KMT2B	INE1	0.654411	4.22E-66	postive
KMT2B	ASMTL-AS1	0.62456	1.14E-58	postive
KMT2B	H1-10-AS1	0.600972	2.41E-53	postive
KMT2B	LINC00893	0.629331	8.38E-60	postive
KMT2B	LINC00174	0.630932	3.45E-60	postive
TCF15	MIR4435-2HG	0.608885	4.42E-55	postive
ZNF182	BMS1P4	0.64249	4.92E-63	postive
ZNF182	PDXDC2P-NPIPB14P	0.637251	9.94E-62	postive

ZNF182	LINC00894	0.627522	2.27E-59	postive
ZNF182	ARHGAP27P1-BPTFP1-KPNA2P3	0.646017	6.30E-64	postive
ZNF182	FAM13A-AS1	0.641742	7.59E-63	postive
ZNF182	INE1	0.629558	7.39E-60	postive
ZNF182	N4BP2L2-IT2	0.672445	5.15E-71	postive
ZNF182	LINC00893	0.636997	1.15E-61	postive
ZNF182	LINC00174	0.634919	3.72E-61	postive
ZNF514	GUSBP11	0.621761	5.16E-58	postive
ZNF514	BMS1P4	0.73938	9.14E-93	postive
ZNF514	PDXDC2P-NPIPB14P	0.73808	2.80E-92	postive
ZNF514	LINC02656	0.623622	1.89E-58	postive
ZNF514	HCG27	0.684052	2.29E-74	postive
ZNF514	LINC00894	0.738214	2.49E-92	postive
ZNF514	LINC01126	0.675093	9.14E-72	postive
ZNF514	DTX2P1-UPK3BP1-PMS2P11	0.613514	4.04E-56	postive
ZNF514	LINC00173	0.669048	4.61E-70	postive
ZNF514	GABPB1-AS1	0.729462	3.92E-89	postive
ZNF514	SSBP3-AS1	0.64195	6.73E-63	postive
ZNF514	ARHGAP27P1-BPTFP1-KPNA2P3	0.714413	6.42E-84	postive
ZNF514	LINC00115	0.663368	1.69E-68	postive
ZNF514	FAM13A-AS1	0.694151	2.07E-77	postive
ZNF514	SNHG20	0.728214	1.09E-88	postive
ZNF514	INE1	0.728629	7.79E-89	postive
ZNF514	MALAT1	0.689401	5.80E-76	postive
ZNF514	N4BP2L2-IT2	0.719615	1.11E-85	postive
ZNF514	ASMTL-AS1	0.686412	4.57E-75	postive
ZNF514	LINC00893	0.744385	1.16E-94	postive
ZNF514	LINC00174	0.728817	6.67E-89	postive
DOT1L	PDXDC2P-NPIPB14P	0.64713	3.27E-64	postive
DOT1L	ARHGAP27P1-BPTFP1-KPNA2P3	0.679143	6.26E-73	postive
DOT1L	SNHG20	0.646268	5.43E-64	postive
DOT1L	INE1	0.675805	5.72E-72	postive
DOT1L	ASMTL-AS1	0.63021	5.15E-60	postive
DOT1L	LINC00893	0.623612	1.90E-58	postive
DMTF1	RFPL3S	0.665932	3.36E-69	postive
DMTF1	GUSBP11	0.712027	4.01E-83	postive
DMTF1	BMS1P4	0.827341	########	postive
DMTF1	C9orf139	0.650445	4.58E-65	postive

DMTF1	PDXDC2P-NPIPB14P	0.817058	########	postive
DMTF1	RRN3P2	0.608462	5.49E-55	postive
DMTF1	LINC02656	0.712263	3.34E-83	postive
DMTF1	HCG27	0.769412	########	postive
DMTF1	LINC00894	0.772932	########	postive
DMTF1	DPP9-AS1	0.634139	5.76E-61	postive
DMTF1	LINC01126	0.666857	1.87E-69	postive
DMTF1	IBA57-DT	0.611724	1.02E-55	postive
DMTF1	DTX2P1-UPK3BP1-PMS2P11	0.711227	7.37E-83	postive
DMTF1	LINC00173	0.681436	1.35E-73	postive
DMTF1	GABPB1-AS1	0.756121	2.74E-99	postive
DMTF1	SNHG3	0.652953	1.02E-65	postive
DMTF1	SSBP3-AS1	0.701765	8.59E-80	postive
DMTF1	ARHGAP27P1-BPTFP1-KPNA2P3	0.841144	########	postive
DMTF1	LINC00115	0.751273	2.40E-97	postive
DMTF1	FAM13A-AS1	0.807469	########	postive
DMTF1	SNHG20	0.74516	5.84E-95	postive
DMTF1	INE1	0.776902	########	postive
DMTF1	C3orf35	0.649526	7.92E-65	postive
DMTF1	MALAT1	0.741206	1.88E-93	postive
DMTF1	N4BP2L2-IT2	0.823183	########	postive
DMTF1	ASMTL-AS1	0.744593	9.65E-95	postive
DMTF1	H1-10-AS1	0.637607	8.12E-62	postive
DMTF1	LINC00893	0.794108	########	postive
DMTF1	LINC00174	0.810574	########	postive
ZNF518A	BMS1P4	0.662742	2.50E-68	postive
MTERF4	SNHG20	0.628969	1.02E-59	postive
PRDM15	RFPL3S	0.618357	3.17E-57	postive
PRDM15	BMS1P4	0.706412	2.78E-81	postive
PRDM15	C9orf139	0.664176	1.02E-68	postive
PRDM15	PDXDC2P-NPIPB14P	0.642036	6.40E-63	postive
PRDM15	DPP9-AS1	0.637747	7.50E-62	postive
PRDM15	GABPB1-AS1	0.625476	6.93E-59	postive
PRDM15	SNHG3	0.620178	1.20E-57	postive
PRDM15	ARHGAP27P1-BPTFP1-KPNA2P3	0.706663	2.30E-81	postive
PRDM15	LINC00115	0.601446	1.90E-53	postive
PRDM15	SNHG20	0.640729	1.36E-62	postive
PRDM15	INE1	0.637718	7.62E-62	postive
PRDM15	N4BP2L2-IT2	0.617516	4.95E-57	postive

PRDM15	H1-10-AS1	0.613995	3.15E-56	postive
PRDM15	LINC00174	0.606718	1.34E-54	postive
ZNF236	BMS1P4	0.604346	4.44E-54	postive
ZNF519	BMS1P4	0.610854	1.61E-55	postive
ZNF449	BMS1P4	0.692799	5.37E-77	postive
ZNF449	PDXDC2P-NPIPB14P	0.639129	3.41E-62	postive
ZNF449	LINC02656	0.613993	3.15E-56	postive
ZNF449	GABPB1-AS1	0.640026	2.04E-62	postive
ZNF449	SSBP3-AS1	0.634793	3.99E-61	postive
ZNF449	ARHGAP27P1-BPTFP1-KPNA2P3	0.709055	3.82E-82	postive
ZNF449	FAM13A-AS1	0.629628	7.11E-60	postive
ZNF449	SNHG20	0.618635	2.73E-57	postive
ZNF449	INE1	0.652407	1.41E-65	postive
ZNF449	MALAT1	0.616513	8.40E-57	postive
ZNF449	N4BP2L2-IT2	0.683576	3.17E-74	postive
ZNF449	LINC00893	0.626555	3.85E-59	postive
ZNF117	BMS1P4	0.702711	4.30E-80	postive
ZNF117	C9orf139	0.615544	1.40E-56	postive
ZNF117	PDXDC2P-NPIPB14P	0.645617	7.96E-64	postive
ZNF117	LINC00894	0.648561	1.40E-64	postive
ZNF117	DTX2P1-UPK3BP1-PMS2P11	0.612763	5.98E-56	postive
ZNF117	GABPB1-AS1	0.61474	2.13E-56	postive
ZNF117	ARHGAP27P1-BPTFP1-KPNA2P3	0.710511	1.27E-82	postive
ZNF117	LINC00115	0.606665	1.37E-54	postive
ZNF117	FAM13A-AS1	0.696248	4.65E-78	postive
ZNF117	INE1	0.631911	2.00E-60	postive
ZNF117	N4BP2L2-IT2	0.68417	2.12E-74	postive
ZNF117	LINC00893	0.645707	7.55E-64	postive
ZNF117	LINC00174	0.700572	2.05E-79	postive
ZNF76	GUSBP11	0.695419	8.39E-78	postive
ZNF76	PDXDC2P-NPIPB14P	0.606645	1.39E-54	postive
ZNF76	HCG27	0.657237	7.54E-67	postive
ZNF76	LINC00894	0.600521	3.02E-53	postive
ZNF76	ARHGAP27P1-BPTFP1-KPNA2P3	0.625784	5.86E-59	postive
ZNF76	SNHG20	0.656819	9.74E-67	postive
ZNF76	ASMTL-AS1	0.643221	3.22E-63	postive
ZNF76	LINC00893	0.67617	4.50E-72	postive
ZNF76	LINC00174	0.637408	9.09E-62	postive

SNAPC4	C9orf139	0.601205	2.15E-53	postive
SNAPC4	ARHGAP27P1-BPTFP1-KPNA2P3	0.663351	1.71E-68	postive
SNAPC4	LINC00115	0.632368	1.55E-60	postive
SNAPC4	SNHG20	0.66489	6.49E-69	postive
SNAPC4	ASMTL-AS1	0.615129	1.74E-56	postive
SNAPC4	H1-10-AS1	0.647515	2.61E-64	postive
SNAPC4	LINC00893	0.628454	1.36E-59	postive
SNAPC4	LINC00174	0.606326	1.63E-54	postive
ARID3B	C9orf139	0.60749	9.02E-55	postive
ARID3B	ARHGAP27P1-BPTFP1-KPNA2P3	0.602673	1.03E-53	postive
ZBED5	BMS1P4	0.613323	4.46E-56	postive
ZBED5	HCG27	0.610349	2.08E-55	postive
ZBED5	LINC00894	0.606953	1.19E-54	postive
ZBED5	N4BP2L2-IT2	0.602815	9.59E-54	postive
ZBED5	LINC00893	0.636703	1.36E-61	postive
SCML4	RRN3P2	0.636078	1.93E-61	postive
SCML4	MIAT	0.627504	2.29E-59	postive
SCML4	PCED1B-AS1	0.77077	########	postive
HSF4	GUSBP11	0.672609	4.63E-71	postive
HSF4	BMS1P4	0.614531	2.38E-56	postive
HSF4	PDXDC2P-NPIPB14P	0.707618	1.13E-81	postive
HSF4	LINC02656	0.661631	5.00E-68	postive
HSF4	HCG27	0.748575	2.77E-96	postive
HSF4	LINC00894	0.620752	8.86E-58	postive
HSF4	DPP9-AS1	0.641609	8.19E-63	postive
HSF4	IBA57-DT	0.607768	7.83E-55	postive
HSF4	LINC00173	0.783929	########	postive
HSF4	GABPB1-AS1	0.601003	2.37E-53	postive
HSF4	ARHGAP27P1-BPTFP1-KPNA2P3	0.724565	2.13E-87	postive
HSF4	LINC00115	0.71521	3.46E-84	postive
HSF4	FAM13A-AS1	0.75495	8.15E-99	postive
HSF4	SNHG20	0.695068	1.08E-77	postive
HSF4	C3orf35	0.610871	1.59E-55	postive
HSF4	MALAT1	0.638508	4.86E-62	postive
HSF4	N4BP2L2-IT2	0.692155	8.45E-77	postive
HSF4	PVT1	0.608695	4.87E-55	postive
HSF4	ASMTL-AS1	0.658215	4.14E-67	postive
HSF4	H1-10-AS1	0.617998	3.83E-57	postive

HSF4	LINC00893	0.665873	3.49E-69	postive
HSF4	LINC00174	0.746425	1.90E-95	postive
CEBPA	PCED1B-AS1	0.62772	2.03E-59	postive
AKAP8	HCG27	0.606667	1.37E-54	postive
AKAP8	LINC00173	0.606023	1.90E-54	postive
AKAP8	ARHGAP27P1-BPTFP1-KPNA2P3	0.637733	7.56E-62	postive
AKAP8	FAM13A-AS1	0.62022	1.18E-57	postive
AKAP8	SNHG20	0.650898	3.49E-65	postive
RXRB	SNHG20	0.603936	5.46E-54	postive
ZNF444	SCGB1B2P	0.667295	1.41E-69	postive
ZNF444	H1-10-AS1	0.619747	1.51E-57	postive
ZNF444	LINC00174	0.608048	6.79E-55	postive
CXXC1	GUSBP11	0.607389	9.50E-55	postive
CXXC1	HCG27	0.614129	2.93E-56	postive
CXXC1	SNHG20	0.67565	6.34E-72	postive
CXXC1	H1-10-AS1	0.614167	2.88E-56	postive
MZF1	RFPL3S	0.736011	1.63E-91	postive
MZF1	GUSBP11	0.744754	8.37E-95	postive
MZF1	BMS1P4	0.753049	4.72E-98	postive
MZF1	C9orf139	0.717116	7.86E-85	postive
MZF1	PDXDC2P-NPIPB14P	0.831488	########	postive
MZF1	LINC02656	0.660161	1.25E-67	postive
MZF1	HCG27	0.747785	5.63E-96	postive
MZF1	LINC00894	0.809186	########	postive
MZF1	DPP9-AS1	0.737185	6.02E-92	postive
MZF1	LINC01126	0.614874	1.99E-56	postive
MZF1	IBA57-DT	0.691447	1.39E-76	postive
MZF1	DTX2P1-UPK3BP1-PMS2P11	0.655819	1.79E-66	postive
MZF1	LINC00173	0.625438	7.07E-59	postive
MZF1	GABPB1-AS1	0.699674	3.94E-79	postive
MZF1	SNHG3	0.679828	3.96E-73	postive
MZF1	SSBP3-AS1	0.657801	5.34E-67	postive
MZF1	ARHGAP27P1-BPTFP1-KPNA2P3	0.836038	########	postive
MZF1	LINC00115	0.782649	########	postive
MZF1	FAM13A-AS1	0.785023	########	postive
MZF1	SNHG20	0.764769	########	postive
MZF1	INE1	0.788491	########	postive
MZF1	C3orf35	0.721035	3.59E-86	postive
MZF1	LINC00265	0.621093	7.38E-58	postive

MZF1	MALAT1	0.718508	2.64E-85	postive
MZF1	N4BP2L2-IT2	0.752625	6.97E-98	postive
MZF1	ASMTL-AS1	0.857247	########	postive
MZF1	SCGB1B2P	0.610437	1.99E-55	postive
MZF1	H1-10-AS1	0.724946	1.57E-87	postive
MZF1	LINC00893	0.858125	########	postive
MZF1	LINC00174	0.861752	########	postive
ZNF780B	BMS1P4	0.621146	7.17E-58	postive
ZNF780B	GABPB1-AS1	0.604889	3.38E-54	postive
ZNF780B	INE1	0.604538	4.03E-54	postive
ZGPAT	GUSBP11	0.671315	1.07E-70	postive
ZGPAT	PDXDC2P-NPIPB14P	0.680318	2.86E-73	postive
ZGPAT	LINC00894	0.609456	3.30E-55	postive
ZGPAT	DPP9-AS1	0.612036	8.71E-56	postive
ZGPAT	ARHGAP27P1-BPTFP1-KPNA2P3	0.643056	3.55E-63	postive
ZGPAT	LINC00115	0.643505	2.73E-63	postive
ZGPAT	SNHG20	0.620854	8.38E-58	postive
ZGPAT	INE1	0.633836	6.83E-61	postive
ZGPAT	ASMTL-AS1	0.711693	5.17E-83	postive
ZGPAT	LINC00893	0.667945	9.33E-70	postive
ZGPAT	LINC00174	0.668782	5.47E-70	postive
ZNF683	PCED1B-AS1	0.654448	4.12E-66	postive
ZNF789	RFPL3S	0.68264	5.98E-74	postive
ZNF789	GUSBP11	0.778836	########	postive
ZNF789	BMS1P4	0.807557	########	postive
ZNF789	C9orf139	0.720674	4.78E-86	postive
ZNF789	PDXDC2P-NPIPB14P	0.827989	########	postive
ZNF789	LINC02656	0.657885	5.07E-67	postive
ZNF789	HCG27	0.760057	########	postive
ZNF789	LINC00894	0.782869	########	postive
ZNF789	DPP9-AS1	0.706741	2.17E-81	postive
ZNF789	LINC01126	0.63923	3.22E-62	postive
ZNF789	IBA57-DT	0.663147	1.94E-68	postive
ZNF789	DTX2P1-UPK3BP1-PMS2P11	0.679736	4.22E-73	postive
ZNF789	LINC00173	0.678789	7.93E-73	postive
ZNF789	GABPB1-AS1	0.753663	2.68E-98	postive
ZNF789	SNHG3	0.619302	1.92E-57	postive
ZNF789	SSBP3-AS1	0.664334	9.21E-69	postive
ZNF789	ARHGAP27P1-BPTFP1-KPNA2P3	0.831434	########	postive

ZNF789	LINC00115	0.758225	########	postive
ZNF789	FAM13A-AS1	0.779557	########	postive
ZNF789	SNHG20	0.758745	########	postive
ZNF789	INE1	0.767824	########	postive
ZNF789	C3orf35	0.698495	9.25E-79	postive
ZNF789	MALAT1	0.727268	2.37E-88	postive
ZNF789	N4BP2L2-IT2	0.773049	########	postive
ZNF789	ASMTL-AS1	0.795642	########	postive
ZNF789	H1-10-AS1	0.642315	5.45E-63	postive
ZNF789	LINC00893	0.818574	########	postive
ZNF789	LINC00174	0.87169	########	postive
NR2C1	BMS1P4	0.681718	1.11E-73	postive
NR2C1	PDXDC2P-NPIPB14P	0.67342	2.73E-71	postive
NR2C1	LINC00894	0.653376	7.89E-66	postive
NR2C1	ARHGAP27P1-BPTFP1-KPNA2P3	0.658098	4.45E-67	postive
NR2C1	LINC00115	0.604483	4.15E-54	postive
NR2C1	FAM13A-AS1	0.625207	8.02E-59	postive
NR2C1	INE1	0.604116	4.99E-54	postive
NR2C1	C3orf35	0.630782	3.75E-60	postive
NR2C1	N4BP2L2-IT2	0.639091	3.48E-62	postive
NR2C1	ASMTL-AS1	0.600865	2.54E-53	postive
NR2C1	LINC00893	0.651881	1.94E-65	postive
NR2C1	LINC00174	0.62632	4.37E-59	postive
L3MBTL1	RFPL3S	0.687031	2.99E-75	postive
L3MBTL1	GUSBP11	0.722559	1.07E-86	postive
L3MBTL1	BMS1P4	0.798323	########	postive
L3MBTL1	C9orf139	0.672706	4.35E-71	postive
L3MBTL1	PDXDC2P-NPIPB14P	0.848044	########	postive
L3MBTL1	LINC02656	0.681252	1.52E-73	postive
L3MBTL1	HCG27	0.758931	########	postive
L3MBTL1	LINC00894	0.845303	########	postive
L3MBTL1	DPP9-AS1	0.672634	4.55E-71	postive
L3MBTL1	LINC01126	0.694264	1.91E-77	postive
L3MBTL1	IBA57-DT	0.701407	1.12E-79	postive
L3MBTL1	DTX2P1-UPK3BP1-PMS2P11	0.702074	6.86E-80	postive
L3MBTL1	LINC00173	0.723097	6.93E-87	postive
L3MBTL1	GABPB1-AS1	0.746824	1.33E-95	postive
L3MBTL1	SNHG3	0.644562	1.48E-63	postive
L3MBTL1	SSBP3-AS1	0.711126	7.96E-83	postive

L3MBTL1	ARHGAP27P1-BPTFP1-KPNA2P3	0.812989	########	postive
L3MBTL1	LINC00115	0.782463	########	postive
L3MBTL1	FAM13A-AS1	0.781405	########	postive
L3MBTL1	SNHG20	0.761052	########	postive
L3MBTL1	INE1	0.79099	########	postive
L3MBTL1	C3orf35	0.699207	5.53E-79	postive
L3MBTL1	LINC00265	0.644286	1.73E-63	postive
L3MBTL1	MALAT1	0.75022	6.26E-97	postive
L3MBTL1	N4BP2L2-IT2	0.783338	########	postive
L3MBTL1	ASMTL-AS1	0.799566	########	postive
L3MBTL1	H1-10-AS1	0.67999	3.56E-73	postive
L3MBTL1	LINC00893	0.854823	########	postive
L3MBTL1	LINC00174	0.834788	########	postive
CENPT	RFPL3S	0.759797	########	postive
CENPT	GUSBP11	0.775667	########	postive
CENPT	BMS1P4	0.719317	1.40E-85	postive
CENPT	C9orf139	0.71465	5.34E-84	postive
CENPT	PDXDC2P-NPIPB14P	0.856766	########	postive
CENPT	LINC02656	0.653421	7.68E-66	postive
CENPT	HCG27	0.756776	1.49E-99	postive
CENPT	LINC00894	0.774998	########	postive
CENPT	DPP9-AS1	0.726965	3.04E-88	postive
CENPT	LINC01126	0.621923	4.73E-58	postive
CENPT	IBA57-DT	0.739349	9.39E-93	postive
CENPT	DTX2P1-UPK3BP1-PMS2P11	0.638184	5.85E-62	postive
CENPT	LINC00173	0.702364	5.55E-80	postive
CENPT	GABPB1-AS1	0.655879	1.73E-66	postive
CENPT	SNHG3	0.7211	3.41E-86	postive
CENPT	SSBP3-AS1	0.613895	3.31E-56	postive
CENPT	ARHGAP27P1-BPTFP1-KPNA2P3	0.857083	########	postive
CENPT	LINC00115	0.820339	########	postive
CENPT	FAM13A-AS1	0.765485	########	postive
CENPT	SNHG20	0.794799	########	postive
CENPT	SNHG17	0.627731	2.02E-59	postive
CENPT	INE1	0.750536	4.69E-97	postive
CENPT	C3orf35	0.738255	2.41E-92	postive
CENPT	MALAT1	0.664381	8.93E-69	postive
CENPT	N4BP2L2-IT2	0.737577	4.30E-92	postive
CENPT	ASMTL-AS1	0.855198	########	postive

CENPT	SCGB1B2P	0.646207	5.63E-64	postive
CENPT	H1-10-AS1	0.813894	########	postive
CENPT	LINC00893	0.836603	########	postive
CENPT	LINC00174	0.847834	########	postive
POU6F1	PDXDC2P-NPIPB14P	0.661213	6.48E-68	postive
POU6F1	ARHGAP27P1-BPTFP1-KPNA2P3	0.659639	1.72E-67	postive
POU6F1	FAM13A-AS1	0.610717	1.72E-55	postive
POU6F1	INE1	0.600836	2.58E-53	postive
POU6F1	N4BP2L2-IT2	0.605196	2.89E-54	postive
POU6F1	ASMTL-AS1	0.653705	6.47E-66	postive
POU6F1	H1-10-AS1	0.683003	4.68E-74	postive
POU6F1	LINC00893	0.669794	2.86E-70	postive
POU6F1	LINC00174	0.636202	1.80E-61	postive
ZMAT1	RFPL3S	0.605255	2.81E-54	postive
ZMAT1	GUSBP11	0.722891	8.17E-87	postive
ZMAT1	BMS1P4	0.730182	2.16E-89	postive
ZMAT1	PDXDC2P-NPIPB14P	0.756707	1.58E-99	postive
ZMAT1	LINC02656	0.695684	6.95E-78	postive
ZMAT1	HCG27	0.763067	########	postive
ZMAT1	LINC00894	0.768662	########	postive
ZMAT1	DPP9-AS1	0.605612	2.34E-54	postive
ZMAT1	LINC01126	0.646889	3.77E-64	postive
ZMAT1	DTX2P1-UPK3BP1-PMS2P11	0.627484	2.31E-59	postive
ZMAT1	LINC00173	0.686604	4.01E-75	postive
ZMAT1	GABPB1-AS1	0.657049	8.46E-67	postive
ZMAT1	SSBP3-AS1	0.633569	7.93E-61	postive
ZMAT1	ARHGAP27P1-BPTFP1-KPNA2P3	0.770638	########	postive
ZMAT1	LINC00115	0.703161	3.09E-80	postive
ZMAT1	FAM13A-AS1	0.807374	########	postive
ZMAT1	SNHG20	0.670569	1.73E-70	postive
ZMAT1	INE1	0.715069	3.86E-84	postive
ZMAT1	C3orf35	0.619656	1.59E-57	postive
ZMAT1	MALAT1	0.758332	########	postive
ZMAT1	N4BP2L2-IT2	0.77154	########	postive
ZMAT1	ASMTL-AS1	0.744186	1.38E-94	postive
ZMAT1	LINC00893	0.801994	########	postive
ZMAT1	LINC00174	0.806195	########	postive
GLI4	LINC00115	0.622198	4.08E-58	postive
GLI4	C3orf35	0.616495	8.48E-57	postive

GLI4	ASMTL-AS1	0.660729	8.76E-68	postive
GLI4	SCGB1B2P	0.62548	6.91E-59	postive
GLI4	LINC00174	0.6792	6.03E-73	postive
ZNF202	BMS1P4	0.602702	1.02E-53	postive
ZNF202	PDXDC2P-NPIPB14P	0.623539	1.98E-58	postive
ZNF202	ARHGAP27P1-BPTFP1-KPNA2P3	0.680855	1.99E-73	postive
ZNF202	SNHG20	0.657431	6.70E-67	postive
ZNF202	INE1	0.607582	8.61E-55	postive
ZNF202	N4BP2L2-IT2	0.634828	3.91E-61	postive
ZNF202	LINC00893	0.60416	4.88E-54	postive
ZNF341	C9orf139	0.611895	9.37E-56	postive
ZNF341	DPP9-AS1	0.612166	8.15E-56	postive
TFAP2E	RFPL3S	0.743405	2.75E-94	postive
TFAP2E	BMS1P4	0.689226	6.55E-76	postive
TFAP2E	C9orf139	0.726595	4.11E-88	postive
TFAP2E	PDXDC2P-NPIPB14P	0.647385	2.81E-64	postive
TFAP2E	RRN3P2	0.712163	3.61E-83	postive
TFAP2E	HCG27	0.614791	2.07E-56	postive
TFAP2E	DPP9-AS1	0.665936	3.35E-69	postive
TFAP2E	DTX2P1-UPK3BP1-PMS2P11	0.638741	4.25E-62	postive
TFAP2E	MIAT	0.623597	1.92E-58	postive
TFAP2E	SNHG3	0.690395	2.90E-76	postive
TFAP2E	ARHGAP27P1-BPTFP1-KPNA2P3	0.708283	6.84E-82	postive
TFAP2E	LINC00115	0.647644	2.42E-64	postive
TFAP2E	LINC00937	0.608971	4.23E-55	postive
TFAP2E	FAM13A-AS1	0.678251	1.13E-72	postive
TFAP2E	INE1	0.627106	2.85E-59	postive
TFAP2E	C3orf35	0.635023	3.51E-61	postive
TFAP2E	N4BP2L2-IT2	0.686078	5.75E-75	postive
TFAP2E	ASMTL-AS1	0.641554	8.46E-63	postive
TFAP2E	LINC00893	0.630868	3.58E-60	postive
TFAP2E	LINC00174	0.6845	1.69E-74	postive
RUNX3	PCED1B-AS1	0.72817	1.13E-88	postive
MXD3	RFPL3S	0.732949	2.16E-90	postive
MXD3	BMS1P4	0.626781	3.40E-59	postive
MXD3	C9orf139	0.68367	2.97E-74	postive
MXD3	PDXDC2P-NPIPB14P	0.672051	6.65E-71	postive
MXD3	RRN3P2	0.6011	2.26E-53	postive
MXD3	LINC00894	0.649777	6.82E-65	postive

MXD3	DPP9-AS1	0.688974	7.80E-76	postive
MXD3	SNHG3	0.774868	########	postive
MXD3	ARHGAP27P1-BPTFP1-KPNA2P3	0.687804	1.75E-75	postive
MXD3	LINC00115	0.713536	1.26E-83	postive
MXD3	FAM13A-AS1	0.642126	6.08E-63	postive
MXD3	SNHG17	0.62159	5.65E-58	postive
MXD3	C3orf35	0.71829	3.14E-85	postive
MXD3	PVT1	0.622252	3.96E-58	postive
MXD3	ASMTL-AS1	0.726635	3.98E-88	postive
MXD3	SCGB1B2P	0.603663	6.27E-54	postive
MXD3	H1-10-AS1	0.631953	1.96E-60	postive
MXD3	LINC00893	0.659038	2.49E-67	postive
MXD3	LINC00174	0.733424	1.45E-90	postive
DLX5	MIR4435-2HG	0.612696	6.19E-56	postive
ZNF83	RFPL3S	0.744241	1.32E-94	postive
ZNF83	GUSBP11	0.7009	1.62E-79	postive
ZNF83	BMS1P4	0.774191	########	postive
ZNF83	C9orf139	0.680892	1.94E-73	postive
ZNF83	PDXDC2P-NPIPB14P	0.798136	########	postive
ZNF83	RRN3P2	0.604794	3.54E-54	postive
ZNF83	LINC02656	0.707621	1.12E-81	postive
ZNF83	HCG27	0.761718	########	postive
ZNF83	LINC00894	0.791774	########	postive
ZNF83	DPP9-AS1	0.723511	4.97E-87	postive
ZNF83	LINC01126	0.647427	2.75E-64	postive
ZNF83	IBA57-DT	0.614468	2.46E-56	postive
ZNF83	DTX2P1-UPK3BP1-PMS2P11	0.671096	1.23E-70	postive
ZNF83	LINC00173	0.681485	1.30E-73	postive
ZNF83	GABPB1-AS1	0.699516	4.42E-79	postive
ZNF83	SNHG3	0.666795	1.94E-69	postive
ZNF83	SSBP3-AS1	0.610844	1.61E-55	postive
ZNF83	ARHGAP27P1-BPTFP1-KPNA2P3	0.793862	########	postive
ZNF83	LINC00115	0.726871	3.28E-88	postive
ZNF83	FAM13A-AS1	0.825292	########	postive
ZNF83	SNHG20	0.659115	2.38E-67	postive
ZNF83	INE1	0.705426	5.78E-81	postive
ZNF83	C3orf35	0.727944	1.37E-88	postive
ZNF83	MALAT1	0.677569	1.78E-72	postive
ZNF83	N4BP2L2-IT2	0.783658	########	postive

ZNF83	ASMTL-AS1	0.768693	########	postive
ZNF83	H1-10-AS1	0.611192	1.35E-55	postive
ZNF83	LINC00893	0.823136	########	postive
ZNF83	LINC00174	0.824809	########	postive
LHX4	BMS1P4	0.628421	1.38E-59	postive
USF1	RFPL3S	0.61586	1.18E-56	postive
USF1	C9orf139	0.661909	4.21E-68	postive
USF1	SNHG3	0.60498	3.23E-54	postive
USF1	ARHGAP27P1-BPTFP1-KPNA2P3	0.605018	3.17E-54	postive
GTF2IRD2	IBA57-DT	0.635968	2.06E-61	postive
GTF2IRD2	LINC00173	0.615323	1.57E-56	postive
GTF2IRD2	SNHG20	0.607226	1.03E-54	postive
GTF2IRD2	LINC00893	0.611044	1.46E-55	postive
GTF2IRD2	LINC00174	0.626559	3.84E-59	postive
ZNF736	BMS1P4	0.615368	1.53E-56	postive
VENTX	C9orf139	0.659542	1.83E-67	postive
VENTX	ARHGAP27P1-BPTFP1-KPNA2P3	0.62853	1.30E-59	postive
CARF	BMS1P4	0.644338	1.68E-63	postive
CARF	HCG27	0.605685	2.26E-54	postive
CARF	GABPB1-AS1	0.617026	6.41E-57	postive
CARF	N4BP2L2-IT2	0.602301	1.24E-53	postive
ETV7	PCED1B-AS1	0.662554	2.81E-68	postive
RFX8	ARHGAP27P1-BPTFP1-KPNA2P3	0.668903	5.06E-70	postive
RFX8	SNHG20	0.618159	3.52E-57	postive
RFX8	H1-10-AS1	0.640233	1.81E-62	postive
TBX21	PCED1B-AS1	0.613785	3.51E-56	postive
MBD6	LINC00174	0.606945	1.19E-54	postive
ZBTB37	BMS1P4	0.672552	4.80E-71	postive
ZBTB37	GABPB1-AS1	0.601601	1.76E-53	postive
ZBTB37	N4BP2L2-IT2	0.604559	3.99E-54	postive
FOXG1	WT1-AS	0.656233	1.39E-66	postive
ZNF587	BMS1P4	0.762784	########	postive
ZNF587	PDXDC2P-NPIPB14P	0.76708	########	postive
ZNF587	HCG27	0.602517	1.11E-53	postive
ZNF587	LINC00894	0.727156	2.60E-88	postive
ZNF587	DTX2P1-UPK3BP1-PMS2P11	0.629974	5.87E-60	postive
ZNF587	SNHG4	0.605391	2.62E-54	postive
ZNF587	GABPB1-AS1	0.731445	7.59E-90	postive

ZNF587	SSBP3-AS1	0.651846	1.98E-65	postive
	ARHGAP27P1-BPTFP1-			
ZNF587	KPNA2P3	0.695366	8.72E-78	postive
ZNF587	LINC00115	0.627847	1.90E-59	postive
ZNF587	FAM13A-AS1	0.646268	5.43E-64	postive
ZNF587	SNHG20	0.638336	5.36E-62	postive
ZNF587	INE1	0.794089	########	postive
ZNF587	LINC00265	0.693948	2.39E-77	postive
ZNF587	MALAT1	0.675571	6.67E-72	postive
ZNF587	N4BP2L2-IT2	0.690194	3.34E-76	postive
ZNF587	ASMTL-AS1	0.660728	8.77E-68	postive
ZNF587	LINC00893	0.7449	7.36E-95	postive
ZNF587	LINC00174	0.675765	5.87E-72	postive
ZNF700	RFPL3S	0.657163	7.89E-67	postive
ZNF700	GUSBP11	0.660094	1.30E-67	postive
ZNF700	BMS1P4	0.794663	########	postive
ZNF700	C9orf139	0.640581	1.48E-62	postive
ZNF700	PDXDC2P-NPIPB14P	0.752429	8.34E-98	postive
ZNF700	LINC02656	0.633522	8.15E-61	postive
ZNF700	HCG27	0.707721	1.04E-81	postive
ZNF700	LINC00894	0.779581	########	postive
ZNF700	DPP9-AS1	0.609615	3.04E-55	postive
ZNF700	LINC01126	0.60205	1.41E-53	postive
ZNF700	DTX2P1-UPK3BP1-PMS2P11	0.64605	6.17E-64	postive
ZNF700	LINC00173	0.631795	2.14E-60	postive
ZNF700	GABPB1-AS1	0.70929	3.20E-82	postive
ZNF700	SSBP3-AS1	0.622523	3.42E-58	postive
	ARHGAP27P1-BPTFP1-			
ZNF700	KPNA2P3	0.773506	########	postive
ZNF700	LINC00115	0.670476	1.84E-70	postive
ZNF700	FAM13A-AS1	0.739275	1.00E-92	postive
ZNF700	SNHG20	0.662775	2.45E-68	postive
ZNF700	INE1	0.714763	4.90E-84	postive
ZNF700	C3orf35	0.646969	3.60E-64	postive
ZNF700	LINC00265	0.606141	1.79E-54	postive
ZNF700	MALAT1	0.699618	4.11E-79	postive
ZNF700	N4BP2L2-IT2	0.766273	########	postive
ZNF700	ASMTL-AS1	0.712135	3.69E-83	postive
ZNF700	LINC00893	0.771865	########	postive
ZNF700	LINC00174	0.757356	########	postive
CCDC17	RFPL3S	0.724008	3.33E-87	postive

CCDC17	GUSBP11	0.786559	########	postive
CCDC17	BMS1P4	0.715992	1.89E-84	postive
CCDC17	C9orf139	0.706886	1.95E-81	postive
CCDC17	PDXDC2P-NPIPB14P	0.814263	########	postive
CCDC17	RRN3P2	0.675583	6.62E-72	postive
CCDC17	LINC02656	0.676569	3.46E-72	postive
CCDC17	HCG27	0.758635	########	postive
CCDC17	LINC00894	0.74777	5.70E-96	postive
CCDC17	DPP9-AS1	0.754049	1.88E-98	postive
CCDC17	IBA57-DT	0.693728	2.79E-77	postive
CCDC17	DTX2P1-UPK3BP1-PMS2P11	0.629108	9.47E-60	postive
CCDC17	LINC00173	0.649324	8.93E-65	postive
CCDC17	GABPB1-AS1	0.671947	7.11E-71	postive
CCDC17	SNHG3	0.646587	4.50E-64	postive
CCDC17	SSBP3-AS1	0.668367	7.13E-70	postive
CCDC17	ARHGAP27P1-BPTFP1-KPNA2P3	0.846908	########	postive
CCDC17	LINC00115	0.819839	########	postive
CCDC17	FAM13A-AS1	0.799791	########	postive
CCDC17	SNHG20	0.767051	########	postive
CCDC17	INE1	0.715957	1.94E-84	postive
CCDC17	C3orf35	0.734788	4.61E-91	postive
CCDC17	MALAT1	0.69701	2.69E-78	postive
CCDC17	N4BP2L2-IT2	0.804643	########	postive
CCDC17	ASMTL-AS1	0.833754	########	postive
CCDC17	H1-10-AS1	0.791163	########	postive
CCDC17	LINC00893	0.800549	########	postive
CCDC17	LINC00174	0.831831	########	postive
ZNF512B	PDXDC2P-NPIPB14P	0.634175	5.65E-61	postive
ZNF512B	ARHGAP27P1-BPTFP1-KPNA2P3	0.605485	2.50E-54	postive
ZNF512B	SNHG20	0.622883	2.82E-58	postive
ZNF512B	INE1	0.651082	3.13E-65	postive
ZNF512B	ASMTL-AS1	0.650406	4.69E-65	postive
ZNF512B	LINC00893	0.617923	3.99E-57	postive
ZNF512B	LINC00174	0.61463	2.26E-56	postive
ZNF224	RFPL3S	0.62712	2.82E-59	postive
ZNF224	GUSBP11	0.661674	4.87E-68	postive
ZNF224	BMS1P4	0.697155	2.43E-78	postive
ZNF224	C9orf139	0.605738	2.20E-54	postive
ZNF224	PDXDC2P-NPIPB14P	0.704049	1.60E-80	postive

ZNF224	LINC02656	0.665279	5.07E-69	postive
ZNF224	HCG27	0.735961	1.71E-91	postive
ZNF224	LINC00894	0.679655	4.45E-73	postive
ZNF224	LINC00173	0.607522	8.88E-55	postive
ZNF224	GABPB1-AS1	0.654178	4.86E-66	postive
ZNF224	SSBP3-AS1	0.606517	1.48E-54	postive
ZNF224	ARHGAP27P1-BPTFP1-KPNA2P3	0.763254	########	postive
ZNF224	LINC00115	0.657694	5.70E-67	postive
ZNF224	FAM13A-AS1	0.756089	2.82E-99	postive
ZNF224	SNHG20	0.643661	2.49E-63	postive
ZNF224	INE1	0.623483	2.04E-58	postive
ZNF224	C3orf35	0.644252	1.77E-63	postive
ZNF224	MALAT1	0.62581	5.77E-59	postive
ZNF224	N4BP2L2-IT2	0.774841	########	postive
ZNF224	ASMTL-AS1	0.669823	2.80E-70	postive
ZNF224	H1-10-AS1	0.618647	2.72E-57	postive
ZNF224	LINC00893	0.727853	1.47E-88	postive
ZNF224	LINC00174	0.707301	1.43E-81	postive
ZNF345	GUSBP11	0.672376	5.39E-71	postive
ZNF345	BMS1P4	0.715009	4.05E-84	postive
ZNF345	PDXDC2P-NPIPB14P	0.723555	4.80E-87	postive
ZNF345	LINC02656	0.617432	5.17E-57	postive
ZNF345	HCG27	0.730065	2.38E-89	postive
ZNF345	LINC00894	0.727812	1.52E-88	postive
ZNF345	DPP9-AS1	0.601484	1.87E-53	postive
ZNF345	LINC01126	0.62739	2.44E-59	postive
ZNF345	IBA57-DT	0.669827	2.80E-70	postive
ZNF345	LINC00173	0.656417	1.25E-66	postive
ZNF345	GABPB1-AS1	0.712628	2.53E-83	postive
ZNF345	SSBP3-AS1	0.657587	6.09E-67	postive
ZNF345	ARHGAP27P1-BPTFP1-KPNA2P3	0.76783	########	postive
ZNF345	LINC00115	0.670786	1.51E-70	postive
ZNF345	FAM13A-AS1	0.694819	1.29E-77	postive
ZNF345	SNHG20	0.741657	1.27E-93	postive
ZNF345	INE1	0.717085	8.06E-85	postive
ZNF345	MALAT1	0.677256	2.19E-72	postive
ZNF345	N4BP2L2-IT2	0.737716	3.82E-92	postive
ZNF345	ASMTL-AS1	0.675847	5.57E-72	postive
ZNF345	H1-10-AS1	0.66582	3.60E-69	postive

ZNF345	LINC00893	0.761468	########	postive
ZNF345	LINC00174	0.706847	2.01E-81	postive
BATF	PCED1B-AS1	0.892812	########	postive
IRF9	RFPL3S	0.716283	1.51E-84	postive
IRF9	GUSBP11	0.755088	7.17E-99	postive
IRF9	BMS1P4	0.802913	########	postive
IRF9	C9orf139	0.773444	########	postive
IRF9	PDXDC2P-NPIPB14P	0.832477	########	postive
IRF9	RRN3P2	0.707594	1.15E-81	postive
IRF9	LINC02656	0.694302	1.86E-77	postive
IRF9	HCG27	0.803829	########	postive
IRF9	LINC00894	0.767463	########	postive
IRF9	DPP9-AS1	0.719793	9.60E-86	postive
IRF9	LINC01126	0.681483	1.31E-73	postive
IRF9	IBA57-DT	0.643014	3.63E-63	postive
IRF9	DTX2P1-UPK3BP1-PMS2P11	0.683173	4.17E-74	postive
IRF9	LINC00173	0.719166	1.58E-85	postive
IRF9	GABPB1-AS1	0.689481	5.48E-76	postive
IRF9	SNHG3	0.712042	3.96E-83	postive
IRF9	SSBP3-AS1	0.718028	3.85E-85	postive
IRF9	ARHGAP27P1-BPTFP1-KPNA2P3	0.863732	########	postive
IRF9	LINC00115	0.812297	########	postive
IRF9	LINC00937	0.614085	3.00E-56	postive
IRF9	FAM13A-AS1	0.82527	########	postive
IRF9	SNHG20	0.782876	########	postive
IRF9	INE1	0.779917	########	postive
IRF9	C3orf35	0.665466	4.51E-69	postive
IRF9	MALAT1	0.716399	1.38E-84	postive
IRF9	N4BP2L2-IT2	0.834356	########	postive
IRF9	ASMTL-AS1	0.820903	########	postive
IRF9	H1-10-AS1	0.717068	8.17E-85	postive
IRF9	LINC00893	0.839643	########	postive
IRF9	LINC00174	0.802713	########	postive
HOXB3	PDXDC2P-NPIPB14P	0.603808	5.83E-54	postive
HOXB3	ARHGAP27P1-BPTFP1-KPNA2P3	0.601661	1.71E-53	postive
HOXB3	ASMTL-AS1	0.611121	1.40E-55	postive
HOXB3	LINC00893	0.605608	2.35E-54	postive
ZNF354B	RFPL3S	0.609331	3.52E-55	postive
ZNF354B	GUSBP11	0.665432	4.60E-69	postive

ZNF354B	BMS1P4	0.738602	1.79E-92	postive
ZNF354B	PDXDC2P-NPIPB14P	0.756045	2.94E-99	postive
ZNF354B	LINC02656	0.663745	1.33E-68	postive
ZNF354B	HCG27	0.71409	8.23E-84	postive
ZNF354B	LINC00894	0.679141	6.27E-73	postive
ZNF354B	DPP9-AS1	0.612168	8.14E-56	postive
ZNF354B	LINC01126	0.663988	1.14E-68	postive
ZNF354B	DTX2P1-UPK3BP1-PMS2P11	0.664822	6.77E-69	postive
ZNF354B	LINC00173	0.612395	7.23E-56	postive
ZNF354B	GABPB1-AS1	0.651724	2.13E-65	postive
ZNF354B	SSBP3-AS1	0.632483	1.46E-60	postive
ZNF354B	ARHGAP27P1-BPTFP1-KPNA2P3	0.758777	########	postive
ZNF354B	LINC00115	0.677726	1.61E-72	postive
ZNF354B	FAM13A-AS1	0.725528	9.77E-88	postive
ZNF354B	SNHG20	0.677036	2.54E-72	postive
ZNF354B	INE1	0.68046	2.60E-73	postive
ZNF354B	MALAT1	0.684079	2.25E-74	postive
ZNF354B	N4BP2L2-IT2	0.760569	########	postive
ZNF354B	ASMTL-AS1	0.680929	1.89E-73	postive
ZNF354B	LINC00893	0.718449	2.77E-85	postive
ZNF354B	LINC00174	0.719126	1.63E-85	postive
ZNF493	BMS1P4	0.610935	1.54E-55	postive
ZNF493	PDXDC2P-NPIPB14P	0.61016	2.30E-55	postive
ZNF493	LINC00894	0.672712	4.33E-71	postive
ZNF493	GABPB1-AS1	0.605495	2.49E-54	postive
ZNF493	INE1	0.633753	7.16E-61	postive
ZNF493	LINC00265	0.677058	2.50E-72	postive
ZNF493	MALAT1	0.644953	1.17E-63	postive
ZNF493	N4BP2L2-IT2	0.62728	2.59E-59	postive
ZNF493	LINC00893	0.638284	5.52E-62	postive
SGSM2	GUSBP11	0.631511	2.50E-60	postive
SGSM2	PDXDC2P-NPIPB14P	0.644456	1.57E-63	postive
SGSM2	LINC00894	0.62706	2.92E-59	postive
SGSM2	ARHGAP27P1-BPTFP1-KPNA2P3	0.676746	3.08E-72	postive
SGSM2	SNHG20	0.652073	1.73E-65	postive
SGSM2	INE1	0.657233	7.56E-67	postive
SGSM2	ASMTL-AS1	0.704342	1.29E-80	postive
SGSM2	H1-10-AS1	0.617526	4.92E-57	postive
SGSM2	LINC00893	0.675133	8.90E-72	postive

SGSM2	LINC00174	0.674763	1.13E-71	postive
